# ω-3 Fatty Acids in Pediatric Major Depressive Disorder: A Randomized Clinical Trial

**DOI:** 10.1001/jamanetworkopen.2025.48703

**Published:** 2026-01-02

**Authors:** Gregor Berger, Isabelle Häberling, Sophie Emery, Mona Albermann, Noemi Baumgartner, Kristin Nalani, Michael Strumberger, Lars Wöckel, Suzanne Erb, Silke Bachmann, Ulrich Müller-Knapp, Brigitte Contin-Waldvogel, Amir Yamini, Bruno Rhiner, Renate Drechsler, Ulrike Held, Kelly Reeve, Priska Heinz, Dagmar Pauli, Klaus Schmeck, Martin Hersberger, Susanne Walitza

**Affiliations:** 1 Department of Child and Adolescent Psychiatry and Psychotherapy, Psychiatric University Hospital, University of Zürich, Zürich, Switzerland; 2 Psychiatry St Gallen, Wil St Gallen, St Gallen, Switzerland; 3 Department of Child and Adolescent Forensic Psychiatry, University Hospital of Psychiatry, Zurich, Switzerland; 4 Department of Clinical Research, University of Basel, Basel, Switzerland; 5 Clienia Littenheid AG, Winterthur, Switzerland; 6 Child and Adolescent Psychiatric Services St Gallen, St Gallen, Switzerland; 7 Hôpitaux Universitaires de Genève/HUG, Geneva, Switzerland; 8 Department of Psychiatry, Psychotherapy and Psychosomatics, University Hospitals Halle (Saale), Halle-Wittenburg, Germany; 9 Child and Adolescent Psychiatry Klinik Sonnenhof, Ganterschwil, Switzerland; 10 Child and Adolescent Psychiatric Services Baselland, Liestal, Switzerland; 11 Child and Adolescent Psychiatric Services Thurgau, Weinfelden, Switzerland; 12 Epidemiology, Biostatistics and Prevention Institute, University of Zurich, Zurich, Switzerland; 13 Center for Integrative Human Physiology, University of Zurich, Zurich, Switzerland; 14 Division of Clinical Chemistry and Biochemistry, University Children’s Hospital Zurich, University of Zurich, Zurich, Switzerland; 15 Neuroscience Center Zurich, University of Zurich and Federal Institute of Technology Zurich, Zurich, Switzerland

## Abstract

**Importance:**

Meta-analyses of ω-3 fatty acids for depression have reported inconsistent results, and pediatric evidence is sparse. Promotion of unproven supplements may delay evidence-based care.

**Objective:**

To evaluate whether adjunctive ω-3 fatty acid supplementation improves outcomes in moderate-to-severe pediatric major depressive disorder (MDD).

**Design, Setting, and Participants:**

In a multicenter, double-blind, placebo-controlled randomized clinical trial at 5 Swiss child and adolescent psychiatry centers, 257 youths with MDD were enrolled and randomized between April 28, 2017, and March 24, 2022, and followed up for 36 weeks, with final analysis from July 1, 2022, to January 26, 2023. Analysis was based on intention to treat.

**Interventions:**

Participants received ω-3 fatty acids, 1.5 g/d (1 g of eicosapentaenoic acid [EPA] and 0.5 g of docosahexaenoic acid [DHA], 2:1 ratio), or medium-chain triglyceride placebo in combination with standardized psychotherapy. Antidepressant use was permitted per national guidelines.

**Main Outcomes and Measures:**

The primary outcome was the trajectory of Children’s Depression Rating Scale–Revised (CDRS-R) scores analyzed with a joint mixed-effects and time-to-event model accounting for dropout or initiation of off-trial antidepressant therapy. Secondary outcomes included response (≥30% reduction in CDRS-R scores), remission (CDRS-R score ≤28), self-rated depression, quality of life, suicidality, and safety.

**Results:**

Of 257 randomized participants (mean [SD] age, 15.7 [1.7] years; 188 [73.2%] female; mean [SD] CDRS-R score, 58.5 [8.8]), 129 received ω-3 supplements and 128 received placebo. The mean (SD) CDRS-R scores decreased similarly in both groups: at 12 weeks, 45.93 (11.98) vs 46.08 (12.99); at 36 weeks, 36.50 (13.12) vs 36.83 (15.46). The adjusted mean difference in CDRS-R scores was 0.77 (95% CI, −1.39 to 2.93; *P* = .49) points. The hazard ratio for time to dropout was 1.22 (95% CI, 0.83-1.79; *P* = .32). Response occurred in 34 of 109 (31.2%) ω-3 recipients vs 43 of 110 (39.1%) placebo recipients at 12 weeks; remission occurred at 36 weeks in 30 of 94 ω-3 recipients (31.9%) vs 37 of 90 (41.1%) placebo recipients (all differences were nonsignificant). Secondary measures and suicidality improved without between-group differences. EPA plus DHA levels expressed by the ω-3 index rose by a mean (SD) of 4.33% (1.54%) and 4.88% (2.38%) at 12 and 36 weeks, respectively, in the ω-3 arm, confirming adherence. A total of 76 serious adverse events were reported in 97 participants, with 31 occurring in the placebo arm and 45 in the ω-3 arm. These included 28 suicide attempts, but no deaths or permanent disabilities. None of these were judged to be causally related to the study medication.

**Conclusions and Relevance:**

In this randomized clinical trial, adjunctive ω-3 administration, 1.5 g/d (EPA:DHA, 2:1), did not outperform placebo in youths with MDD. Future work should assess EPA-enriched formulations and biomarker-guided approaches.

**Trial Registration:**

ClinicalTrials.gov Identifier: NCT03167307

## Key Points

**Question:**

Does adding 1.5 g of ω-3 fatty acids to multimodal treatment improve outcomes in children and adolescents with moderate to severe pediatric major depressive disorder (MDD)?

**Findings:**

In a 36-week randomized clinical trial with 257 participants, ω-3 fatty acids showed no significant benefit over placebo in depression severity, response and remission rates, quality of life, or antidepressant use. Adherence was high, and adverse event rates were similar between groups; approximately one-half of participants still met criteria for moderate to severe pediatric MDD despite treatment.

**Meaning:**

These findings do not support the use of 1.5 g of ω-3 fatty acids as adjunctive therapy for pediatric MDD.

## Teaser

This randomized clinical trial assesses the efficacy of ω-3 fatty acids in reducing depressive symptoms and improving quality of life in children and adolescents with moderate to severe major depressive disorder in Switzerland.

## Introduction

Depression in children and adolescents is a leading contributor to disease burden in this age group and a major modifiable risk factor for suicide, the second leading cause of death among US adolescents.^1^ Approximately 17.0% of US adolescents aged 12 to 17 years reported at least 1 depressive episode in 2020.^2^ Rates are notably higher in female (25.2%) than male adolescents (9.2%). During the past decade, depressive symptoms, self-harm, suicide attempts, and suicides have increased globally among youths, especially females.^3^ Despite the high prevalence, fewer than half (41.6%) of affected adolescents receive appropriate treatment. Early-onset clinical depression also incurs higher costs than adult-onset depression due to the greater psychosocial impact.^4^

ω-3 Fatty acids have gained attention as a possible adjunctive treatment for clinical depression. National surveys show that about 7.4% of US and 9.6% of Swiss citizens take ω-3 supplements, typically for cardiovascular, inflammatory, neurological, or mental health benefits.^5^

Most meta-analyses of randomized clinical trials (RCTs) have found small to moderate improvements in depression symptoms with ω-3 supplements,^6,7,8,9,10,11,12,13,14,15,16,17,18,19,20^ especially in adults with severe depression or when combined with antidepressants.^7,21^ However, more rigorous meta-analyses^17,22,23^ and a large RCT conducted in adolescents and young adults^24^ found no significant benefits, highlighting mixed results.^25^ The present study aimed to assess the efficacy of ω-3 fatty acids in reducing depressive symptoms and improving quality of life in children and adolescents with moderate to severe major depressive disorder (MDD) and whether ω-3 fatty acids could reduce or prevent the need for antidepressant medications, offering insights into both effectiveness and safety in managing pediatric depression.

## Methods

### Study Design and Setting

This investigator-initiated, 36-week, multicenter, double-blind, placebo-controlled phase 3 RCT used 2 parallel groups to test the superiority of ω-3 fatty acid supplementation over placebo. Patients were assigned via dynamic computerized 1:1 randomization with a minimization algorithm based on age, sex, treatment setting (inpatient or outpatient) and high-sensitivity C-reactive protein (hsCRP) levels across 5 Swiss child and adolescent psychiatry centers. Full details of the study rationale, design, and methods, including the trial protocol, statistical analysis plan, and statistical reports, are provided in Supplement 1 and in a previously published clinical trial design paper.^21^

The trial followed the International Council for Harmonisation Good Clinical Practice Guidelines and the ethical principles of the Declaration of Helsinki.^26^ The protocol was approved by the local ethics committee of Kantonale Ethikkommission Zürich . All participants and legal guardians gave written informed consent. We followed the Consolidated Standards of Reporting Trials (CONSORT) reporting guideline.

### Participants and Inclusion Criteria

At inclusion, patients had to be aged 8.0 to 18.0 years and had experienced a moderate to severe MDD episode. A primary diagnosis of MDD (single or recurrent) per *Diagnostic and Statistical Manual of Mental Disorders* (Fourth Edition) criteria was confirmed using the standardized Schedule for Affective Disorders and Schizophrenia for School-Age Children–Present and Lifetime Version interview.^27^ Symptom severity was assessed with the German version of the Children Depression Rating Scale–Revised (CDRS-R).^28^ A total CDRS-R score of 40 or greater (range, 17-113) was required at both the screening visit and after the placebo run-in phase at baseline.

### Treatments

The active supplementation group received 1.5 g of ω-3 polyunsaturated fatty acids daily, including 1000 mg of eicosapentaenoic acid (EPA) and 500 mg of docosahexaenoic acid (DHA). The control group received a placebo with an equivalent amount of medium-chain triglycerides. To reduce gastrointestinal discomfort, children younger than 13 years were given half the dose, following a previous RCT in prepubertal children with depression.^29^ Patients were instructed to take the supplement daily for 36 weeks, ideally with a meal. The placebo capsule contained a trace of fish oil and was encased fish intestine–derived gelatin to mimic the fishy flavor, with both active and placebo capsules flavored artificially. Investigators confirmed that, in terms of appearance, texture, and taste, placebo and active ω-3 capsules were indistinguishable.

During the study, all participants received treatment at official child and adolescent psychiatric services, including both inpatient and outpatient facilities. Clinicians were trained in German evidence and consensus-based guidelines for treating depressive disorders in minors.^30^ To include patients with severe MDD, the use of antidepressants before and/or during the 36-week trial was permitted. This approach helped prevent bias via the exclusion of participants with severe pediatric MDD or those likely to require antidepressants during the trial.

### Outcome Measures

The primary end point was the trajectory of CDRS-R total scores at baseline and 6, 12, 24, and 36 weeks. The first full clinical assessment was scheduled at 6 weeks to minimize participant burden and the risk of early withdrawal while maintaining comparability with the assessment schedule of the Treatment for Adolescents with Depression Study (TADS). Secondary end points included remission (CDRS-R total score, ≤28) at each point and response (30% reduction in CDRS-R total score) at the 6- and 12-week assessments. Additional secondary outcomes were number of participants starting or increasing antidepressant dosage, quality of life measured using the 10-item KIDSCREEN–computerized adaptive test (KIDSCREEN-CAT-10)^31^ and self-rated depression severity using the Depression Inventory for Kids and Youth (DIKJ)^32^ at baseline and at 12 and 36 weeks.

The Suicidal Ideation Questionnaire–Junior (SIQ-Jr)^33^ assessed suicidal ideation at each time point.^34,35^ To monitor medication safety, patients reported adverse events at each visit and completed the general Antidepressant Side Effect Checklist (ASEC).^36^ Severe adverse events were coded using the Medical Dictionary for Regulatory Activities (MedDRA). The Clinical Trials Unit of the University of Zurich supervised the process of data collection and entry, with special attention to serious adverse events.

Researchers estimated adherence by counting returned study capsules at each visit. Additionally, red blood cell levels of ω-3 fatty acids were measured at baseline and at 12 and 36 weeks to assess adherence.^34,35^

### Sample Size

The study was powered to detect a small to medium effect size based on the standardized mean difference derived from a published meta-analysis investigating ω-3 supplementation in MDD.^7^ To achieve 80% power for detecting of a standardized mean difference of 0.4 on the CDRS-R scale at 36 weeks with a 2-sided α = .05 and 10% dropout allowance, 111 patients per arm were required for a 2-sample *t* test. Due to a higher than expected dropout rate, mainly from participants starting antidepressant therapy before 6 weeks (ie, intervention dropouts), the sample size was increased after consulting the drug safety monitoring board to ensure each arm reached 111 patients with at least 1 follow-up.

### Statistical Analysis

Data analysis was conducted from July 1, 2022, to January 26, 2023 (with data coding initiated on March 15, 2022). The primary analysis was based on the full analysis set, defined as all randomized patients, following the intention-to-treat principle. Details are in the statistical analysis plan (Supplement 1), finalized before data export. As per protocol and the statistical analysis plan, data after loss to follow-up or nonadherence (study dropouts) and after introduction or escalation of off-trial antidepressant therapy (intervention dropouts) were treated as missing. These cases were addressed through multiple imputation techniques, as detailed in the analysis descriptions.

The main focus of our study was to estimate the effectiveness of the ω-3 treatment strategy (primary estimand) combined with standard multimodal treatment (treatment policy). This aimed to measure the mean treatment effect of ω-3 supplementation vs placebo during a 36-week period, including all randomized participants regardless of adherence.

The primary analysis used a joint model linking (1) a linear mixed-effects model for CDRS-R total score (time as continuous variable) with (2) a survival model for risk of new initiation of or increase in antidepressant use (intervention dropout) or study dropout. Missingness was assumed at nonrandom, and participants were censored at the earliest of antidepressant initiation or increase, loss to follow-up, withdrawal of consent, or study exit due to nonadherence. The mixed model included baseline minimization factors and unbalanced variables as covariates. A set of sensitivity analyses was prespecified in advance. Treatment effect consistency was tested by adding an interaction term for treatment effect, time, and ω-3 index; post hoc interactions with sex, COVID-19 period, and anxiety were explored. Additional prespecified analyses examined model sensitivity: linear mixed models applied to observed, censored, complete-case, and multiply imputed data. Multiple imputation was performed separately within arms using models with minimization factors, baseline antidepressant use, and the ω-3 index. Continuous secondary end points (KIDSCREEN-CAT-10^31^ and DIKJ^32^) were analyzed using linear regression models with 36-week scores as outcome, treatment as the main factor associated with outcome, and covariates as in the primary analysis plus baseline score. Post hoc, the primary joint model was reapplied. Remission and response were analyzed with logistic regression adjusting for minimization factors, baseline antidepressant use, ω-3 index, and baseline CDRS-R were expressed as adjusted odds ratios.

We used the Kaplan-Meier method to visualize intervention dropout by treatment arm, followed by a Cox proportional hazards regression model that included all covariates from the primary analysis. The risk of additional off-trial antidepressant use was compared between arms using hazard ratios with 95% CIs. Adverse events were summarized as count and percentages for the full sample and for each treatment group (including serious events), as well as based on the ASEC.^36^ All *P* values correspond to hypotheses defined by the statistical analysis plan, 2 sided with 5% type I error rate. Analyses were fully scripted in R, version 4.2.2 (R Foundation for Statistical Computing) with dynamic reporting.

## Results

### Participant Flow

**Figure 1.**
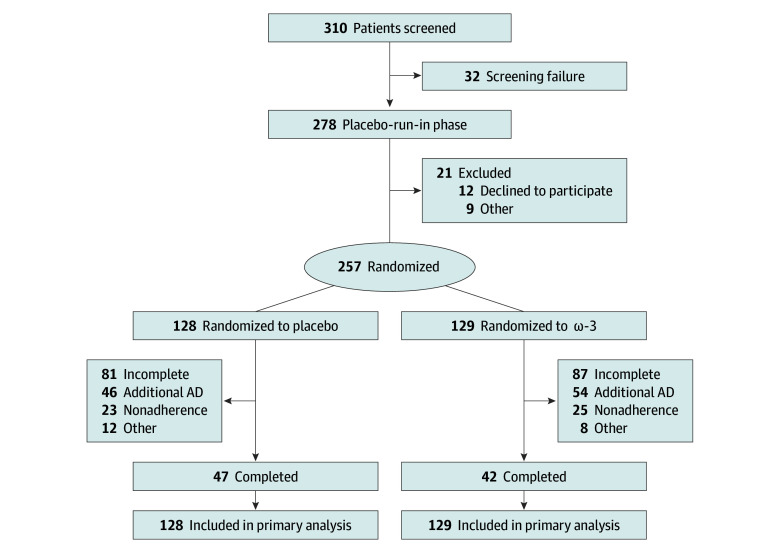
Study Flowchart AD indicates antidepressant.

The study ran from April 28, 2017, to March 24, 2022. Of 310 participants screened, 53 were excluded (32 for screening failure, 12 for lack of informed consent, and 9 for other reasons). A total of 257 participants were randomized: 128 to the placebo arm and 129 to the ω-3 arm, including 188 (73.2%) female and 69 (26.8%) male (mean [SD] age, 15.7 [1.7] years). By the 36-week follow-up, 100 patients initiated antidepressant use or changed their antidepressant dose, 48 were nonadherent, and 20 dropped out for other reasons (Figure 1). All analyses are summarized in the statistical reports in Supplement 1.

A single-blind placebo lead-in phase lasting 1 week was implemented prior to randomization to assess tolerability and reinforce adherence expectations, without stratifying participants based on response. Except for a higher baseline rate of recurrent depression in the ω-3 group, baseline characteristics were balanced between arms (Table 1). A total of 189 participants (73.5%) completed the 36-week follow-up (Figure 1 and eTable 1 in Supplement 2), but many initiated or increased antidepressant use, leading to intervention dropouts.

**Table 1.** Baseline Characteristics

Characteristic	Participant group			Missing, No. (%)
	Overall (N = 257)	Placebo (n = 128)	ω-3 (n = 129)	
Age, mean (SD), y	15.66 (1.66)	15.64 (1.74)	15.69 (1.58)	0
Sex, No. (%)				
Female	188 (73.2)	94 (73.4)	94 (72.9)	0
Male	69 (26.8)	34 (26.6)	35 (27.1)	
BMI, mean (SD)	22.38 (5.28)	22.11 (4.52)	22.65 (5.95)	24 (9.3)
hsCRP level, No. (%)^a^				
Low	179 (69.6)	90 (70.3)	89 (69.0)	0
Medium	53 (20.6)	25 (19.5)	28 (21.7)	
High	25 (9.7)	13 (10.2)	12 (9.3)	
ω-3 Index^b^	4.58 (1.09)	4.54 (1.10)	4.63 (1.08)	0
Recurrent episode, No. (%)				
No	201 (78.2)	107 (83.6)	94 (72.9)	0
Yes	56 (21.8)	21 (16.4)	35 (27.1)	
Age of onset, mean (SD), y	13.79 (2.38)	13.93 (2.27)	13.65 (2.49)	0
Duration of previous episodes, mean (SD), wk	16.28 (17.14)	16.76 (18.98)	15.79 (15.12)	6 (2.3)
Anxiety and/or panic, No. (%)				
No	233 (90.7)	118 (92.2)	115 (89.1)	0
Yes	24 (9.3)	10 (7.8)	14 (10.9)	
Attention-deficit/hyperactivity disorder, No. (%)				
No	220 (85.6)	112 (87.5)	108 (83.7)	0
Yes	37 (14.4)	16 (12.5)	21 (16.3)	
Psychotic features, No. (%)				
No	245 (95.3)	121 (94.5)	124 (96.1)	0
Yes	12 (4.7)	7 (5.5)	5 (3.9)	
Antidepressant use, No. (%)				
No	167 (65.0)	81 (63.3)	86 (66.7)	0
Yes	90 (35.0)	47 (36.7)	43 (33.3)	
Antidepressant used, No. (%)^c^				
Fluoxetine	42 (46.7)	16 (34.0)	26 (60.5)	NA
Sertraline hydrochloride	30 (33.3)	20 (42.6)	10 (23.3)	
Escitalopram	11 (12.2)	6 (12.8)	5 (11.6)	
Other	7 (7.8)	5 (10.6)	2 (4.7)	
CDRS-R score, mean (SD)^d^	58.5 (8.8)	58.1 (8.4)	59.0 (9.2)	0
DIKJ score, mean (SD)^e^	28.09 (9.46)	28.12 (9.91)	28.06 (9.06)	11 (4.3)
KIDSCREEN-CAT-10 score, mean (SD)^f^	29.05 (5.72)	29.25 (5.74)	28.87 (5.71)	17 (6.6)
SIQ-Jr score, mean (SD)^g^	40.91 (23.49)	39.80 (24.31)	41.95 (22.73)	17 (6.6)
EPA level, mean (SD), %^h^	0.40 (0.10)	0.41 (0.10)	0.40 (0.10)	2 (0.8)
DHA level, mean (SD), %^h^	4.18 (1.04)	4.13 (1.05)	4.23 (1.04)	2 (0.8)
Center, No. (%)				
St Gallen outpatient	32 (12.5)	17 (13.3)	15 (11.6)	0
St Gallen Ganterschwil	29 (11.3)	14 (10.9)	15 (11.6)	
Thurgau outpatient	17 (6.6)	10 (7.8)	7 (5.4)	
Thurgau Littenheid	55 (21.4)	27 (21.1)	28 (21.7)	
Baselland	12 (4.7)	6 (4.7)	6 (4.7)	
Basel-Stadt	18 (7.0)	7 (5.5)	11 (8.5)	
Zürich	94 (36.6)	47 (36.7)	47 (36.4)	
Clinic type, No. (%)				
Inpatient	80 (31.1)	39 (30.5)	41 (31.8)	0
Outpatient	164 (63.8)	82 (64.1)	82 (63.6)	
Day unit	13 (5.1)	7 (5.5)	6 (4.7)	

Abbreviations: BMI, body mass index (calculated as weight in kilograms divided by height in meters squared); CDRS-R, Children’s Depression Rating Scale–Revised; DHA, docosahexaenoic acid; DIKJ, Depression Inventory for Kids and Youth; EPA, eicosapentaenoic acid; hsCRP, high-sensitivity C-reactive protein; KIDSCREEN-CAT-10, 10-item KIDSCREEN–computerized adaptive test; NA, not applicable; SIQ-Jr, Suicidal Ideation Questionnaire–Junior.

^a^ Categorized in 3 categories as a proxy of low-grade inflammation: (low [<1], medium [1-3], and high [3-10]).

^b^ Calculated as the sum of eicosapentaenoic acid (EPA) and docosahexaenoic acid (DHA) expressed as a percentage of total identified fatty acids in erythrocyte membranes: *X* = (EPA + DHA)/Total Fatty Acids × 100. Higher ω-3 index values indicate greater incorporation of long-chain ω-3 fatty acids into red-blood-cell membranes, reflecting better long-term ω-3 status. Clinically, higher values have been associated with reduced risk of depressive symptoms and cardiovascular disease, whereas lower values suggest insufficient ω-3 intake or absorption.

^c^ Includes 90 participants.

^d^ Scores range from 17 to 113, with higher scores indicating greater symptom severity.

^e^ Consists of 33 items scored from 0 to 2, yielding a total score between 0 and 66, where higher scores reflect greater depressive symptoms.

^f^ Scores range from 10 to 50, with higher scores indicating better perceived physical, psychological, and social well-being.

^g^ Consists of 15 items, each rated on a 7-point Likert scale from 0 (“I never had this thought”) to 6 (“Almost every day”), yielding a total score from 0 to 90. Higher scores indicate greater severity and frequency of suicidal ideation. A total score of 31 or greater is often used as a clinical cut-off suggestive of elevated suicide risk.

^h^ Reported as percentage of total fatty acids.

### Primary End Point

Depression (CDRS-R score) improved in both groups, with mean (SD) scores decreasing from 58.13 (8.42) in the placebo group and 58.95 (9.15) in the ω-3 group (moderate to severe) at baseline to 36.83 (15.46) and 36.50 (13.12) (mild to moderate), respectively, by 36 weeks (Table 2). The primary analysis adjusted for baseline imbalance in recurrent episodes and modeled dropout events using estimated CDRS-R scores. No evidence of differing trajectories emerged between the ω-3 and placebo arms. The mean difference was 0.77 points (95% CI, –1.39 to 2.93 points; *P* = .49), and the hazard ratio for time to dropout was 1.22 (95% CI, 0.83-1.79; *P* = .32). Results were consistent across all prespecified sensitivity analyses. Regardless of the sensitivity analysis, only small differences in CDRS-R mean scores were found at all time points. The placebo arm generally showed slightly lower scores than the ω-3 arm (indicating less severe depression) in both observed and censored data. When censoring after off-trial antidepressant use or analyzing participants who completed the study per protocol group, differences narrowed. Controlling for antidepressant use or stratifying by first vs recurrent MDD did not change results (eTables 2 to 9 in Supplement 2). Reanalyzing the data for children before and after the 13th birthday did not change the outcomes.

**Table 2.** Continuous Primary and Secondary Outcome Measurements Over Time

Participant group	Baseline	Follow-up, wk^a^			
		6	12	24	36
**CDRS-R score^b^**					
Placebo	58.13 (8.42)	49.03 (12.05)	46.08 (12.99)	42.41 (13.85)	36.83 (15.46)
No. of participants	128	100	79	58	47
ω-3	58.95 (9.15)	49.4 (12.26)	45.93 (11.98)	40.24 (14.06)	36.50 (13.12)
No. of participants	129	90	69	51	42
**DIKJ score^c^**					
Placebo	28.12 (9.91)	25.53 (9.61)	23.03 (11.23)	20.50 (11.87)	18.21 (12.12)
No. of participants	120	97	69	50	42
ω-3	28.06 (9.06)	25.86 (11.24)	22.18 (9.28)	19.78 (11.29)	17.86 (11.04)
No. of participants	126	81	62	45	37
**KIDSCREEN-CAT-10 score** ^d^					
Placebo	29.25 (5.74)	NA	32.26 (7.12)	NA	34.88 (8.36)
No. of participants	115	NA	65	NA	41
ω-3	28.87 (5.71)	NA	32.38 (5.91)	NA	34.28 (7.23)
No. of participants	125	NA	60	NA	36
**SIQ-Jr score** ^e^					
Placebo	39.80 (24.31)	31.86 (20.76)	29.52 (23.46)	27.54 (23.83)	22.95 (22.44)
No. of participants	116	95	71	52	43
ω-3	41.95 (22.73)	33.42 (21.74)	28.25 (17.02)	27.45 (22.92)	20.86 (18.00)
No. of participants	124	83	61	47	35

Abbreviations: CDRS-R, Children’s Depression Rating Scale–Revised; DIKJ, Depression Inventory for Kids and Youth; KIDSCREEN-CAT-10, 10-item KIDSCREEN–computerized adaptive test; NA, not applicable; SIQ-Jr, Suicidal Ideation Questionnaire–Junior.

^a^ Data censored at nonadherence or additional antidepressant use.

^b^ Scores range from 17 to 113, with higher scores indicating greater symptom severity.

^c^ Consists of 33 items scored from 0 to 2, yielding a total score between 0 and 66, where higher scores reflect greater depressive symptoms.

^d^ Scores range from 10 to 50, with higher scores indicating better perceived physical, psychological, and social well-being.

^e^ Consists of 15 items, each rated on a 7-point Likert scale from 0 (“I never had this thought”) to 6 (“Almost every day”), yielding a total score from 0 to 90. Higher scores indicate greater severity and frequency of suicidal ideation. A total score of 31 or greater is often used as a clinical cut-off suggestive of elevated suicide risk.

### Confirmatory Secondary End Points

#### Response and Remission Rates

Logistic regression of remission and response (CDRS-R score), adjusted for age, sex, hsCRP level, off-trial antidepressant use, ω-3 index, and CDRS-R scores at baseline, showed no significant difference between groups. At 36 weeks, remission was achieved in 30 of 91 participants (33.0%) in the ω-3 arm and 37 of 91 (40.7%) in the placebo arm. Similarly, within 12 weeks of the intervention, 34 of 111 participants (30.6%) in the ω-3 arm and 43 of 110 (39.1%) in the placebo arm met response criteria (eTables 10 and 11 in Supplement 2).

#### Self-Rated Emotional Symptoms and Quality of Life

Similar to the primary CDRS-R outcome, KIDSCREEN-CAT-10^31^ and DIKJ^32^ trajectories showed clinically relevant improvement in both groups, with no significant difference between the ω-3 and placebo arms, regardless of analysis method (observed, censored, or completers). Mean differences remained small throughout for KIDSCREEN-CAT-10 (eTables 12-14 in Supplement 2) and KIKJ (eTables 15-17 in Supplement 2).

#### Additional Antidepressant Use

**Figure 2.**
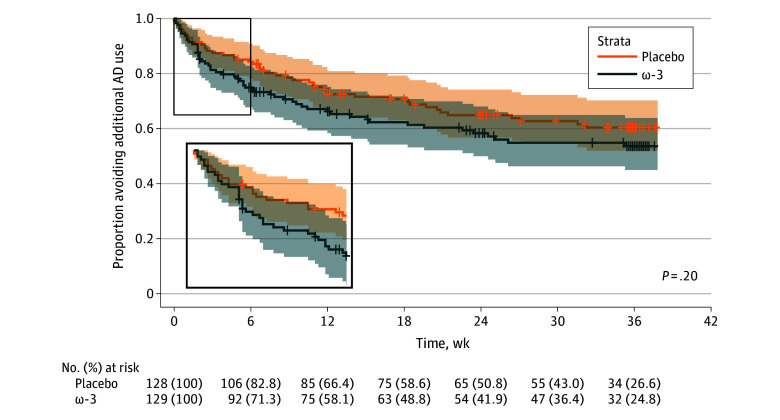
Kaplan-Meier Curve for Avoiding Additional Antidepressant (AD) Use Shaded areas indicate 95% CIs.

Figure 2 shows the probability of avoiding additional antidepressant use over time by treatment arm. The prespecified hypothesis that ω-3 supplementation would reduce off-trial antidepressant use was not supported (Figure 2). A log-rank test showed no significant difference in time to additional antidepressant use between the ω-3 and placebo groups (log rank statistic, 1.6; *P* = .20), although the ω-3 group appeared to receive antidepressants earlier.

A Cox proportional hazards regression model adjusting for age, sex, hsCRP level, baseline antidepressant use, and ω-3 index (eTable 18 in Supplement 2) estimated a 24% higher risk of additional antidepressant use in the ω-3 group compared with the placebo group, although this estimate was not statistically significant (hazard ratio, 1.24; 95% CI, 0.83-1.85; *P* = .30). Interestingly, the HR for risk of requiring additional antidepressant use among those with high hsCRP levels was less than 1, but the result was not statistically significant (hazard ratio, 0.43; 95% CI, 0.17-1.06; *P* = .07).

#### ω-3 Index

Our analysis showed no evidence that the ω-3 index moderated treatment efficacy for pediatric MDD. The longitudinal model, adjusting for treatment, time, and their interaction, along with baseline covariates (age, sex, hsCRP level, and ω-3 index), showed no significant effect. Neither the interaction between ω-3 index and treatment (estimate, 0.35; 95% CI, −0.76 to 1.48; *P* = .54) nor the 3-way interaction with time (estimate, −0.10; 95% CI, −0.23 to 0.03; *P* = .14) was significant (eTables 19-23 in Supplement 2).

#### Safety Measures, Suicidality and Adverse Events

SIQ-Jr score trajectories did not differ between treatment groups (eTables 24-26 in Supplement 2). No differences were found in serious adverse events or in adverse effects assessed with the ASEC between the ω-3 and placebo groups (eTables 27 and 28 in Supplement 2). A total of 76 serious adverse events were reported in 97 participants, with 31 occurring in the placebo arm and 45 in the omega-3 arm. These included 28 suicide attempts, but no deaths or permanent disabilities. None of these adverse events were judged to be causally related to the study medication.

### Adherence

Adherence level was high, confirmed by pill counts and blood test results at 12 and 36 weeks. The ω-3 group showed marked increases in EPA levels (mean [SD], 1.63% [0.67%] at 12 weeks and 1.54% [0.96%] at 36 weeks) and DHA levels, with the ω-3 index rising by 4.33% (1.54%) and 4.88% (2.38%), respectively, reaching the recommended range within 3 months, compared with −0.29% (0.73%) and −0.09% (094%), respectively, in the placebo arm (Table 3), confirming strong adherence and treatment fidelity.

**Table 3.** Percentage Change in ω-3 Fatty Acids Between Baseline and 12 and 36 Weeks

Measurement	Study group					
	Placebo			ω-3		
	Baseline	Follow-up	Change	Baseline	Follow-up	Change
**Change to week 12** ^a^						
EPA level	0.40 (0.09)	0.36 (0.08)	−0.04 (0.08)	0.41 (0.10)	2.04 (0.67)	1.63 (0.67)
DHA level	4.22 (1.13)	3.97 (0.84)	−0.25 (0.70)	4.20 (0.91)	6.90 (1.20)	2.70 (1.03)
ω-3 Index	4.62 (1.18)	4.33 (0.88)	−0.29 (0.73)	4.61 (0.95)	8.94 (1.71)	4.33 (1.54)
**Change to week 36** ^b^						
EPA level	0.41 (0.10)	0.40 (0.27)	−0.01 (0.26)	0.40 (0.11)	1.94 (0.96)	1.54 (0.96)
DHA level	4.40 (1.10)	4.32 (1.02)	−0.08 (0.83)	4.28 (1.00)	7.62 (1.58)	3.34 (1.60)
ω-3 Index	4.81 (1.15)	4.72 (1.13)	−0.09 (0.94)	4.68 (1.04)	9.56 (2.38)	4.88 (2.38)

Abbreviations: DHA, docosahexaenoic acid; EPA, eicosapentaenoic acid.

^a^ Includes 74 participants in the placebo group and 77 in the ω-3 group.

^b^ Includes 64 participants in the placebo group and 69 in the ω-3 group.

## Discussion

Our study found no statistically significant benefit of ω-3 supplementation (1000 mg of EPA and 500 mg of DHA) as an adjunct to multimodal treatment for pediatric MDD compared with placebo, despite a marked increase in ω-3 status (ω-3 index >8% by trial end). This finding was consistent across all outcomes—symptom trajectories, remission and response rates, additional antidepressant use, and quality of life measures—regardless of analytical approach.

Compared with prior RCTs in adults^18^ and youths,^21,25^ our study was larger and longer, addressing limitations of earlier studies that reported mixed results.^25^ Nemets et al^29^ and Trebatická et al^37^ observed benefits of ω-3, but their placebo groups showed no improvement, a uncommon pattern in depression trials, possibly due to ω-6–based placebos (with potentially proinflammatory properties), which may not have served as neutral comparators. Conversely, Fristad et al^38^ reported effects in youths with comorbid attention-deficit/hyperactivity disorder, while Gabbay et al^39^ found none.

Our study uniquely examined a clinical, help-seeking population with moderate to severe MDD, without excluding patients who expressed thoughts of suicide. Baseline suicidality was markedly higher than in the TADS (mean [SD] baseline SIQ-Jr, 41 [23] vs 16 [12]).^40^ Remission rates in the current trial were lower than those reported in TADS. At 12 weeks, remission was observed in 6.3% of participants in the ω-3 group and 14.7% in the placebo group, compared with 23% in TADS. By 36 weeks, remission increased to 33.0% (ω-3 group) and 40.7% (placebo group), whereas 60% remission was reported in TADS.^40^ This difference, along with potential factors such as increased social media use and pandemic-related stressors may explain outcome disparities. Notably, more than one-third of participants initiated guideline-concordant selective serotonin reuptake inhibitor therapy within 6 weeks yet showed only modest gains, underscoring the limited effectiveness of current pharmacological strategies for severe pediatric MDD and the urgent need to identify treatment-resistant trajectories earlier.

Environmental factors such as social media use, recreational screen time, sleep, and physical as well as social activities are well-documented moderators and mediators of adolescent depression.^41^ These influences may not respond to biological interventions such as ω-3 supplementation or selective serotonin reuptake inhibitors. Additionally, COVID-19 introduced potential confounders affecting depression severity and course.^42^ However, controlling for the time of study completion did not alter results (eTables 21 and 22 in Supplement 2).

Despite widespread belief in the mood-enhancing properties of ω-3 fatty acids, supported by multiple meta-analyses^6,7,8,9,10,11,12,13,14,15,16,17^ and expert panels,^43^ our findings emphasize the need for rigorous evaluation of even seemingly benign interventions. Promoting ineffective treatments risks delaying effective care and may worsen outcomes for adolescents with depression. Such delays can perpetuate the chronicity in which depressive disorders increase the risk of future suicide.^1^

Recent research on ω-3 fatty acids and suicidality has shown mixed results.^44^ Some studies suggest low levels of ω-3 polyunsaturated fatty acids, particularly EPA and DHA, may increase suicide risk through pathways involving inflammatory mediators.^45^ However, larger studies, such as those conducted by the Harvard School of Public Health,^46^ report no significant protective effect. Consistent with these findings, our trial found no evidence that ω-3 fatty acids influenced suicidality during the 36-week study duration (eTables 24-26 in Supplement 2). The discrepancy between clinical and epidemiological studies highlights the need for further research in specific subgroups or in combination with other interventions.

Unexpectedly, only a small number of participants had an ω-3 index of less than 3.2%, limiting our ability to examine whether marked deficiency modulates treatment response. Neuroimaging research suggests ω-3–related neuroprotective effects,^47^ particularly on prefrontal and anterior cingulate circuitry, that are most evident when baseline levels are very low.^47,48^ Importantly, the substantial increase of the ω-3 index by 4.33 and 4.88 percentage points in the ω-3 arm at weeks 12 and 36, respectively, compared with 0.4 percentage points or less in the placebo arm confirms excellent adherence to the study medication. Subgroup analyses by age, sex, baseline severity, and inflammatory status did not reveal any significant treatment-by-subgroup interactions, suggesting that the null findings were consistent across clinically relevant strata.

The low prevalence of elevated hsCRP levels in our sample suggests that low-grade inflammation may play a lesser role in adolescent than in adult depression (although assessment of other inflammatory markers is pending).^49^ Future research should investigate whether low-grade inflammation in adolescent depression might be secondary to factors such as the chronicity of illness or comorbidities such as obesity.

### Limitations

This study has some limitations. The multicenter, clinical design—including inpatient and outpatient settings with limited restrictions on standard care—enhances generalizability but introduces heterogeneity in treatment exposure. This approach mirrors clinical practice yet may dilute detectable adjunctive effects of ω-3 supplementation. The COVID-19 pandemic likely influenced the outcomes, despite efforts to adjust for timing effects. ω-3 Dose-response characteristics could not be addressed in our study design, as we used a fixed dose, but we cannot discount the possibility that different ω-3 fatty acid doses or ratios might have antidepressive effects. Antidepressant use may have increased placebo response rates; however, more antidepressants were prescribed in the ω-3 group, although the difference was not significant (Figure 2), contradicting this concern. We did not control for media use, a key moderator of adolescent depression, a factor that was not that strong when we designed the study. Baseline assessment with the Swiss ω-3 PUFA (Polyunsaturated Fatty Acid) Food-Frequency Questionnaire showed no significant differences in ω-3 intake between groups.^50^ However, we cannot rule out other dietary influences requiring broader dietary assessments with gut-microbiome profiling to clarify how nutrition-microbiota interactions may shape mental-health trajectories.^51^

## Conclusions

In this RCT that included help-seeking children and adolescents with moderate-to-severe MDD receiving professional multimodal treatment, we found no evidence that ω-3 supplementation provided benefit compared with placebo. Furthermore, ω-3 supplementation neither reduced antidepressant use nor conferred protection against suicidality. Future studies may need to focus on more specific subgroups, such as those with recurrent depression or inflammation-linked depression, while also considering the influence of modern psychosocial stressors like social media use.

## Notes

Supplement 1.
Trial Protocol, Statistical Analysis Plan, and Statistical Reports

Supplement 2. **eTable 1**. Participants Retention Rate **eTable 2**. Primary Analysis Results From Joint Modeling of the Primary Outcome and Time to Dropout **eTable 3**. Sensitivity Analysis I (Data as Observed): Adjusted Linear Mixed Model Results for the Relationship Between Treatment Arm and CDRS Slopes, Including Random Intercepts for Participant and Center **eTable 4**. Sensitivity Analysis I (Data as Observed): Adjusted Linear Mixed Model Results for the Relationship Between Treatment Arm and CDRS Slopes, Including Random Intercepts for Participant and Center and Random Slopes for Participants **eTable 5**. Sensitivity Analysis II (Censored Observations After AD Use): Adjusted Linear Mixed Model Results for the Relationship Between Treatment Arm and CDRS Slopes, Including Random Intercepts for Participant and Center **eTable 6**. Sensitivity Analysis II (Censored Observations After AD Use): Adjusted LLMl results for the Relationship Between Treatment Arm and CDRS Slopes, Including Random Intercepts for Participant and Center and Random Slopes for Participants **eTable 7**. Sensitivity Analysis III (per Protocol Data): Adjusted Linear Mixed Model Results for the Relationship Between Treatment Arm and CDRS Slopes, Including Random Intercepts for Participant and Center **eTable 8**. Sensitivity Analysis III (per Protocol Data): Adjusted LMM Results for the Relationship Between Treatment Arm and CDRS Slopes, Including Random Intercepts for Participants and Center and Random Slopes for Participants **eTable 9**. Sensitivity Analysis IV: Delta-Based Multiple Imputation for MNAR Analysis **eTable 10**. Remission Status per Visit **eTable 11**. Response Status per Visit **eTable 12**. Results From Joint Modeling of the Outcome KIDSCREEN-CAT-10 and Time to Dropout **eTable 13**. ANOVA of KIDSCREEN-CAT-10 After 36 Weeks, Adjusted for Age, Sex, hsCRP, Antidepressants, ω-3 Index, and KIDSCREEN-CAT-10 at Baseline **eTable 14**. ANOVA of Self-Reported Quality of Life Measured by the KIDSCREEN-CAT-10 at Week 36, Adjusted for Age, Sex, hsCRP, Antidepressants, ω-3 Index, and KIDSCREEN-CAT-10 at Baseline **eTable 15**. Results From Joint Modeling of the Outcome DIKJ and Time to Dropout **eTable 16**. ANOVA of DIKJ After 36 Weeks, Adjusted for Age, Sex, hsCRP, Antidepressants, ω-3 Index, and DIKJ at Baseline **eTable 17**. ANOVA of Depression Severity Measured by DIKJ at Week 36, Adjusted for Age, Sex, hsCRP, Antidepressants, ω-3 Index, and DIKJ at Baseline **eTable 18**. Cox Proportional Hazards Regression Results for the Relationship Between Treatment Arm and Time to Additional Antidepressant Use **eTable 19**. Results From Joint Modeling of the Primary Outcome and Time to Dropout, Including an Interaction Term Between Among Treatment Effect, Time, and ω-3 Index **eTable 20**. Results From Joint Modeling of the Primary Outcome and Time to Dropout, Including an Interaction Term Between Group and Sex **eTable 21**. Results From Joint Modeling of the Primary Outcome and Time to Dropout, Including an Interaction Term Between Group and Before or After COVID-19 Pandemic **eTable 22**. Results From Joint Modeling of the Primary Outcome and Time to Dropout, Including an Interaction Term Between Group and Influence of COVID-19 Restrictions **eTable 23**. Results From Joint Modeling of the Primary Outcome and Time to Dropout, Including an Interaction Term Between Group and Anxiety and/or Panic **eTable 24**. Results From Joint Modeling of the Outcome SIQ-Jr and Time to Study Dropout **eTable 25**. Safety Analysis (Censored Observations After AD Use): Adjusted Linear Mixed Model Results for the Relationship Between Treatment Arm and SIQ-Jr Slopes **eTable 26**. Sensitivity Analysis IV, Delta-Based Multiple Imputation for MNAR Analysis **eTable 27**. Severe Adverse Events **eTable 28**. Adverse Events

Supplement 3.
Nonauthor Collaborators

Supplement 4.
Data Sharing Statement
