# Supplementary material for: ω-3 Fatty Acids in Pediatric Major Depressive Disorder: A Randomized Clinical Trial
Source: JAMA Netw Open. 2026 Jan 2;9(1):e2548703. doi: 10.1001/jamanetworkopen.2025.48703 (PMC12761337; doi:10.1001/jamanetworkopen.2025.48703)
Supplement: Supplement 1. — Trial Protocol, Statistical Analysis Plan, and Statistical Reports [file jamanetwopen-e2548703-s001.pdf]

Supplement 2-6 Table of content

2 Supplement 2 Study protocol Omega3-pmdd study (version 6; date 2021_03_09)

3 Supplement 3 Statistical Analysis Plan SAP Omega3-pMDDstudy (date 2022_05_31)

4 Supplement 4 Statistical Report SR Omega3-pMDD (version 4.1; date October 21, 2024)

5 Supplement 5 Statistical Report SR Omega3_pMDD amendment1_Fatty acid levels (date 2024_11_11)

6 Supplement 6 Statistical Report SR_omega3_amendment2_Reanalyzing data children below_above 13 years of age (date 2025_08_04)

Psychiatrische
Universitätsklinik Zürich

Clinical Study Protocol

OMEGA-3 FATTY ACIDS AS FIRST-LINE TREATMENT IN PAEDIATRIC DEPRESSION.

A phase III, 36-week, multi-centre, double-blind, placebo-controlled randomized superiority Study.

The Omega-3-pMDD Study

**Study Type:**	Intervention with Investigational Medicinal Product (IMP)
**Study Categorisation:**	Clinical Trial with IMP Category C
**Study Registration:**	Swiss Federal Complementary Database<br>Clinicaltrials.gov
**Study Identifier:**	SNF 33IC30_166826
**Sponsor, Sponsor-Investigator and Principal Investigator:**	Gregor Berger<br>Department of Child and Adolescent Psychiatry<br>University Hospital of Psychiatry<br>University of Zurich<br>Neumünsterallee 9

CH 8032 Zürich

Switzerland

Phone: +41 43 499 26 71

Mobile: +41 76 464 61 54

E-Mail: gregor.berger@puk.zh.ch

Investigational Product: Omega-3 fatty acids

(1000mg EPA / 500mg DHA in > in 13 years old and 500mg EPA / 250mg DHA in < in 13 years old)

Protocol Version and Date: Version 6 of 09.03.2021

CONFIDENTIAL

The information contained in this document is confidential and the property of the Department of Child and Adolescent Psychiatry of the University of Zurich. The information may not - in full or in part - be transmitted, reproduced, published, or disclosed to others than the applicable Independent Ethics Committee(s) and Competent Authority(ies) without prior written authorization from the Department of Child and Adolescent Psychiatry of the University of Zurich, except to the extent necessary to obtain informed consent from those participants who will participate in the study.

SIGNATURE PAGES

**Study number**	Swiss Federal Complementary Database
**Study Title**	**Omega-3 fatty acids as first-line treatment in Paediatric Depression. A phase III, 36-week, multi-centre, double-blind, placebo-controlled randomized superiority Study.**

Sponsor-Investigator (Principal Investigator):

The Sponsor-Investigator and trial statistician have approved the protocol version 6 of 09.03.2021, and confirm hereby to conduct the study according to the protocol, current version of the World Medical Association Declaration of Helsinki, ICH-GCP guidelines and the local legally applicable requirements.

Gregor Berger

Place/Date

Signature

Trial Statistican:

The Sponsor-Investigator and trial statistician have approved the protocol version 6 of 09.03.2021, and confirm hereby to conduct the study according to the protocol, current version of the World Medical Association Declaration of Helsinki, ICH-GCP guidelines and the local legally applicable requirements.

Ulrike Held

____________________________ _______________________________________

Place/Date Signature

Local Principal Investigator at study site:

I have read and understood this trial protocol and agree to conduct the trial as set out in this study protocol, the current version of the World Medical Association Declaration of Helsinki, ICH-GCP guidelines and the local legally applicable requirements.

**Site**	**Zürich**<br>Department of Child and Adolescent Psychiatry<br>University Hospital of Zurich<br>University of Zurich<br>Neumünsterallee 9<br>8032 Zürich
**Principal investigator**	**Susanne Walitza**

________________________ __________________________________

Place/Date Signature

Local Principal Investigator at study site:

I have read and understood this trial protocol and agree to conduct the trial as set out in this study protocol, the current version of the World Medical Association Declaration of Helsinki, ICH-GCP guidelines and the local legally applicable requirements.

**Site**	**Basel-Stadt**<br>Department of Child and Adolescent Psychiatry<br>Universitäre Psychiatrische Kliniken (UPK)<br>Schaffhauserrheinweg 55<br>4058 Basel
**Principal investigator**	**Klaus Schmeck**

______________________________ ______________________________________

Place/Date *Signature*

Local Principal Investigator at study site:

I have read and understood this trial protocol and agree to conduct the trial as set out in this study protocol, the current version of the World Medical Association Declaration of Helsinki, ICH-GCP guidelines and the local legally applicable requirements.

**Site**	**Thurgau**<br>Clienia Littenheid AG<br>Privatklinik für Psychiatrie und Psychotherapie<br>9573 Littenheid
**Principal investigator**	**Lars Wöckel**

Place/Date

Signature

Local Principal Investigator at study site:

I have read and understood this trial protocol and agree to conduct the trial as set out in this study protocol, the current version of the World Medical Association Declaration of Helsinki, ICH-GCP guidelines and the local legally applicable requirements.

**Site**	**St. Gallen**<br>Child and Adolescent Psychiatric Services St. Gallen<br>Brühlgasse 35/37<br>Postfach 447<br>9004 St. Gallen
**Principal investigator**	**Suzanne Erb**

Place/Date

Signature

Local Principal Investigator at study site:

I have read and understood this trial protocol and agree to conduct the trial as set out in this study protocol, the current version of the World Medical Association Declaration of Helsinki, ICH-GCP guidelines and the local legally applicable requirements.

**Site**	**St. Gallen**<br>Klinik Sonnenhof<br>Sonnenhofstrasse 15<br>9608 Ganterschwil
**Principal investigator**	**Ulrich Müller-Knapp**

________________________ __________________________________

Place/Date Signature

Local Principal Investigator at study site:

I have read and understood this trial protocol and agree to conduct the trial as set out in this study protocol, the current version of the World Medical Association Declaration of Helsinki, ICH-GCP guidelines and the local legally applicable requirements.

**Site**	**Thurgau**<br>Child and Adolescent Psychiatric Services<br>Schützenstrasse 15<br>8570 Weinfelden
**Principal investigator**	**Bruno Rhiner**

Place/Date   Signature

Local Principal Investigator at study site:

I have read and understood this trial protocol and agree to conduct the trial as set out in this study protocol, the current version of the World Medical Association Declaration of Helsinki, ICH-GCP guidelines and the local legally applicable requirements.

**Site**	**Basel-Land**<br>Psychiatrie Baselland<br>Bienentalstrasse 7<br>4410 Liestal
**Principal investigator**	**Brigitte Contin-Waldvogel**

__________________________ ____________________________________

Place/Date Signature

Coordinating investigator

I have read and understood this trial protocol and agree to conduct the trial as set out in this study protocol, the current version of the World Medical Association Declaration of Helsinki, ICH-GCP guidelines and the local legally applicable requirements.

Gregor Berger

Place/Date Signature

TABLE OF CONTENTS

SIGNATURE PAGES 3
STUDY SYNOPSIS 17
ABBREVIATIONS 22
STUDY SCHEDULE 27
1 STUDY ADMINISTRATIVE STRUCTURE 29
1.1 Sponsor, Sponsor-Investigator 29
1.2 Principal Investigator(s) 29
1.3 Neuropsychology 31
1.4 Coordinating Investigator 32
1.5 Statistician (“Biostatistician”) 32
1.6 Laboratory (if applicable) 32
1.7 Monitoring Institution 33
1.8 Independent Data Monitoring Committee 34
1.9 Any other relevant Committee, Person, Organisation, Institution 34
2 ETHICAL AND REGULATORY ASPECTS 35
2.1 Study Registration 35
2.2 Categorisation of Study 35
2.3 Competent Ethics Committee (CEC) 35
2.4 Competent authority (CA) 35
2.5 Ethical Conduct of the Study 36
2.6 Declaration of Interest 36
2.7 Patient Information and Informed Consent 36
2.8 Participant Privacy and Confidentiality 37
2.9 Early Termination of the Study 37
2.10 Protocol Amendments 38
3 INTRODUCTION 39
3.1 Background and Rationale 39
3.1.1. Adult and paediatric major depressive disorders 39
3.1.2 MDD and suicidality 39
3.1.3 The importance of Omega-3 fatty acids 41
3.1.4 Dietary intake of Omega-3 fatty acids and MDD 41
3.1.5 Omega-3 fatty acids deficiency in MDD 41
3.1.6 Inflammatory mediators, Omega-3 fatty acids and MDD 42
3.2 Investigational Product and Indication 42
3.3 Preclinical Evidence 42
3.4 Clinical Evidence to Date 44
3.4.1 Omega-3 fatty acids RCTS in MDD and related disorders 44
3.4.2 Omega-3 fatty acids RCTs in MDD 44
3.4.3 Omega-3 fatty acids RCTs in minors 45
3.4.4 Omega-3 fatty acids RCTs in other psychiatric conditions 46
3.4.5 Unpublished RCTs with Omega-3 fatty acids in pMDD 46

3.5 Dose Rationale....47
3.6 Explanation for Choice of Comparator (or Placebo) 47
3.7 Risk / Benefits 48
3.8 Justification of Choice of Study Population....50
4 STUDY OBJECTIVES....50
4.1 Overall Objective....50
4.2 Primary Objective....50
4.3 Secondary Objectives 51
4.4 Safety Objectives 51
5 STUDY OUTCOMES 51
5.1 Primary Outcome 51
5.2 Secondary Outcomes....51
5.3 Other Outcomes of Interest 53
5.4 Safety Outcomes....53
6 STUDY DESIGN AND COURSE OF STUDY....53
6.1 General Study Design and Justification of the Design 53
6.2 Study Duration and Study Schedule....55
6.3 Methods of Minimising Bias....55
6.3.1 Randomisation....55
6.3.2 Other Methods of Minimising Bias....56
6.4 Unblinding Procedures (Code break) 57
7 STUDY POPULATION....58
7.1 Eligibility Criteria....58
7.1.1 Inclusion Criteria....58
7.1.2 Exclusion Criteria....59
7.2 Recruitment and Screening....60
7.2.1 Incentives 61
7.3 Assignment to Study Groups....61
7.4 Criteria for Withdrawal/ Discontinuation of Participants 62
8 STUDY INTERVENTION....63
8.1 Identity of Investigational Product(s)....63
8.1.1 Experimental Intervention 63
8.1.2 Control Intervention 63
8.1.3 Packaging, Labelling and Supply (Re-Supply) 64
8.1.4 Storage Conditions 65
8.2 Administration of Experimental and Control interventions....65
8.2.1 Experimental Intervention 65
8.2.2 Control Intervention 65
8.3 Dose Modifications....65
8.4 Compliance with Study Intervention 66
8.5 Data Collection and Follow-up for Withdrawn Participants 66
8.6 Trial Specific Preventive Measures 66
8.7 Concomitant Intervention(s) 67
8.7.1 Hospitalisation 67

8.7.2 Starting an antidepressant 67
8.7.3 Use of sedative medications (Benzodiazepine, antipsychotics) 67
8.8 Study Drug Accountability 68
8.9 Return or Destruction of Study Drug 68
9 STUDY ASSESSMENTS 68
9.1 Study Flow Chart(s)/Table of Study Procedures and Assessments 68
9.2 Assessments of Outcomes 70
9.2.1 Assessment of Primary Outcome 70
9.2.2 Assessment of Secondary Outcomes 70
9.2.3 Assessment of Other Outcomes of Interest 74
9.2.4 Assessment of Safety Outcomes 75
9.3 Procedures at Each Visit 76
9.3.1 Screening and lead-in phase 76
9.3.2 Visit 1: baseline visit 77
9.3.3 Visit 2: 6 weeks 77
9.3.4 Visit 3: 12 weeks 78
9.3.5 Visit 4: 24 weeks 78
9.3.6 Visit 5: 36 weeks 78
9.3.7 Clinical Care and monitoring of clinical visits 78
10 SAFETY 78
10.1 Definition of (Serious) Adverse Events and Other Safety Related Events 78
10.2 Recording of (Serious) Adverse Events and Other Safety Related Events 80
10.3 Assessment of (Serious) Adverse Events and Other Safety Related Events 81
10.4 Reporting of Serious Adverse Events and Other Safety Related Events 81
10.5 Follow up of (Serious) Adverse Events 83
11 STATISTICAL METHODS 84
11.1 Hypothesis 84
11.2 Determination of Sample Size 84
11.3 Statistical Criteria of Termination of Trial 85
11.4 Planned Analyses 85
11.4.1 Datasets to be Analysed, Analysis Populations 85
11.4.2 Primary Analysis 86
11.4.3 Secondary Analyses 87
11.4.4 Interim Analyses 88
11.4.5 Safety Analysis 88
11.4.6 Deviation(s) from the Original Statistical Plan 89
11.5 Handling of Missing Data and Drop-Outs 89
12 ELIGIBILITY OF THE PROJECT SITE(S) 89
13 DATA QUALITY ASSURANCE AND CONTROL 89
13.1 Data Handling and Record Keeping / Archiving 90
13.1.1 Case Report Forms 90
13.1.2 Specification of Source Documents 91
13.1.3 Record Keeping / Archiving 91
13.2 Data Management 91

13.2.1 Data Management System..92
13.2.2 Data Security, Access and Back-up..92
13.2.3 Analysis and Archiving...92
13.2.4 Electronic and Central Data Validation..93
13.3 Monitoring 93
13.4 Audits and Inspections 93
13.5 Confidentiality, Data Protection 94
13.6 Storage of Biological Material and Related Health Data 94
14 PUBLICATION AND DISSEMINATION POLICY 94
15 FUNDING AND SUPPORT 95
15.1 Funding 95
15.2 Other Support 95
16 INSURANCE 95
17 REFERENCES 97

STUDY SYNOPSIS

**Sponsor / Sponsor-Investigator**	**Gregor Berger**<br>Department of Child and Adolescent Psychiatry<br>University Hospital of Zurich<br>University of Zurich<br>Neumünsterallee 3<br>Postfach 1482<br>8032 Zürich<br>Switzerland
**Study Title:**	**Omega-3 fatty acids as first-line treatment in paediatric depression. A phase III, 36-week, multi-centre, double-blind, placebo-controlled randomized superiority Study.**
**Short Title / Study ID:**	Omega-3-pMDD
**Protocol Version and Date:**	Version 6 of 09.03.2021
**Trial registration:**	Swiss Federal Complementary Database<br>Clinicaltrials.gov
**Study category and Rationale**	Clinical Phase with IMP Category C
**Clinical Phase:**	Clinical Phase III
**Background and Rationale:**	About 10% teenagers report moderate to marked depressive symptoms and between 1-6% will develop a paediatric major depressive disorder (pMDD) until adulthood. However, evidence-based treatment approaches are sparse, and the use of SSRIs is heavily debated due to reports of an increase in suicidal ideation and limited efficacy in this age group. Growing evidence suggests that omega-3 fatty acids may be a beneficial treatment in adult MDD (aMDD) with no published study in teenagers, despite of its face validity as a valuable first-line treatment. Meta-analyses of published randomized controlled trials (RCTs) in aMDD show moderate effect sizes, if the proportion of eicosapentaenoic acid (EPA) is >60% of the total omega-3 fatty acids. One small RCT in prepubertal children shows an even larger effect size in favour of omega-3 fatty acids. Higher inflammatory mediators (e.g. c-reactive protein, interleukins and others) have been reported in aMDD and pMDD. Preliminary data suggests that a pro-inflammatory state may serve as predictor for omega-3 fatty acids response. Furthermore, low levels of omega-3 fatty acids have been found in aMDD and pMDD potentially also serving as EPA-response predictors. As MDD is a heterogeneous disease entity, such response predictors should be incorporated into MDD RCTs.

**Objective(s):**	1) To investigate the therapeutic efficacy and safety of omega-3 fatty acids rich in EPA in pMDD, 2) to demonstrate clinical meaningful effects of omega-3 fatty acid treatment, 3) to investigate inflammatory and bioactive lipid markers as response predictors, and 4) to investigate the relationship between psychopathology (in particular suicidal ideation), illness course and cognition in relation to inflammatory and bioactive lipid markers. 5.) To establish a tissue repository of phenotypically well characterised children and adolescents with pMDD.
**Outcome(s):**	The German S3 Guidelines for the treatment of depression in children and youth define the background treatment for all participants. All clinical partners will be trained and monitored accordingly. The primary outcomes are the (continuous) Children's Depression Rating Scale-revised (CDRS-R) total score and the (dichotomous) rates of recovery defined by the absence of pMDD for >4months at 36 weeks, as well as response and remission rates at 12 and 36 weeks. Inflammatory mediators in serum using immunoassays, red blood cell omega-3, 6, 9 and trans fatty acids using gas chromatography (GC) and bioactive lipid mediators (e.g. E-series resolvins) using mass spectrometry (LC-MS/MS) will be measured as potential response predictors. Adverse events/ harm endpoints (in particular suicidality) will be coded using MedDRA. Adherence measurements are pill counts, as well as n-3 EPA/DHA levels across the study. Blood samples will be taken at study entry, week 12 and 36.
**Study design:**	A Swiss, multicentre, randomised, double-blind, placebo-controlled clinical trial.
**Inclusion / Exclusion criteria:**	The study aims to randomize a sample of 220 individuals aged 8 - 17 years, who are in- or outpatients of a participating centre and have a present primary diagnosis of major depression disorder with depressive symptoms of at least moderate severity. Participants with pre-existing neurological or medical conditions likely to be responsible for the depressive symptoms or other psycho-pathological diagnoses are excluded.
**Measurements and procedures:**	The study design incorporates a 1-2week screening, a 7 -10days placebo lead-in and a 36week double-blind placebo-controlled treatment phase. The severity of the depression and psychosocial functioning will be assessed at baseline and at each study visit (twice in the acute phase and twice in the maintenance phase) using a variety of different questionnaires and rating scales. Cognitive testing and biological markers (blood, urine and saliva) will be sampled at baseline and at 12 and 36 weeks. Adherence to the study will be checked by pill count at each study visit and PUFA level measurements in red blood cell membranes at baseline, 12 and 36 weeks will be performed.

**Study Product / Intervention:**	In the proposed study we use for the active treatment a daily dose of 500mg EPA/ 250mg DHA in our 8 to <13 year olds, and 1000mg EPA / 500mg DHA in our 13 to <18 years olds (which corresponds with the omega-3 fatty acid doses used in adult MDD RCTs). The drug will be administered for 36 weeks.
**Control Intervention (if applicable):**	Placebo capsules will contain mostly medium chain triglycerides (MCT) and each capsule will also contain 1.6mg of fish oil to mimic the fishy flavour and taste.
**Number of Participants with Rationale:**	We aim to randomize 220 participants with at least one follow-up assessment in total, resulting in 110 participants per treatment group. Furthermore, we will recruit up to 20 additional patients to account for the number of patients who started antidepressant within the first 6 weeks of treatment. We performed a sample size calculation based on the effect size of 0.54 found in a previous meta-analysis on the effect of omega-3 fatty acids in aMDD. Sample size calculations were then adjusted for a higher placebo-response rate in minors given our multi-centre study. The analysis resulted in the inclusion of 108 patients per treatment group to achieve 80% or greater power to detect a difference of 20% in response rates between the two treatment groups. Our sample of 220 participants exceeds therefore the projected sample size needed to detect a clinical meaningful difference. A detailed explanation of the sample size calculation can be found under Section 11.2.
**Study Duration:**	The study duration is projected to be about four years months for patient recruitment and assessment and another year to finish up all the analysis and generate the final study report. We expect that all assessments with patients will be completed by March 2022.
**Study Schedule:**	First participant in: 01.03.2017<br>Last participant in: 31.05.2021<br>Last participant out: 31.03.2022<br>Database closure: 31.04.2022<br>Final analysis: 01.09.2022<br>Study report: 31.12.2022

**Investigator(s):**	**Gregor Berger**<br>Department of Child and Adolescent Psychiatry<br>University Hospital of Psychiatry<br>University of Zurich<br>Neumünsterallee 3<br>Postfach 1482<br>8032 Zürich<br>Phone: +41 43 499 26 71<br>Mobile: +41 76 464 61 54<br>Email: gregor.berger@puk.zh.ch<br><br>**Susanne Walitza**<br>Professor and Medical Director<br>Department of Child and Adolescent Psychiatry<br>University Hospital of Psychiatry<br>University of Zurich<br>Neumünsterallee 9<br>8032 Zürich<br>Phone: +41 43 499 27 30<br>Email: susanne.walitza@puk.zh.ch<br><br>**Klaus Schmeck**<br>Research Director<br>Department of Child and Adolescent Psychiatry Research Department<br>Universitäre Psychiatrische Kliniken (UPK) Basel<br>Wilhelm Klein-Strasse 27<br>4002 Basel<br>Phone: +41 61 325 80 38<br>Email: klaus.schmeck@upkbs.ch<br><br>All other investigators will be documented in the trial master file.
**Study Centre(s):**	A total of five Cantons of the German part of Switzerland agreed to participate in the multi-centre Omega-3-pMDD trial. The five Cantons include two academic centres (Zürich ZH, Basel-Stadt BS) as well as the inpatient and outpatient services of Canton St. Gallen (SG), Thurgau (TG) and Basel-Land (BL) encompassing all public child- and adolescent psychiatric services in a catchment area of 2.7 Million habitants (ZH 1446.4M, BS 190.6M, SG 495.8M, TG 263.7, BL 283.2).

**Statistical Considerations:**	The continuous primary outcome measure is the CDRS-R total score. The dichotomous primary outcome measures are recovery, response and remission. Data analysis will be performed in the intention-to-treat sample using a (generalized) linear random coefficient regression model. We will use multiple imputations for missing data. Under the assumption of random intercepts and slopes for each patient, the overall and treatment group-specific rate of change for the two treatment groups for the primary outcomes will be examined. A comparison on treatment slopes (linear trends with time) will then be conducted. As antidepressant medication prescription is allowed during the trial but might be related to treatment group, the data will be reanalyzed using multiple imputation to mimic observations of depression scores without antidepressant treatment.
**GCP Statement:**	This study will be conducted in compliance with the protocol, the current version of the Declaration of Helsinki, the ICH-GCP as well as all national legal and regulatory requirements.

ABBREVIATIONS

5-HIAA	5-Hydroxindoleacetc Acid
5-HT2	5-Hydroxytryptamine
AA	Arachidonic Acid
ADHD	Attention Deficit Hyperactivity Disorder
AE	Adverse Event
ALA	Alpha-Linolenic Acid
ALAT	Alanine Transaminase
ANT	Amsterdam Neuropsychological Tasks
aMDD	Adult Major Depressive Disorder
APA	American Psychological Association
ASAT	Aspartate Transaminase
ASEC	Antidepressant Side Effect Checklist
ASR	Annual Safety Report
AWMF	Arbeitsgemeinschaft der Wissenschaftlichen Medizinischen Fachgesellschaften
BAI	Beck Anxiety Inventory
BDI	Beck Depression Inventory
BHS	Beck Hopelessness Scale
BL	Basel -Land
BPD	Borderline Personality Disorder
BRIEF	Behaviour Rating Inventory of Executive Function
BS	Basel-Stadt
CA	Competent Authority (e.g. Swissmedic)
CAM	Complementary Alternative Medicine
CBCL	Child Behaviour Checklist
CBT	Cognitive behavioural therapy
CDI-2	Children’s Depression Inventory
CD-RISC	Connor-Davidson Resilience Scale

CDRS-R	Children's Depression Rating Scale-revised
CEC	Competent Ethics Committee
CGAS	Children's Global Assessment of Functioning
CGI	Clinical Global Impression rating scale
CGI-I	Clinical Global Impression rating scale-improvement
ClinO	Clinical Trials Ordinance
CRF	Case Report Form
CRH	Corticotropin-releasing Hormone
CSF	Cerebrospinal Fluid
CTC	Clinical Trials Centre
CTQ-SV	Childhood Trauma Questionnaire-Short Version
D2	Dopaminreceptor 2
DHA	Docosahexaenoic acid
DNA	Desoxyribonuclein acid
DM	Data Management
DSM-IV	Diagnostic and Statistical Manual of Mental Disorders-4th edition
EBPI	Epidemiology, Biostatistics and Prevention Institute
ECNP	European College of Neuropsychopharmacology
eCRF	Electronic Case Report Form
EFAs	Essential fatty acids
EPA	Eicosapentaenoic acid
EPS	Extrapyramidal-motorische Störung
ES	Effect Size
FDA	Food and Drug Administration (USA)
FFQ	Food Frequency Questionnaire
fMRI	Functional Magnetic Resonance Imaging
GC	Gas Chromatography
GCP	Good Clinical Practice
HETE	Hydroxyeicosatetraenoic Acid
HFG	Humanforschungsgesetz
HFV	Verordnung über die Humanforschung mit Ausnahme der klinischen Versuche
HoNOSCA	Health of the Nation Outcome Scales for Children and Adolescents
HPA	Hypothalamic-pituitary-adrenal

hsCRP	High-sensitivity C-reactive Protein
ICD-10	International Classification of Diseases- 10th edition
ICH	International Council on Harmonization
ICH-GCP	International Conference of Harmonisation- Good Clinical Practice
IDMC	Independent Data Monitoring Committee
IEPA	Early Intervention in Mental Health
IES-27-J&E	Jugend- & Elternfragebogen über impulsives Verhalten und Erleben
IFN	Interferon
IICT	Investigator Initiated Clinical Trials
IL	Interleukin
IMP	Investigational Medicinal Product
IQ	Intelligence Quotient
ITT	Intention to Treat
KAZ	Kantonsapotheke Zurich
KJPP	Klinik für Kinder- und Jugendpsychiatrie und Psychotherapie Zürich
KlinV	Verordnung über klinische Versuche in der Humanforschung
K-SADS-PL	Kiddie-Schedule for Affective Disorders and Schizophrenia-Present & Lifetime
LA	Linoleic acid
LC-MS	Liquid chromatography-mass spectrometry
LC-PUFAs	Long-chain polyunsaturated fatty acids
MARS	Medication Adherence Rating Scale
MARS-D	Medication Adherence Reporting Scale-Deutsch
MAQ	Medication Adherence Questionnaire
MCP-1	Monocyte Chemoattractant Protein-1
MCT	Medium Chain Triglycerides
MDD	Major Depressive Disorder
MedDRA	Medical Dictionary for Regulatory Activities
MS	Mass Spectrometry
n-3 FFQ	Omega-3 Food Frequency Questionnaire
OCD	Obsessive Compulsive Disorder

PI	Principal Investigator
pMDD	paediatric Major Depressive Disorder
PMS	Premenstrual Syndrome
PRN	Pro Re Nata (as needed)
PUFAs	Polyunsaturated Fatty Acids
RA	Responsible Applicant
Rbc	Red blood cell membranes
RCTs	Randomized Controlled Trials
RIAS	Reynolds Intellectual Assessment Scales
RR	Relative Risk
RWFT	Regensburger Wortflüssigkeits-Test
SAE	Serious Adverse Event
SD	Standard Deviation
SDQ	Strength and Difficulty Questionnaire
SG	St. Gallen
SIQ-Jr	Suicide Ideation Questionnaire-Junior
SMD	Standard Mean Difference
SNSF	Swiss national science foundation
SOP	Standard Operating Procedure
SSRI	Selective Serotonin Reuptake Inhibitor
SUSAR	Suspected Unexpected Serious Adverse Reaction
TADS	Treatment for Adolescents with Depression Study
TARMED	Tarif Médical
TG	Thurgau
TNF	Tumor Necrosis Factor
TSH	Thyroid Stimulating Hormone
UHR	Ultra High Risk
UK	United Kingdom
USZ	Universitätsspital Zürich

VMLT	Verbaler Lern- und Merkfähigkeitstest
WH	Working Hypothesis
WHO	World Health Organization
WI	Working Instruction
WISC IV	Wechsler Intelligence Scale for Children Version IV
WM	White Matter
ZH	Zürich

STUDY SCHEDULE

Study visit			-1		1	2	3	4	5	
	Time (min)	Examiner	Screening/ And Lead in Phase		Baseline	Acute phase		Main-tenance phase		Drop-out
Weeks			7 to 14 days prior baseline		0	6 +/-3d	12 +/-3d	24 +/-5d	36 +/-5d	
Study information	15	RC	x							
Informed consent	5	RSP	x							
Inclusion / exclusion	5	R	x		x					
Sociodemographic data	1	R	x							
Medical incl. psychiatric history	1	R	x							
Family history	1	RSP	x							
Physical Health (incl. Tanner)	1	R	x						x	
Current medication use	1	R	x		x	x	x	x	x	x
Omega-3 Food questionnaire	5	S	x				x		x	x
WHO Assist 3.0 Drug Use	10	R	x				x		x	x
Childhood Trauma & Olweus victimization scale	5	S	x							
Intervention			Pla (7-10 d)		Active/Placebo					
**Main outcome variables**										
Children's Depression Rating Scale CDRS-R	15	R	x		x	x	x	x	x	x
Diagnostic interview (K-SADS-PL)	60	R	x		x*	x*	x*	x*	x	x
**Clinician rated variables (at each clinical visit) +/- 2 weeks**										
Clinical Global impression Scale CGI-S/I	1	C	x			x	x	x	x	x
Children Global Assessment Scale CGAS	1	C	x			x	x	x	x	x
Attrition check list inkl. HoNOSCA	8	C	x			x	x	x	x	x
**Self-report variables (self rating for 13-<18yr, parent rating for 8-≤13yr)**										
Children's Depression Inventory DIKJ	15	S			x	x	x	x	x	x
Beck Hopelessness Scale II BHS	5	S			x	x	x	x	x	x
Beck Anxiety inventory BAI II	5	S			x	x	x	x	x	x
Suicidal ideation questionnaire SIQ-Jr	5	S			x	x	x	x	x	x
The Insomnia Severity Index	1	S			x	x	x	x	x	x
Perceived Stress Scale PSS-10	5	S			x		x		x	x
Connor-Davidson Resilience Scale	10	S			x	x	x	x	x	x
KIDscreen-CAT-27	15	SP	x(P)		x		x		x	x
SDQ	5	SP	x(P)		x		x		x	x
IES-27-J	15	SP	x(P)		x		x		x	x
ASEC (AD side effect self report scale)	5	S			x	x	x	x	x	x
**Cognitive outcome variables**										
BRIEF (executive Functioning questionnaire)	10	SP	x(S,P)				x		x	
Cognitive battery	50	R	x				x		x	
RIAS (short IQ test for matching)	20	R				x				x
**Biological outcome variables**										
Clinical bloods, hsCRP (2-3d prior randomization)<br>Red blood cells (PUFAs), Serum (e.g. immune multiplex marker chip), Buffy coat for tissue repository (e.g. genetic, epigenetic markers)	5	N<br>N	x<br>x				 x		x<br>x	x<br>x
Urine (drug screen, F2 isoprostane)	5	N	x				x		x	x
Saliva cortisol	1	N	x				x		x	x
Hair	1	R				x			x	
**Other measures**										
Drug dispensing/ Pill count/appointment	1	R	x		x	x	x	x		
AE					x	x	x	x	x	x
MARS-D	3	S			x	x	x	x	x	
Approx. time requirement for patient / family only (min)			180	90	90	90	210	120	210	200
	* Only subscale 'depressive disorders' will be performed									
	R = researcher; C = clinician; S = self-rated; P = parent-rated; N = study nurse									

1 STUDY ADMINISTRATIVE STRUCTURE

This multicentre trial is centrally coordinated by the Department of Child and Adolescent Psychiatry of the University of Zurich and conducted in five Cantons of the German part of Switzerland (ZH, BS, BL, TG, SG). Below are the personnel involved in the study at this stage. A list of co-investigators and other study team members such as doctoral students and study nurses involved in the clinical trial will be given in the study team contact summery form in the Study Master File and at each Investigator Site File.

1.1 Sponsor, Sponsor-Investigator

Gregor Berger
Department of Child and Adolescent Psychiatry
University Hospital of Psychiatry
University of Zurich
Neumünsterallee 3
Postfach 1482
8032 Zürich
Phone: +41 43 499 26 71
Mobile: +41 76 464 61 54
Email: gregor.berger@puk.zh.ch

Main applicant and PI, overall governance of the project, responsible for the conceptualisation, trial design, overall management and implementation of the Omega-3-pMDD Study in the main and partner centres, development of the research protocol, case report forms (CRFs), standard operating procedures (SOPs), training and supervision of research staff, recruitment process, management of serious adverse events (SAE), data management (DM) and analysis, contact and coordination with laboratories and external agencies (incl. industrial partners, Clinical Trials Centre (CTC), clinical trials pharmacy and Independent Data Monitoring Commitee (IDMC), local and national ethics committees, as well as the Antistress AG Burgerstein who provides the study medication incl. placebo, dissemination of trial results (organization of conference attendances, publication strategy, media releases).

1.2 Principal Investigator(s)

Zurich
Susanne Walitza

Professor and Medical Director
Department of Child and Adolescent Psychiatry
University Hospital of Psychiatry
University of Zurich
Neumünsterallee 9
8032 Zürich
Phone: +41 43 499 27 30
Email: susanne.walitza@puk.zh.ch

Clinical responsibility for the management / realization of the trial at Zurich. Overall governance of project, conceptualisation, realisation, data analysis, writing, publication and promotion of the project, support in networking, academic mentorship Dr. G. Berger.

Basel-Stadt

Klaus Schmeck
Research Director
Department of Child and Adolescent Psychiatry Research Department
Universitäre Psychiatrische Kliniken (UPK) Basel
Wilhelm Klein-Strasse 27
4002 Basel
Phone: +41 61 325 80 38
Email: klaus.schmeck@upkbs.ch

Governance of Basel site, concepualisation, realisation, data analzsis, writing, publication and promotion of the project.

Alain Di Gallo
Department of Child and Adolescent Psychiatry
Universitäre Psychiatrische Kliniken (UPK)
Schaffhauserrheinweg 55
4058 Basel
Phone: +41 61 685 21 21
Email: alain.digallo@upkbs.ch

Basel-Land

Brigitte Contin-Waldvogel
Psychiatrie Baselland
Bienentalstrasse 7
4410 Liestal
Phone: +41 61 553 59 01
Email: brigitte.contin@pbl.ch

Thurgau

Lars Wöckel
Clienia Littenheid
Privatklinik für Psychiatrie und Psychotherapie

9573 Littenheid
Phone: +41 71 929 60 60
Email: Lars.Woeckel@clienia.ch

Bruno Rhiner
Child and Adolescent Psychiatric Services Thurgau
Schützenstrasse 15
8570 Weinfelden
Phone: +41 71 686 47 01
Email : bruno.rhiner@stgag.ch

St. Gallen
Suzanne Erb
Child and Adolescent Psychiatric Services St. Gallen
Brühlgasse 35/37
Postfach 447
9004 St. Gallen
Phone: +41 71 243 45 45
Email: Suzanne.Erb@kjpd-sg.ch

St. Gallen
Michael Schmid
Child and Adolescent Psychiatric Services St. Gallen
Brühlgasse 35/37
Postfach 447
9004 St. Gallen
Phone: +41 71 243 45 45
Email: Michael.Schmid@kjpd-sg.ch

St. Gallen
Ulrich Müller-Knapp
Klinik Sonnenhof
Sonnenhofstrasse 15
9608 Ganterschwil
Phone:+41 71 983 26 33
Email: Ulrich.Mueller-Knapp@kjpz.ch

1.3 Neuropsychology

Renate Drechsler
Head of Neuropsychology laboratory
University Clinic of Child and Adolescent Psychiatry
University of Zurich

Eisengasse 16
8008 Zürich
Phone: + 41 43 556 40 50
Email: renate.drechsler@kjpd.uzh.ch

Investigation of omega-3 fatty acids effects on cognition. PhD Supervisor.

1.4 Coordinating Investigator

Gregor Berger
Department of Child and Adolescent Psychiatry
University of Zurich
Neumünsterallee 3
Postfach 1482
8032 Zürich
Phone: +41 43 499 26 71
Mobile: +41 76 464 61 54
Email: gregor.berger@puk.zh.ch

1.5 Statistician ("Biostatistician")

Ulrike Held
Epidemiology, Biostaticis and Prevention Institute (EBPI) Department of Biostatistics
University of Zurich
Hirschengraben 84
8001 Zürich
Phone : +41 (0)44 634 4539
Email : Ulrike.held@uzh.ch

Alexander Roth
Biostatistician KJPP
Department of Child and Adolescent Psychiatry
University of Zurich
Neumünsterallee 9
8032 Zürich
Phone : +41 (0)43 499 27 67
Email : alexander.roth@kjpd.uzh.ch

1.6 Laboratory (if applicable)

Edna Grünblatt
Head of Neurobiochemistry laboratory
University Clinic of Child and Adolescent Psychiatry
University Zurich

Wagistrasse 12 (5th Floor Room K118)
8952 Schlieren
Phone: +41 (0)43 556 40 39
Email: edna.gruenblatt@kjpd.uzh.ch

Inflammatory mediators / Blood processing / Tissue repository

Martin Hersberger
Head of the Division of Clinical Chemistry and Biochemistry University Children's Hospital Zurich
Steinwiesstrasse 75
8032 Zürich
Phone: +41 (0)44 266 75 41
Email: martin.hersberger@kispi.uzh.ch
Bioactive Lipids

Clemens von Schacky
CEO
Omegametrix GmbH
Am Klopferspitz 19
82152 Martinsried
Germany
Phone:+49 89 559 63 007
Email: c.vonschacky@omegametrix.eu
PUFAs

Anne Eckert

Department of Neurobiology
Universitäre Psychiatrische Kliniken Basel
Wilhelm Klein-Strasse 27
4002 Basel
Phone: +41 61 325 5487
Email: anne.eckert@upkbs.ch

1.7 Monitoring Institution

Clinical Trials Centre (CTC)
University of Zurich
Rämistrasse 100
8091 Zürich
Phone : +41 (0)44 634 55 09
Email : ctc-zkf@usz.ch

Monitoring and data management

1.8 Independent Data Monitoring Committee

The safety and integrity of the study will be judged on a regular basis by an independent committee of experts (Independent Data Monitoring Committee; IDMC), at a frequency of at least once a year. Members of the IDMC will include an expert in child and adolescent psychiatry (Prof. Dr. med. Romuald Brunner, Vice Director of the Centre for Child and Adolescent Psychiatry, Heidelberg University Hospital), an expert in biostatistics (Dr. Julia Braun, Scientific Staff of the Epidemiology, Biostatistics and Prevention Institute of the University of Zurich) and an expert in pharmacology and ethics (Prof. Dr. Med. Jürgen Drewe, Director of Preclinical Research of Max Zeller Soehne AG).
The members of this board will have access to all SAEs and SUSARs and to the inclusion and drop-out rates. Key task will be to monitor safety and harm data, in particular to monitor the number of suicide attempts. SAE will be reported to the ethics committee and the IDMC in writing. If the IDMC deems it necessary additional information can be provided. If the IDMC deems it necessary to perform an interim analysis of the safety or harms data, the IDMC can directly liaise with the CTC and clinical trials pharmacist to do so. In case that the IDMC deems it unethical to continue the trial because of a premature (prior to achieving the n=220) difference between active and placebo (safety data), the trial can be terminated at their request.
The IDMC is also authorised to suggest changes to the protocol or to provide an altered judgement of feasibility when information from the annual safety report or new information about the applied study medication has become available. If deemed necessary for their evaluation, the IDMC is allowed to review unblinded data. The central study team members will not be exposed to these data analysis, unless the study is terminated prematurely.

1.9 Any other relevant Committee, Person, Organisation, Institution

Clinical Trials Pharmacy

Kantonsapotheke Zürich

Spöndlistrasse 9
8006 Zürich
Phone : +41 44 255 45 46
Email : info@kaz.zh.ch
Blinding, packaging and labelling of medication

Burgerstein Vitamine
Antistress AG
Fluhstrasse 30
8640 Rapperswil-Jona
Switzerland
Phone: +41 55 220 12 12
www.burgerstein.ch

Production of IMP and Placebo

2 ETHICAL AND REGULATORY ASPECTS

Before this study will be conducted, the protocol, the proposed participant information and consent form as well as other study-specific documents will be submitted to a properly constituted Competent Ethics Committee (CEC) and Competent Authorities (Swissmedic) in agreement with local legal requirements, for formal approval. Any amendment to the protocol must as well be approved.

The decision of the CEC and Swissmedic concerning the conduct of the study will be made in writing to the Sponsor-Investigator before commencement of this study. The clinical study can only begin once approval from all required authorities has been received. Any additional requirements imposed by the authorities shall be implemented.

2.1 Study Registration

The study will be registered in both the Swiss Federal Complementary Database ("Portal") and the Clinicaltrials.gov registry database, before participant recruitment in the study starts.

2.2 Categorisation of Study

The study is considered to fall under category C according to ClinO Art. 19.

2.3 Competent Ethics Committee (CEC)

Approval from the appropriate constituted Competent Ethics Committee is sought for each study site in the clinical trial (zuständige kantonale Ethikkommissionen) and the national competent authority (SwissMedic). The reporting duties and allowed time frame are respected. No substantial changes are made to the protocol without prior Sponsor, CEC, CA approval, except where necessary to eliminate apparent immediate hazards to study participants. Premature study end or interruption of the study is reported within 15 days. The regular end of the study is reported to the CEC within 90 days, the final study report shall be submitted within one year after study end. Amendments are reported according to chapter 2.10.

2.4 Competent authority (CA)

The Sponsor will obtain approval from Swissmedic before the start of the clinical trial. Reporting will be done within the allowed time frame. Planned or premature study end are reported within 90 and 15 days, respectively. The final report will be submitted to the CA within one year after the end of the study. Amendments are reported according to chapter 2.10.

2.5 Ethical Conduct of the Study

The study will be carried out in accordance with principles enunciated in the current version of the Declaration of Helsinki, the guidelines of Good Clinical Practice (GCP) issued by ICH, and Swiss competent authority's requirements.

CEC and competent authorities will receive annual safety and interim reports and be informed about study stop/ end in agreement with local requirements.

2.6 Declaration of Interest

We declare no conflict of interest.

2.7 Patient Information and Informed Consent

Participation in the study is preceded by counselling by the treating doctor/ psychologist or investigator in accordance with ICH-GCP guidelines, during which the patients and their guardians must be informed about the nature and entire course of the study, potential individual benefits and personal risk shall be explained. Here it will be re-emphasised that participation is absolutely voluntary. Given that this study is conducted in a psychiatric setting, the study information may also be given by a research psychologist. In this case, the research psychologist will sign the informed consent additionally to the psychiatrist. Potential participants and their parents/ guardians are given sufficient time to read all the provided information (approved by the appropriate authorities) and clarify any questions with the treating doctor/ psychologist or investigator. Participation only becomes possible after the patients, their guardians as well as the investigator/doctor have signed the informed consent document. This consent can be revoked at any time without citing reasons and without any consequences. No examinations or other activities will take place before the informed consent procedure is completed. A copy of the consent form and the patient information sheet will be given to the participants and their guardians.
In case the patient information sheet or the consent form change, participants will be informed immediately and relevant information will be passed on to the ethic committees for approval. New patient information and consent will be discussed in detail again, the participant will again be asked for written consent, and a copy of the documents will be given to the subject.

Given participants are between 8 and 17 years old at the start of the study, informed consent are provided by their legal representatives/parents, in line with the Clinical Trial Directive, the Declaration of Helsinki and ICH-GCP. Their consent must represent the minor's presumed will and may be revoked at any time, without detriment to the minor. Whenever appropriate, the minor should participate in the informed consent process together with the parents. The researcher will also obtain the minor's assent in addition to the consent of the legal representatives/parents. The minor's assent is not sufficient to allow participation in the study; informed consent of the legal representatives/parents is required. Consent from legal representatives/parents and assent from the minor should be sought at the same time. In any case, the minor will receive information

according to its capacity of understanding, from staff with experience in minors, regarding the study, its risks and benefits. The explicit wish of a minor who is capable of forming an opinion and assessing this information to refuse participation will be followed; in such case, the minor can be withdrawn from the study at any time. If a minor subject does not want to participate, they will not be included in the study. This is also explicitly stated in the patient information sheet.
In case a minor reaches adulthood (age of 18) during the study, it will not be necessary to obtain novel informed consent as patients have already signed thier own informed consent form at study inclusion (in addition to the consent form signed by the legal representatives, usually the parents).

2.8 Participant Privacy and Confidentiality

The investigator affirms and upholds the principle of the participant's right to privacy and that they shall comply with applicable privacy laws. Especially, anonymity of the participants shall be guaranteed when presenting the data at scientific meetings or publishing them in scientific journals.

Individual subject medical information obtained as a result of this study is considered confidential and disclosure to third parties is prohibited. Subject confidentiality will be further ensured by utilising subject identification code numbers to correspond to treatment data in the computer files.

Such medical information may be given to the participant’s personal physician or to other appropriate medical personnel responsible for the participant’s welfare, if the patient has given his/her written consent to do so.

For data verification purposes, authorised representatives of the Sponsor (-Investigator), a competent authority (e.g. Swissmedic), or an ethics committee may require direct access to parts of the medical records relevant to the study, including participants’ medical history.

Regarding data privacy, subjects will be informed about pseudonymous recording. The allocation of a unique study number that can be traced back to the patient if necessary will be used. The list with patients’ names and contact details are kept with the principal investigators. Outside laboratories will only be provided with coded documentation not enabling them to identify the individual patient. The sharing of data will be done in accordance with ICH-GCP guidelines. In case subjects cannot agree to this central collection of data they cannot participate in the study.

2.9 Early Termination of the Study

The Sponsor-Investigator may terminate the study prematurely according to the following circumstances:

- ethical concerns,
- insufficient participant recruitment,
- when the safety of the participants is doubtful or at risk, respectively,
- alterations in accepted clinical practice that make the continuation of a clinical trial unwise,
- early evidence of benefit or harm of the experimental intervention
- recommendations of the Independent Data Monitoring Committee

- the sponsors general expertise regarding the significance of the accumulating data or
- other important reasons such as accumulating external evidence or quality concerns.

2.10 Protocol Amendments

In order to maintain comparable conditions in all study sites and to obtain an unobjectionable data analysis changes of the protocol are not intended. If nonetheless changes become necessary they are reported as amendment and submitted to the responsible ethics committees (*die zuständigen kantonale Ethikkommissionen*) and, if necessary, to the national authorities (SwissMedic) according to the regulations, taking into account the distinction between substantial and non-substantial amendments.

Substantial amendments (significant changes) are only implemented after approval of the CEC and CA respectively.

Significant changes to be authorised by the CEC are the following:

- changes affecting the participants' safety and health, or their rights and obligations;
- changes to the protocol, and in particular changes based on new scientific knowledge which concern the trial design, the method of investigation, the endpoints or the form of statistical analysis;
- a change of trial site, or conducting the clinical trial at an additional site; or
- a change of sponsor, coordinating investigator or investigator responsible at a trial site.

Significant changes to be authorised by Swissmedic are the following:

- changes to the therapeutic product, or to its administration or use;
- changes based on new preclinical or clinical data which may affect product safety; or
- changes concerning the production of the therapeutic product which may affect product safety.

Under emergency circumstances, deviations from the protocol to protect the rights, safety and well-being of human participants may proceed without prior approval of the sponsor and the CEC/CA. Such deviations shall be documented and reported to the sponsor and the CEC/CA as soon as possible.
All Non-substantial amendments are communicated to the CA as soon as possible if applicable and to the CEC within the Annual Safety Report (ASR).

The sponsor is responsible for all protocol amendments and for communicating potential protocol modifications to all relevant parties. All principal investigators are allowed to make suggestions for protocol amendments to the sponsor, who will decide whether the proposed amendments are necessary. In case of an amendment, all study centres and other relevant parties will be informed by the sponsor by email, and the amendments will be submitted to the responsible ethic committees. In addition, a private protected study website will be provided. All the relevant documents will also be available in the master trial file and at each individual site file.

3 INTRODUCTION

3.1 Background and Rationale

3.1.1. Adult and paediatric major depressive disorders

Adult Major Depressive Disorders (aMDDs) are characterized by the presence of at least five of the following symptoms for most of the time lasting for at least 2 weeks or more: sadness, emptiness or hopelessness (in children and adolescents sadness often manifests as irritable mood); loss of interest or pleasure in activities; psychomotor retardation or agitation (observed); decreased energy or fatigue; worthlessness or inappropriate guilt; thoughts of death and suicide; diminished concentration or indecisiveness; insomnia or hypersomnia; change in weight or appetite [1]. In childhood most presentations are first-onset presentations that have a slightly better prognosis than recurrent forms of MDD. About 60% of a clinical cohort meeting DSM criteria for paediatric major depressive disorder (pMDD) will recover within 9 months (in contrast to adjustment disorders with depressive features that have a faster and better recovery rate). The mean length of pMDD is about 9 months [2, 3]. Childhood- and adolescent-onset depression have similar clinical presentations except of the melancholic symptoms that are more prevalent in adolescence [4]. The natural illness course warrants a long study duration to address the efficacy and effectiveness of a proposed treatment.
In a recent school survey about one in ten adolescents in Zürich reported moderate to marked depressive symptoms, and one in three if you include mild depressive symptoms [5], which corresponds quite well to national and international surveys [6]. Upon use of structured clinical interviews in <18year olds 1-6% meet criteria for a MDD [7]. Depressive disorders are often emerging in adolescence, are often not recognized even by professionals [8] and only about a quarter receive appropriate treatment [9]. These findings are alarming given that between 10 and 24 year olds MDD is the leading cause of disability [10]. An early onset of the disease is a risk factor for chronic and recurrent forms of depression in adulthood with more than half experiencing a first relapse within five years [11]. MDD is associated with difficulties in relationships, impaired school and work functioning, and an increased risk of substance abuse [12, 13]. MDD is a major contributor to the burden of suicide and poor long-term health later in life (in particular ischaemic heart disease) [14, 15].

3.1.2 MDD and suicidality

Three recent Cochrane reviews analysing psychological [16], pharmacological [17] and relapse preventive interventions [18] in pMDD illustrate how poor the currently available evidence-based knowledge is for treating this vulnerable patient population. In fact, there have been major concerns that the use of antidepressant medications in this age group may worsen suicidal behaviours [19]. Following a thorough and comprehensive review of all available published and unpublished RCTs of antidepressants in children and adolescents in October 2004, the U.S. Food and Drug Administration (FDA) issued a public warning about an increased risk of suicidal thoughts or behaviours in children and adolescents treated with SSRI medications. In 2006, the

FDA extended the warning to include young adults up to the age of 25. Other agencies across the world such as the Swissmedic, the UK Medicines and Health Care Products Regulatory Agency and the European Medicines Agency followed [20]. The debate that SSRIs may increase suicidal ideation has contributed to a lot of insecurity in affected individuals and their families, as well as in the professional community [21-23]. A consequence of the “black box warning” was that prescribing practices of antidepressants have decreased for young people [24]. Additionally a recent reanalysis of the SmithKline Beecham study 329, a placebo-controlled RCT with paroxetine in pMDD [25, 26] provides evidence that despite negative findings on all a priori primary outcomes, the study was “sold” as a positive study based on a post-hoc outcome that was introduced after trial initiation. As a consequence Paroxetine has been prescribed to tens of thousands of children and adolescents with pMDD without sound scientific evidence. In contrary, the safety data showed that the paroxetine group reported more suicidal ideations than the placebo group [27]. However, epidemiological data provides some evidence that in regions with the strongest decrease in antidepressant use showed an increase in suicide rates in children and adolescents [28]. More recent data in severe forms of depression provides some evidence that the regular intake of fluoxetine, sertraline and to some extent citalopram in severe forms of depression may be protective against suicide (probably by treating the underlying illness) despite of an increase in suicidal ideation [29]. This debate is certainly also of relevance for Switzerland, as the age-corrected mean suicide rate in this age group over the last 13 years is 7.6 per 100’000 teenagers and is above the worldwide average [30].

Based on the current partially conflicting evidence, the use of SSRIs in the treatment of pMDD should be evaluated very carefully and should mainly be considered in the more severe cases of pMDD. Nevertheless, in regions where child and adolescent therapists trained in cognitive behavioural or interpersonal therapy practices are not readily available, the use of antidepressants may often be the only choice [16]. However, patients and their parents are often very sceptical about the use of antidepressants, in particular since the intensive media attention about the increase in suicidal ideation has emerged [31]. In some cases this critical attitude probably results in a delay or premature discontinuation of necessary treatments of severe forms of pMDD potentially contributing to a poorer outcome, or even to suicides [32, 33].

Experts in the field propose a stepwise approach from more benign to more invasive treatments. In line with the German S3 Guidelines (AWMF-Nr. 028-043) [34] cognitive behavioural or interpersonal psychotherapy is considered a good first-line treatment and the golden standard in the treatment of pMDD. The timing at what point a combination of fluoxetine with CBT shall be introduced depends on the severity of depression and the availability of psychological treatment options. The decision to start an antidepressant should whenever possible be reserved for the more severe cases and be based upon a careful clinical expert decision. The latter decision should not be made easily as no single novel antidepressant is approved for pMDD in Switzerland (e.g. Swissmedic even does not recommend the use of Fluoxetine in <18 year olds despite the strongest evidence within its class; in Switzerland any SSRI use in pMDD is therefore “off label”). Nevertheless, in the real world about 50% of all SSRIs in children and adolescents are prescribed by general practitioners and paediatricians despite the fact that they themselves report that they have not been adequately trained to do so. Non-specialists often overestimate the benefit over the risk associated with SSRI use in minors [35, 36].

3.1.3 The importance of Omega-3 fatty acids

The precursors of omega-3 fatty acids (alpha-Linolenic acid ALA) and omega-6 fatty acids (Linoleic acid LA) are essential fatty acids (EFAs) meaning that humans cannot synthesise them de novo. Humans have to supply EFAs through diet similar to vitamins. Since the industrial revolution human lifestyle and diet underwent major changes, one of them being a dramatic shift from a balanced omega-6 to omega-3 fatty acids diet to an excess in omega-6 and trans fatty acids intake in Western societies [37]. Therefore, humans living in modern societies are exposed to a nutritional environment that does not match their genetic constitution resulting in the hypothesis that the rapid rise in civilization diseases such as diabetes, cardiovascular disorders, but also psychiatric disorders such as depression may beside other reasons also be the consequence to these dietary changes [38]. The source of the omega-3 LC-PUFAs is mainly oily fish, e.g. salmon or sardines, whereas the precursor ALA is also present in plant oils, such as canola oil, nut oils, or algae oils. A small proportion of the essential precursors ALA and LA can be transformed into bioactive long-chain polyunsaturated fatty acids (LC-PUFAs), such as the omega-6 arachidonic acid (AA) and its metabolites, and the omega-3 eicosapentaenoic acid (EPA) and docosahexaenoic acid (DHA) and their bioactive metabolites (see page 20 Fig.1). LC-PUFAs and their bioactive lipids have important physiological roles in normal brain development, but also other processes such as inflammation and immunity. A range of epidemiological and experimental studies provide some evidence for a link between cardiovascular disorders, metabolic syndrome or depressive disorders that are all proposed to be associated with an increase in proinflammatory cytokines and a deficiency in omega-3 fatty acids. The latter may have direct implications for prevention and treatment [38, 39].

3.1.4 Dietary intake of Omega-3 fatty acids and MDD

Epidemiological studies found an inverse association between the intake of oily fish (rich in omega-3 fatty acids) and the prevalence [40-45] and incidence [46-48] of depressive disorders, postpartum depression [49] and bipolar disorder [50]. A recent meta-analysis of 26 studies involving 150’278 participants found that fish intake may have a protective effect comparing the highest versus lowest fish consumption group with a pooled relative risk (RR) of depression of 0.83 [51]. Furthermore, inverse relationships between fish intake, membrane omega-3 fatty acids and number of suicide attempts have also been reported by independent groups [41, 52, 53].

3.1.5 Omega-3 fatty acids deficiency in MDD

LC-PUFAs can be measured in red blood cell membranes (rbc) or plasma. Due to the high fluctuation of free fatty acids, the measurement of rbc LC-PUFAs is a better proxy for the mean LC-PUFA intake over the last couple of weeks. A meta-analysis including a total of 3318 patients with MDD [51, 54] confirms a significant decrease in total omega-3 fatty acids (ES=-0.51, $p<0.0001$), in particular in EPA (ES=-0.18, $p=0.004$) and DHA (ES=-0.35, $p=0.0002$) [49]. Similar deficits are also present in postpartum depression [55] and social anxiety disorder [56]. High n-6/n-3 omega fatty acid ratios are frequently reported [57-60]. Similar changes could also be observed in pMDD [61].

3.1.6 Inflammatory mediators, Omega-3 fatty acids and MDD

Proinflammatory cytokines in both plasma and CSF have been found to influence the progression and severity of depressive disorders in different populations. Studies have shown elevated serum levels of Il-1, Il-6, TNF-alpha und MCP-1 in depressed patients, but have presented mixed results with Il-8 serum levels and with Il-6 and MCP-1 CSF levels [62]. A recent omega-3 fatty acid RCT in aMDD found that the ratio between pro- and antiinflammatory markers (hsCRP, IL-6, IL-1ra, Leptin, Adiponectin) is a strong predictor of omega-3 fatty acids response [63].

3.2 Investigational Product and Indication

In the proposed study we decided to use for the active treatment a daily dose 500mg EPA/ 250mg DHA in our 8 to <13 year old, and 1000mg EPA / 500mg DHA for the 13 to <18 years old. Omega-3 fatty acids are used worldwide as nutritional supplements with important physical and psychosocial health benefits. Pre-clinical data demonstrate very low level of oral toxicity of omega-3 fatty acids. Please note that omega-3 fatty acids have been proven safe at much larger doses of up to 3 g/day in a total of more than 100'000 cardiovascular patients [64, 65].
Active and placebo will contain mixed tocopherol (vitamin E) to prevent oxidation and the capsule shell will be made out of gelatin (from fish) and glycerol. The main filling ingredient in the placebo capsules will be medium chain triglycerides, because they do not contain any insaturated fatty acids and are therefore very stable and in these small quantities they do not have any pharmacological effect. Placebo will also contain a very small amount of fish oil to mimic the fishy flavour and taste and prevent unblinding. More information can be found in the Investigator's Brochure.
Burgerstein will produce specially manufactured child capsules as study medication that can easily be swallowed by children.

3.3 Preclinical Evidence

The underlying mechanisms of the potential preventive and therapeutic actions of omega-3 fatty acids against depression are still unclear. Preclinical and clinical data point towards several mechanisms that most likely act in concert [66]. Some of them might be more responsible for the postulated short-term, other for the more long-term effects of omega-3 fatty acids:

- **Omega-3 fatty acids, stress and the HPA-axis**: Major depression is linked to a range of mediators of the stress response system (e.g. the subgenual prefrontal cortex, the hippocampus and the amygdala) that is mainly modulated by the noradrenergic system and the CRH/HPA axis resulting in a markedly exaggerated, persistent elevation of the stress response [67]. Omega-3 fatty acids have shown to attenuate stress-related changes in animal models with depressive features [68-71] and humans [72-74].
- **Omega-3 fatty acids and brain development**: An association between depression and abnormal brain development has been suggested [75]. Myelination and synaptic pruning are core processes during normal pubertal brain

development. The regulation of PUFA metabolism is crucial for both processes [76, 77]. Of particular interest is a preclinical study investigating cognition and behaviour across different developmental stages. Omega-3 fatty acids deficient diets across consecutive generations produced a modality-selective and task-dependent impairment in cognitive and motivated behaviour in adolescence distinct from the deficits observed in adults [78, 79]. Omega-3 fatty acids attenuate such depression-like animal behaviours during critical periods of brain development [80]. Furthermore, the FADS haplotype determining LC-PUFAs availability and concentrations in white matter (WM) showed age-related WM differences (significant age x genotype interactions, p(corrected) < 0.05) in humans. PUFA metabolism is therefore likely to play a role in disorders of neurodevelopmental origin [81].

- **Omega-3 fatty acids, neuroplasticity, neurotrophic and protective hippocampal effects**: Hippocampal changes in depression are well documented [82]. Animal models with structural hippocampal alterations with depression-like and anxiety-like behaviours [83, 84] provide evidence that omega-3 fatty acids have a preventive and neurotrophic effect against such changes [85, 86]. Our own group could demonstrate that omega-3 fatty acids enhance hippocampal cell viability and are able to protect hippocampal cells from stress-related damage [87].
- **Omega-3 fatty acids and monoaminergic neurotransmission**: Monoaminergic transmitter systems are proposed to be involved in the pathogenesis of depression. Animal experiments of omega-3 fatty acids deprived rats provide evidence for an increase in serotonin 2 (5-HT2) and a decrease in dopamine 2 (D2) receptor density in the frontal cortex, as well as an increased serotonin turnover in the prefrontal cortex and decreased midbrain tryptophan hydroxylase-2 expression [88-94]. In humans, omege-3 intake is associated with an increase in cerebrospinal fluid 5-HIAA release [95, 96].
- **Omega-3 fatty acids, immune-modulation, anti-inflammation and anti-oxidation**: Several lines of evidence support an altered immune-modulation in the pathophysiology of depression [97-99]. Omega-3 fatty acids have immune-modulatory, anti-inflammatory and pro-resolving properties [100], e.g. via the down-regulation of pro-inflammatory omega-6, the promotion of proresolvins, neuroprotectins and anti-inflammatory mediators [101-103]. Omega-3 fatty acids seem to induce protective in vivo brain mechanisms against oxidative stress. The main applicant could demonstrate that Ethyl-EPA supplementation is associated with a marked increase of glutathione, a strong intracellular antioxidant using proton magnetic resonance spectroscopy in patients with a first-episode psychosis [104]. Another group found similar effects in older patients at risk for depression [105]. Some evidence regarding the measurement of glutathione in peripheral blood is also suggestive that omega-3 fatty acids may support the antioxidative defence system in individuals at ultra-high risk for psychosis [106].
- **Omega-3 fatty acids, membrane structure and function**: A decrease in membrane fluidity can affect the rotation and diffusion of proteins and other bio-molecules within the membrane, thereby affecting the functions of these molecules and processes. An increase in membrane fluidity results in a more flexible membrane and facilitates transmission (e.g. in the retina) [107]. In vivo imaging techniques such as diffusion tensor imaging could demonstrate that omega-3 fatty

acids are closely linked to PUFA metabolism [81]. The effect of omega-3 fatty acids on membrane structure [108] may contribute to its clinical effects, in particular in augmentation studies. The principal investigator was involved in a study that could demonstrate that T2-relaxation time normalizes under the influence omega-3 fatty acids potentially being a signifier of normalization in membrane structure [109].

3.4 Clinical Evidence to Date

3.4.1 Omega-3 fatty acids RCTS in MDD and related disorders

Several clinical research groups investigated the use of omega-3 fatty acids in controlled treatment trials in a range of conditions that also assessed depressive symptoms:

- Primary diagnosis of adult major depressive disorders (MDD) [63, 110-123]
- Depressive episodes in bipolar affective disorders [124-130]
- Depression during or post pregnancy (postpartum depression) [131-133]
- Depression in non-MDD mood disorders (e.g. PMS, dysthymia) [134-139]
- Depression in other psychiatric conditions (e.g. borderline personality, self-harm, OCD) [140-144]
- Depression in established schizophrenia [110, 145]
- Depression in Alzheimer's dementia/mild cognitive impairment [121, 146, 147]
- Depression in Parkinson disease [148]
- Depression in medical conditions (cerebro-vascular and metabolic diseases or cancer) [149-153]
- Depressive symptoms in healthy individuals [154-159]

Several critical meta-analytic reviews have tried to integrate the above-mentioned very heterogeneous group of controlled treatment trials investigating the effects of omega-3 fatty acids on mood symptoms [160-166]. Most meta-analysis confirmed a statistical significant effect in favour of omega-3 fatty acids with minimal to moderate effect sizes depending on the selection of studies (except of one meta-analysis [164]). Effect sizes in favour of omega-3 fatty acids [51] are larger if RCTs are selected based on 1.) a EPA/DHA ratio >60% of the overall omega-3 fatty acids [161, 163] and 2.) only RCTs with a primary diagnosis of MDD are included [161, 166]. To our knowledge, only one pilot RCT (n=20) in children with a mean age of 10 was performed [167].

3.4.2 Omega-3 fatty acids RCTs in MDD

Martins et al [161] meta-analysis including RCTs with primary and secondary MDD found a significant overall SMD = -0.291 but with study heterogeneity and evidence for publication bias. A more recent meta-analysis by Sublette et al [163] only including primary MDD RCTs dichotomized according to a EPA-content >60% of the overall omega-3 fatty acids content found a moderate effect size (SMD=0.558) with negligible contribution of random effects or heteroscedasticity. Bloch and Hannestadl's meta-analysis [164] including studies with mild depression could not replicate previous meta-analyses, however a sub-analysis restricted to moderate to marked depression confirmed a SMD of 0.42. It is likely that Rogers et al [134] study investigating the effects of omega-3 fatty acids in mild depression in a large non-clinical population was responsible for the negative overall outcome as the study accounted for 31.7% of the overall

weight [168]. Grosso et al [166] found a SMD=0.56 for primary MDD, an SMD=0.22 for non-primary MDD, and an overall SMD=0.38 in favour of omega-3 fatty acids treatment compared to placebo. The above mentioned meta-analyses confirmed that the use of EPA rather than DHA rich formulations is responsible for the clinical efficacy of omega-3 fatty acids. Two RCTs encompassing a large proportion of patients with refractory depression highlight the potential use of EPA-enriched omega-3 fatty acids as an augmentation treatment (potentially via an increase in membrane fluidity) [110, 111]. Two RCTs in populations with other than a primary MDD provide evidence of an association between inflammation and omega-3 fatty acids response: 1.) A placebo-controlled trial investigating the positive effects of omega-3 fatty acids on depressive symptoms and chronic inflammation in haemodialysis patients [169], and 2.) a study [170] that found a preventive effect of EPA against the development of depressive symptoms in IFN-alpha-treated hepatitis C virus carriers (associated with a very high risk of drug-induced depressive symptoms). The latter two studies suggest that omega-3 fatty acids rich in EPA may modulate its antidepressant properties via immune-modulatory strategies (see below, mechanism of action), which is of interest in the light of more recent models of the underlying pathophysiology of depression [171].

Interestingly, the use of purified or DHA-enriched oils was not successful in treating depression, postnatal depression or OCD [112, 172, 173]. This finding is in contrast to the greater face validity of DHA, which is the major brain omega-3 fatty acids and lower in brain tissue of depressed suicide victims [174].

3.4.3 Omega-3 fatty acids RCTs in minors

To our knowledge no omega-3 fatty acids RCT in teenagers with MDD has been published so far. However, the above mentioned small pilot omega-3 RCT in prepubertal children with childhood-onset depression showed the largest effect size (SMD=1.2) of all omega-3 fatty acids RCTs in MDD [167] Most other omega-3 fatty acids RCTs in children were done in attention deficit and hyperactivity disorders (ADHD). Bloch et al meta-analysis [175] including 699 ADHD children of ten RCTs between 7 – 12 years found a beneficial effect in favour of omega-3 fatty acids with a SMD of 0.31 with no evidence of publication bias and a significant dose dependency; RCTs using a daily dose of 500 to 750mg EPA were the most effective ones [176]. But the effect size of stimulant treatment with methylphenidate or dexamphetamines is still two to three times stronger compared to omega-3 fatty acids. Future research has to address the question if subgroups of ADHD patients may benefit more from omega-3 fatty acids (e.g. those with co-morbid depression, or low baseline levels of omega-3 fatty acids or increased inflammatory mediators) [177]. Omega-3 fatty acids also seem to help children with developmental coordination disorders that often present with ADHD symptoms [178]. Furthermore, a RCT in adolescents with a first-episode psychosis [179], as well as a RCT in adolescents at ultra-high risk for psychosis [180] were performed. The use of omega-3 fatty acids in prevention of mental disorders is promising, yet an avenue to be further explored. The main applicant is the senior author of a pilot RCT in 81 adolescents at ultra-high risk (UHR) for developing a psychotic disorder (mean age 16.4) that used 1.2g of an EPA-enriched omega-3 fatty acids oil as a sole agent [180]. 27.5% in the placebo group progressed towards a first psychotic episode compared to only 4.9% in the omega-3 fatty acids group. This pilot study has recently been replicated but study results are not available yet (the NEURAPRO study [181]). Another multinational multi-centre omega-3 RCT in prodromal schizophrenia will start in the near future (the PURPOSE trial), where the main applicant acts as

a consultant. A further study carried out by the main applicant demonstrates that omega-3 fatty acids augmentation treatment in first episode psychosis may result in a better tolerability (less EPS, less sexual side effects) and faster response to antipsychotic medication, however at the end of the three month treatment period, there was no difference in treatment effects on all primary outcome measures between active and placebo [179] (the main applicant was supported by a young investigator fellowship of the Swiss National Science Foundation (University of Basel)). A meta-analysis of Dr. Fusar-Poli and the main applicant came to the conclusion that omega-3 fatty acids in established (but not prodromal) schizophrenia have no or only minor additional efficacy compared to currently available treatments (also not on depressive symptoms in schizophrenia) [182].

3.4.4 Omega-3 fatty acids RCTs in other psychiatric conditions

There is some evidence that omega-3 fatty acids augmentation may have some beneficial effects in bipolar affective disorders [124], in particular against depressive symptoms [126, 183]. Furthermore, EPA-enriched omega-3 fatty acids may also attenuate impulsivity in patients with Borderline Personality Disorder [142, 184] and incarcerated young males [185]. The latter findings may be of particular importance for male pMDD individuals that sometimes presents with impulsive and aggressive behaviour rather than sadness [132]. A recently published trial in adolescents with conduct disorders highlights the importance to implement long study durations (e.g. one year) to be able to demonstrate potential positive effects of omega-3 fatty acids on difficult to treat behavioural traits [186]. Worth mentioning is a recent RCT in premenstrual syndrome (PMS) showing some beneficial effects on depression, nervousness, anxiety, lack of concentration and a reduction of somatic symptoms such as bloating, headaches and breast tenderness [139].

3.4.5 Unpublished RCTs with Omega-3 fatty acids in pMDD

We searched the US National (https://clinicaltrials.gov/) and the WHO trials registries (http://apps.who.int/trialsearch/) using “omega-3 fatty acids”, “depression” or “MDD”; “child” or “children” or “adolescents” to identify planned or currently on-going RCTS. We identified five RCTs: 1. Arnold et al from NIMH (NCT01341925) recruit children from 7-14 with MDD or dysthymic disorder. Gabbay et al (NCT00962598) from the Mount Sinai School of Medicine recruit 12-19 year old adolescents meeting the criteria for MDD using psychopathology and proton magnetic resonance spectroscopy as primary outcome. McNamara et al (NCT00511810) recruit children and adolescents with MDD using psychopathology and functional magnetic resonance imaging (fMRI) as primary outcomes. All these studies have target numbers of 40 to 80 and often use biological outcome measures as primary outcomes. Simon Rice et al (ACTRN12613001352796) aim to recruit 400 depressed adolescents and young adults aged 15 to 25 in a nationwide multi-centre RCT of omega-3 fatty acids supplementation (the YoDa-F study) [187]. Participants will be recruited in headspace centres across Australia (http://headspace.org.au/about-us/). Headspace centres are community health centres with a very low threshold (not like the specialist mental health service). The clients accessing the Headspace centres are comparable with our youth counselling services of the social welfare system in Zurich (e.g. Sozialzentren der Stadt Zürich oder die regionalen Jugend- und Familienberatungsstellen). The majority of the YoDA-F participants will be outpatients and most likely encompass less severe cases than in our planned omega-3 pMDD study that will solely

enrol patients treated in specialised child and adolescent psychiatric inpatient and outpatient services. We can therefore expect that our sample will have a higher baseline depression severity. Furthermore, we will recruit in fewer centres compared to the YoDa-F study (across Australia 60 headspace centres are in the process of being established). The latter two aspects are of major concern for the YoDa-F study, as the Australian study will most likely need to deal with a large placebo-effect which might be quite challenging as demonstrated by two reviews [188, 189]). Based on previous studies conducted by the main applicant who worked at ORYGEN Youth Health, Melbourne Australia from 1999-2006 [104, 179], about 35% of participants in the YoDa-F study will be under the age 18. Our study will include younger pMDD cases with higher baseline depression severity compared to the Australian study. Finally, our study will implement several means to reduce the known placebo effect in adolescent depression studies (see below). Rice et al targets a n=400 based on the assumption of a relatively small effect size of 0.31, probably to account for exactly these challenges associated with their study design [187]. According to the WHO trials registry, the study has not started recruitment yet (18.9.2015).

3.5 Dose Rationale

Omega-3 fatty acids have been tested in many different conditions. However, a wide array of omega-3 fatty acid compositions in different doses and EPA/ DHA ration has been used. In dyslipidaemia and cardiovascular disorders the efficacy has been proven in many studies encompassing several thousand patients with an excellent safety profile [190]. Thousands of hypertriglyceridemia patients have been treated with sometimes very large doses of omega-3 fatty acids up to 10g per day with no life-threatening side effects [191]. Omega-3 fatty acids doses up to 4g per day have also been used in minors with metabolic disorders without any major tolerability issues [192, 193].
Most successful omega-3 fatty acids RCTs in adult MDD used around 1000mg EPA per day. As outlined above [163], any study drug should contain an EPA content > 60% of all omega-3 fatty acids as only those preparations seem to be more effective than placebo; a small amount of DHA seems to be favourable in comparison to purified Ethyl-EPA (probably by inhibiting the conversion of EPA to DHA). In prepubertal children Nemets et al [167] used 400mg EPA and 200mg DHA. Bloch et al meta-analysis in ADHD children [175] found a significant dose dependency with RCTs using a daily dose of 500 to 750mg EPA having the biggest effect. In the proposed study we decided to use for the active treatment a daily dose 500mg EPA/ 250mg DHA in our 8 to <13 year olds, and 1000mg EPA / 500mg DHA for the 13 to <18 years olds (which corresponds with the omega-3 fatty acid doses used in adult MDD RCTs).

3.6 Explanation for Choice of Comparator (or Placebo)

Placebo capsules will be matched to the active comparator in size and appearance and will contain medium chain triglycerides (MCT). MCT oils do not contain any unsaturated fatty acids and therefore are very stable and do not show tendency to rancidity. In addition, MCT oils in these quantities do not have any pharmacological effect. Worth mentioning is that many different forms of placebo have been used in previous trials, such as canola, sunflower or olive oil. However, these oils may be physiological active (e.g. olive oil is a constituent of the Mediterranean diet that

has shown a range of health benefits [194, 195]). Mineral or liquid paraffin oils as placebo (like the first trial of the PI [179] or the YoDa-F trial [187]) may cause diarrhea, in particular in children that may already react quite sensitively to more than 1g of mineral oil.
A major concern is often that omega-3 fatty acids capsules could be “unblinded” because of the fishy taste. However, the recent formulations have nearly no fishy taste anymore. To further minimise the risk of “unblinding”, we will put a very small amount of fish oil into the placebo capsules to mimic the taste. A recent double blind controlled trial addressing exactly this question demonstrated that test persons were not able to differentiate between active (omega-3 fatty acids) and placebo [198]. Another concern could be the size of the capsules. Burgerstein will produce specially manufactured child capsules as study medication that can easily be swallowed by children. In addition, both capsules will contain natural orange oil for a pleasant odor when opening the bottle and so optimizing compliance.

3.7 Risk / Benefits

Children and adolescents suffering from mental problems, in particular depression often do not seek help because of the stigma associated with mental illness [196]. Parents are hesitant to see professionals because they are ashamed that they may have failed as parents. Three out of four patients with a mental illness are not treated [197-199]. Psychiatrists are often looked at as “drug vendors”. Being open to novel treatments such as omega-3 fatty acids that are closely linked to lifestyle, food and general wellbeing may indirectly encourage families of children and adolescents suffering from mental illness to seek help sooner. We can have the best and most effective treatments, but our patients need to take them early enough. In other areas of medicine, early recognition and treatment has become the gold standard, but in psychiatry the opposite still often applies. However, the consequences for children and adolescents with an emerging serious mental illness such as depression are underestimated, as the duration of untreated illness is an important prognostic factor for long-term outcome in depression [9]. While patients and their families wait and see, consequences of an untreated depression for social, psychological and neurobiological development may be neglected for far too long. Omega-3 fatty acids as a benign first-line treatment are likely to be a well-accepted first step in a comprehensive treatment plan and may serve as a “door opener” to engage patients and families for other more invasive treatments if omega-3 fatty acids have failed. But if the omega-3 fatty acids prove ineffective, other more effective treatments might be delayed because of an unjustified trial of omega-3 fatty acids as a sole, but ‘ineffective’ treatment. There is a need to investigate novel evidence based treatment approaches as already today depression causes the largest burden of disease in the age group from 10 – 24 [10]. Despite over a dozen well-designed relatively small RCTs in adult MDD and several critical and well-performed meta-analyses, the available evidence is not conclusive enough to promote or refute the use of omega-3 fatty acids in standard clinical care (see above) [51]. There is a need to perform larger scale studies (similar to Phase III trials of novel drugs). Further small scale RCTs are unlikely to provide any additional value, as they can easily be questioned whatever outcome they will have.
Omega-3 fatty acids are potentially even more efficacious in paediatric depression than in aMDD because of the bigger brain plasticity [200] and the underlying on-going major developmental processes, e.g. via the modulation of pruning and myelinisation that all involve a large turn-over of LC-PUFAs. The use of omega-3 fatty acids in child development and critical transition periods such as the onset of puberty might therefore be very relevant for its efficacy (see above: Potential

mechanism of action). In line with this assumption are some animal studies and pilot studies in children that have shown some unexpected large effect sizes [167, 180].
Psychiatric patients often seek complementary alternative medicine (CAM) in addition to school medicine [201] that usually offer non-evidence based treatments. However, such treatments may delay or even hinder access to evidence-based treatments. Patients often do not inform their doctors about such alternative treatments [202]. The current lack of evidence-based data for CAM underscores the need for controlled trials like the proposed one that may lift omega-3 fatty acids from an alternative treatment approach into the field of evidence-based best practice.
If omega-3 fatty acids prove efficient in pMDD, the consequences will be significant for several reasons 1.) routine clinical care, 2.) the health food industry (they are already now testing encapsulated omega-3 fatty acids to introduce larger doses of omega-3 fatty acids in processed food, and 3.) in basic and clinical research, for new drug targets, as well as future development and use of bioactive lipids in treatment of affective disorders. In particular, the new avenues for the health food industry may finally even have an impact on the prevalence and incidence of depressive disorders (similar to the addition of iodine to the table salt which contributed to the near disappearance of thyroid goitres in Switzerland). At this stage, the epidemiological, preclinical and limited clinical evidence supports such a rather optimistic view of the omega-3 story in depression [49, 203, 204].
In the field of lipid research, increasing evidence is emerging that pro- and anti-inflammatory mediators are of pivotal importance for brain development, synaptic plasticity and brain functioning [62, 63, 101, 205]. The identification of inflammatory and/or bioactive lipid markers that are predictive for omega-3 fatty acids response might open up new treatment targets for future drug development for MDD. Our study includes the a priori hypothesis that in particular pMDD cases with elevated inflammatory mediators may benefit from omega-3 fatty acids treatment. If such an approach can be proven, the measurement of such markers will identify omega-3 fatty acids responsive patients contributing towards a personalized and evidence-based decision-making process to indicate who might benefit at what point from the use of EPA-rich omega-3 fatty acids – and possibly even more importantly, under what conditions omega-3 fatty acids are not helpful.
The relationship between important psychopathological features such as suicidal ideation or overt aggression and bioactive lipids is another more explorative aim of the study that may help to further characterize subgroups of pMDD at increased risk of harm, a group of subjects where early detection and monitoring is critical.
The establishment of a BioBank in accordance with the Swiss HFG/KlinV/HFV regulations of this well-characterized patient population with medium-term follow up data will enable further exploration of identified candidate biomarkers at a gene and protein level. There are very few tissue repositories of pMDD or first onset depression cases. In our pMDD sample effects of chronicity of illness and past treatments are minimized enabling the investigation of the underlying neurobiology with minimal confounders. The resulting tissue repository will therefore become a source for other researchers to investigate the underlying causes and dysfunctions of pMDD and MDD. The repository will be open to the wider research community in accordance with the Swiss HFG/KlinV/HFV regulations.

3.8 Justification of Choice of Study Population

Depressive disorders already emerge in childhood and adolescence, but no pharmacological treatment with antidepressant medication is approved in Switzerland. All prescribed treatments are “off label”. There is some evidence that in particular adolescents respond with an increase in suicidal ideation in the early phase of SSRI treatment. There is a need for novel treatment as the treatment with cognitive behavioural therapy might not always be feasible or sufficiently effective. Omega-3 fatty acids in adults have shown some beneficial effects in MDD, however, so far only small scale RCTs have been performed. Even so meta-analyses have shown moderate effect sizes in adults, results may not be directly transferrable to paediatric MDD given the bigger brain plasticity and the underlying on-going major developmental processes, as described above (see chapter 3.4.3). Therefore, the inclusion of minors is necessary. Participation in the study is preceded by counselling by the treating doctor / psychologist or investigator, during which the minor and its legal representative/parents are informed about the nature and entire course of the study. Age-adjusted patient information sheets for adults and for minors are available. Informed consent will be sought at the same time by the minors themselves and their legal representatives/ parents. The explicit wish of a minor who is capable of forming an opinion and assessing this information to refuse participation will be followed; in such case, the minor can be withdrawn from the study at any time. If a minor subject does not want to participate, they will not be included in the study. This is also explicitly stated in the patient information sheet.

4 STUDY OBJECTIVES

4.1 Overall Objective

The key research question is if omega-3 fatty acids rich in EPA are an effective and safe treatment for pMDD. In addition, we want to investigate if inflammatory mediators, LC-PUFAs and their bioactive lipid markers are response predictors for omega-3 fatty acids treatment or not. Furthermore, we want to investigate if LC-PUFAs and bioactive lipids are associated with particular phenotypic characteristics, e.g. suicidal ideation, impulsivity, cognitive impairment, symptom severity and illness course. Finally, we want to establish a tissue repository (BioBank) in accordance with the HFG/KlinV/HFV.

4.2 Primary Objective

Our primary objective is to investigate the therapeutic efficacy of omega-3 fatty acids in pMDD (WH 1).

4.3 Secondary Objectives

Our secondary objective is to demonstrate a clinical meaningful effectiveness of omega-3 fatty acids (WH 2a-d). Furthermore, we want to investigate immune-modulatory markers, Lc-PUFAs and their bioactive lipids and inflammatory mediators (e.g. cytokines) as predictors for Omega-3 fatty acids treatment response (WH 3a-c). Additionally, we want to investigate if phenotypic characteristics (e.g. suicidal ideation, impulsivity, aggression, anhedonia, hopelessness, cognitive impairment and illness course) are associated with particular LC-PUFAs or their bioactive lipids (WH 4a-d).

Finally, we want to establish a Biobank of this phenotypically well-characterized pMDD sample with longitudinal outcome data that will be made available for future research in accordance with the Swiss HFG/KlinV/HFV.

4.4 Safety Objectives

Our safety objectives are to investigate if Omega-3 fatty acids have an impact on suicidal ideation (in particular because to the reported increased suicidal ideation in association with SSRIs), and is well tolerated in children and adolescents (WH 5a).

5 STUDY OUTCOMES

5.1 Primary Outcome

WH 1 – Based on the findings of Omega-3 fatty acids treatment studies in adult MDD [161, 163, 166], and one small RCT in prepubertal children [167], we assume that we will find a Treatment x Time interaction for the CDRS-R total score in pMDD in favour for Omega-3 fatty acids treatment. The recovery and remission rate based on the structured clinical interview (K-SADS-P) in conjunction with a CDRS-R total score ≤28, as well as the response rates (a 30% decrease in total baseline CDRS-R score after the placebo-lead-in week as proposed by Emslie et al [206]) will be higher in the Omega-3 fatty acids compared to the placebo group at week 12 (response, remission rate) and 36 (recovery, remission rate).

5.2 Secondary Outcomes

WH 2a – We hypothesize that more pMDD patients in the placebo group will be put on antidepressant medication compared to the Omega-3 fatty acids group.

WH 2b – We hypothesize that the Omega-3 fatty acids group will achieve a higher level of functioning (a higher CGAS score) and a better quality of life (overall and within the five dimensions as assessed with KIDscreen-CAT -27) compared to the placebo group.

WH 2c – We hypothesize that the Omega-3 fatty acids group will spend fewer days in hospital and will have a lower outpatient service use (measured with the attrition check list) compared to the placebo group also resulting in a reduction in overall costs (calculated via TARMED points).

WH 2d – We hypothesize that the overall retention rate will be higher in the Omega-3 fatty acids group compared to the placebo group (or, in other words the placebo group will have more and earlier drop outs due to lack of efficacy). This hypothesis will be tested by two means: 1.) in the ITT sample independent if they received an antidepressant in the due course of the trial, as well as 2.) in a modified ITT sample, where those subjects that are put on an antidepressant will be considered as drop-outs from the day they received the first dose of an antidepressant.

WH 3a – Based on the recent meta-analysis [62] showing increased inflammatory mediators in adult MDD, we assume that at least 50% of participants will have a baseline increase of inflammatory mediators (in particular hsCRP, IL-1, IL-6) and a decrease of anti-inflammatory markers (IL-10). Based on a recent Omega-3 fatty acids treatment study in adult MDD [63], we hypothesize that Omega-3 fatty acid response will be predicted by the ratio between pro- and anti-inflammatory markers.

WH 3b – Based on the meta-analysis by Lin et al [54] as well as the study of Potalla et al [61] in pMDD, we hypothesize that about 80% of participants with pMDD will have a reduction in red blood cell Omega-3 fatty acids content, in particular EPA and DHA (used to calculate the Omega-3 index® [207, 208]). We hypothesize that those with an omega-3 index® <4 at baseline will benefit more from Omega-3 fatty acids treatment, whereas those with an omega-3 index >5 (approx. 20%) will not show a marked benefit from Omega-3 fatty acids treatment. We further hypothesize that a high n-6/n-3 Omega ratio at baseline will be associated with a better response to Omega-3 fatty acids.

WH 3c – We hypothesize that low levels of direct metabolites of EPA, in particular HETEs and the E-series-resolvins (RvE1 & 2) will be a strong predictor of Omega-3 fatty acids response.

WH 4a – Based on the meta-analysis of Lin et al [54], we hypothesize that severity of depressive symptoms will be associated with a pro-inflammatory state, as well as an increase in omega-6/3 ratio and a decrease in omega-3 index. Normalization of these parameters will correlate with improvements in depression scores (CDRS-R, DIKJ).

WH 4b - Based on the study by Huan et al [52], we assume an inverse correlation between Omega-3 fatty acids and suicidal ideation (SIQ-Jr), in particular high levels of EPA will protect against suicidal ideation.

WH 4c – Based on the study by Beier et al [209] we assume that low Omega-3 fatty acids, in particular low EPA levels (and a low omega-3 index) will be associated with affect dysregulation, impulsivity/ emotionality (IES-27) and overall psychopathology.

WH 4d – Based on Kiecolt-Glaser et al [205], we hypothesize that high levels of overall stress (as measured with the PSS scale) will correlate with low levels of Omega-3 fatty acids, a pro-inflammatory state, increased saliva cortisol, and high levels of PUFAs oxidation products as

measured by F2 Isoprostanes in the urine and be inversely correlated with the Conner-Davidsons Resilience Scale.

5.3 Other Outcomes of Interest

Cognitive deficits in MDD are consistent, replicable, nonspecific and clinically significant. Pronounced deficits in executive function (≥1 SD below the normative mean) are evident in about 20-30% of individuals with adult MDD. A recent meta-analysis of children and adolescents with MDD demonstrates that they perform 0.194-0.772 ($p < 0.001$) standardized mean differences worse than healthy controls in neuropsychological test procedures. The most pronounced deficits of children and adolescents with MDD were seen in inhibition capacity (SMD = 0.772; $p = 0.002$), phonemic verbal fluency (SMD = 0.756; $p = 0.0001$), sustained attention (SMD = 0.522; $p = 0.000$), verbal memory (SMD = 0.516; $p = 0.0009$) and planning (SMD = 0.513; $p = 0.014$) [210], with some conflicting results [211]. Other replicated abnormalities are in the domains of working memory, attention, and psychomotor processing speed. Cognitive deficits may account for the largest percentage of variance with respect to the link between psychosocial dysfunction (notably workforce performance) and MDD [212]. We expect that those with the lowest omega-3 index and the highest omega-6/omega-3 index will have the most severe impairments in cognition. We expect that those patients are the ones that benefit most from omega-3 fatty acids.

5.4 Safety Outcomes

WH 5a – We hypothesize that Omega-3 fatty acids treatment will be a safe treatment with no drug-related SUSARs, in particular with no increase in suicidal ideation (SIQ-Jr).

6 STUDY DESIGN AND COURSE OF STUDY

6.1 General Study Design and Justification of the Design

We will apply a 36week, multi-centre, double-blind, placebo-controlled, fixed dose, parallel group design to study the efficacy of Omega-3 fatty acids on pMDD. The double blind RCT includes a 12week acute treatment phase and a 24 week maintenance phase. A single-blinded placebo-run in phase will try to minimize the initial placebo response and contribute to the elimination of false positive inclusions in rapid placebo responders. In order to do this reliably, 220 patients aged 8 – 17 years with a primary diagnosis of depression will be randomly allocated to receive a daily dose of omgea-3 fatty acid (500mg EPA / 250 mg DHA for the 8 < 13 years old; 1000mg EPA / 500mg DHA for the 13 < 18 years old) or matching placebo capsules for 36 weeks. A study of this size should have enough power to detect whether omega-3 fatty acid is an efficient treatment for pMDD.

After written consent and extensive screening of the patients, the 7 to 10 days placebo lead-in phase will commence. After completion of the placebo lead-in phase, the Children's Depression Rating Scale (CDRS-R) will be applied to determine whether the patients still fulfill the criterion of a moderate depression (score > 40), and then the patient will be randomly assigned to a study group. After an extensive baseline examination, subsequent assessments will occur at 6 weeks, 12 weeks, 24 weeks and 36 weeks. At each assessment depressive symptoms and other psychological variables will be assessed using a variety of different rating scales. Assessment of cognitive functioning occurs at baseline, week 12 and week 36. Blood, urine and saliva will be taken at the screening visit and after 12 and 36 weeks.

Assignment to a treatment arm will be performed randomly to eliminate the possible influence of arbitrary allocation of subjects on the study results. Using random assignment, known and currently unknown factors potentially influencing outcomes (e.g. demographic factors, findings at screening examination) are evenly distributed among the two groups, which increases the validity of statistical analyses. Administration of medication will be double-blind in order to minimize the influence of expectancy regarding the type of medication on data collection and analysis. A placebo arm is included as the effectiveness of omega-3 fatty acids in the treatment of depression will be examined, and no other psychopharmacological treatment is recommended in pMDD in children and adolescents. All the children will receive concomitant standard treatment as advised by their clinicians.

The study examines the effectiveness of omega-3 fatty acids in children aged 8-17 years old. Inclusion of minors is essential as no current pharmacological treatment for pMDD exists and data of adults might be not transferrable as fatty acids have been implicated in the process of adolescent brain maturation.

6.2 Study Duration and Study Schedule

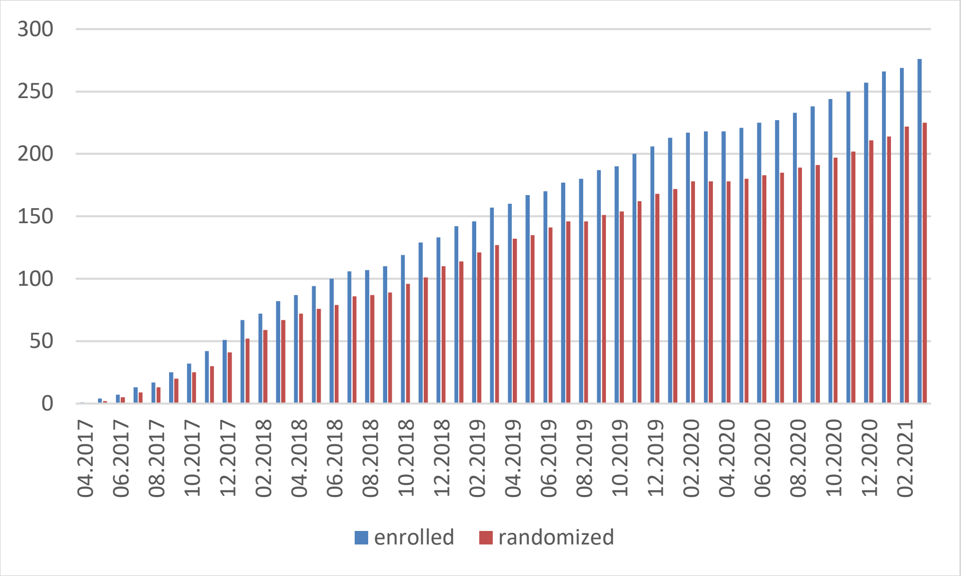

Our recruitment goal is to include 220 patients with at least one follow-up assessment in the trial. Until now, we have randomized 225 patients. However, 21 patients dropped out before reaching the first follow-up assessment and thus, these need to be replaced. Therefore, we will need to randomise another 20 patients in order to reach our target of 220 patients with at least one follow-up assessment. We estimate that we will reach this target in May 2021. In addition, we will recruit up to 20 additional patients to account for the ones who started antidepressant treatment within the first 6 weeks of the trial. This will give more power to our a priori-defined analyses in which start of antidepressant treatment will count as a drop out criteria. The intention-to-treat analyses will still include all patients who were enrolled in the trial. Last follow-up assessments will then be carried out in March 2022 at the latest. After, we will close the database and start analysing the data set.

6.3 Methods of Minimising Bias

6.3.1 Randomisation

The active and maintenance treatment phase consists of a 1:1 randomized placebo-controlled allocation sequence for the 36-week study period. A dynamic computerized 1:1 randomisation stratified for age, gender, type of treatment (in- or outpatient) and hsCRP across each site will be applied. A minimisation algorithm integrated in SecuTrial® will provide a balanced distribution between the two treatment groups (active/ placebo) for each site and strata.

Access to the SecuTrial® generated randomisation list will be restricted to the Clinical Trial pharmacist, in case of an interims analysis to the IDMC, upon request to the regulatory authorities

and to the data management at the USZ CTC. The study medication will be labelled at the KAZ and will be sent out to the study sites in two batches.

The algorithm and allocation process is implemented in SecuTrial and the development of the algorithm is done by the data manager of the Clinical Trial Centre and cannot be influenced by the investigators. The clinicians and researchers who enrol the patients just provide the relevant stratification data. The minimization algorithm contains an element of chance so that even with the knowledge of all previous allocations the next allocation cannot be determined.
Participants, parents, treating clinicians and doctors, research assistants, all involved researchers including the PI remain blind to the allocation of participants to the study drug (active/ placebo). SecuTrial® only provides one single randomisation number making it impossible to guess the allocation. Data analyst (PD Dr. rer. nat. Ulrike Held, Dr. Alex Roth) will only get access to the randomisation code list after completion of data entry, double entry of a subset of data and final closure of the database. The only person who has access to the computer-generated allocation code is the data manager of the CTC and the clinical trial pharmacist of the Clinical Trials Pharmacy of the Universities of Zürich (*Kantonsapotheke Zürich* KAZ).
If deemed necessary, the IDMC is allowed to review unblinded data as further specified in the IDMC SOP.

6.3.2 Other Methods of Minimising Bias

Based on two comprehensive meta-analyses on placebo-response [188, 189] and a RCT comparing fluoxetine versus placebo in pMDD with a low placebo-response [206] we implemented a number of measures to minimize the placebo-response:

- As the number of sites is a strong predictor of placebo-response, we have limited the number of sites (the lead centre (ZH), one additional academic centre (BS), as well as three further Cantons (TG, SG, BL) in the proximity of the lead centre (ZH)). Many of the senior clinicians in the regional centres were trained or worked as consultants in the academic lead centre (ZH). Furthermore, the academic and non-academic centres have a joint training program for their trainees for several years providing a good foundation that all participant will undergo a similar treatment approach.
- We will implement regular meetings with therapists from the participating centres to improve adherence with the S3 guidelines.
- The number of research assessments has also been identified as a predictor for placebo-response [188, 189]. We have limited the research assessments to three assessments during the active treatment phase (baseline, 6 and 12 week), and two further assessments in the maintenance phase (24 and 36 week), what is much lower than in comparable trials.
- Based on previous RCTs in pMDD [213], we expect a 10-20% drop in overall CDRS-R score during the 7-10 days placebo-lead in phase. If a participant drops below a CDRS-R total score <40 at the end of the placebo lead in phase, the participant will not be enrolled in the double-blind randomized study period (as he does not meet inclusion criteria anymore). Similar strategies were already applied in other RCTs of pMDD and could attenuate placebo response and eliminated false-positive enrolments (a participants that has such a dramatic decrease in symptom load within the placebo lead-in phase is unlikely to suffer from “true MDD”, but more likely from an adjustment disorder) [213, 214]. We will

offer placebo-responders to be followed up separately as a non-randomized comparison group to better understand the characteristics of such placebo-responders (an important "by-product" of the proposed study [215]). Finally, to reduce the likelihood that a site can estimate a possible allocation sequence, we will not provide stratification blocks to the clinicians (see allocation concealment) [206].

- To avoid a selection bias, we decided to allow the use of SSRIs as clinically required. Elsewise, we would probably not be able to include a representative sample of moderately or severely ill children meeting criteria for major depressive disorders. However, the latter may result in an unequal distribution of antidepressant use between the treatment arm and increase the response rate across both treatment arms, making it less likely to address the primary aim of the study. To address this flow of the study design, we decided to include a further a priori defined analysis approach: We will reanalyse the primary outcomes using "start of an antidepressant medication" as a "drop out criteria" and use multiple imputation to simulate the estimated course of illness. This will allow us to investigate if omega-3 fatty acids as a sole treatment have antidepressant activity or not. As interim analyses showed that about 20 of our patients started antidepressant treatment within the first 6 weeks (prior to the first follow-up assessment), we will recruit up to 20 additional patients to gain more power for our secondary analyses.

6.4 Unblinding Procedures (Code break)

An Emergency Code Break will be available through SecuTrial®. The only person authorised to unblind a patient is the sponsor-investigator Dr. Gregor Berger, and the two principal investigators Prof. S. Walitza and Prof. K. Schmeck who can do so in case of an emergency unblinding, e.g. in case of a Suspected Unexpected Serious Adverse Reaction (SUSAR). The unblinding procedure can be triggered by SecuTrial®. Once a patient is unblinded by SecuTrial®, all users of the software will know the patient's assigned treatment group and the unblinding procedure cannot be reversed. Therefore, the participant will be considered a drop-out. A drop-out visit will be performed in a clinically meaningful time frame.
Emergency unblinding is indicated in the following situations:

- Unblinding is necessary for the subjects emergency treatment at the clinicians or investigator's discretion.
- Unblinding is required by local laws or regulations (in case of SUSAR).
- The IDMC decides that unblinding is necessary for proper study management of the subjects and the overall safety of the other subjects in the study.

After closing of the database every site will be informed about each subject's assigned treatment group.

7 STUDY POPULATION

A total of five Cantons of the German part of Switzerland agreed to participate in the multi-centre omega-3-pMDD trial. The five Cantons include two academic centres (Zürich ZH, Basel-Stadt BS) as well as the inpatient and outpatient services of Canton St. Gallen (SG), Thurgau (TG) and Basel-Land (BL) encompassing all public child- and adolescent psychiatric services in a catchment area of 2.7 Million habitants (ZH 1446.4M, BS 190.6M, SG 495.8M, TG 263.7, BL 283.2).

Based on the referral rates of previous years for each site, all participating centres provided their estimated annual referral rates of eligible patients for screening based on following codes ICD-10 F32, F33, F34.1, F38, F39, F43.2, F41.2, F92. Based on this information and based on the experience of each centre in recruitment, we estimated the following number of enrolments per site:
The main centre, the Clinic for Child and Adolescent Psychiatry and Psychotherapie KJPP in Zürich encompasses seven outpatient clinics (*Ambulatorien*), two inpatient facilities (Neumünsterallee/ Brüschhalde), three day clinics and the policlinic. The KJPP ZH has over 2000 new referrals per year. We expect that about 400 cases will be eligible for screening and that about half of them will meet inclusion criteria. We estimate that about 15-20% of those cases will agree to participate in the study resulting in a conservative enrolment estimate of 35-40 cases per year.

The sites have provided following recruitment estimates based on previous recruitment experiences, annual reports and PSYREC statistics:

- 64 at KJPP ZH
- 33 at the Child and Adolescent Psychiatric Services of Basel
- 25 at the Clienia Klinik Littenheid
- 25 at the Klinik Sonnenhof in Ganterschwil
- 24 at the child- and adolescent outpatient departments of the Canton St. Gallen
- 24 at the psychiatric outpatient services Thurgau
- 25 at the child- and adolescent outpatient departments of the Canton Basel-Land

We will first start to screen and recruit in the seven official centres including solely child and adolescent psychiatric services. If we will not achieve the expected numbers of recruits within the first six months since the first inclusion of a trial patient, we will extend our advertising strategy to paediatric centres of the Cantons Zürich, Basel-Stadt, Basel-Land, St. Gallen and Thurgau, as well as write letters with our study flyers to private child and adolescent psychiatrists, as well as paediatricians and general practitioners.

7.1 Eligibility Criteria

7.1.1 Inclusion Criteria

Participants fulfilling all of the following inclusion criteria are eligible for the study:

- Male or female in- or outpatients of a participating centre (ZH, BS, SG, TG, BL).
- Children aged 8 to ≤ 13 years or teenagers 13 to < 18 years at time of study entry (inclusion up to the 18th birthday is possible, as long as participants remain in the responsibility of a participating child and adolescent psychiatric service until completion of the 36 week trial period).
- Written informed consent of the subject (Appendix Informed Consent Forms). For individuals younger than 18 years of age the parents / legal representatives need to give consent, and the subject need to provide assent.
- Depressive symptoms of at least moderate severity as defined by a CDRS-R total score of ≥40.
- A present primary diagnosis of major depressive disorder (single or recurrent) as defined by DSM-IV criteria and confirmed in the K-SADS-PL.
- Able to swallow the study medication without difficulty.
- No clinically significant laboratory findings in haematology, chemistry, and urine analysis at study entry based on the judgment of the treating doctor.

7.1.2 Exclusion Criteria

The presence of any one of the following exclusion criteria will lead to exclusion of the participant:

<u>Exclusion criteria:</u>

- Contraindications to the class of drugs under study, e.g. known hypersensitivity or allergy to class of drugs or the investigational product (i.e. a checklist will be provided asking patients about intolerance or hypersensitivity to fish and whether they have a coagulation disorder. Coagulation parameters will be additionally assessed through laboratory screening),
- More than 4 weeks of regular omega-3 supplementation (>2 daily capsules standard strength providing > 600mg combined EPA/DHA) within the last 6 months,
- Women who are pregnant or breast feeding,
- Intention to become pregnant during the course of the study,
- Lack of safe contraception, defined as: Female participants of childbearing potential and who are sexually active, not using and not willing to continue using a medically reliable method of contraception for the entire study duration, such as oral, injectable, or implantable contraceptives, or intrauterine contraceptive devices, or who are not using any other method considered sufficiently reliable by the investigator in individual cases,
- Pre-existing neurological (such as brain tumour, temporal lobe epilepsy, HIV encephalopathy) or medical conditions (ICD-10 F06-F07) likely to be responsible for the depressive symptoms,
- Laboratory screening values considered clinically relevant by a medical doctor for transaminases, thyroid hormones or coagulation parameters,
- Known or suspected non-compliance,

- Inability to follow the procedures of the study, e.g. due to language problems, psychological disorders, dementia, etc. of the participant,
- Participation in another study with investigational drug within the 30 days preceding and during the present study,
- Previous enrolment into the current study,
- Enrolment of the investigator, his/her family members, employees and other dependent persons,
- Substance dependency (ICD-10 F1x.2) within the last six months (but not misuse),
- Life-time diagnosis of schizophrenia and related disorders (ICD-10 F20-F25),
- Life-time diagnosis of bipolar affective disorder in the K-SADS-PL (ICD-10 F30, F31),
- Current eating disorders within the last six months (ICD-10 F50.0 & F50.2),
- Mental retardation (ICD-10 F70-73),
- Pervasive developmental disorders (ICD-10 F84.x).

7.2 Recruitment and Screening

Children and Adolescents meeting criteria for clinical depression will be recruited from mental health in- and outpatient units of the Cantons Zürich, Basel-Stadt, Basel-Land, St.Gallen and Thurgau.

There will be three different enrolment strategies:

1) The study site investigator will check all potential eligible new referrals on a weekly basis based on the clinical entry diagnosis. The investigator will check the clinical file and if a patient is potentially suitable for the study, he will contact the responsible clinician and discuss if the patient may be eligible or not. If the treating doctor / psychologist agree on the suitability of the patient, the family will be contacted by the investigator.
2) The treating doctor / psychologist contact the investigator if he has a patient who might be eligible to participate.
3) Depressed children and adolescents or their families learn of the study through other participants or through flyers and posters available at each study site and contact the investigator directly. In that case, the investigator will contact the treating doctor / psychologist to determine eligibility.
4) A study website, accessible to the public, will be established containing the same information as the flyer, poster and patient information sheets.

After the eligibility of a patient has been established, the clinician and/ or the investigator will contact the patient, his parents or his/her guardian and explore the willingness to participate in the study in accordance with ICH-GCP guidelines.
The subject will be informed about the nature and entire course of the study, potential individual benefits and personal risk shall be explained. This may be carried out by a trained research psychologist. Here it will be re-emphasized that participation is absolutely voluntary. Potential patients and their parents/ guardians are given sufficient time to read all the provided information (approved by the appropriate authorities), and clarify any questions with the treating doctor/

psychologist or investigator. Regarding data privacy, subjects will be informed about pseudonymous recording. The allocation of a unique study number that can be traced back to the patient if necessary will be used. The list with patients' names and contact details are kept with the principal investigators. Outside laboratories will only be provided with coded documentation not enabling them to identify the individual patient. The sharing of data will be done in accordance with ICH-GCP guidelines. In case subjects cannot agree to this central collection of data they cannot participate in the study. Participation only becomes possible after the subject as well as the investigator/doctor have signed the informed consent document. This consent can be revoked at any time without citing reasons and without any consequences. No examinations or other activities will take place before the informed consent procedure is completed. A copy of the consent form and the patient information sheet will be given to the patient.
In case the patient information sheet or the consent form change, patients will be informed immediately and relevant information will be passed on to the ethic committees for approval. New patient information and consent will be discussed in detail again, the patient will again be asked for written consent, and a copy of the documents will be given to the subject.

After written informed consent is provided, the patient is screened extensively for in- and exclusion criteria as described in detail in chapter 9.3.1.

7.2.1 Incentives

There may not be any financial inducement to enroll a patient into the study; no financial incentive should be offered except compensation and expenses for the legal representatives/parents or for travel expenses. As a compensation for the invested time and the burden caused by the blood taking procedure, patients will receive a cinema voucher or equivalent at following time points: baseline, week 12 and week 36.

7.3 Assignment to Study Groups

Once the patient/ parent/ guardian have given written informed consent, the investigator will enter the relevant information (Site,Gender, Age, Treatment Type (in- or outpatient) und hsCRP) in the SecuTrial® randomization form. SecuTrial® will then assign the subject to the group which leads to least imbalance within all strata. The software will then provide a randomization number from a list implemented previously and display only this number but not the group name. This number will be transmitted to the clinical trials pharmacy (KAZ) who will send out the trial medication accordingly. Numbered study medication sets will be sent out to the centres in advance with half of them consisting of the active treatment and the other one consisting of placebo capsules. Study medication will be sent out in two different batches. The randomization number of study medication sets will be provided by SecuTrial® to the research psychologist responsible for the study site, as well as the postdoctoral study coordinator. Every centre has slightly more medication sets to incorporate small deviations in recruitment numbers.

7.4 Criteria for Withdrawal/ Discontinuation of Participants

Clinicians/ treating doctors/ patients and their relatives can withdraw a patient at any time if they think it is in his best interest. To support clinicians in their decision making at what point a patients shall be withdrawn from the study for safety reasons, we defined following exit criteria.

Reasons to terminate a subject's participation:

- A subject withdraws her/his consent
- Non-compliance with medication (no intake of study medication for more than three out of seven days in two consecutive weeks)
- Daily omega-3 supplementation in addition to study medication (>600 mg combined EPA/DHA per day for more than one week)
- The clinician or investigator considers a subject's continued participation in the study to be unjustifiable on medical grounds (i.e. because of the emergence of another serious medical condition).
- The subject falls below a score of 40 on the Children's Depression Rating Scale at the end of the placebo lead-in phase.
- Acute Suicidality/ overt hostility: Clinicians and research assistants will monitor suicidality/hostility at each visit. If a clinician/ researcher considers the suicidal risk/ hostility as high requiring some sort of clinical action (e.g. a suicide watch plan as an outpatient, or restricted leave as an inpatient because of intense suicidal ideation/hostility), patients shall be withdrawn from the study if the clinician considers a potential connection with the study drug and a SUSAR shall be reported. The number and type of SUSARs will be compared between the treatment groups (active versus placebo).
- Intolerance to the study drug. Life-threatening side effect other than suicidality/ hostility (SUSAR): In case of an unexpected life-threatening side effect (e.g. a severe allergic reaction to one of the ingredients of the study medication), a patient must be withdrawn from the study.
- The development of a major axis one disorder listed under the exclusion criteria (e.g. a first psychotic or manic episode is considered as a serious adverse event).
- Unwanted but not life-threatening adverse events: If the side effect persists, the study medication shall be ceased and the patient discontinued due to lack of tolerability (an important further outcome criteria).

Given that drop-out is also one of our secondary outcome measures, patients who discontinued the study will not be replaced, except if the patient drops out prior to study visit 1 (six weeks) or meets criteria for non-compliance within the first six weeks of treatment. If a patient drops out of the study (e g. if he changes diagnosis to bipolar affective disorder or schizophrenia) we will perform a drop out visit at the earliest convenient time, as described in section 9.2.5
If somebody does not turn up to a study visit, we will contact the patient and his parents to clarify the reason of non-attendance and schedule a study visit at the earliest convenient time. To avoid "non-shows", we will perform follow up visits at their parent's home. During the acute treatment phase (visit 2, 3), research assessment range is $\pm$5 days, for the maintenance phase (visit 4, 5)

research assessment range is ±7 days. For the clinician-rated scales we allow a time window of ± 2 weeks to account for the usual contact frequency between clinicians and patients.

8 STUDY INTERVENTION

8.1 Identity of Investigational Product(s)

1.5 gram/day omega-3 polyunsaturated fatty acids will be given daily in six 0.6-g gelatin capsules, which contain a daily intake of 1000 mg of EPA and 500 mg of DHA. Patients younger than 13 years of age will receive half of the dose (three capsules a day) resulting in a daily intake of 500mg EPA / 250 mg DHA. This EPA:DHA ratio of 2:1 has been proven safe and effective in multiple clinical trials.
Additionally, the capsules will also contain mixed tocopherol (vitamin E) to prevent oxidation. The placebo-capsules will contain a small amount of fish oil to mimic the taste of the active compound. In addition, both types of capsules contain orange oil for a pleasant odour. The placebo capsules will be indistinguishable from the active treatment in form, size or taste.

8.1.1 Experimental Intervention

Treatment A (Omega-3 fatty acid)

- Omega-3 fatty acids
- Approx.168 mg EPA and 84 mg DHA per capsule
- Children < 13 years of age are advised to take 3 capsules resulting in approx. 500 mg EPA and 250 mg DHA per day
- Teenagers > 13 years of age are advised to take 6 capsules resulting in approx. 1000 mg EPA and 500 mg DHA per day.
- In addition, the capsules also contain mixed tocopherols and d-alpha-tocopherol (vitamin E) derived from soy oil.
- They also contain 3mg of orange oil.

8.1.2 Control Intervention

Treatment B (placebo)

- 418 mg medium chain triglycerides
- In addition, the capsules also contain mixed tocopherols and d-alpha-tocopherol (vitamin E) derived from soy oil.
- The capsules contain about 1.6mg of fish oil and 3mg of orange oil
- As in the active treatment, children < 13 years of age are advised to take 3 capsules and teenagers > 13 years of age are advised to take 6 capsules, respectively.

Burgerstein will produce specially manufactures child capsules as study medication that can easily be swallowed by children. Further information can be found in the Investigator's Brochure.

8.1.3 Packaging, Labelling and Supply (Re-Supply)

The Antistress AG will provide the clinical trials pharmacy of the canton of Zurich with active substance and placebo. The Cantonal Pharmacy will receive the randomization list from the CTC. Package, labelling and storage of study medication will be performed by the Cantonal Pharmacy according to the randomization procedure. Labelled study medication will then be shipped to the centres and study drug will be dispensed through the on-site person responsible for clinical trials.

An example of how the medication is labelled is below:

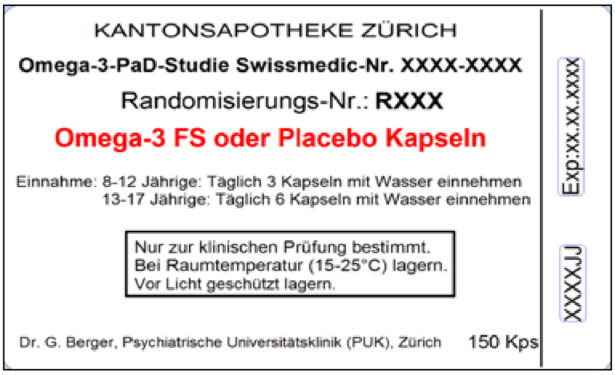

Below is the label for the placebo lead-in phase:

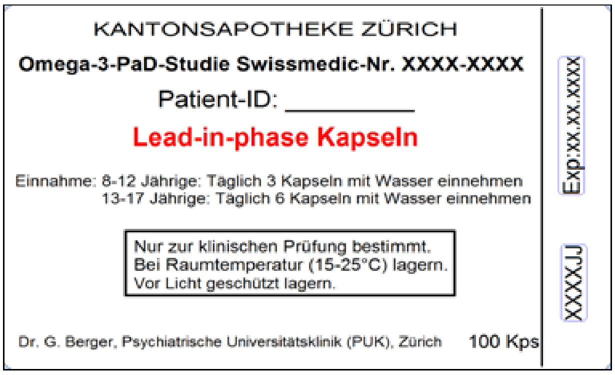

Labelled study medication containers will be dispensed for the whole study period (Clinical Trials unit, Zurich Cantonal Pharmacy) to the Child and Adolescent Psychiatric Service. Upon receipt of the study treatment supplies, an inventory must be performed and a drug receipt log filled out and signed by the person accepting the shipment. It is important that the designated study staff counts and verifies that the shipment contains all the items noted in the shipment inventory. Any damaged or unusable study drug in a given shipment will be documented in the study files.

The research psychologist will dispense the study drug at each study visit. Administration will be double-blinded throughout the trial period. Regular study drug reconciliation will be performed to

document drug assigned; drug consumed, and drug remaining. Patients will be asked to return study medication bottles and unused medication, and/or report non-compliance at each visit. The number of used/ returned capsules will be recorded. Missed doses and reasons for missed doses are entered in the eCRF and explained. The reconciliation will be logged on the drug accountability form, and signed and dated by the study team.

8.1.4 Storage Conditions

The gel capsules used in the clinical trial will be packed in dark brown glass bottles, which are closed with a pressure seal lid in order to maximize stability of the capsules as well as possible. The bottles will be packed in cardboard boxes to further protect the capsules from light. Each single sample including packaging is labelled. Samples are kept in a secure, limited access storage area under the recommended storage condition (see IB).

8.2 Administration of Experimental and Control interventions

8.2.1 Experimental Intervention

Patients above the age of 13 years will be advised to take 6 pills containing a daily dose of 1000mg EPA/ 500mg DHA. Patients under the age of 13 years old will be advised to take only 3 pills receiving only half the dose of the study medication. Patients will be advised to take the study medication once a day with their main meal as the bioavailability seems to be better after intake of large doses compared to smaller split doses (probably due to a favourable oxidation rate and better incorporation into membrane phospholipids [216]).
There will be no titration phase because of the good tolerability of the study medication. If a dose has been forgotten, patients will be advised to take the forgotten dose with the next meal or the next study dose. Compliance will be measured and controlled for (see below).

8.2.2 Control Intervention

As in the experimental intervention, patients over the age of 13 will be advised to take 6 pills while patients under the age of 13 will be advised to take 3 pills. The placebo capsules contain a small amount of fish oil to mimic the fishy taste. All the instructions are exactly the same as in the experimental condition.

8.3 Dose Modifications

Given that side effects of omega-3 fatty acids are usually mild, no dose modifications are permitted during the trial. If a patient cannot tolerate the study medication, he or she will be discontinued from the study.

8.4 Compliance with Study Intervention

The investigator will perform pill counts at each study research visit and patients will complete the MARS-D questionnaire (in <13 year olds, with parents support) -regularly, or as advised by the research psychologist. Red blood cell omega-3 fatty acids measurements will be used to monitor compliance, a commonly used method in previous trials [180]. On a study team level, regular meetings with the omega-3-pMDD study side teams will address site specific adherence issues (e.g. the postdoctoral study coordinator or one of the applicants will join a clinical meeting where most clinicians are present to address questions around the trial).
Non-compliance is defined as the discontinuation of taking the study medication for longer than three days out of seven days in two consecutive weeks, regardless of the circumstances, prior to completion of the trial. If a patient fulfills criteria of non-compliance, the participation will be terminated. The data will still be included into statistical analyses as the number of drop outs due to cessation/non-compliance is also an outcome measure between the groups. The reason for a subject discontinuing the study will be recorded in the eCRF.

8.5 Data Collection and Follow-up for Withdrawn Participants

If a patient drops out of the study, we will try to perform a drop out visit at the earliest convenient time. During the drop out visit, the primary reason for discontinuation will be determined by the investigator and recorded in the eCRF. Current medication, drug use and intake of food rich in omega-3 fatty acids will be recorded. Depressive symptoms will be assessed with the Children's Depression Rating Scale (CDRS-R) and other psychopathological variables will be assessed using a diagnostic interview (K-SADS-PL). Clinician will rate the general functioning of the patients using the Clinical Global Impression rating scale (CGI), the Children's Global Assessment of Functioning (CGAS), and the attrition checklist (HoNOSCA). Further psychopathological variables and symptoms will be assessed using a variety of self- and parent-rated questionnaires (Children's Depression Inventory, Beck Hopelessness Scale, Beck Anxiety Inventory, Suicidal ideation questionnaire, Insomnia Severity Index, Perceived Stress Scale, Connor-Davidson Resilience Scale, KIDscreen-CAT-27, Strength and Difficulty Questionnaire, IES-27). In case that no IQ testing was performed previously in the trial, a short IQ test (RIAS) will be administered. Potential side effects will be assessed with the Side Effect Self Report Scale (ASEC), and any AE will be determined and entered into the eCRF.

8.6 Trial Specific Preventive Measures

Patients will be monitored for the emergence of psychiatric illnesses other than major depression. Otherwise, patients will receive standard treatment for depression as advised by their clinician (see below). Female adolescents that are sexually active are advised to use proper contraceptive methods (the pill, coil). However, at the current state of research, omega-3 fatty acids have no teratogenic effects. We do not expect any impact on study objectives. Hormonal contraception may be associated with mood symptoms that will be monitored in the context of the trial. As this

is a RCT, study participants will be excluded from the trial in case of pregnancy. They will be followed up clinically to determine any unwanted or unknown side effects on the unborn child by the sponsor-investigator. The sponsor-investigator will liaise with the responsible obstetrician to guarantee that all potential unknown side effects of the study medication will be recorded and relevant authorities will be informed.

8.7 Concomitant Intervention(s)

The background treatment across both groups (active/ placebo) is standardized based upon the German S3 Guidelines for the treatment of depression in children and adolescents [34]. All participating sites will be trained accordingly. The core elements of the standardized background treatment will be based on the cognitive behavioural therapy (CBT) method including individual CBT as well as psycho-educational family sessions. The involvement of the school/ employer (in case the adolescent has already started working) is an integral part of the treatment.

8.7.1 Hospitalisation

The clinician together with the responsible consultant will review the clinical progress at each clinical visit. If no clinical improvement or even a worsening in psychopathology and functioning occurs despite regular psychological treatment according to the S3 Guidelines for the treatment of pMDD, the treating team together with the patient, parents and significant others should consider starting a more intensive treatment (e.g. hospital admission and/ or the addition of an antidepressant). This decision shall be made completely independent of the research team.

8.7.2 Starting an antidepressant

Starting an antidepressant is not a withdrawal criterion, but will be considered as an outcome measure in data analysis (e.g. we assume that the omega-3 fatty acids group will need less antidepressants compared to the placebo group). In addition, we will run an analysis where we will multiply impute the data since starting antidepressants, with the aim to mimic observations one would have observed without taking antidepressants. We will consider the missingness generating mechanism to be “not at random” (MNAR). As the number of patients and the time on antidepressants is a secondary outcome criteria, this decision shall be made completely independent of the research team. In order to have sufficient power for this analysis, we will recruit up to 20 additional patients who do not start with antidepressant treatment in the first 6 weeks of the trial.

8.7.3 Use of sedative medications (Benzodiazepine, antipsychotics)

The use of subtherapeutic doses of antipsychotic medication (e.g. 100-200mg quetiapine, or up to 2mg of risperidone) is permitted in case of medically required behavioural control (i.e. significant worsening of behavioural problems in an inpatient setting) at study entry, as well as during the study as long as it is not prescribed for one of the listed conditions under exclusion criteria.

Use of benzodiazepines is not limited or directed by the protocol. Subjects who start using or continue using PRN benzodiazepines (e.g. Temesta® for anxiety, or Stilnox® for sleeping) can

do so. Existing prescriptions of other psychiatric medication is evaluated at baseline and continued if clinically indicated (e.g. stimulant treatment of a longstanding ADHD). We will compare the duration and dose of concomitant (PRN) medication between active and placebo, a further secondary outcome measure.

All concomitant and/or rescue treatment(s) will be recorded in the eCRF.

8.8 Study Drug Accountability

Subjects will be asked to return any unused medication to the site. Accountability and subject adherence will be assessed by maintaining drug dispensing and return records. Adherence to the study medication will also be monitored by self-report, and the measurement of the omega-3 to omega-6 ratio in blood at screening and after six months will provide a measure of drug compliance (see above)
A Drug Accountability Log must be kept current and should contain the following information:

- the identification of the subject to whom the medication was dispensed
- the date[s], quantity of the medication dispensed to the subject
- the date[s] and quantity of the medication returned by the subject

This inventory must be available for inspection by the monitor. The shipping, receipt, returning and destruction will be tracked and documented in line with Good Clinical Practice (GCP) guidelines.

8.9 Return or Destruction of Study Drug

At the completion of the study, there will be a final reconciliation of drug shipped, drug consumed, and drug remaining. This reconciliation will be logged on the drug accountability form, signed and dated. Any discrepancies noted will be investigated, resolved, and documented prior to return or destruction of unused study drug. Drug destroyed on site will be documented in the study files.

9 STUDY ASSESSMENTS

Study assessments will be done according to the Study Flow Chart (9.1).

9.1 Study Flow Chart(s)/Table of Study Procedures and Assessments

Study visit			-1		1	2	3	4	5	
	Time (min)	Examiner	Screening/ And Lead in Phase		Baseline	Acute phase		Main-tenance phase		Drop-out
Weeks			7 to 14 days prior baseline		0	6 +/-3d	12 +/-3d	24 +/-5d	36 +/-5d	
Study information	15	RC	x							
Informed consent	5	RSP	x							
Inclusion / exclusion	5	R	x		x					
Sociodemographic data	1	R	x							
Medical incl. psychiatric history	1	R	x							
Family history	1	RSP	x							
Physical Health (incl. Tanner)	1	R	x						x	
Current medication use	1	R	x		x	x	x	x	x	x
Omega-3 Food questionnaire	5	S	x				x		x	x
WHO Assist 3.0 Drug Use	10	R	x				x		x	x
Childhood Trauma & Olweus victimization scale	5	S	x							
Intervention			Pla (7-10 d)		Active/Placebo					
Main outcome variables										
Children's Depression Rating Scale CDRS-R	15	R	x		x	x	x	x	x	x
Diagnostic interview (K-SADS-PL)	60	R	x		x*	x*	x*	x*	x	x
Clinician rated variables (at each clinical visit) +/- 2 weeks										
Clinical Global impression Scale CGI-S/I	1	C	x			x	x	x	x	x
Children Global Assessment Scale CGAS	1	C	x			x	x	x	x	x
Attrition check list inkl. HoNOSCA	8	C	x			x	x	x	x	x
Self-report variables (self rating for 13-<18yr, parent rating for 8-≤13yr)										
Children's Depression Inventory DIKJ	15	S			x	x	x	x	x	x
Beck Hopelessness Scale II BHS	5	S			x	x	x	x	x	x
Beck Anxiety inventory BAI II	5	S			x	x	x	x	x	x
Suicidal ideation questionnaire SIQ-Jr	5	S			x	x	x	x	x	x
The Insomnia Severity Index	1	S			x	x	x	x	x	x
Perceived Stress Scale PSS-10	5	S			x		x		x	x
Connor-Davidson Resilience Scale	10	S			x	x	x	x	x	x
KIDscreen-CAT-27	15	SP	x(P)		x		x		x	x
SDQ	5	SP	x(P)		x		x		x	x
IES-27-J	15	SP	x(P)		x		x		x	x
ASEC (AD side effect self report scale)	5	S			x	x	x	x	x	x
Cognitive outcome variables										
BRIEF (executive Functioning questionnaire)	10	SP	x(S,P)				x		x	
Cognitive battery	50	R	x				x		x	
RIAS (short IQ test for matching)	20	R				x				x
Biological outcome variables										
Clinical bloods, hsCRP (2-3d prior randomization)	5	N	x						x	x
Red blood cells (PUFAs), Serum (e.g. immune multiplex marker chip), Buffy coat for tissue repository (e.g. genetic, epigenetic markers)		N	x				x		x	x
Urine (drug screen, F2 isoprostane)	5	N	x				x		x	x
Saliva cortisol	1	N	x				x		x	x
Hair	1	R				x			x	
Other measures										
Drug dispensing/ Pill count/appointment	1	R	x		x	x	x	x		
AE					x	x	x	x	x	x
MARS-D	3	S			x	x	x	x	x	
Approx. time requirement for patient / family only (min)			180	90	90	90	210	120	210	200

* Only subscale 'depressive disorders' will be performed

R = researcher; C = clinician; S = self-rated; P = parent-rated; N = study nurse

9.2 Assessments of Outcomes

9.2.1 Assessment of Primary Outcome

- The Children's Depression Rating Scale-revisedTM CDRS-R is the most widely used rating scale for assessing severity of depression and change in depressive symptoms for clinical research trials in children and adolescents. The CDRS-R quantifies childhood [217] and adolescent [218, 219] depressive symptoms and is evaluated in German [218]. The CDRS-R is a 17-item scale with items ranging from 1 to 5 or 1 to 7 (possible total score from 17 to 113), that will be rated by the research interviewer via interviews with the child and parent/ guardian. A score of ≥40 is indicative of moderate to severe depression (35-40 for mild depression), whereas a score ≤28 is often used to define remission (minimal or no symptoms). The CDRS-R will be completed at each research visit. The time requirement is about 15 minutes.
- The Kiddie - Schedule for Affective Disorders and Schizophrenia - Present and Lifetime Version K-SADS-PL [220] is an interviewer-based diagnostic interview designed to assess current and past DSM-IV diagnoses in children and adolescents, by interviewing the parent(s) and child. The section for diagnosing the presence of a current major depressive disorder will confirm the presence of a MDD at baseline, and the recovery from a depressive episode in conjunction with the CDRS-R total score <28. Lifetime assessments will be completed at baseline and week 36, also to exclude the emergence of another major psychiatric disorder. At month three and six, the mood section of the K-SADS-P will be done to determine the recovery status of a study participant.

To increase inter-rater reliability the research coordinator and research interviewers will be trained in the use of all applied instruments. Inter-rater reliability sessions will be done. All researchers will also attend a GCP course.

9.2.2 Assessment of Secondary Outcomes

9.2.2.1 Clinician-Rated secondary outcome measures

- The Clinical Global Impression rating scales CGI [221] is three-item observer-rated scale to measure symptom severity, global improvement/ change and therapeutic response. The Clinical Global Impression – Severity scale (CGI-S) is a 7-point scale that requires the clinician to rate the overall severity. The Clinical Global Impression (CGI-I) is a 7 point scale that requires the clinician to assess how much the patient's illness has improved or worsened relative to a baseline state (1, very much improved; 2, much improved; 3, minimally improved; 4, no change; 5, minimally worse; 6, much worse; or 7, very much worse). The Impression – Efficacy Index is a 4×4 point index that results out of the therapeutic effect versus side effects.

- The Children's Global Assessment of Functioning CGAS [222] is a numeric scale (1 through 100) used by mental health clinicians to rate the general functioning of children under the age of 18.
- The Health of the Nation Outcome Scales for Children and Adolescents – HoNOSCA [223] is a mandatory quality and outcome measure for clinicians working in child- and adolescent psychiatric services in Switzerland (many other countries have also implemented this short and comprehensive outcome scale for service evaluation). The HoNOSCA scores the behaviour, impairments, symptoms and social functioning of children and teenagers with mental health problems using 13 global items. The severity of each problem is scored on a scale of 0-4. Clinician will rate the HoNOSCA together with the CGI and CGAS and the attrition checklist requiring about 10 minutes.

All three scales will be administered at baseline and at each clinical visit. Given that these assessments depend on the clinicians' appointments with the patients, the time frame for these particular scales is extended to ± 2 weeks.

9.2.2.2 Self- and parent-rated secondary outcome measures

- The Children's Depression Inventory DIKJ [224] is a 26-item scale used to assess depressive symptoms in children and adolescents and has been evaluated in German. It is derived from the BDI but modifies some questions to be more appropriate for younger ages. The DIKJ asks about key symptoms of depression, such as a child's feelings of worthlessness and loss of interest in activities. Each item allows the patient to respond to 3 choices that indicate 3 levels of symptoms: 0 (absence of symptoms), 1 (mild or probable symptoms), or 2 (definite symptoms). The DIKJ can be used with patients who are aged 8 to <18years, and usually takes about 15 to 20 minutes to complete.
- The Beck Anxiety Inventory BAI [225] is a 21-question self-report inventory that is used for measuring the severity of anxiety in children and adults. The questions used in this measure ask about common symptoms of anxiety of the past month (such as numbness and tingling, sweating not due to heat, and fear of the worst happening). It is designed for individuals who are of 7 years of age or older and takes 5 to 10 minutes to complete. Several studies have found the Beck Anxiety Inventory to be an accurate measure of anxiety symptoms in children and adults. Scoring the BAI is based on a 0-3 point scale (0: "not at all", 3 severe: "It bothered me a lot"). The BAI has a maximum score of 63.
- The Beck Hopelessness Scale BHS [226, 227] is a 20-item self-report inventory that measures three major aspects of hopelessness: feelings about the future, loss of motivation, and expectations. The test was designed for adults, but is also used in adolescents [228, 229]. It measures the extent of the respondent's negative attitudes, or pessimism about the future. It has been used as an indicator of suicidal risk in depressed people who have made suicide attempts. The scale will only be used in adolescents aged 13 to <18 years.
- The Suicide Ideation Questionnaire-Junior SIQ-Jr [230-232] is a 15-item version for 12-15 year old teenagers to assess suicidal risk, but can also be used with younger children. The 15-item questionnaire takes 5-10 minutes to complete.
- The Insomnia Severity Index [233, 234] consists of 7 items assessing subjective sleep quality and is completed within 1-2min.

- The Cohen's Perceived Stress Scale [235] is a very widely used 10-item self-report questionnaire to quantify the perception of stress (see also http://www.psy.cmu.edu/~scohen/scales.html). The PSS scale has a 5-point scale and has been translated und used in German (Prof. Dr. Arndt Büssing, University of Witten/Herdecke). It takes less than five minutes to complete.
- The 25-item Connor-Davidson Resilience Scale CD-RISC [236, 237] is a well validated instrument that assesses the stress coping ability and, as such. The CD-RISC comprises of 25 items, each rated on a 5-point scale (0–4), with higher scores reflecting greater resilience and can be administered in 5-10 minutes. Resilience is an important factor in depression outcome, as it may explain some of the variance in the illness course. The CDRISC has sound psychometric properties and distinguishes between those with greater and lesser resilience. The scale demonstrates that resilience is modifiable and can improve with treatment, with greater improvement corresponding to higher levels of global improvement. It has been translated and used in German [238].
- The KIDscreen-CAT-27 [239] is a widely used self-report questionnaire to measure quality of life in five dimensions in children and adolescents that has also been used in large-scale German studies. It can be completed in 10 - 15 minutes depending on reading capacity. A self-rated and a parent-rated version will be used.
- Der Jugend- & Elternfragebogen über impulsives Verhalten und Erleben von Kindern und Jugendlichen – IES-27-J&E [240] is a German questionnaire especially developed to measure impulsivity and emotionality in Borderline Personality Disorders. A currently unpublished study (provided to us by Priv.- Doz. Dr. Christoph Kröger, Humboldtstraße 33, D-38106 Braunschweig) was able to discriminate between impulsivity in the context of BPD and externalizing behaviour in minors. The assessment of Borderline features is of importance as several studies could demonstrate that omega-3 fatty acids exert some positive effects on Borderline-like behaviours [142]. Teenagers > 13 years of age will fill in the questionnaire themselves, while the parents will complete a parent-rated version for children < 13 years of age.
- The Strength and Difficulty Questionnaire SDQ is a brief behavioural screening questionnaire, consisting of 25 items asking about difficulties (emotional, conduct, hyperactivity, relationship problems) and strengths (prosocial behaviour). A self-rated (> 11 years of age) and a parent-rated version (< 11 years of age) are available. It takes about 5 min to complete.

9.2.2.3 Cognitive outcome measures

We have selected following battery of neuropsychological tests to capture the key deficits associated with depression at baseline, week 12 and week 36 after study entry: Verbal Memory:

- Verbal Memory Learning Test VMLT (with parallel versions for retests) [241] (20 min)
- Verbal fluency RWFT [242] (5 Min.)
- Inhibition/Flexibility: Shifting Attentional Visual Set (ANT) [243] (3 parts, a total of 12-15 min)
- Emotion Recognition: Identifying Facial Emotions (ANT) [243] (8-10 Min)
- Verbal Working memory: Digit Span forward and backward (WISC IV) [244] (5 min).

- Problem solving skills: Block design (WISC IV): only at baseline

The BRIEF Cognition will be assessed at baseline, week 12 and week 36 after study entry [245]. Total duration of cognitive testing is approximately 50-55 minutes.
A short IQ test (for group matching) will be conducted only once, at week 6(RIAS, 20-25 min) [246].

9.2.2.4 Biological outcome measures

Blood sampling procedures will be described in a Standard Operating Procedure ‘Blood Sampling’ (jointly developed by Prof. Dr. E. Grünblatt and Prof. Dr. M. Hersberger together with the main applicant). Blood samples will be drawn from pMDD subjects after enrolment, at week12 and week36 to assess routine laboratory parameters.

- Haemoglobin
- Haematocrit
- Leucocytes
- Thrombocytes
- Thyroid (TSH only)
- Liver function test (ALAT, ASAT)
- hsCRP
- Ferritin

The remaining blood will be stored for PUFAs (omega-3, 6 and 9 and trans fatty acids), bioactive lipids (e.g. E-series resolvins), immune parameters (including but not restricted to interferon-γ, interleukin (IL)-1α, IL-1RA, IL-5, Il-6, IL-10, IL12p40, IL-15, IL-18 and tumour necrosis factor-α, as well as leptin and adiponetin), and for the tissue repository. In addition, 2.7ml blood will be drawn in order to assess mitochondrial metabolism.
Except for hsCRP (a stratification variable) all markers of prediction will be measured at the end of trial after completion of the final visit of the last patient (to prevent unblinding), except of the baseline data that might be used in the context of planed add-on projects that will be submitted as amendments (e.g. sleep project, imaging project). Altered levels of these parameters have been shown in MDD and other disorders, and may predict both the course of the illness and how levels of bioactive lipids, and immune parameters are related to both: a.) if these blood parameters are able to serve as potential biomarkers that may predict clinical outcome and b.) if these blood parameters are associated with psychopathology and illness course.
Since multiple studies are currently investigating biomarkers in MDD, serum, lymphocytes, erythrocyte, platelets and DNA will be stored after the end of the study to measure potential novel biomarkers. Genetic and epigenetic markers of interest (e.g. FADS haplotypes) include but are not restricted to genes relevant for bioactive lipid metabolism. The establishment of the genetic tissue repository is part of the project but will be financed separately, as this is not core to the proposed IICT. Ethics approval for the generation of the tissue repository has been granted (Applicant Prof. S. Walitza, BASEC-Nr. 2016-00101).
Furthermore, we will also analyze the iron status, as previous studies have shown an interaction between iron status and omega-3 fatty acids. For the correct interpretation of the iron status, we will ask all patients to provide us with information about iron and other vitamin supplementation they might have taken before study start. Patients who have already been enrolled in the trial will be recontacted and asked to provide the missing information.

The aim is to collect all blood samples for all subjects. However if subjects do not want to have subsequent blood samples drawn or urine and saliva collected, they can still participate in the study. In a separate consent form, subjects will be asked to provide blood for BioBanking in accordance with the Swiss legal regulations. Over the course of the entire study, a total amount of approximately 50 ml blood will be maximally drawn: After signing the consent from 3x3ml to perform standard clinical laboratory tests, 4 ml for bioactive lipids, 10 ml for (epi)genetic markers, 10 ml for immune markers, 10ml for specialized pro-resolving lipid mediators (SPM), and 3ml for mitochondrial metabolism will be drawn. The laboratory blood sampling must be done for all subjects after written informed consent has been obtained as this information is required for the minimization procedure (stratification). The further bloods shall be taken if possible, but are not mandatory for participation in the clinical trial. If too many subjects refuse the additional blood samples, total sample size may need to be reconsidered to be able to address secondary and tertiary outcome measures.
In addition, patients will provide a urine sample for later drug screening and F2 isoprostane, and a saliva sample to measure cortisol concentrations.
In addition, cortisol and other hormones will also be measured with a string of hair, which will be sampled at 6 weeks and at study end on condition of the patient's consent, to allow a continuous stereoid profile over the whole study period. In addition, a short hair protocol will be filled in. If patients provide consent for storage of material in the BioBank, the remaining hair will be stored in the BioBank for potential future analysis.

9.2.3 Assessment of Other Outcomes of Interest

- The Childhood Trauma Questionnaire – short version CTQ-SV is a 25-item self-report scale that aims to detect experiences of childhood abuse and neglect in adults and adolescents. The Olweus Bully/Victim Questionnaire (BVQ) is a questionnaire which assesses bullying behaviour and victimization in children and adolescents, and can be filled in online. While the victimization part will be administered for all patients, the bullying part is optional. Patients who are already participating in the trial will be asked whether they agree to additionally fill in the two questionnaires, irrespective on how long they already have been participating in the trial.
- Tanner Criteria [247] Self-report form on pubertal status. The assessment of the pubertal status will enable us to investigate if the developmental stage has an impact on omega-3 fatty acids effects. If the children are > 13 years of age, parents are asked to fill in the questionnaire due to the explicit nature of the pictures.
- The Omega-3 Food Frequency Questionnaire n-3 FFQ [248] is a 21-item self-report that was developed using the National Cancer Institute's Diet History Questionnaire as a model. The scale was adapted to reflect local fish intake and was developed in conjunction with Prof. Herter of the ETH. The n-3 FFQ takes 10 minutes to complete, and assesses the average n-3 intake over the last 6 months. Items in the n-3 FFQ included an extensive list of specific seafood and fish available in Switzerland, as well as walnuts, flaxseed, flaxseed oil, cod liver oil and canola oil. The questionnaire also includes specific questions about type and dosage of n-3 PUFA dietary supplements (individuals taking supplements will be excluded).

- The WHO Assist 3.0 [249] is a widely used structured Interview to quantify substance and drug abuse that will be completed by the research interviewer enabling us to control for substance misuse.
- The Antidepressant Side Effect Checklist (ASEC) [250] is a 21-item self-report scale quantifying the most commonly described side-effects of antidepressants. It has a 0-3 scoring (0 = absent, 1 = mild, 2 = moderate, 3 = severe) and asks the patients if he believes that there is a link with the study medication for each item.
- The Medication Adherence Reporting Scale-Deutsch (MARS-D) is the German Version of a self-report adherence reporting scale that has been developed by K. Thompson at ORYGEN Youth Health [251], a specialized clinic for young people with emerging mental disorders, but is now used in a broader sense [252]. The scale includes 5 items. In the initial validation study, an internal consistency reliability of α = 0.75 was found. MARS examines adherence behaviours and attitudes toward medication. We choose MARS as it has been used in young people with emerging mental illness and has been evaluated in German.

9.2.4 Assessment of Safety Outcomes

9.2.4.1 Adverse Events

The investigator controls weekly if any AE or SAE has occurred. If so, the investigator contacts the clinician to get informed about the circumstances which led to the AE, and together with the principal investigator the connection between the AE and the study drug is clarified. If a connection has been established, the SUSAR will be reported according to the principles stated in Section 10. Furthermore, at each study visit, the Antidepressiva side effect Self Report Scale (ASEC) will be administered. In case the patient reports any abnormalities, the investigator will inform the clinician and principal investigators to determine whether an AE or SAE occurred. A detailed description of collaboration and responsibilities between researchers and clinicians can be found in the SOP (collaboration between research and clinic).

9.2.4.2 Laboratory Parameters

At screening and at nine months (or the drop out visit), blood will be drawn for standard clinical laboratory tests. Clinical blood parameters (haemoglobin, haematocrit, leucocytes, thrombocytes, TSH (thyroid function test), ALAT & ASAT (liver function test), and thromboplastin (blood coagulation) will be assessed in order to screen the patient for abnormal blood parameters. These analyses will be done in the Children's hospital of Zurich. The results will be entered into the eCRF as they are part of the inclusion criteria and will also provide the stratification parameter (hSRF). All the other blood samples will be stored for analyses as outlined in the protocol.

9.2.4.3 Vital Signs

Vital signs will be assessed in the lead-in phase and again at the end of the study (week36) by the study nurse or investigator (e.g. heartbeat, blood pressure, body temperature, height and weight).

A drop-out visit shall be performed including documentation of reasons for drop out in case a patient drops out, or a parent / clinician / investigator withdraws a patient from the study. The drop-out visit will be performed at the earliest convenient time and will include an assessment of physical health and current medication use. Depressive symptoms will be assessed using the CDRS-R and the K-SADS-PL. In addition, psychosocial functioning will be assessed with a range of clinician rated scales (CGI-S/I, CGAS, HoNOSCA) and self-reported scales (DIKJ, BHS, BAI II, SIQ-Jr, PSS-10, Connor-Davidson Resilience Scale, SDQ, Insomnia Severity Index, IES-27-J). Patients will fill in the ASEC for assessing putative side effects. If the patient agrees, blood, urine, saliva and hair will be sampled, and in case no IQ test was done previously during the trial, a short IQ test will be performed.

9.3 Procedures at Each Visit

9.3.1 Screening and lead-in phase

During the first meeting with the investigator or psychologist, the study will be explained to the subject including possible benefits and disadvantages of participation. When the patient's willingness is assured and written informed consent is provided, the subject will be screened for in- and exclusion criteria. Information about sociodemographic, medical and family history, current medication use, psychiatric history, drug use, and childhood trauma will be collected. Current and past episodes of psychopathology will be determined using the semi-structured diagnostic interview Schedule for Affective Disorders and Schizophrenia for School Aged Children (K-SADS-PL) in cases where no K-SADS-PL interview has been done in the last 4 weeks. If a K-SADS-PL interview has already been performed in the last 4 weeks in the context of standard clinical praxis, the K-SADS-PL shall not be repeated given the extensive nature of the full interview. In that case, the results should be transferred from the clinical records into the study database. The Children's Depression Rating Scale (CDRS-R) is used to determine severity of depressive symptoms.
The K-SADS and the CDRS are both completed once with the patient and once with a parent / caregiver. At the time of the appointment with the parents, also the other parent-rated questionnaires (PTBS Checklist, BRIEF-E, SDQ-E, KIDscreen-Cat-E,IES27-E) shall be administered so that only one appointment in the first week with the parent is necessary. Alternatively, these questionnaires can also be filled in online (via a unique SecuTrial access code) by the parents any time prior baseline. The interview with the patient may be at the same time or at a different time, depending on the convenience and age of the patient. Research assessments may be carried out either at the participating centres or at the patients' home, as wished by the patients and their families.
Thereafter the patient enters the single-blind, placebo lead-in period (7 to 10 days), The capsules may be given to the patient any time before baseline but the duration of the intake of the placebo lead in capsules should not exceed 12 days. The lead in phase is single blind, meaning that the patient and parents do not know that the participants takes placebo capsules during the lead in phase. The research interviewer will also schedule further appointments prior to baseline to sample biological variables and further assess psychosocial and cognitive functioning. Similar to the questionnaires and the cognitive testing, biological sampling can take place any time before baseline but not thereafter.

To minimise the burden for participants, blood to assess levels of bioactive lipids, (epi)genetic markers and immune parameters will be collected simultaneously. In addition, a physical examination will be conducted where heart rate, blood pressure, weight, height and body temperature will be measured. Cognitive testing will be performed anytime during the lead-in phase but before baseline and a short questionnaire regarding executive functioning (BRIEF) will be filled in. The current dietary intake of Omega-3 fatty acids will be assessed with the FFQ. An appointment for the baseline visit will be scheduled.

9.3.2 Visit 1: baseline visit

The baseline visit takes place at the end of the placebo-lead-in phase. First, depressive symptoms will be assessed with the CDRS-R and the subscale depressive disorders of the K-SADS-PL. Parent’s ratings can be obtained in person or over the phone. If the patient still fulfils the diagnosis of a major depressive disorder with at least moderate severity, the patient is enrolled using the computerized 1:1 randomization procedure described above. Current medication use, AEs and the Medication Adherence Reporting Scale is assessed. Clinician rate the patient according to the CGI, CGAS, and the HoNOSCA. Depressive symptoms will be further assessed with the Children’s Depression Inventory (DIKJ), the Beck Hopelessness Scale II (BHS), the Beck Anxiety Inventory (BAI II), the suicidal ideation questionnaire and the Insomnia Severity Index. Further self-reported questionnaires are filled in by the patients including the Perceived Stress Scale (PSS-10), the Connor-Davidson Resilience Scale, the KIDscreen-Cat-27, the Strength and Difficulty Questionnaire (SDQ), and the IES-27-J. In patients > 13 years of age, self-rating questionnaires shall be completed with help of the parents or the research investigators. Patients > 13 years shall complete the questionnaires themselves (parents are instructed not to influence the patent’s self-ratings). Self-rating questionnaires may be directly entered online into the data base by the patients / families themselves. Perceived side effects will be assessed with the Antidepressant Side Effect Checklist (ASEC). At the end of the baseline visit, study medication will be dispensed for the first time and new appointments will be scheduled.

9.3.3 Visit 2: 6 weeks

After 6 weeks a brief follow-up assessment will be done. During this visit, information about current medication use, adverse events and possible side effects (ASEC) will be collected. In addition, certain measures of symptomatology and psychosocial functioning that are performed at baseline will be repeated (CDRS-R, affective disorder subscale of the K-SADS-PL, DIKJ, BHS, BAI II, SIQ-Jr, Insomnia Severity Index, Connor-Davidson Resilience Scale). Clinician will rate the patients’ functioning on the CGI and CGAS, and will fill in the attrition checklist. Patients and their parent/ guardian will return study medication at each research visit for pill counts and complete a Medication Adherence Rating Scale (MARS German version). In addition, a short IQ test (RIAS) will be performed. If the patient previously agreed on the consent form that hair can be sampled, then a string of hair will be collected and a short hair protocol will be filled in. New study medication will be dispensed and a further appointment scheduled.

9.3.4 Visit 3: 12 weeks

At the third visit (week 12) a longer assessment will be held. In addition to the psychological rating scales which are repeated as in the baseline condition, also cognitive testing will be performed and biological parameters will be sampled (blood, urine and saliva). The study nurse will go and see the patients either at home, or at the clinic to take blood and urine. Patients and their parents will be given a saliva kit with the instruction to sample saliva in the morning after waking up. New study medication will be dispensed and a further appointment scheduled.

9.3.5 Visit 4: 24 weeks

The fourth visit the primary outcome measures will be assessed and different questionnaires will be filled in according to the study plan.

9.3.6 Visit 5: 36 weeks

This will be the last visit of the trial. and a more extensive l research assessment will be held. All scales already assessed at baseline will be repeated, cognitive testing will be performed, and blood, urine, saliva, and hair will be sampled, and non-used pills will be counted.

9.3.7 Clinical Care and monitoring of clinical visits

Usually clinicians will see outpatients on a weekly basis for the first six weeks, and gradually increase the time lap between visits (e.g. on a two weekly bases between week 6-12), and thereafter as clinically indicated. However, clinicians are allowed to see patients whenever clinically indicated. Clinicians will perform clinician-based assessments (CGI, CGAS, and HoNOSCA) and complete the attrition checklist that includes the documentation of service use and adverse events at any clinical visit.

10 SAFETY

The Sponsor's SOPs provide more detail on safety reporting.

During the entire duration of the study, all adverse events (AE) and all serious adverse events (SAEs) are collected, fully investigated and documented in source documents and case report forms (eCRF). Study duration encompassed the time from when the patient signs the informed consent until the last protocol-specific procedure has been completed, including a safety follow-up period.

10.1 Definition of (Serious) Adverse Events and Other Safety Related Events

Adverse events

Adverse events (AEs) are defined as any untoward medical occurrence in a patient or clinical investigation participant administered a pharmaceutical product and which does not necessarily have a causal relationship with this treatment. An AE can therefore be any unfavourable and unintended sign (including an abnormal laboratory finding), symptom, or disease temporally associated with the use of a medicinal study product, whether or not related to the medicinal study product.

An AE may also consist of a new disease, an exacerbation of a pre-existing illness or condition, a recurrence of an intermittent illness or condition, a set of related signs or symptoms, or a single sign or symptom.

AEs observed by the investigator and/or reported by the participant must be reported in the eCRF during the entire study period, i.e. the period of time from the first (= signature of informed consent) to the last protocol-specific procedure regardless of the medicinal study product relation assessment.

For all AEs, sufficient information will be pursued and/or obtained so as to permit an adequate determination of the outcome of the event (i.e., whether the event should be classified as an SAE) and an assessment of the causal relationship between the AE and the investigational drug or study treatment(s).

Whenever available, the underlying disease or condition for which a therapeutic or diagnostic procedure is required should be reported as the AE term. Surgeries or other invasive procedures that had already been planned prior to the start of the study do not have to be documented as AEs. These planned procedures will be recorded in the eCRF by the investigator at the baseline visit. It is not important if the condition was known before enrolment, only if the procedure was planned before.

Serious Adverse Event

An SAE is any untoward medical occurrence that at any dose results in

- death,
- is life-threatening,
- requires participant hospitalization or prolongation of current hospitalization,
- results in persistent or significant disability/incapacity, or
- is a congenital anomaly/birth defect,
- any important medical event and any event which, though not included in the above, may jeopardise the participant or may require intervention to prevent one of the outcomes listed above.

Any other medically important condition that may be not immediately life-threatening or results in death or hospitalization but may jeopardize the participant or may require intervention to prevent one of the outcomes listed above should also usually (i.e. based on medical and scientific judgment) be considered serious. For example: intensive treatment at home for allergic bronchospasm; certain laboratory abnormalities (e.g. blood dyscrasias); convulsions that do not result in hospitalisation; development of drug dependency.

SAEs should be followed until resolution or stabilisation. Participants with ongoing SAEs at study termination (including safety visit) will be further followed up until recovery or until stabilisation of the disease after termination.

Unexpected Adverse Drug Reaction

An "unexpected" adverse drug reaction is an adverse reaction, the nature or severity of which is not consistent with the applicable product information (e.g. Investigator's Brochure for drugs that are not yet approved and Product Information for approved drugs, respectively).

Suspected unexpected serious adverse reaction (SUSAR)

A serious adverse reaction, the nature or severity of which is suspected to be not consistent with the applicable product information as stated in the Investigator's Brochure.

Safety Signals

All suspected new risks and relevant new aspects of known adverse reactions that require safety-related measures.

10.2 Recording of (Serious) Adverse Events and Other Safety Related Events

Clinical investigators and ultimately the protocol Principal Investigator (PI) have the primary responsibility for AE identification, documentation, grading, and assignment of attribution to the investigational agent/intervention.

Clinical study participants will be routinely questioned about AEs at study visits. The well-being of the participants will be ascertained by neutral questioning ("How are you?"). The investigator is responsible for reporting all AEs occurring during the course of the study. In case of any reported (S)AE, the research interviewer will inform the clinician and the GCP-certified study doctor (Prüfarzt) who will then classify the AE according to the criterion noted below.

All observed or volunteered adverse drug events (serious or non-serious) and abnormal test findings, regardless of treatment group or suspected causal relationship to the investigational drug or study treatment(s) will be recorded in the patient file and subsequently in the eCRF.

AEs or abnormal test findings felt to be associated with the study treatment(s) will be followed until the event (or its sequelae) or the abnormal test finding resolves or stabilizes at a level acceptable to the investigator.

An abnormal test finding will be classified as an AE if one or more of the following criteria are met:

- The test finding is accompanied by clinical symptoms.
- The test finding necessitates additional diagnostic evaluation(s) or medical/surgical intervention; including significant additional concomitant drug treatment or other therapy
  Note: simply repeating a test finding, in the absence of any of the other listed criteria, does not constitute an AE.
- The test finding leads to a change in study dosing or discontinuation of participant participation in the clinical study.

All AEs, serious and non-serious, will be fully documented in the appropriate eCRF. For each AE, the investigator will provide the onset, duration, intensity, treatment required, outcome and action taken with the investigational product.

10.3 Assessment of (Serious) Adverse Events and Other Safety Related Events

The investigator will promptly review documented AEs and abnormal test findings to determine if
- the abnormal test finding should be classified as an AE,
- if there is a reasonable possibility that the AE was caused by the investigational drug or study treatment(s), and
- if the AE meets the criteria for an SAE.

The intensity of an AE will be assessed by the investigator as being
- mild (hardly noticeable, negligible impairment of well-being),
- moderate (marked discomfort, but tolerable without immediate relief), or
- severe (overwhelming discomfort, calling for immediate relief).

The assessment of causality to the study drug by the investigator is done according to the following definitions:

Unrelated	• The event started in no temporal relationship to medicinal product applied and<br>• The event can be definitely explained by underlying diseases or other situations.
Related	• The event started in a plausible temporal relationship to medicinal product applied and<br>• The event cannot be definitely explained by underlying diseases or other situations.

10.4 Reporting of Serious Adverse Events and Other Safety Related Events

The principal investigator is responsible for reporting of any SAEs to the Sponsor immediately, i.e. within 24 hours.
The Investigator is responsible for SAE reporting to the CEC according to the following details:
- Reporting to CEC any SAE which resulted in death:

  - **without delay**, and no later than **7 calendar days**.
- Reporting to CEC of fatal SAEs if evaluated as “suspected”, “unexpected” and “drug related” (SUSAR)

  - **without delay** and no later than **7 calendar days** following awareness that event meets criteria for an SUSAR.

- Reporting to CEC of non-fatal SAEs if evaluated as "suspected", "unexpected" and "drug related" (SUSAR):
  - **promptly** and no later than **15 calendar days** following awareness that event meets criteria for a SUSAR.
- All other SAEs will be summed up in the **annual safety update report**.

The Sponsor is responsible for SAE reporting to Swissmedic according to the following details:

- Compliance with the regulatory requirements of Swissmedic regarding prompt reporting of unexpected SAEs for which a causal relationship with the study drug cannot be ruled out.
- Reporting to Swissmedic of fatal SAEs if evaluated as "suspected", "unexpected" and "drug related" (SUSAR):
  - **without delay** and no later than **7 calendar days** following awareness that event meets criteria for a SUSAR;
- Reporting to Swissmedic of non-fatal SAEs if evaluated as "suspected", "unexpected" and "drug related" (SUSARs):
  - **promptly** and no later than **15 calendar days** following awareness that event meets criteria for a SUSAR.
- Sending Annual Safety Reports (ASR), starting one year after the date of notification to Swissmedic. These reports should contain:
  - A concise critical summary of the safety profile of the drug studied as well as the safety issues that have arisen;
  - A listing of all SUSARs that have occurred in Switzerland and at international level (if applicable);
  - Ideally all adverse drug reactions at international level.
  - The accompanying letter provided with the Annual Safety Report should contain a short summary of the status of the clinical trial in Switzerland (number of centres open/closed, number of patients recruited/recruitment closed, and number of SAR/SUSAR.

A list of all SAEs and SUSARs will be generated annually. The annual safety report contain information from all sites. The summery table will be composed by the central study team, signed by the applicant and co-applicant and submitted to the participating investigators, the IDMC and to the appropriate authorities. The participating investigators submit it to the local committees. The summary table will be arranged by organ systems. A review of all new scientific data as well as new safety signals will be included. If possible (if timing of the meeting and Annual Safety and Progress Report coincide), a cost-benefit evaluation performed by the Independent Data Monitoring Committee is included, based on a complete safety analysis and an evaluation of the balance between the efficacy and the harmfulness of the medicine under investigation.

<u>Reporting of Safety Signals</u>

All suspected new risks and relevant new aspects of known adverse reactions that require safety-related measures, i.e. so called safety signals, must be reported to the Sponsor-Investigator within 24 hours. The Investigator must report the safety signals within 7 days to the local Ethics Committee (local event via local Investigator) and the Sponsor to Swissmedic, respectively. The Sponsor-Investigator must immediately inform all participating Investigators about all safety signals. The other in the trial involved Ethics Committees will be informed about safety signals via the Sponsor-Investigator.

Reporting and Handling of Pregnancies

Pregnancy per se does not classify as an AE. However, AEs related to a pregnancy have to be reported like any other AEs. Pregnancy should be confirmed by a reliable laboratory test. Pregnant participants must be immediately withdrawn from the clinical study. All pregnancies occurring during the treatment phase of the study and within 30 days after discontinuation of study medication have to be reported to the Sponsor-Investigator within 24 hours of the investigational sites knowledge of the pregnancy and recorded on the eCRF. The Sponsor-Investigator will contact the attendant physician by phone during pregnancy and after the estimated date of delivery to enquire about course and outcome of the pregnancy. Course of the pregnancy and health status of the new born child have to be documented on the Follow-Up Pregnancy Report Form.

10.5 Follow up of (Serious) Adverse Events

Participants terminating the study (either regularly or prematurely) with

- reported ongoing SAE, or
- any ongoing AEs of laboratory values or of vital signs being beyond the alert limit

will return for a follow-up investigation. This visit will take place up to 30 days after terminating the treatment period. Follow-up information on the outcome will be recorded on the respective AE page in the eCRF. All other information has to be documented in the source documents. Source data has to be available upon request.

In case of participants lost to follow-up, efforts should be made and documented to contact the participant to encourage him/her to continue study participation as scheduled. In case of minor AEs a telephone call to the participants may be acceptable.

All new SAE or pregnancies that the investigators will be notified of within 30 days after discontinuation of study medication have to be reported in appropriate report forms and in the eCRF if required.

Follow-up investigations may also be necessary according to the investigator’s medical judgment even if the participant has no AE at the end of the study. However, information related to these investigations does not have to be documented in the eCRF but must be noted in the source documents.

11 STATISTICAL METHODS

Descriptive statistics will be provided for all data broken down by medication (omga-3 fatty acids and placebo), group (patients who recovered from the depression, patients who did not recover) and visit. Mean, median, standard deviation, range and number of observations will describe continuous variables. Frequencies and percentages will describe discrete variables. All statistical tests will be carried out two-tailed; the alpha (level of significance) is 5%. Efficacy of the treatment will be assessed using a linear random coefficient regression model.

11.1 Hypothesis

The Null Hypothesis is defined as no difference in response rates between the two treatment groups while the Alternative Hypothesis states that the two groups differ at least 20% in response rates. If the treatment group shows an increase in response rates of at least 20%, the main study objective will be met.

11.2 Determination of Sample Size

Sample size estimation was done by Prof. Burkhardt Seifert from the Epidemiology, Biostatistics and Prevention Institute (EBPI), based on the assumption that we will perform a continuous outcome trial using a 1:1 randomization parallel-group design. Meta-analyses selecting studies of omega-3 fatty acids with high proportion of EPA in aMDD as primary diagnosis found an SMD between 0.28-0.56 [160-166]. However, no studies were performed in pMDD, except of one very small (n=20) pilot study in childhood depression with a large effect size (SMD=1.2) [167].
We performed a sample size calculation using nQuery Advisor 7.0. Assuming the optimistic effect size of 0.54 using a two-group t-test for the CRSR-R scale with a 0.05 two-sided significance level and a 90% power to detect a difference between the groups would results a sample size of n=74 per treatment arm. However, as the placebo-response rate in minors is probably higher compared to the single centre RCTs in aMDD integrated in the above mentioned meta-analyses, we calculated our sample size estimation under following more conservative assumptions:

a. P(omega-3) = .60, P(placebo) = .40
b. no adjustment for loss to follow-up (see below data analysis)
c. no adjustment for multiple comparisons; and
d. α level of .05 for a 2-tailed test chi-scare test with continuity correction.

Under these assumptions, 108 patients per treatment group (n = 216) will be needed to achieve 80% or greater power to detect a difference of 20% in response rates between the two treatment groups (what is considered a clinical meaningful difference). The two RCTs in pMDD that were used as the foundation for the proposed trial design (the TADS [253, 254] and Emslie RCT in pMDD [213]) had similar sample sizes.

11.3 Statistical Criteria of Termination of Trial

The Independent Data Monitoring Committee (has the responsibility to prevent harm as a consequence of the RCT. Therefore, the core task of the IDMC will be to monitor the safety of the trial. Every SAE and SUSAR will be reported to the IDMC who is the sole group of researchers that can access SecuTrial in an unblinded manner and are able to investigate if there is a true relationship of a SAE with the study drug. The trial will be terminated if the IDMC considers the safety of the study population seriously at risk as a consequence of their monitoring activity, or if a clinical highly significant and relevant superiority/ inferiority of the active treatment group could be shown with sufficient certainty. However, premature early termination of the RCT for benefit reasons manifest a serious problem for adequate reporting and is likely associated with effect overestimation as could be shown in oncology. As all participants will receive treatment according to the S3 German Guidelines for the treatment of depressive disorders in children and adolescents, premature termination for benefit reason shall only be considered if effect size between treatment groups is considered large (SMD > .0.8) on the children's depression rating scale, as well as clinical significant in recovery rates between the two treatment groups. The first interims analysis shall be performed after completion of 60 participants.

11.4 Planned Analyses

All analyses will be carried out after closure of the database to avoid unblinding with the exception of baseline data, which might be exported previously for master and PhD projects. All randomized patients will be included in the model, and the efficacy of omega-3 fatty acids on depressive symptoms will be analyzed using a linear random coefficient regression model, as described below.
Furthermore, we will compare the scores between baseline and end of study (week 36) for all the different scales and questionnaires, which were assessed previously. Additionally, cognitive functioning will be compared between the beginning of the study and its end while controlling for the effect of depressive symptoms on cognitive outcome measures. An SOP will describe all planned a priori analysis.

11.4.1 Datasets to be Analysed, Analysis Populations

The intention-to-treat (ITT) approach includes all randomized patients. In order to account for the effects of antidepressant medication we will perform following analyses: The anticipated proportion of patients receiving additional antidepressants at some point during follow-up is estimated to be around 30%. The analysis of treatment efficacy of Omega-3 fatty acids needs to address that patients received additional antidepressants, and we propose the following analyses:

- Perform analysis according to intention to treat (ITT) principle including all randomized patients in their treatment groups, and ignoring the additional antidepressant prescription.
- Perform primary endpoint analysis considering the outcomes of patients with additional antidepressant prescription as missing values (from the time point of the antidepressant prescription).
- Both analyses described above will be prone to bias, because there might be differential

antidepressant prescription, related to treatment group. In the efficacy analysis of the outcomes additional antidepressant prescription should not be ignored. We propose an approach in which the “missing” outcomes of patients with antidepressant prescriptions will be multiply imputed. By this approach, we mimic observations of depression scores that one would have received without antidepressant prescription. We plan to perform 10-fold multiple imputation. The missingness generating mechanism is considered to be “missing not at random” (MNAR), so the imputation method will need to address this. A method proposed by S. Jolani and S. van Buuren, 2013) is the “drawn indicator method” for missingness not at random.

- A subsequent sensitivity analysis of the results will reveal the dependence of the results on specific assumptions during the multiple imputation process.
- Furthermore, we plan to reanalyze the IIT sample according to the PUFA baseline status. In particular we want to compare participants with a omega-3 index <3 compared to those with an omega-3 index >3/5 or 8, as increasing evidence suggest that in particular those with a low omega-3 index may benefit from omega-3 supplementation.
- Furthermore, we plan to investigate the influence of inflammatory mediators as response predictors [63].
- Finally we will reanalyze the ITT sample using compliance data (pill count, rbc EPA/DPA change levels, MARS score) to compare fully compliant study participants with partially compliant and non-compliant patients.
- More exploratory will be the analysis of EPA metabolites (HETE; E-series resolvins, prostaglandine, leukotriene) that will be measured in the context of a PhD project as potential predictors of omega-3 fatty acid response.

11.4.2 Primary Analysis

The continuous primary outcome measure is the CDRS-R total score. The dichotomous primary outcome measures are recovery, response and remission. Recovery is defined as the absence of the diagnosis of MDD for > than 4 months according to the K-SADS, remission is defined as a CDRS total score <28 and response is defined as a 30% drop in CRDS total baseline score (post placebo lead in). We will analyze efficacy measures using a (generalized) linear random effects regression model (similar to the TADS study [253, 254]). Random regression in an ITT sample permits estimation of changes in continuous repeated measures in the presence of missing data on both a population and participant-specific level without necessitating last observation carried forward or exclusion of participants because of missing data. We will model the impact of treatment on outcome as a linear function of fixed effects for treatment, time (defined as the natural log of days since baseline + 1) and treatment-by-time interaction and random effects for participant and clinical site, including all 2- and 3-way interactions in the initial model. Clinical site and its interaction effects will be fitted in the model as random effects. Under the assumption of random intercepts and slopes for each patient, the overall and treatment group-specific rate of change for the 2 treatment groups for the primary CDRS-R outcome will be examined. A comparison on treatment slopes (linear trends with time) will then be conducted. Supplemental between-treatment contrast analyses will be conducted on the adjusted week 12 and 36 means. We will ass adherence using pill count, MARS-D, as well as EPA baseline change levels at week

12 and week 36. We will reanalyse the data according to fully adherent, partial adherent and non-adherent.

11.4.3 Secondary Analyses

For analysis of the secondary clinician-rated outcome CGI-improvement (CGI-I), the values at the measurement time points will be compared, since this scale inherently measures total improvement. We will compare the clinician rated CGI remission rates (defined as a score of <2) with the researcher-rated remission rates based on the CDRS-R/ K-SADS-P. CGI-I remission rates will be dichotomized by the end-of-treatment CGI-I score for each treatment group and will be compared using a logistic regression model using multiple imputations for missing data with site as a covariate.

For further secondary outcomes, we will apply the same statistical strategy as for the primary outcome measures. Analysis will include changes in overall and subscores of the in chapter 9.2 listed secondary outcome instruments. We will analyze CDRS-R subscores, DIKJ, BHS, BAI, SIQ-Jr, IES-27, KIDscreen-CAT-27, PSS-10, and Resilience Scale scores and compare them between the treatment groups.
Furthermore, we'll run the following analyses to test our secondary working hypotheses:

- **WH2a**: We will test differences in antidepressant medication between the treatment groups by means of the two proportions z-Test
- **WH2b**: We will compare level of functioning and quality of life between the treatment groups by comparing the scores of CGAS and the KIDscreen-CAT-27 with an independent two-sample t-Test or Mann-Whitney-U-Test, depending on the distribution of the data.
- **WH2c**: We will test the hypothesis of fewer hospital admission and lower outpatient service in the Omega-3 fatty acids group than the placebo group by applying an independent t-Test (for normal distributed data) or Mann-Whitney-U-Test (for non-normal data).
- **WH2d**: Retention rates between the two groups will be tested by means of the two proportions z-Test. In addition, Kaplan-Meier survival curves will be compared using the log rank test, and possible mediators of drop-out rates will be assessed with a cox regression analysis.
- **WH3a**: We will test the effect of inflammatory mediators on the efficacy of omega-3 treatment by introducing an additional continuous factor (ratio between pro- and anti-inflammatory markers) into the linear regression model described in 11.4.2.
- **WH3b**: We will calculate an omega-3 index based on red blood cell Omega-3 fatty acids content and introduce an additional binary factor (n-6/n-3 Omega ration < 3 vs. n-6/n-3 Omega ratio > 5) into the linear regression model described in 11.4.2.
- **WH3c**: We will test whether direct metabolites of EPA are a strong predictor of Omega-3 fatty acids by introducing additional continuous factors (levels of HETEs and E-series-resolvins at baseline) into the linear regression model described in 11.4.2
- **WH4a**: We will test association between pro-inflammatory state and depressive symptoms by calculating Pearson's correlation coefficient or Spearman's

correlation coefficient between the severity of symptoms at baseline and the ratio between pro- and anti-inflammatory markers. The same procedure is also applied to omega-6/3 ratio and omega-3 index. To test whether normalization of these parameters will correlate with improvements in depression scores we'll run a linear regression model with depression scores as dependent, and time and pro-inflammatory state, increase in omega-6/3 and omega-3 index as independent variables.

- **WH4b**: We will calculate Pearson's correlation coefficient or Spearman's correlation coefficient of omega-3 index with SIQ-Jr scores depending on the distribution of the data.
- **WH4c**: As above, we will calculate either Pearson's correlation coefficient or Spearman's correlation coefficient (depending on the distribution of the data) between omega-3 index and scores on the IES-27 and overall psychopathology scores.
- **WH4d**: We also test correlations between levels of Omega-3 fatty acids with salvia cortisol, F2 isoprostanes and Connor-Davidsons Resilience Scale scores by calculating Pearson's correlation coefficient or Spearman's correlation coefficient, respectively.
- **WH5a**: In order to compare treatment groups regarding occurrence of SUSARs, we will run a two proportions z-Test. Differences in suicidal ideation scores are tested by independent t-Test (for normal distributed data) or Mann-Whitney-U-Test (for non-normal data).

The analyses will be performed after closure of the database. The trial statisticians will perform all a-priori defined statistical analyses according to the statistical analyses plan. All other clinical outcome analyses will be declared as post-hoc analysis.

Future analysis based on the BioBank or other add on projects will be subject of an own protocol with separately formulated hypotheses that will be approved the appropriate authorities.

11.4.4 Interim Analyses

No interim analyses are carried out to maintain the blinding of all researchers over the whole course of the study. Only the IDMC will run an interim analysis after 60 patients to ensure the safety of the patients as specified in the IDMC SOP. If the IDMC considers the number of study participants insufficient to address the primary outcome in the due course of the study (e.g. because of too many drop outs, not sufficient prepubertal participants, other reasons…), the total sample size may be reconsidered.

11.4.5 Safety Analysis

Safety and harms frequency data will be described based on actual treatment received using MedDRA (integrated in SecuTrial®) and the self-report side effect checklist (ASEC). The rate of harm- and suicide-related adverse events (e.g. SIQ-Jr) in each treatment group will be compared using $\chi 2$ and Fisher exact tests, and ORs will be calculated to provide an indicator of relative risk of the active versus the placebo condition.

11.4.6 Deviation(s) from the Original Statistical Plan

We will report protocol violations (non-adherence to the treatment protocol) in the CONSORT flow chart, in the methodology section and if appropriate in the discussion. We will state how many participants will have been included in the placebo-lead in phase, how many of those dropped below a CDRS-R score of 40 within the lead in phase, how many will have been randomized, how many droped out within the first six weeks due to non-compliance and how many will have completed the acute and maintenance treatment phase. The intention-to-treat (ITT) approach includes all randomized patients. We will declare all analysis not defined a priori as post hoc analysis.

11.5 Handling of Missing Data and Drop-Outs

As stated above, the applied regression model already deals with missing data. For all other analysis we will use multiple imputations for missing data as described by Rubin [255, 256] using the software 'SOLAS for missing data analysis' (version 3.0).

12 ELIGIBILITY OF THE PROJECT SITE(S)

The department of Child and Adolescent Psychiatry of the University of Zurich is the biggest institution of its kind in Switzerland with close to 400 employees and is well suited to accommodate a clinical study. All the project sites are well-established official treatment centres for pMDD, and emergency care is readily available at each institution. All the centres are in close proximity to the lead centre, and many of the senior clinicians were trained or worked as consultants in the academic lead centre (ZH). Furthermore, the academic and non-academic centres have a joint training program for their trainees for several years providing a good foundation for similar concomitant treatment of pMDD.

13 DATA QUALITY ASSURANCE AND CONTROL

The Sponsor-Investigator is implementing and maintaining quality assurance and quality control systems with written SOPs and Working Instructions to ensure that trials are conducted and data are generated, documented (record), and reported in compliance with the protocol, GCP, and applicable regulatory requirement(s).

The Sponsor-Investigator is responsible to have written SOP`s and WIs in place for the study and to provide those to all participating study sites. The Principal Investigators at all sites must have a manual of the relevant SOPs and WIs for the study on site and are responsible for proper training of all involved study personnel for the respective procedures.

Monitoring and Audits will be conducted during the course of the study for quality assurance purposes.

13.1 Data Handling and Record Keeping / Archiving

The study will strictly follow the protocol. If any changes become necessary, they must be laid down in an amendment to the protocol. All amendments of the protocol must be signed by the Sponsor-Investigator and submitted to CEC and Swissmedic.

13.1.1 Case Report Forms

The investigators will use electronic case report forms (eCRF), one for each enrolled study participant, to be filled in with all relevant data pertaining to the participant during the study. All participants who either entered the study or were considered not-eligible or were eligible but not enrolled into the study additionally have to be documented on a screening log. The investigator will document the participation of each study participant on the Enrolment Log.

For data and query management, monitoring, reporting and coding an internet-based secure data base secuTrial® developed in agreement to the Good Clinical Practice (GCP) guidelines provided by the Clinical Trials Center (CTC) Zurich will be used for this study. It is the responsibility of the investigator to assure that all data in the course of the study will be entered completely and correctly in the respective data base. Corrections in the eCRF may only be done by the investigator or by other authorised persons. In case of corrections the original data entries will be archived in the system and can be made visible. For all data entries and corrections date, time of day and person who is performing the entries will be generated automatically.

ECRFs must be kept current to reflect participant status at each phase during the course of study. Participants must not to be identified in the eCRF by name. Appropriate coded identification (e.g. Participant Number) must be used.

It must be assured that any authorised person, who may perform data entries and changes in the eCRF, can be identified. A list with signatures and initials of all authorised persons will be filed in the study site file and the trial master file, respectively.

Documented medical histories and narrative statements relative to the participant’s progress during the study will be maintained. These records will also include the following: originals or copies of laboratory and other medical test results which must be kept on file with the individual participants’ eCRF. A detailed listing of all the study parameters for each participant can be found in the Study Manual.

The investigators assure to perform a complete and accurate documentation of the participant data in the eCRF. All data entered into the eCRF with exception of self-rating instruments that are entered directly online by the study participant/ parents and computerized neurocognitive data (for which data the eCRF will be source data) will also be available in the individual participant file either as print-outs or as notes taken by either the investigator or another responsible person assigned by the investigator.

Essential documents must be retained for at least 10 years after the regular end or a premature termination of the respective study (KlinV Art. 45). Any patient files and source data must be archived for the longest possible period of time according to the feasibility of the investigational site, e.g. hospital, institution or private practice.

13.1.2 Specification of Source Documents

The following documents are considered source data, including but not limited to:

• SAE worksheets

• Nurse records, records of clinical coordinators, and

• Medical records from other department(s), or other hospital(s), or discharge letters and correspondence with other departments/hospitals, if participant visited any during the study period and the post study period.

Source data must be available at the site to document the existence of the study participants and substantiate the integrity of study data collected. Source data must include the original documents relating to the study, as well as the medical treatment and medical history of the participant.

The following information (at least but not limited to) should be included in the source documents:

- Demographic data (age, sex)
- Inclusion and Exclusion Criteria details
- Participation in study and signed and dated Informed Consent Forms
- Visit dates
- Medical history and physical examination details (including documentation of scars)
- Key efficacy and safety data (as specified in the protocol)
- AEs and concomitant medication
- Results of relevant examinations
- Laboratory printouts
- Dispensing and return of study drug details
- Reason for premature discontinuation
- Randomization number

13.1.3 Record Keeping / Archiving

In accordance with Swiss national laws and guidelines and the specifications of the ICH-GCP guidelines, the investigators from participating sites and the coordinating centre are obligated to archive all documents pertaining to the study for 10 years after the last subject has completed or discontinued from the study. Electronic data will be archived at the CTC on a server pertaining to the University hospital of Zurich.

13.2 Data Management

Research assistants and local investigators will enter the acquired data and examination results into an eCRF (SecuTrail®) that is accessible via the internet. Investigators will receive personal

user names and passwords for this purpose, and data will be encrypted for transfer. For each site, it will be agreed before the start of the study which documents serve as source documents for all data entered into the eCRF. The investigator must (electronically) sign that entries into the eCRF are true and complete. At least a subset of data will be entered doubly to ensure data quality.
Study sites and associated investigators will be carefully selected and comprehensively informed and trained regarding GCP, all study procedures and the required examinations and documentation before the start of the study. The quality of data acquisition will be ensured by regular monitoring visits and the continuous availability of the monitor and trial-coordinator for consultation. After data have been submitted to the study centre, another thorough inspection of the completeness and plausibility of entries will be conducted. If needed, questions for clarification will be addressed to the sites. Only after all questions regarding data quality have been answered, the database will be locked.

13.2.1 Data Management System

For the electronic data capture, storage, monitor and export for statistical analyses, the latest version of the study software SecuTrial® will be used. SecuTrial® meets the regulatory requirements according to GCP and FDA 21 CFR Part 11. SecuTrial® is installed on a secure server provided by the University Hospital of Zurich. Only members of the CTC can access the server. The sponsor will set up the software before starting the trial under close supervision of the data manager of the CTC. All users will be trained in the handling of the software and SOPs will be available at each site file and in the master trial file.

13.2.2 Data Security, Access and Back-up

The sponsor is responsible for granting access to the electronic data. Every involved party will get his own password protected login and associated authorization. An audit trail documents all changes that are made in the system. All communication between the software and its users will be encrypted and a firewall protects the access to the software. A backup of the data will be made every 24 hours, and the University Hospital of Zurich provides a backup server in case the main server would be compromised.

13.2.3 Analysis and Archiving

After data validation the sponsor will notify the CTC about the closure of the database. After that, no new data or changes to existing data can be made. However, the data can still be accessed and be used for statistical analyses. After the last statistical analyses have been completed, the sponsor will notify the data manager about the timing of the archiving of the database, after which the project cannot be accessed anymore. After archiving, the database can only be accessed by written request of the sponsor or on request of the relevant authorities.

13.2.4 Electronic and Central Data Validation

SecuTrial® includes among others Audit-Trial, electronic signature, programmable tests of plausibility, consistency and value range, a query management system for online monitoring and a customizable system to assign roles and authorizations. Details for the software and its functionalities are described in separate documents which can be provided on request. The CTC will perform checks of accumulating study data. These are performed regularly through the study, and include range, plausibility, and consistency checks.

13.3 Monitoring

Regular monitoring visits at the investigator's site prior to the start and during the course of the study will help to follow up the progress of the clinical study, to assure utmost accuracy of the data and to detect possible errors at an early time point. The Sponsor-Investigator organises professional independent monitoring for the study.

All original data including all patient files, progress notes and copies of laboratory and medical test results must be available for monitoring. The project monitor, located at the CTC Zurich, will perform monitoring according to national laws and guidelines and the specifications of the ICH-GCP guidelines. The CTC project monitor will visit study sites at regular intervals to monitor the execution of the study. They will have access to all documents that are needed to perform their task according to the above mentioned guidelines. The CTC project monitor will check whether requirements to conduct the study are met and study procedures are followed correctly, and will check the study site's documentation, the participants' source data, eCRF entries, and the correct maintenance of the Investigator Site File. The monitor will review all or a part of the eCRFs and written informed consents. The accuracy of the data will be verified by reviewing the above referenced documents. The investigator's site will collaborate with the Clinical Trials Center (CTC) of the University Hospital Zurich to ensure regular monitoring. According to the CTC's Monitoring SOP the extent and nature of monitoring activities based on the objective and design of the study will be defined in a study specific Monitoring Plan.

13.4 Audits and Inspections

A quality assurance audit/inspection of this study may be conducted by the competent authority or CEC, respectively. The quality assurance auditor/inspector will have access to all medical records, the investigator's study related files and correspondence, and the informed consent documentation that is relevant to this clinical study.

The investigator will allow the persons being responsible for the audit or the inspection to have access to the source data/documents and to answer any questions arising. All involved parties will keep the patient data strictly confidential.

13.5 Confidentiality, Data Protection

Direct access to source documents will be permitted for purposes of monitoring, audits and inspections. Privacy laws and regulations will be adhered to during all procedures related to this study. The collection and processing of participants' personal information will be limited to what is necessary to insure the study's scientific practicability and to assess the research questions.
Information collected about participants during this clinical investigation will be treated confidentially. The local investigator or her/his co-workers will collect data and transfer it without recording the subject's name or date of birth, but coded with a subject identification code. Therefore, data is not directly traceable to individual subjects. A subject identification code links the data to the individual subject.
The code will be safeguarded by the data manager of the CTC Zürich; the key to this subject identification code will be kept at the CTC (via SecuTrial®). The emergency unblinding procedure or access to the randomisation code list by the authorities or the IDMC is described above.
Anonymous (coded) data can be relayed to the central study team for scientific analysis or made available, if necessary, to the responsible federal supervisory authority (in case of audits during the course of the study).
Only qualified and authorised collaborators of the study sponsor will enter the pseudonymous data into a computerised database. The acquired data will be used without participants' names for scientific analysis. Participants' names will not be mentioned in any publication of study results. Persons monitoring the data will have access to all information needed to ensure the validity of the study data, and are required to keep information confidential as well as to respect data privacy. Participants have the right to look into their data and can check their data in accordance with the relevant judicial regulations and procedures.

13.6 Storage of Biological Material and Related Health Data

A tissue repository (BioBank) will be established in accordance with the HFG/KlinV/HFV. In a separate consent form, subjects will be asked to provide blood for BioBanking in accordance with the Swiss legal regulations. If subjects do not want to have their blood samples stored, they can still participate in the study.

14 PUBLICATION AND DISSEMINATION POLICY

After the statistical analysis of this trial the sponsor will make every endeavour to publish the data in a medical journal. A Publications Committee (applicants, co-applicants, others) will be formed and establish a publication guideline. The scientific integrity of the project requires that the data from all omega-3-pMDD study sites will be analyzed and reported as such (whatever outcome the omega-3 pMDD Study will have). An individual center is not expected to report the data collected from its center alone. All presentations and publications are expected to protect the

integrity of the major objective(s) of the study; Data that the IDMC potentially generates will not be presented prior to the release of mainline results. Recommendations as to the timing of presentation of such endpoint data and the meetings at which they might be presented will be given by the Publications Committee. Data will be presented as original papers in key journals. Prior to study commencement, the main and co-applicants will contact different high impact journals (JAMA, Lancet, NEJM) to explore their interest, if they would consider to publish the original key paper once the study is completed and the main publication is finalized. They will be granted access to the protocol after signing a confidentiality agreement in case they want to have some adaptions.
Once the study has commenced, a trials methodology paper will be submitted. Once the recruitment phase has been completed, the demographic data and patient characteristic will be published. Papers will be published alongside the formulated hypothesis. A key responsible researcher will be appointed to drive the data analysis and writing of the paper. The senior researcher is responsible for the establishment of an optimal team of experts to address the key research questions. Each paper or abstract must be submitted to the appropriate Publications committee for review of its appropriateness and scientific merit prior to submission. The final approval is granted by the applicant and co-applicants (which form the publications committee). Study results will be presented at local, national and international conferences of the field (e.g. Swiss Society of Child and Adolescent annual meeting, the German Society of Child and Adolescent Psychiatry annual meeting, APA, ECNP, IEPA and other conferences).

Reproducibility
No later than 5 years after the collection of the 36-week post-randomisation interviews, we will deliver a completely de-identified data set to an appropriate data archives for sharing purposes.

15 FUNDING AND SUPPORT

15.1 Funding

This study is financed by the Swiss National Science Foundation SNSF (Nr. 33IC30-166826).

15.2 Other Support

The study is also supported by the University of Zurich, Department of Child and Adolescent Psychiatry (Prof. S. Walitza).

16 INSURANCE

Insurance is covered by “Haftpflichtversicherung für den Kanton Zürich betreffend das UniversitätsSpital Zürich“ (Policy no.: 14.970.885*).*

Any damage developed in relation to study participation is covered by this insurance. So as not to forfeit their insurance cover, the participants themselves must strictly follow the instructions of the study personnel. Participants must not be involved in any other medical treatment without permission of the principal investigator (emergency excluded). Medical emergency treatment must be reported immediately to the investigator. The investigator must also be informed instantly, in the event of health problems or other damages during or after the course of study treatment.

The investigator will allow delegates of the insurance company to have access to the source data/documents as necessary to clarify a case of damage related to study participation. All involved parties will keep the patient data strictly confidential.

A copy of the insurance certificate will be placed in the Investigator's Site File.

17 REFERENCES

1. Uher, R., et al., *Major depressive disorder in DSM-5: implications for clinical practice and research of changes from DSM-IV.* Depress Anxiety, 2014. **31**(6): p. 459-71.
2. Kovacs, M., et al., *Depressive disorders in childhood. I. A longitudinal prospective study of characteristics and recovery.* Arch Gen Psychiatry, 1984. **41**(3): p. 229-37.
3. Kovacs, M., *Presentation and course of major depressive disorder during childhood and later years of the life span.* J Am Acad Child Adolesc Psychiatry, 1996. **35**(6): p. 705-15.
4. Birmaher, B., et al., *Clinical presentation and course of depression in youth: does onset in childhood differ from onset in adolescence?* J Am Acad Child Adolesc Psychiatry, 2004. **43**(1): p. 63-70.
5. Schuler, D., et al., *Gesundheit im Kanton Zürich. Kurzfassung: Ergebnisse der Schweizerischen Gesundheitsbefragung 2012.*, in *Institut für Sozial- und Präventivmedizin der Universität Zürich* 2014: Zürich.
6. Steinhausen, H.C., *Developmental psychopathology in adolescence: findings from a Swiss study--the NAPE Lecture 2005.* Acta Psychiatr Scand, 2006. **113**(1): p. 6-12.
7. Kessler, R.C., et al., *Age of onset of mental disorders: a review of recent literature.* Curr Opin Psychiatry, 2007. **20**(4): p. 359-64.
8. Scott, E.G., et al., *Screening for adolescent depression in a pediatric emergency department.* Acad Emerg Med, 2006. **13**(5): p. 537-42.
9. Ghio, L., et al., *Duration of untreated illness and outcomes in unipolar depression: a systematic review and meta-analysis.* J Affect Disord, 2014. **152-154**: p. 45-51.
10. Gore, F.M., et al., *Global burden of disease in young people aged 10-24 years: a systematic analysis.* Lancet, 2011. **377**(9783): p. 2093-102.
11. Lewinsohn, P.M., et al., *Natural course of adolescent major depressive disorder in a community sample: predictors of recurrence in young adults.* Am J Psychiatry, 2000. **157**(10): p. 1584-91.
12. Korczak, D.J. and B.I. Goldstein, *Childhood onset major depressive disorder: course of illness and psychiatric comorbidity in a community sample.* J Pediatr, 2009. **155**(1): p. 118-23.
13. Weissman, M.M., et al., *Depressed adolescents grown up.* JAMA, 1999. **281**(18): p. 1707-13.
14. Ferrari, A.J., et al., *The burden attributable to mental and substance use disorders as risk factors for suicide: findings from the Global Burden of Disease Study 2010.* PLoS One, 2014. **9**(4): p. e91936.
15. Richardson, L.P., et al., *Collaborative care for adolescents with depression in primary care: a randomized clinical trial.* JAMA, 2014. **312**(8): p. 809-16.
16. Cox, G.R., et al., *Psychological therapies versus antidepressant medication, alone and in combination for depression in children and adolescents.* Cochrane Database Syst Rev, 2012. **11**: p. CD008324.
17. Hetrick, S.E., et al., *Newer generation antidepressants for depressive disorders in children and adolescents.* Cochrane Database Syst Rev, 2012. **11**: p. CD004851.
18. Cox, G.R., et al., *Interventions for preventing relapse and recurrence of a depressive disorder in children and adolescents.* Cochrane Database Syst Rev, 2012. **11**: p. CD007504.
19. Newman, T.B., *A black-box warning for antidepressants in children?* N Engl J Med, 2004. **351**(16): p. 1595-8.
20. Ho, D., *Antidepressants and the FDA's black-box warning: determining a rational public policy in the absence of sufficient evidence.* Virtual Mentor, 2012. **14**(6): p. 483-8.
21. Hawton, K., K.E. Saunders, and R.C. O'Connor, *Self-harm and suicide in adolescents.* Lancet, 2012. **379**(9834): p. 2373-82.
22. Barry, C.L. and S.H. Busch, *News coverage of FDA warnings on pediatric antidepressant use and suicidality.* Pediatrics, 2010. **125**(1): p. 88-95.
23. Hernandez, J.F., et al., *A 10-year analysis of the effects of media coverage of regulatory warnings on antidepressant use in The Netherlands and UK.* PLoS One, 2012. **7**(9): p. e45515.
24. Olfson, M., S.C. Marcus, and B.G. Druss, *Effects of Food and Drug Administration warnings on antidepressant use in a national sample.* Arch Gen Psychiatry, 2008. **65**(1): p. 94-101.
25. Doshi, P., *EFPIA-PhRMA's principles for clinical trial data sharing have been misunderstood.* BMJ, 2013. **347**: p. f5164.
26. Doshi, P., *No correction, no retraction, no apology, no comment: paroxetine trial reanalysis raises questions about institutional responsibility.* BMJ, 2015. **351**: p. h4629.

27. Le Noury, J., et al., *Restoring Study 329: efficacy and harms of paroxetine and imipramine in treatment of major depression in adolescence.* BMJ, 2015. **351**: p. h4320.
28. Gibbons, R.D., et al., *Early evidence on the effects of regulators' suicidality warnings on SSRI prescriptions and suicide in children and adolescents.* Am J Psychiatry, 2007. **164**(9): p. 1356-63.
29. Gibbons, R.D., et al., *Suicidal thoughts and behavior with antidepressant treatment: reanalysis of the randomized placebo-controlled studies of fluoxetine and venlafaxine.* Arch Gen Psychiatry, 2012. **69**(6): p. 580-7.
30. Berger, G.E., A. Della Casa, and D. Pauli, *Suizidalität bei Adoleszenten – Prävention und Behandlung.* Therapeutische Rundschau, 2015. **72**(10): p. 619-632.
31. Lu, C.Y., et al., *Changes in antidepressant use by young people and suicidal behavior after FDA warnings and media coverage: quasi-experimental study.* BMJ, 2014. **348**: p. g3596.
32. Beardslee, W.R., et al., *Prevention of depression in at-risk adolescents: longer-term effects.* JAMA Psychiatry, 2013. **70**(11): p. 1161-70.
33. Garber, J., et al., *Prevention of depression in at-risk adolescents: a randomized controlled trial.* JAMA, 2009. **301**(21): p. 2215-24.
34. Dolle, K. and G. Schulte-Korne, *The treatment of depressive disorders in children and adolescents.* Dtsch Arztebl Int, 2013. **110**(50): p. 854-60.
35. Volkers, A.C., E.R. Heerdink, and L. van Dijk, *Antidepressant use and off-label prescribing in children and adolescents in Dutch general practice (2001-2005).* Pharmacoepidemiol Drug Saf, 2007. **16**(9): p. 1054-62.
36. Rushton, J.L., S.J. Clark, and G.L. Freed, *Pediatrician and family physician prescription of selective serotonin reuptake inhibitors.* Pediatrics, 2000. **105**(6): p. E82.
37. Simopoulos, A.P., *Evolutionary aspects of diet: the omega-6/omega-3 ratio and the brain.* Mol Neurobiol, 2011. **44**(2): p. 203-15.
38. Simopoulos, A.P., *Omega-3 fatty acids in health and disease and in growth and development.* Am J Clin Nutr, 1991. **54**(3): p. 438-63.
39. Simopoulos, A.P., *Omega-3 fatty acids in inflammation and autoimmune diseases.* J Am Coll Nutr, 2002. **21**(6): p. 495-505.
40. Bountziouka, V., et al., *Long-term fish intake is associated with less severe depressive symptoms among elderly men and women: the MEDIS (MEDiterranean ISlands Elderly) epidemiological study.* J Aging Health, 2009. **21**(6): p. 864-80.
41. Tanskanen, A., et al., *Fish consumption, depression, and suicidality in a general population.* Archives of General Psychiatry, 2001. **58**(5): p. 512-3.
42. Timonen, M., et al., *Fish consumption and depression: the Northern Finland 1966 birth cohort study.* J Affect Disord, 2004. **82**(3): p. 447-52.
43. Suominen-Taipale, A.L., et al., *Fish consumption and omega-3 polyunsaturated fatty acids in relation to depressive episodes: a cross-sectional analysis.* PLoS One, 2010. **5**(5): p. e10530.
44. Appleton, K.M., et al., *Depressed mood and dietary fish intake: direct relationship or indirect relationship as a result of diet and lifestyle?* J Affect Disord, 2007. **104**(1-3): p. 217-23.
45. Murakami, K., et al., *Fish and n-3 polyunsaturated fatty acid intake and depressive symptoms: Ryukyus Child Health Study.* Pediatrics, 2010. **126**(3): p. e623-30.
46. Li, Y., et al., *Fish consumption and severely depressed mood, findings from the first national nutrition follow-up study.* Psychiatry Res, 2011. **190**(1): p. 103-9.
47. Sanchez-Villegas, A., et al., *Long chain omega-3 fatty acids intake, fish consumption and mental disorders in the SUN cohort study.* Eur J Nutr, 2007. **46**(6): p. 337-46.
48. Astorg, P., et al., *Association of fish and long-chain n-3 polyunsaturated fatty acid intakes with the occurrence of depressive episodes in middle-aged French men and women.* Prostaglandins Leukot Essent Fatty Acids, 2008. **78**(3): p. 171-82.
49. Hibbeln, J.R., *Fish consumption and major depression.* Lancet, 1998. **351**(9110): p. 1213.
50. Noaghiul, S. and J.R. Hibbeln, *Cross-national comparisons of seafood consumption and rates of bipolar disorders.* Am J Psychiatry, 2003. **160**(12): p. 2222-7.
51. Li, F., X. Liu, and D. Zhang, *Fish consumption and risk of depression: a meta-analysis.* J Epidemiol Community Health, 2015.
52. Huan, M., et al., *Suicide attempt and n-3 fatty acid levels in red blood cells: a case control study in China.* Biol Psychiatry, 2004. **56**(7): p. 490-6.
53. Sublette, M.E., et al., *Omega-3 polyunsaturated essential fatty acid status as a predictor of future suicide risk.* Am J Psychiatry, 2006. **163**(6): p. 1100-2.

54. Lin, P.Y., S.Y. Huang, and K.P. Su, *A meta-analytic review of polyunsaturated fatty acid compositions in patients with depression.* Biol Psychiatry, 2010. **68**(2): p. 140-7.
55. De Vriese, S.R., A.B. Christophe, and M. Maes, *Lowered serum n-3 polyunsaturated fatty acid (PUFA) levels predict the occurrence of postpartum depression: further evidence that lowered n-PUFAs are related to major depression.* Life Sci, 2003. **73**(25): p. 3181-7.
56. Green, P., et al., *Red cell membrane omega-3 fatty acids are decreased in nondepressed patients with social anxiety disorder.* Eur Neuropsychopharmacol, 2006. **16**(2): p. 107-13.
57. Adams, P.B., et al., *Arachidonic acid to eicosapentaenoic acid ratio in blood correlates positively with clinical symptoms of depression.* Lipids, 1996. **31 Suppl**: p. S157-61.
58. Maes, M., et al., *Fatty acid composition in major depression: decreased omega 3 fractions in cholesteryl esters and increased C20: 4 omega 6/C20:5 omega 3 ratio in cholesteryl esters and phospholipids.* J Affect Disord, 1996. **38**(1): p. 35-46.
59. Edwards, R., et al., *Omega-3 polyunsaturated fatty acid levels in the diet and in red blood cell membranes of depressed patients.* Journal of Affective Disorders, 1998. **48**(2-3): p. 149-55.
60. Frasure-Smith, N., F. Lesperance, and P. Julien, *Major depression is associated with lower omega-3 fatty acid levels in patients with recent acute coronary syndromes.* Biol Psychiatry, 2004. **55**(9): p. 891-6.
61. Pottala, J.V., et al., *Red blood cell fatty acids are associated with depression in a case-control study of adolescents.* Prostaglandins Leukot Essent Fatty Acids, 2012. **86**(4-5): p. 161-5.
62. Young, J.J., D. Bruno, and N. Pomara, *A review of the relationship between proinflammatory cytokines and major depressive disorder.* J Affect Disord, 2014. **169**: p. 15-20.
63. Rapaport, M.H., et al., *Inflammation as a predictive biomarker for response to omega-3 fatty acids in major depressive disorder: a proof-of-concept study.* Mol Psychiatry, 2015.
64. De Caterina, R., *n-3 Fatty Acids in Cardiovascular Disease REPLY.* New England Journal of Medicine, 2011. **365**(12): p. 1159-1159.
65. Mozaffarian, D. and J.H.Y. Wu, *Omega-3 Fatty Acids and Cardiovascular Disease Effects on Risk Factors, Molecular Pathways, and Clinical Events.* Journal of the American College of Cardiology, 2011. **58**(20): p. 2047-2067.
66. Grosso, G., et al., *Omega-3 fatty acids and depression: scientific evidence and biological mechanisms.* Oxid Med Cell Longev, 2014. **2014**: p. 313570.
67. Gold, P.W., *The organization of the stress system and its dysregulation in depressive illness.* Mol Psychiatry, 2015. **20**(1): p. 32-47.
68. El-Ansary, A.K., S.K. Al-Daihan, and A.R. El-Gezeery, *On the protective effect of omega-3 against propionic acid-induced neurotoxicity in rat pups.* Lipids Health Dis, 2011. **10**: p. 142.
69. Song, C., et al., *Omega-3 fatty acid ethyl-eicosapentaenoate attenuates IL-1beta-induced changes in dopamine and metabolites in the shell of the nucleus accumbens: involved with PLA2 activity and corticosterone secretion.* Neuropsychopharmacology, 2007. **32**(3): p. 736-44.
70. Goshen, I., et al., *Brain interleukin-1 mediates chronic stress-induced depression in mice via adrenocortical activation and hippocampal neurogenesis suppression.* Mol Psychiatry, 2008. **13**(7): p. 717-28.
71. Larrieu, T., et al., *Nutritional omega-3 modulates neuronal morphology in the prefrontal cortex along with depression-related behaviour through corticosterone secretion.* Transl Psychiatry, 2014. **4**: p. e437.
72. Mocking, R.J., et al., *Relationship between the hypothalamic-pituitary-adrenal-axis and fatty acid metabolism in recurrent depression.* Psychoneuroendocrinology, 2013. **38**(9): p. 1607-17.
73. Assies, J., et al., *Effects of oxidative stress on fatty acid- and one-carbon-metabolism in psychiatric and cardiovascular disease comorbidity.* Acta Psychiatr Scand, 2014. **130**(3): p. 163-80.
74. Mocking, R.J., et al., *Fatty acid metabolism and its longitudinal relationship with the hypothalamic-pituitary-adrenal axis in major depression: Associations with prospective antidepressant response.* Psychoneuroendocrinology, 2015. **59**: p. 1-13.
75. Lyons, D.M., *Stress, depression, and inherited variation in primate hippocampal and prefrontal brain development.* Psychopharmacol Bull, 2002. **36**(1): p. 27-43.
76. McNamara, R.K., J.J. Vannest, and C.J. Valentine, *Role of perinatal long-chain omega-3 fatty acids in cortical circuit maturation: Mechanisms and implications for psychopathology.* World J Psychiatry, 2015. **5**(1): p. 15-34.
77. McNamara, R.K. and S.E. Carlson, *Role of omega-3 fatty acids in brain development and function: potential implications for the pathogenesis and prevention of psychopathology.* Prostaglandins Leukot Essent Fatty Acids, 2006. **75**(4-5): p. 329-49.

78. Bourre, J.M., *Roles of unsaturated fatty acids (especially omega-3 fatty acids) in the brain at various ages and during ageing.* J Nutr Health Aging, 2004. **8**(3): p. 163-74.
79. Janssen, C.I. and A.J. Kiliaan, *Long-chain polyunsaturated fatty acids (LCPUFA) from genesis to senescence: The influence of LCPUFA on neural development, aging, and neurodegeneration.* Prog Lipid Res, 2013. **53C**: p. 1-17.
80. Pudell, C., et al., *Fish oil improves anxiety-like, depressive-like and cognitive behaviors in olfactory bulbectomised rats.* Eur J Neurosci, 2014. **39**(2): p. 266-74.
81. Peters, B.D., et al., *Brain white matter development is associated with a human-specific haplotype increasing the synthesis of long chain fatty acids.* J Neurosci, 2014. **34**(18): p. 6367-76.
82. Bremner, J.D., et al., *Hippocampal volume reduction in major depression.* Am J Psychiatry, 2000. **157**(1): p. 115-8.
83. Song, C. and H. Wang, *Cytokines mediated inflammation and decreased neurogenesis in animal models of depression.* Prog Neuropsychopharmacol Biol Psychiatry, 2011. **35**(3): p. 760-8.
84. Song, C., X.Y. Zhang, and M. Manku, *Increased phospholipase A2 activity and inflammatory response but decreased nerve growth factor expression in the olfactory bulbectomized rat model of depression: effects of chronic ethyl-eicosapentaenoate treatment.* J Neurosci, 2009. **29**(1): p. 14-22.
85. Carlezon, W.A., Jr., et al., *Antidepressant-like effects of uridine and omega-3 fatty acids are potentiated by combined treatment in rats.* Biol Psychiatry, 2005. **57**(4): p. 343-50.
86. Huang, S.Y., et al., *Omega-3 fatty acids on the forced-swimming test.* J Psychiatr Res, 2008. **42**(1): p. 58-63.
87. Bartl, J., S. Walitza, and E. Grunblatt, *Enhancement of cell viability after treatment with polyunsaturated fatty acids.* Neurosci Lett, 2014. **559**: p. 56-60.
88. Delion, S., et al., *alpha-Linolenic acid dietary deficiency alters age-related changes of dopaminergic and serotoninergic neurotransmission in the rat frontal cortex.* J Neurochem, 1996. **66**(4): p. 1582-91.
89. Delion, S., et al., *Age-related changes in phospholipid fatty acid composition and monoaminergic neurotransmission in the hippocampus of rats fed a balanced or an n-3 polyunsaturated fatty acid-deficient diet.* J Lipid Res, 1997. **38**(4): p. 680-9.
90. Zimmer, L., et al., *Modification of dopamine neurotransmission in the nucleus accumbens of rats deficient in n-3 polyunsaturated fatty acids.* J Lipid Res, 2000. **41**(1): p. 32-40.
91. Yao, J.K., et al., *Effects of omega-3 fatty acid on platelet serotonin responsivity in patients with schizophrenia.* Prostaglandins Leukot Essent Fatty Acids, 2004. **71**(3): p. 171-6.
92. Song, C. and D. Horrobin, *Omega-3 fatty acid ethyl-eicosapentaenoate, but not soybean oil, attenuates memory impairment induced by central IL-1beta administration.* J Lipid Res, 2004. **45**(6): p. 1112-21.
93. Chalon, S., *Omega-3 fatty acids and monoamine neurotransmission.* Prostaglandins Leukot Essent Fatty Acids, 2006. **75**(4-5): p. 259-69.
94. McNamara, R.K., et al., *Omega-3 fatty acid deficiency during perinatal development increases serotonin turnover in the prefrontal cortex and decreases midbrain tryptophan hydroxylase-2 expression in adult female rats: dissociation from estrogenic effects.* J Psychiatr Res, 2009. **43**(6): p. 656-63.
95. Hibbeln, J.R., et al., *Essential fatty acids predict metabolites of serotonin and dopamine in cerebrospinal fluid among healthy control subjects, and early- and late-onset alcoholics.* Biol Psychiatry, 1998. **44**(4): p. 235-42.
96. Hibbeln, J.R., et al., *A replication study of violent and nonviolent subjects: cerebrospinal fluid metabolites of serotonin and dopamine are predicted by plasma essential fatty acids.* Biol Psychiatry, 1998. **44**(4): p. 243-9.
97. Leonard, B. and M. Maes, *Mechanistic explanations how cell-mediated immune activation, inflammation and oxidative and nitrosative stress pathways and their sequels and concomitants play a role in the pathophysiology of unipolar depression.* Neurosci Biobehav Rev, 2012. **36**(2): p. 764-85.
98. Lotrich, F.E., *Inflammatory cytokine-associated depression.* Brain Res, 2015. **1617**: p. 113-25.
99. Rosenblat, J.D., et al., *Inflamed moods: a review of the interactions between inflammation and mood disorders.* Prog Neuropsychopharmacol Biol Psychiatry, 2014. **53**: p. 23-34.
100. Serhan, C.N., *Pro-resolving lipid mediators are leads for resolution physiology.* Nature, 2014. **510**(7503): p. 92-101.

101. Freeman, M.P. and M.H. Rapaport, *Omega-3 fatty acids and depression: from cellular mechanisms to clinical care.* J Clin Psychiatry, 2011. **72**(2): p. 258-9.
102. Maes, M., et al., *Why fish oils may not always be adequate treatments for depression or other inflammatory illnesses: docosahexaenoic acid, an omega-3 polyunsaturated fatty acid, induces a Th-1-like immune response.* Neuro Endocrinol Lett, 2007. **28**(6): p. 875-80.
103. Muller, N., A.M. Myint, and M.J. Schwarz, *The impact of neuroimmune dysregulation on neuroprotection and neurotoxicity in psychiatric disorders--relation to drug treatment.* Dialogues Clin Neurosci, 2009. **11**(3): p. 319-32.
104. Berger, G.E., et al., *Ethyl-Eicosapentaenoic Acid in First-Episode Psychosis. A 1H-MRS Study.* Neuropsychopharmacology, 2008.
105. Duffy, S.L., et al., *The effect of 12-wk omega-3 fatty acid supplementation on in vivo thalamus glutathione concentration in patients "at risk" for major depression.* Nutrition, 2015. **31**(10): p. 1247-54.
106. Smesny, S., et al., *Effects of omega-3 PUFA on the vitamin E and glutathione antioxidant defense system in individuals at ultra-high risk of psychosis.* Prostaglandins Leukot Essent Fatty Acids, 2015. **101**: p. 15-21.
107. Said, T., et al., *Effects of vegetable oils on biochemical and biophysical properties of membrane retinal pigment epithelium cells.* Can J Physiol Pharmacol, 2013. **91**(10): p. 812-7.
108. Onuki, Y., et al., *Docosahexaenoic acid and eicosapentaenoic acid induce changes in the physical properties of a lipid bilayer model membrane.* Chem Pharm Bull (Tokyo), 2006. **54**(1): p. 68-71.
109. Wood, S.J., et al., *Neuroprotective effects of ethyl-eicosapentaenoic acid in first episode psychosis: a longitudinal T2 relaxometry pilot study.* Psychiatry Res, 2010. **182**(2): p. 180-2.
110. Peet, M. and D.F. Horrobin, *A dose-ranging study of the effects of ethyl-eicosapentaenoate in patients with ongoing depression despite apparently adequate treatment with standard drugs.* Arch Gen Psych, 2002. **59**(10): p. 913-9.
111. Nemets, B., Z. Stahl, and R.H. Belmaker, *Addition of omega-3 fatty acid to maintenance medication treatment for recurrent unipolar depressive disorder.* Am J Psychiatry, 2002. **159**(3): p. 477-9.
112. Marangell, L.B., et al., *A double-blind, placebo-controlled study of the omega-3 fatty acid docosahexaenoic acid in the treatment of major depression.* Am J Psychiatry, 2003. **160**(5): p. 996-8.
113. Su, K.P., et al., *Omega-3 fatty acids in major depressive disorder. A preliminary double-blind, placebo-controlled trial.* Eur Neuropsychopharmacol, 2003. **13**(4): p. 267-71.
114. Silvers, K.M., et al., *Randomised double-blind placebo-controlled trial of fish oil in the treatment of depression.* Prostaglandins Leukot Essent Fatty Acids, 2005. **72**(3): p. 211-8.
115. Grenyer, B.F., et al., *Fish oil supplementation in the treatment of major depression: a randomised double-blind placebo-controlled trial.* Prog Neuropsychopharmacol Biol Psychiatry, 2007. **31**(7): p. 1393-6.
116. Jazayeri, S., et al., *Comparison of therapeutic effects of omega-3 fatty acid eicosapentaenoic acid and fluoxetine, separately and in combination, in major depressive disorder.* Aust N Z J Psychiatry, 2008. **42**(3): p. 192-8.
117. Mischoulon, D., et al., *A double-blind, randomized controlled trial of ethyl-eicosapentaenoate for major depressive disorder.* J Clin Psychiatry, 2009. **70**(12): p. 1636-44.
118. Rondanelli, M., et al., *Effect of omega-3 fatty acids supplementation on depressive symptoms and on health-related quality of life in the treatment of elderly women with depression: a double-blind, placebo-controlled, randomized clinical trial.* J Am Coll Nutr, 2010. **29**(1): p. 55-64.
119. Rondanelli, M., et al., *Long chain omega 3 polyunsaturated fatty acids supplementation in the treatment of elderly depression: effects on depressive symptoms, on phospholipids fatty acids profile and on health-related quality of life.* J Nutr Health Aging, 2011. **15**(1): p. 37-44.
120. Lesperance, F., et al., *The efficacy of omega-3 supplementation for major depression: a randomized controlled trial.* J Clin Psychiatry, 2011. **72**(8): p. 1054-62.
121. Sinn, N., et al., *Effects of n-3 fatty acids, EPA v. DHA, on depressive symptoms, quality of life, memory and executive function in older adults with mild cognitive impairment: a 6-month randomised controlled trial.* Br J Nutr, 2012. **107**(11): p. 1682-93.
122. Gertsik, L., et al., *Omega-3 fatty acid augmentation of citalopram treatment for patients with major depressive disorder.* J Clin Psychopharmacol, 2012. **32**(1): p. 61-4.
123. Rizzo, A.M., et al., *Comparison between the AA/EPA ratio in depressed and non depressed elderly females: omega-3 fatty acid supplementation correlates with improved symptoms but does not change immunological parameters.* Nutr J, 2012. **11**: p. 82.

124. Stoll, A.L., et al., *Omega 3 fatty acids in bipolar disorder: a preliminary double-blind, placebo-controlled trial [see comments].* Arch Gen Psych, 1999. **56**(5): p. 407-12.
125. Hirashima, F., et al., *Omega-3 fatty acid treatment and T(2) whole brain relaxation times in bipolar disorder.* Am J Psychiatry, 2004. **161**(10): p. 1922-4.
126. Chiu, C.C., et al., *Omega-3 fatty acids are more beneficial in the depressive phase than in the manic phase in patients with bipolar I disorder.* J Clin Psychiatry, 2005. **66**(12): p. 1613-4.
127. Frangou, S., M. Lewis, and P. McCrone, *Efficacy of ethyl-eicosapentaenoic acid in bipolar depression: randomised double-blind placebo-controlled study.* Br J Psychiatry, 2006. **188**: p. 46-50.
128. Keck, P.E., Jr., et al., *Double-blind, randomized, placebo-controlled trials of ethyl-eicosapentanoate in the treatment of bipolar depression and rapid cycling bipolar disorder.* Biol Psychiatry, 2006. **60**(9): p. 1020-2.
129. Frangou, S., et al., *Preliminary in vivo evidence of increased N-acetyl-aspartate following eicosapentanoic acid treatment in patients with bipolar disorder.* J Psychopharmacol, 2007. **21**(4): p. 435-9.
130. Murphy, B.L., et al., *Omega-3 fatty acid treatment, with or without cytidine, fails to show therapeutic properties in bipolar disorder: a double-blind, randomized add-on clinical trial.* J Clin Psychopharmacol, 2012. **32**(5): p. 699-703.
131. Freeman, M.P., et al., *Omega-3 fatty acids and supportive psychotherapy for perinatal depression: a randomized placebo-controlled study.* J Affect Disord, 2008. **110**(1-2): p. 142-8.
132. Breland, D.J. and M.J. Park, *Depression: focus on the adolescent male.* Am J Mens Health, 2008. **2**(1): p. 87-93.
133. Rees, A.M., M.P. Austin, and G.B. Parker, *Omega-3 fatty acids as a treatment for perinatal depression: randomized double-blind placebo-controlled trial.* Aust N Z J Psychiatry, 2008. **42**(3): p. 199-205.
134. Rogers, P.J., et al., *No effect of n-3 long-chain polyunsaturated fatty acid (EPA and DHA) supplementation on depressed mood and cognitive function: a randomised controlled trial.* Br J Nutr, 2008. **99**(2): p. 421-31.
135. Lucas, M., et al., *Ethyl-eicosapentaenoic acid for the treatment of psychological distress and depressive symptoms in middle-aged women: a double-blind, placebo-controlled, randomized clinical trial.* Am J Clin Nutr, 2009. **89**(2): p. 641-51.
136. Tajalizadekhoob, Y., et al., *The effect of low-dose omega 3 fatty acids on the treatment of mild to moderate depression in the elderly: a double-blind, randomized, placebo-controlled study.* Eur Arch Psychiatry Clin Neurosci, 2011. **261**(8): p. 539-49.
137. Antypa, N., et al., *Effects of omega-3 fatty acid supplementation on mood and emotional information processing in recovered depressed individuals.* J Psychopharmacol, 2012. **26**(5): p. 738-43.
138. Mozaffari-Khosravi, H., et al., *Eicosapentaenoic acid versus docosahexaenoic acid in mild-to-moderate depression: a randomized, double-blind, placebo-controlled trial.* Eur Neuropsychopharmacol, 2013. **23**(7): p. 636-44.
139. Sohrabi, N., et al., *Evaluation of the effect of omega-3 fatty acids in the treatment of premenstrual syndrome: "a pilot trial".* Complement Ther Med, 2013. **21**(3): p. 141-6.
140. Behan, P.O., W.M. Behan, and D.F. Horrobin, *Effect of high doses of essential fatty acids on the postviral fatigue syndrome.* Acta Neurol Scand, 1990. **82**(3): p. 209-16.
141. Warren, G., M. McKendrick, and M. Peet, *The role of essential fatty acids in chronic fatigue syndrome. A case-controlled study of red-cell membrane essential fatty acids (EFA) and a placebo-controlled treatment study with high dose of EFA.* Acta Neurologica Scandinavica, 1999. **99**(2): p. 112-6.
142. Zanarini, M.C. and F.R. Frankenburg, *omega-3 Fatty acid treatment of women with borderline personality disorder: a double-blind, placebo-controlled pilot study.* Am J Psychiatry, 2003. **160**(1): p. 167-9.
143. Fux, M., J. Benjamin, and B. Nemets, *A placebo-controlled cross-over trial of adjunctive EPA in OCD.* J Psychiatr Res, 2004. **38**(3): p. 323-5.
144. Hallahan, B., et al., *Omega-3 fatty acid supplementation in patients with recurrent self-harm. Single-centre double-blind randomised controlled trial.* Br J Psychiatry, 2007. **190**: p. 118-22.
145. Fenton, W.S., et al., *A placebo-controlled trial of omega-3 fatty acid (ethyl eicosapentaenoic acid) supplementation for residual symptoms and cognitive impairment in schizophrenia.* Am J Psychiatry, 2001. **158**(12): p. 2071-4.

146. Chiu, C.C., et al., *The effects of omega-3 fatty acids monotherapy in Alzheimer's disease and mild cognitive impairment: a preliminary randomized double-blind placebo-controlled study.* Prog Neuropsychopharmacol Biol Psychiatry, 2008. **32**(6): p. 1538-44.
147. Freund-Levi, Y., et al., *Omega-3 supplementation in mild to moderate Alzheimer's disease: effects on neuropsychiatric symptoms.* Int J Geriatr Psychiatry, 2008. **23**(2): p. 161-9.
148. da Silva, T.M., et al., *Depression in Parkinson's disease: a double-blind, randomized, placebo-controlled pilot study of omega-3 fatty-acid supplementation.* J Affect Disord, 2008. **111**(2-3): p. 351-9.
149. Carney, R.M., et al., *Omega-3 augmentation of sertraline in treatment of depression in patients with coronary heart disease: a randomized controlled trial.* JAMA, 2009. **302**(15): p. 1651-7.
150. Bot, M., et al., *Eicosapentaenoic acid as an add-on to antidepressant medication for co-morbid major depression in patients with diabetes mellitus: a randomized, double-blind placebo-controlled study.* J Affect Disord, 2010. **126**(1-2): p. 282-6.
151. Giltay, E.J., J.M. Geleijnse, and D. Kromhout, *Effects of n-3 fatty acids on depressive symptoms and dispositional optimism after myocardial infarction.* Am J Clin Nutr, 2011. **94**(6): p. 1442-50.
152. Bot, M., et al., *Supplementation with eicosapentaenoic omega-3 fatty acid does not influence serum brain-derived neurotrophic factor in diabetes mellitus patients with major depression: a randomized controlled pilot study.* Neuropsychobiology, 2011. **63**(4): p. 219-23.
153. Andreeva, V.A., et al., *Supplementation with B vitamins or n-3 fatty acids and depressive symptoms in cardiovascular disease survivors: ancillary findings from the SUpplementation with FOLate, vitamins B-6 and B-12 and/or OMega-3 fatty acids (SU.FOL.OM3) randomized trial.* Am J Clin Nutr, 2012. **96**(1): p. 208-14.
154. Fontani, G., et al., *Cognitive and physiological effects of Omega-3 polyunsaturated fatty acid supplementation in healthy subjects.* Eur J Clin Invest, 2005. **35**(11): p. 691-9.
155. van de Rest, O., et al., *Effect of fish-oil supplementation on mental well-being in older subjects: a randomized, double-blind, placebo-controlled trial.* Am J Clin Nutr, 2008. **88**(3): p. 706-13.
156. Antypa, N., et al., *Omega-3 fatty acids (fish-oil) and depression-related cognition in healthy volunteers.* J Psychopharmacol, 2009. **23**(7): p. 831-40.
157. Kiecolt-Glaser, J.K., et al., *Omega-3 supplementation lowers inflammation and anxiety in medical students: a randomized controlled trial.* Brain Behav Immun, 2011. **25**(8): p. 1725-34.
158. DeFina, L.F., et al., *Effects of omega-3 supplementation in combination with diet and exercise on weight loss and body composition.* Am J Clin Nutr, 2011. **93**(2): p. 455-62.
159. Kiecolt-Glaser, J.K., et al., *Omega-3 supplementation lowers inflammation in healthy middle-aged and older adults: a randomized controlled trial.* Brain Behav Immun, 2012. **26**(6): p. 988-95.
160. Parker, G., et al., *Omega-3 fatty acids and mood disorders.* Am J Psychiatry, 2006. **163**(6): p. 969-78.
161. Martins, J.G., *EPA but not DHA appears to be responsible for the efficacy of omega-3 long chain polyunsaturated fatty acid supplementation in depression: evidence from a meta-analysis of randomized controlled trials.* J Am Coll Nutr, 2009. **28**(5): p. 525-42.
162. Freeman, M.P., et al., *Complementary and alternative medicine in major depressive disorder: the American Psychiatric Association Task Force report.* J Clin Psychiatry, 2010. **71**(6): p. 669-81.
163. Sublette, M.E., et al., *Meta-analysis of the effects of eicosapentaenoic acid (EPA) in clinical trials in depression.* J Clin Psychiatry, 2011. **72**(12): p. 1577-84.
164. Bloch, M.H. and J. Hannestad, *Omega-3 fatty acids for the treatment of depression: systematic review and meta-analysis.* Mol Psychiatry, 2012. **17**(12): p. 1272-82.
165. Lin, P.Y., et al., *Are omega-3 fatty acids antidepressants or just mood-improving agents? The effect depends upon diagnosis, supplement preparation, and severity of depression.* Mol Psychiatry, 2012. **17**(12): p. 1161-3; author reply 1163-7.
166. Grosso, G., et al., *Role of omega-3 fatty acids in the treatment of depressive disorders: a comprehensive meta-analysis of randomized clinical trials.* PLoS One, 2014. **9**(5): p. e96905.
167. Nemets, H., et al., *Omega-3 treatment of childhood depression: a controlled, double-blind pilot study.* Am J Psychiatry, 2006. **163**(6): p. 1098-100.
168. Martins, J.G., H. Bentsen, and B.K. Puri, *Eicosapentaenoic acid appears to be the key omega-3 fatty acid component associated with efficacy in major depressive disorder: a critique of Bloch and Hannestad and updated meta-analysis.* Mol Psychiatry, 2012. **17**(12): p. 1144-9; discussion 1163-7.

169. Gharekhani, A., et al., *The effect of omega-3 fatty acids on depressive symptoms and inflammatory markers in maintenance hemodialysis patients: a randomized, placebo-controlled clinical trial.* Eur J Clin Pharmacol, 2014.
170. Su, K.P., et al., *Omega-3 Fatty Acids in the Prevention of Interferon-Alpha-Induced Depression: Results from a Randomized, Controlled Trial.* Biol Psychiatry, 2014.
171. Lang, U.E. and S. Borgwardt, *Molecular mechanisms of depression: perspectives on new treatment strategies.* Cell Physiol Biochem, 2013. **31**(6): p. 761-77.
172. Chiu, C.C., S.Y. Huang, and K.P. Su, *Omega-3 polyunsaturated fatty acids for postpartum depression.* Am J Obstet Gynecol, 2004. **190**(2): p. 582-3; author reply 583.
173. Chiu, C.C., et al., *Omega-3 fatty acids for depression in pregnancy.* Am J Psychiatry, 2003. **160**(2): p. 385.
174. McNamara, R.K., et al., *Lower docosahexaenoic acid concentrations in the postmortem prefrontal cortex of adult depressed suicide victims compared with controls without cardiovascular disease.* J Psychiatr Res, 2013. **47**(9): p. 1187-91.
175. Bloch, M.H. and A. Qawasmi, *Omega-3 fatty acid supplementation for the treatment of children with attention-deficit/hyperactivity disorder symptomatology: systematic review and meta-analysis.* J Am Acad Child Adolesc Psychiatry, 2011. **50**(10): p. 991-1000.
176. Sonuga-Barke, E.J., et al., *Nonpharmacological interventions for ADHD: systematic review and meta-analyses of randomized controlled trials of dietary and psychological treatments.* Am J Psychiatry, 2013. **170**(3): p. 275-89.
177. Gillies, D., et al., *Polyunsaturated fatty acids (PUFA) for attention deficit hyperactivity disorder (ADHD) in children and adolescents.* Cochrane Database Syst Rev, 2012. **7**: p. CD007986.
178. Richardson, A.J. and P. Montgomery, *The Oxford-Durham study: a randomized, controlled trial of dietary supplementation with fatty acids in children with developmental coordination disorder.* Pediatrics, 2005. **115**(5): p. 1360-6.
179. Berger, G.E., et al., *Ethyl-eicosapentaenoic acid in first-episode psychosis: a randomized, placebo-controlled trial.* J Clin Psychiatry, 2007. **68**(12): p. 1867-75.
180. Amminger, G.P., et al., *Long-chain omega-3 fatty acids for indicated prevention of psychotic disorders: a randomized, placebo-controlled trial.* Arch Gen Psychiatry, 2010. **67**(2): p. 146-54.
181. Markulev, C., et al., *NEURAPRO-E study protocol: a multicentre randomized controlled trial of omega-3 fatty acids and cognitive-behavioural case management for patients at ultra high risk of schizophrenia and other psychotic disorders.* Early Interv Psychiatry, 2015.
182. Fusar-Poli, P. and G. Berger, *Eicosapentaenoic acid interventions in schizophrenia: meta-analysis of randomized, placebo-controlled studies.* J Clin Psychopharmacol, 2012. **32**(2): p. 179-85.
183. Su, K.P., W.W. Shen, and S.Y. Huang, *Are omega3 fatty acids beneficial in depression but not mania?* Arch Gen Psychiatry, 2000. **57**(7): p. 716-7.
184. Bellino, S., et al., *Efficacy of omega-3 fatty acids in the treatment of borderline personality disorder: a study of the association with valproic acid.* J Psychopharmacol, 2014. **28**(2): p. 125-32.
185. Gesch, C.B., et al., *Influence of supplementary vitamins, minerals and essential fatty acids on the antisocial behaviour of young adult prisoners. Randomised, placebo-controlled trial.* Br J Psychiatry, 2002. **181**: p. 22-8.
186. Raine, A., et al., *Reduction in behavior problems with omega-3 supplementation in children aged 8-16 years: a randomized, double-blind, placebo-controlled, stratified, parallel-group trial.* J Child Psychol Psychiatry, 2015. **56**(5): p. 509-20.
187. Rice, S.M., et al., *Youth depression alleviation: the Fish Oil Youth Depression Study (YoDA-F): A randomized, double-blind, placebo-controlled treatment trial.* Early Interv Psychiatry, 2014.
188. Bridge, J.A., et al., *Placebo response in randomized controlled trials of antidepressants for pediatric major depressive disorder.* Am J Psychiatry, 2009. **166**(1): p. 42-9.
189. Parellada, M., et al., *Placebo effect in child and adolescent psychiatric trials.* Eur Neuropsychopharmacol, 2012. **22**(11): p. 787-99.
190. Schlogelhofer, M., et al., *Polyunsaturated fatty acids in emerging psychosis: a safer alternative?* Early Interv Psychiatry, 2014. **8**(3): p. 199-208.
191. Harris, W.S., T.D. Dayspring, and T.J. Moran, *Omega-3 fatty acids and cardiovascular disease: new developments and applications.* Postgrad Med, 2013. **125**(6): p. 100-13.
192. Gidding, S.S., et al., *A double-blind randomized trial of fish oil to lower triglycerides and improve cardiometabolic risk in adolescents.* J Pediatr, 2014. **165**(3): p. 497-503 e2.
193. de Ferranti, S.D., et al., *Using high-dose omega-3 fatty acid supplements to lower triglyceride levels in 10- to 19-year-olds.* Clin Pediatr (Phila), 2014. **53**(5): p. 428-38.

194. Bonaccio, M., et al., *Adherence to a Mediterranean diet is associated with a better health-related quality of life: a possible role of high dietary antioxidant content.* BMJ Open, 2013. **3**(8).
195. Valls-Pedret, C., et al., *Mediterranean Diet and Age-Related Cognitive Decline: A Randomized Clinical Trial.* JAMA Intern Med, 2015. **175**(7): p. 1094-103.
196. Corrigan, P.W., et al., *Challenging the public stigma of mental illness: a meta-analysis of outcome studies.* Psychiatr Serv, 2012. **63**(10): p. 963-73.
197. Henderson, C., et al., *Mental health-related stigma in health care and mental health-care settings.* Lancet Psychiatry, 2014. **1**(6): p. 467-82.
198. Henderson, C. and G. Thornicroft, *Stigma and discrimination in mental illness: Time to Change.* Lancet, 2009. **373**(9679): p. 1928-30.
199. Thornicroft, G., *Most people with mental illness are not treated.* Lancet, 2007. **370**(9590): p. 807-8.
200. Su, K.P., Y. Matsuoka, and C.U. Pae, *Omega-3 Polyunsaturated Fatty Acids in Prevention of Mood and Anxiety Disorders.* Clin Psychopharmacol Neurosci, 2015. **13**(2): p. 129-37.
201. Mischoulon, D., *Update and critique of natural remedies as antidepressant treatments.* Psychiatr Clin North Am, 2007. **30**(1): p. 51-68.
202. Simon, G.E., et al., *Mental health visits to complementary and alternative medicine providers.* Gen Hosp Psychiatry, 2004. **26**(3): p. 171-7.
203. Hibbeln, J.R., *Seafood consumption, the DHA content of mothers' milk and prevalence rates of postpartum depression: a cross-national, ecological analysis.* J Affect Disord, 2002. **69**(1-3): p. 15-29.
204. Hibbeln, J.R., *Depression, suicide and deficiencies of omega-3 essential fatty acids in modern diets.* World Rev Nutr Diet, 2009. **99**: p. 17-30.
205. Kiecolt-Glaser, J.K., H.M. Derry, and C.P. Fagundes, *Inflammation: Depression Fans the Flames and Feasts on the Heat.* Am J Psychiatry, 2015: p. appiajp201515020152.
206. Emslie, G.J., et al., *A double-blind, randomized, placebo-controlled trial of fluoxetine in children and adolescents with depression.* Arch Gen Psychiatry, 1997. **54**(11): p. 1031-7.
207. Harris, W.S., *The omega-3 index: from biomarker to risk marker to risk factor.* Curr Atheroscler Rep, 2009. **11**(6): p. 411-7.
208. Harris, W.S. and C. Von Schacky, *The Omega-3 Index: a new risk factor for death from coronary heart disease?* Prev Med, 2004. **39**(1): p. 212-20.
209. Beier, A.M., et al., *Low plasma eicosapentaenoic acid levels are associated with elevated trait aggression and impulsivity in major depressive disorder with a history of comorbid substance use disorder.* J Psychiatr Res, 2014. **57**: p. 133-40.
210. Wagner, S., et al., *A meta-analysis of cognitive functions in children and adolescents with major depressive disorder.* Eur Child Adolesc Psychiatry, 2015. **24**(1): p. 5-19.
211. Vilgis, V., T.J. Silk, and A. Vance, *Executive function and attention in children and adolescents with depressive disorders: a systematic review.* Eur Child Adolesc Psychiatry, 2015. **24**(4): p. 365-84.
212. McIntyre, R.S., *Major depressive disorder and cognitive impairment.* J Psychiatry Neurosci, 2014. **39**(5): p. E36-7.
213. Emslie, G.J., et al., *Fluoxetine for acute treatment of depression in children and adolescents: a placebo-controlled, randomized clinical trial.* J Am Acad Child Adolesc Psychiatry, 2002. **41**(10): p. 1205-15.
214. Emslie, G.J., N.D. Ryan, and K.D. Wagner, *Major depressive disorder in children and adolescents: clinical trial design and antidepressant efficacy.* J Clin Psychiatry, 2005. **66 Suppl 7**: p. 14-20.
215. Kovacs, M., *The Emanuel Miller Memorial Lecture 1994. Depressive disorders in childhood: an impressionistic landscape.* J Child Psychol Psychiatry, 1997. **38**(3): p. 287-98.
216. Ghasemifard, S., et al., *What Is the Most Effective Way of Increasing the Bioavailability of Dietary Long Chain Omega-3 Fatty Acids--Daily vs. Weekly Administration of Fish Oil?* Nutrients, 2015. **7**(7): p. 5628-45.
217. Poznanski, E.O., S.C. Cook, and B.J. Carroll, *A depression rating scale for children.* Pediatrics, 1979. **64**(4): p. 442-50.
218. Keller, F., et al., *[Children's Depression Rating Scale-Revised (CDRS-R): development of a German version and psychometric properties in a clinical sample].* Z Kinder Jugendpsychiatr Psychother, 2011. **39**(3): p. 179-85.
219. Plener, P.L., et al., *Convergence of Children's Depression Rating Scale-revised Scores and Clinical Diagnosis in Rating Adolescent Depressive Symptomatology.* Ment Illn, 2012. **4**(1): p. e7.

220. Kaufman, J., et al., *Schedule for Affective Disorders and Schizophrenia for School-Age Children-Present and Lifetime Version (K-SADS-PL): initial reliability and validity data.* J Am Acad Child Adolesc Psychiatry, 1997. **36**(7): p. 980-8.
221. Guy , W., *Assessment Manual for Psychopharmacology —Revised* 1976, NIMH Psychopharmacology Research Branch, Division of Extramural Research Programs: Rockville, MD, U.S. . p. 218–222.
222. Shaffer, D., et al., *A children's global assessment scale (CGAS).* Arch Gen Psychiatry, 1983. **40**(11): p. 1228-31.
223. Gowers, S.G., et al., *Brief scale for measuring the outcomes of emotional and behavioural disorders in children. Health of the Nation Outcome Scales for children and Adolescents (HoNOSCA).* Br J Psychiatry, 1999. **174**: p. 413-6.
224. Stiensmeier-Pelster, J., M. Schürmann, and K. Duda, *Depressions-Inventar für Kinder und Jugendliche (DIKJ). Handandweisung (2., überarb. und neunormierte Auflage).* 2000, Göttingen: Hogrefe.
225. Leyfer, O.T., J.L. Ruberg, and J. Woodruff-Borden, *Examination of the utility of the Beck Anxiety Inventory and its factors as a screener for anxiety disorders.* J Anxiety Disord, 2006. **20**(4): p. 444-58.
226. Beck, A.T., *Hopelessness as a predictor of eventual suicide.* Ann N Y Acad Sci, 1986. **487**: p. 90-6.
227. Beck, A.T., et al., *The measurement of pessimism: the hopelessness scale.* J Consult Clin Psychol, 1974. **42**(6): p. 861-5.
228. Becker, D.F. and C.M. Grilo, *Prediction of suicidality and violence in hospitalized adolescents: comparisons by sex.* Can J Psychiatry, 2007. **52**(9): p. 572-80.
229. Steer, R.A., G. Kumar, and A.T. Beck, *Self-reported suicidal ideation in adolescent psychiatric inpatients.* J Consult Clin Psychol, 1993. **61**(6): p. 1096-9.
230. Reynolds, W.M. and J.J. Mazza, *Assessment of Suicidal Ideation in Inner-City Children and Young Adolescents: Reliability and Validity of the Suicide Ideation Questionnaire.* School Psychology Review, 1999. **28**(1): p. 17-30.
231. Mazza, V.J. and W.M. Reynolds, *An investigation of psychopathology in nonreferred suicidal and nonsuicidal adolescents.* Suicide Life Threat Behav, 2001. **31**(3): p. 282-302.
232. Reynolds, W.M., *Psychometric characteristics of the Adult Suicidal Ideation Questionnaire in college students.* J Pers Assess, 1991. **56**(2): p. 289-307.
233. Gerber, M., et al., *Validation of the German version of the insomnia severity index in adolescents, young adults and adult workers: results from three cross-sectional studies.* Bmc Psychiatry, 2016. **16**.
234. Bastien, C.H., A. Vallieres, and C.M. Morin, *Validation of the Insomnia Severity Index as an outcome measure for insomnia research.* Sleep Medicine, 2001. **2**(4): p. 297-307.
235. Cohen, S., T. Kamarck, and R. Mermelstein, *A global measure of perceived stress.* J Health Soc Behav, 1983. **24**(4): p. 385-96.
236. Vaishnavi, S., K. Connor, and J.R. Davidson, *An abbreviated version of the Connor-Davidson Resilience Scale (CD-RISC), the CD-RISC2: psychometric properties and applications in psychopharmacological trials.* Psychiatry Res, 2007. **152**(2-3): p. 293-7.
237. Connor, K.M. and J.R. Davidson, *Development of a new resilience scale: the Connor-Davidson Resilience Scale (CD-RISC).* Depress Anxiety, 2003. **18**(2): p. 76-82.
238. Schafer, J., et al., *Is trait resilience characterized by specific patterns of attentional bias to emotional stimuli and attentional control?* J Behav Ther Exp Psychiatry, 2015. **48**: p. 133-9.
239. Ravens-Sieberer, U., et al., *The KIDSCREEN-27 quality of life measure for children and adolescents: psychometric results from a cross-cultural survey in 13 European countries.* Qual Life Res, 2007. **16**(8): p. 1347-56.
240. Witt, O., et al., *[Adult ADHD versus borderline personality disorder: criteria for differential diagnosis].* Fortschr Neurol Psychiatr, 2014. **82**(6): p. 337-45.
241. Helmstaedter, C., M. Lendt, and S. Lux, *VLMT Verbaler Lern- und Merkfähigkeitstest*. 2001, Göttingen: Hogrefe
242. Aschenbrenner, S., O. Tucha, and K.W. Lange, *RWFT Regensburger Wortflüssigkeits-Test*. 2000, Göttingen: Hogrefe.
243. De Sonneville, L., *Amsterdam Neuropsychological Tasks (ANT)*. 2011, Amsterdam: Sonares.
244. Wechsler, D., F. Petermann, and U. Petermann, *Wechsler Intelligence Scale for Children - Fourth Edition. WISC-IV.* 2014, Frankfurt: Pearson.

245. Drechsler, R. and H.-C. Steinhausen, *Verhaltensinventar zur Beurteilung exekutiver Funktionen BRIEF. Deutschsprachige Adaptation des Behavior Rating Inventory of Executive Function (BRIEF) von G. A. Gioia, P. K. Isquith, S. C. Guy und L. Kenworthy undder Self-Report Version (BRIEF-SR) von S. C. Guy, P. K. Isquith und G. A. Gioia.* . 2013, Bern: Huber.
246. Hagmann-von Arx, P. and G. A., *Reynolds Intellectual Assessment Scales and Screening. Deutschsprachige Adaptation der Reynolds Intellectual Assessment Scales (RIAS) & des Reynolds Intellectual Screening Test (RIST) von Cecil R. Reynoldsund Randy W. Kamphaus.* 2014, Bern: Huber.
247. Marshall, W.A. and J.M. Tanner, *Variations in pattern of pubertal changes in girls.* Arch Dis Child, 1969. **44**(235): p. 291-303.
248. Sublette, M.E., et al., *Validation of a food frequency questionnaire to assess intake of n-3 polyunsaturated fatty acids in subjects with and without major depressive disorder.* J Am Diet Assoc, 2011. **111**(1): p. 117-123 e1-2.
249. Newcombe, D.A., R.E. Humeniuk, and R. Ali, *Validation of the World Health Organization Alcohol, Smoking and Substance Involvement Screening Test (ASSIST): report of results from the Australian site.* Drug Alcohol Rev, 2005. **24**(3): p. 217-26.
250. Uher, R., et al., *Adverse reactions to antidepressants.* Br J Psychiatry, 2009. **195**(3): p. 202-10.
251. Thompson, K., J. Kulkarni, and A.A. Sergejew, *Reliability and validity of a new Medication Adherence Rating Scale (MARS) for the psychoses.* Schizophr Res, 2000. **42**(3): p. 241-7.
252. Mahler, C., et al., *Assessing reported adherence to pharmacological treatment recommendations. Translation and evaluation of the Medication Adherence Report Scale (MARS) in Germany.* J Eval Clin Pract, 2010. **16**(3): p. 574-9.
253. March, J., et al., *Fluoxetine, cognitive-behavioral therapy, and their combination for adolescents with depression: Treatment for Adolescents With Depression Study (TADS) randomized controlled trial.* JAMA, 2004. **292**(7): p. 807-20.
254. March, J., et al., *The Treatment for Adolescents with Depression Study (TADS): methods and message at 12 weeks.* J Am Acad Child Adolesc Psychiatry, 2006. **45**(12): p. 1393-403.
255. Rubin, D.B., *Multiple imputation after 18+ years.* Journal of the American Statistical Association, 1996. **91**(434): p. 473-489.
256. Rubin, D.B., *Multiple imputation for nonresponse in surveys*. 1987, New York: John Wiley & Sons.

Epidemiology, Biostatistics
and Prevention Institute,
Biostatistics Department
+41 44 634 46 41
www.biostat.uzh.ch

Omega-3 Fatty Acids as First-Line Treatment in Pediatric Depression

Statistical Analysis Plan for Gregor Berger, Isabelle Häberling

Kelly Reeve (*kelly.reeve@uzh.ch*)
Ulrike Held

Version 1.0 of May 31, 2022

List of abbreviations

AD	antidepressant
AE	adverse event
CDRS-R	Children's Depression Rating Scale - Revised
DIKJ	Children's Depression Inventory (Depressionsinventar für Kinder und Jugendliche)
EPA	eicosapentaenoic acid
KIDscreen-CAT-27	quality of life questionnaire
pMDD	pediatric major depressive disorder
SIQ-Jr	Suicide Ideation Questionnaire-Junior
SUSAR	Suspected Unexpected Serious Adverse Reaction

Introduction

1 Type of trial

This is a 36-week multi-center 1:1 randomized, double-blind, placebo-controlled, two-armed, parallel group study investigating the superiority of EPA-rich Omega-3 to placebo in the treatment of children and adolescents with pediatric major depressive disorder. The randomization included a minimization algorithm based on the following factors: site, age (8-12 vs. 13-18), gender (male vs. female), and hsCRP (<1 mg/l; 1-3 mg/l; >3mg/l). For sites offering both in- and out-patient care, in-/out-patient type was used as an additional minimization factor. The trial aimed to recruit children/teenagers between 8 and 18 years old with a primary diagnosis of major depressive disorder (DSM-IV, K-SADS-PL) and at least moderate depressive symptoms (CDRS-R ≥ 40).

In order to include a representative sample of moderately and severely ill children with major depressive disorders, the use of clinically required SSRIs is permitted. The anticipated proportion of patients receiving additional antidepressants at some point during follow-up is estimated to be around 30%. The use of additional antidepressants is expected to influence primary outcome measurements and be influenced by treatment group, with greater antidepressant use hypothesized in the control arm. Antidepressant use may therefore bias the estimation of the omega-3 treatment effect.

2 Scope of the statistical analysis plan

This statistical analysis plan (SAP) is based on the protocol for the trial SNF 33IC30 166826 ”Omega3 Fatty Acids as First-Line Treatment in Paediatric Depression” version 6 dated 09.03.2021. Its scope is limited to the primary trial objectives, namely efficacy and safety. This SAP includes further details regarding the statistical analysis of the primary and secondary efficacy and safety endpoints to be conducted after closure of the database. Specifically, the following changes were introduced:

- A single primary outcome, longitudinal CDRS-R total score, was selected;
- Remission and response were changed from additional primary outcomes to confirmatory secondary outcomes, while recovery was removed as redundant;
- The scope of the primary trial analysis was reduced, reserving exploration of the relationships between inflammatory and lipid markers and symptoms and treatment response for follow-up analyses described in future analysis plans and manuscripts;
- Further details for statistical analyses per outcome were specified;
- A method assuming MNAR was explicitly specified for the primary analysis of the primary outcome and sensitivity analyses exploring robustness to the selected assumptions were specified.

Changes to the statistical analyses made across protocol versions are documented in the final section of this document.

3 Objectives

The study protocol defines the following study objectives:

1. To investigate the therapeutic efficacy and safety of omega-3 fatty acids rich in EPA in pMDD;
2. to demonstrate clinically meaningful effects of omega-3 fatty acid treatment;
3. to investigate inflammatory and bioactive lipid markers as response predictors;
4. to investigate the relationship between psychopathology (in particular suicidal ideation), illness course and cognition in relation to inflammatory and bioactive lipid markers.

For the purpose of this statistical analysis plan, we focus on the primary trial objectives, i.e., efficacy and safety objectives, as described below.

3.1 Primary objective

- To demonstrate the superiority of daily EPA-rich Omega-3 over placebo as an adjunct to the standard of care (German S3 guidelines) in children and adolescents with pediatric major depressive disorder.

3.2 Secondary objectives

- To demonstrate the clinically meaningful efficacy of daily EPA-rich Omega-3 relative to placebo as an adjunct to the standard of care (German S3 guidelines) in children and adolescents with pediatric major depressive disorder.

- To compare the safety and tolerability of daily EPA-rich Omega-3 versus placebo as an adjunct to the standard of care (German S3 guidelines) in children and adolescents with pediatric major depressive disorder.

4 Estimands

4.1 Primary estimand

Effectiveness (i.e., 'de facto', 'treatment policy') estimand The effectiveness estimand quantifies the average treatment effect of EPA-rich Omega-3 relative to placebo over 36 weeks, as an adjunct to the German S3 guideline standard of care, in all randomized subjects regardless of adherence to treatment or initiation of antidepressants.

4.2 Secondary estimand

Efficacy (i.e., 'du jure', 'hypoethetical') estimand The efficacy estimand quantifies the average treatment effect of EPA-rich Omega-3 relative to placebo over 36 weeks, as an adjunct to the German S3 guideline standard of care, if all randomized subjects had adhered to the randomly assigned treatment for the entire trial duration.

5 Endpoints

5.1 Primary endpoints

- CDRS-R total score trajectories from baseline to week 36 (measured at baseline, 6, 12, 24, and 36 weeks)

5.2 Secondary endpoints

5.2.1 Confirmatory secondary endpoints

- Remission (CDRS-R total score less than or equal to 28) at any point during the 36 weeks of trial observation
- Response (30% reduction in CDRS-R total score) at any point within 12 weeks from baseline
- Participants newly initiating antidepressant medication (or newly increasing dose if on antidepressant at baseline) at any point during the 36 weeks of trial observation
- Number of days on trial before new antidepressant initiation (or new antidepressant dose increase if on antidepressant at baseline)
- KIDscreen-CAT-27 overall quality of life score at 36 weeks
- DIKJ depression score at 36 weeks

5.2.2 Supportive secondary endpoints

Effect endpoints

- Number of days in hospital
- Lower outpatient service use

Safety endpoints

- Suicidal ideation (SIQ-Jr) from baseline to week 36 (measured at baseline, 6, 12, 24, and 36 weeks)
- Number of SUSARs from baseline to week 36

Statistical considerations

6 Sample size determination

The sample size calculations were performed for the primary outcome, as well as closely related secondary outcomes in order to ensure all questions of high interest are sufficiently powered. All calculations assume a parallel two-arm design with a 1:1 randomization ratio.

6.1 CDRS-R Difference

Meta-analyses selecting studies of omega-3 fatty acids with high proportion of EPA in aMDD as primary diagnosis found an SMD between 0.28-0.56 (see Protocol references 160-166). However, no studies were performed in pMDD, except for one very small (n=20) pilot study in childhood depression with a large effect size (SMD=1.2) (see Protocol reference 167).

A two-sample t-test (equal variances) would require 74 participants per treatment arm to have 90% power to detect an optimistic standardized mean difference of 0.54 on the CDRS-R scale at the 0.05 two-sided significance level. The trial would then require a total of 148 participants.

As the placebo response rate in minors is probably higher compared to that reported in the single center RCTs in adults summarized in the meta-analyses referenced above, we calculated our sample size estimation under more conservative assumptions as well.

The sample size calculation above for testing a difference in CDRS-R scores considered a relatively optimistic effect size and did not account for loss to follow-up. A two-sample t-test (equal variances) would require 111 participants per treatment arm to have 80% power to detect a smaller standardized mean difference of 0.4 on the CDRS-R scale at the 0.05 two-sided significance level. The total sample size needed would then be 222 participants.

6.2 Difference in Proportion of Responders

A two-proportion z-test would require 97 participants per treatment arm to have 80% power to detect a difference in response of 20% (with 60% response in the treatment group and 40% response in the control group) at the 0.05 two-sided significance level. Accounting for a 10% loss to follow-up results in a total sample size of 216 participants.

7 Statistical analyses

Analysis sets The following analysis sets are defined in accordance with the ICH-E9 guidelines:

- The full analysis set (FAS) includes all randomized subjects. The FAS will be used for 'as randomized' evaluation. Only in exceptional cases may subjects be excluded from the FAS. In such cases the reason for exclusion will be justified and documented.

- The safety analysis set includes all randomized subjects receiving at least one dose of randomized treatment. The safety analysis set will be used for 'as treated' evaluation.

Baseline comparability of randomized groups Baseline descriptive variables of participants (including antidepressant use) will be summarized overall and by treatment arm using suitable measures of central tendencies and variability for continuous data (means or medians, SD or IQR) and frequencies and proportions for categorical data. No hypotheses are tested.

The number of participants lost to follow-up, initiating antidepressants, and adhering to the protocol will each be tabulated by treatment arm and visit. Baseline characteristics will also be summarized across the groups of participants completing the trial, lost to follow-up, and initiating antidepressant use.

General statistical considerations Statistical analysis results will generally be presented as point estimates with two-sided 95% confidence intervals. A limited number of analysis results will additionally be reported with *p*-values, which will be interpreted as indicative of superiority when the value is less than 5% and the estimated treatment contrast favors the omega-3 treatment arm.

Descriptive statistics for all efficacy and safety endpoints will be presented according to treatment arm.

The baseline values are collected at visit 1 (0 weeks), which is the first week after the lead-in phase. Further measurements are taken at visits 2 through 5 at 6, 12, 24, and 36 weeks.

The clinical trials registry number (03167307) will be used as a seed number, if needed.

Missing data By design, several baseline model covariates cannot be missing, as they are required for minimization during randomization. We distinguish between study dropouts and intervention dropouts. Study dropouts are participants whom withdraw consent, are monotone not available for follow-up visits, or are removed from the trial according to the protocol due to non-compliance (< 60% pills taken). Intervention dropouts are participants whom newly initiate an off-trial antidepressant or are newly prescribed an increased dose of an off-trial antidepressant prescribed before baseline.

Methods to handle missing data are outlined per analysis type below. Where multiple is used, 50 imputed datasets will computed. Results across datasets will be combined using Rubin's rule.

Modeling In case of covariate imbalance on baseline covariates other than antidepressant use, omega-3 index, age, gender, and hsCRP, these covariates will also be included in the models described below. In case of computational convergence issues, simpler models will be estimated.

7.1 Primary endpoint

- CDRS-R total score from baseline (post-placebo-lead-in) to week 36

The primary analysis consists of a joint model linking (1) a linear mixed effects model for CDRSR total score over time with (2) a survival model for the risk of the event new initiation of or increase in antidepressant use (intervention dropout) and (3) a survival model for the risk of study dropout as a competing event. This assumes that whether a participant has missing data and at what time this occurs contains additional information that should be taken into account and corresponds to an MNAR missing data mechanism.

Participants are censored at whichever occurs first of: antidepressant initiation/increase, loss to follow up, consent withdrawal, and study exit due to non-adherence. Participants lost to follow up, withdrawing consent, and exiting the study (as per protocol) due to non-adherence are considered alike and these events considered one event type: study dropout.

The linear mixed effects model for the longitudinal CDRS-R total score outcome $Y_i(t)$ measured at time t for participant i takes the following form:

$$Y_i(t) = m_i(t)+\varepsilon_i(t),\ m_i(t) = \beta_0 + \beta_1 \text{intervention}_i + \beta_2 t_{base} + \beta_3 t_{6weeks} + \beta_4 t_{12weeks} + \beta_5 t_{24weeks}$$

$$+\beta_6 t_{36weeks}$$

$$+\beta_7 t_{base} * \text{intervention}_i + \beta_8 t_{6weeks} * \text{intervention}_i + \beta_9 t_{12weeks} * \text{intervention}_i$$

$$+\beta_{10} t_{24weeks} * \text{intervention}_i + \beta_{11} t_{36weeks} * \text{intervention}_i$$

$$+\beta_{12}\text{age}_i + \beta_{13}\text{gender}_i + \beta_{14}\text{hsCRP}_i$$

$$+\beta_{15}\text{antidepressant} + \beta_{16}\text{omega-3 index}$$

$$+ b_{i0} + b_{i1}t + b_{s0},$$

where time t is considered a factor (while also investigating possibility of linearizing time effect) and randomized treatment group (intervention) and the interaction between randomized treatment group and time are included as fixed effects. The baseline values of the minimization factors age, gender, and hsCRP, as well as baseline antidepressant use and omega-3 index are included as covariates. The baseline outcome measure is modeled in the response, not as a covariate. The terms b_{i0}, b_{i1}, and b_{s0} represent participant-specific random intercepts and slopes and site-specific random intercepts. The random effects are assumed to be normally distributed and an unstructured covariance matrix will be assumed. The error term $\varepsilon_i(t)$ is assumed to be normally distributed with mean zero and variance σ^2, independent of the random effects and is independent over time. An overall treatment effect for CDRS-R score at 36 weeks ($\beta_1 + \beta_{11}$) will be reported with a 95% confidence interval and corresponding p-value.

The competing survival models for time to event r (intervention dropout or study dropout) take the form:

$$\lambda_{ir}(t) = \lambda_{0r}\exp\{\gamma_1 \text{intervention} + \alpha_r m_i(t)\}$$

where the cause-specific survival models include intervention as a time-independent effect and the estimated true trajectory of CDRS-R as a time-varying effect. The parameters α_r measure the strength of association between the longitudinal outcome and risk of the corresponding dropout event.

7.1.1 Subgroup analysis for the primary endpoint

The consistency of the treatment effect will be evaluated by adding an interaction term between treatment effect, time, and omega-3 index to the model described above. The p-value for the comparison between the interaction model and the simpler model will be used to determine consistency.

7.1.2 Sensitivity analyses for the primary endpoint

1. **Typical ITT analysis**: In order to compare results with other trials, the linear mixed model part of the analysis model above will be applied to the full analysis set, with all participants analyzed as randomized. The data from intervention dropout participants will be used as observed, i.e, the outcomes observed while on non-randomized antidepressants will be used. The mixed model assumes that missing data is missing at random. This analysis is expected to be biased toward the placebo arm,

as the number of participants requiring antidepressants is expected to be higher in the placebo arm and the additional antidepressant will improve outcomes.

2. **Modified ITT analysis**: The first sensitivity analysis will be repeated, but the data from intervention dropout participants will be considered missing in the mixed model upon nonrandomized antidepressant initiation/dose increase. The responses of participants with missing data will be assumed similar to those with complete data and similar covariate characteristics. The bias resulting from imbalance in the use of non-randomized antidepressants across treatment groups is curtailed, but the amount of data available for analysis, and therefore power, is diminished.

3. **Typical PP analysis**: In order to address the efficacy estimand, the sensitivity analysis model above will be applied to all full analysis set data during which participants were 'on protocol'. This means the participants are adhering to the treatment they were randomized to and are not using additional antidepressants. Non-compliers and those taking non-randomized antidepressants are excluded completely (i.e., complete case analysis). This yields an estimate of efficacy that is unbiased under the MAR assumption. It assumes that participants with missing data (those with additional antidepressants and those not adhering to randomized treatment) would have had similar outcomes to participants completing the study in the same treatment arm and with similar baseline characteristics and score trajectories. However, the use of additional anti-depressants is not considered to be random across participants, but rather related to intermediate outcomes and possibly treatment assignment. Selection bias is then expected to affect this estimate. Additionally, the reduction in participants decreases the power to detect a treatment effect.

4. **Delta-based multiple imputation for MNAR analysis**: The data after intervention dropout or study dropout is considered to be missing. Multiple imputation under MAR will be performed separately within each treatment arm (White et al 2011) using imputation models containing the minimization factors, baseline antidepressant use and omega-3 index. It will be assumed that participants initiating/increasing non-randomized antidepressant use would have had worse outcomes than observed had they not used the non-randomized antidepressant. A range of mean differences δ from a 25% increase in CDRS-R score (25% worse outcome) to a 100% increase (by 25% increments) in the CDRS-R score in the intervention dropouts as compared to the observed cases will be considered. Delta will then be added to the imputed CDRS-R scores for the participants with intervention dropout. Results will be combined using Rubin's rule. This is repeated for each delta.

7.2 Secondary endpoints

7.2.1 Confirmatory secondary endpoints

The binary confirmatory secondary outcomes include:

- **Remission**, defined as a CDRS-R total score < 28 at any time up until 36 weeks
- **Response**, defined as a 30% drop in CDRS-R total score at any time up until **12 weeks**
- **Intervention dropout**, defined as initiation of non-randomized antidepressant use while on trial for participants not on a non-randomized antidepressant at baseline or an increase in antidepressant dose while on trial for participants on a non-randomized antidepressant at baseline

Unadjusted proportions per treatment arm will be presented with 95% confidence intervals. To adjust for baseline antidepressant use, omega-3 index, and CDRS score, as well as the minimization factors age, gender, and hsCRP, these binary outcomes will each be assessed using log-binomial models with treatment arm as the effect of interest. Each model will be interpreted in terms of the the adjusted risk ratio (RR) comparing the outcome probability in the omega-3 group to that in the placebo group ($RR_{\text{omega-3/placebo}}$). The null hypothesis that the risk ratio is equal to 1 (no difference in risk across treatment groups) will be rejected if the 95% confidence interval for the risk ratio does not include 1. If the 95% confidence interval for the risk ratio is above 1, omega-3 treatment will be considered superior.

These models will include data from all randomized participants and analyze the participant data according to the treatment arms participants were randomized to (as-randomized). Data after intervention dropout will be considered missing. Multiple imputation under MAR will be used to impute longitudinal CDRS-R scores for study and intervention dropouts before the binary outcomes are determined. MI will be performed separately within each treatment arm (White et al 2011) using imputation models containing the minimization factors, baseline antidepressant use and omega-3 index.

As for the primary outcome, a delta-based sensitivity analysis approach will be used to address MNAR. For analysis of intervention dropout, imputation is only needed for participants with study dropout before intervention dropout and no sensitivity analysis is planned.

The continuous confirmatory secondary outcomes include:

- self-reported **quality of life** measured by the KIDscreen-CAT-27 overall score at 36 weeks
- **Depression severity** measured by DIKJ at 36 weeks

These continuous outcomes will each be assessed using ANCOVA linear regression models with the 36-week measurement as the outcome, treatment arm as the effect of interest, and the baseline measurement, antidepressant use, omega-3 index, CDRS-R score, and the minimization factors age, gender, and hsCRP as covariates.

The treatment coefficient denotes the mean difference in outcome score across the treatment groups. The null hypothesis that the mean scores are equal across treatment groups will be rejected if the 95% confidence interval for the treatment group coefficient does not include 0. If the 95% confidence interval for the treatment group coefficient is above 0 for KIDscreen-CAT-27 (below 0 for DIKJ), omega-3 treatment will be considered superior with respect to its effect on quality of life (depressive symptoms).

These models will include data from all randomized participants and analyze the participant data according to the treatment arms participants were randomized to (as-randomized). Data after intervention dropout will be considered missing. Multiple imputation under MAR will be used to impute scores at 36 weeks for study and intervention dropouts. MI will be performed separately within each treatment arm (White et al 2011) using imputation models containing the minimization factors, baseline antidepressant use, omega-3 index, and baseline score. As for the primary outcome, a delta-based sensitivity analysis approach will be used to address MNAR. Sub-domains will be described with tables or figures.

The final confirmatory secondary outcome is:

- the time on trial before initiation of new or increased antidepressant use (intervention dropout).

A Kaplan Meier approach will be used to estimate unadjusted median time to this event overall and per treatment arm. A log rank test will be used to compare time to intervention dropout across treatment arms.

7.2.2 Supportive secondary endpoints

Effect endpoints

- **Number of days in the hospital**
- **Outpatient service use**

These endpoints will be described using summary statistics across treatment arm.

Safety endpoints

- **Suicidal ideation** from baseline to week 36 will be modeled jointly by a linear mixed effects model for SIQ-Jr score over time combined with survival models for time to intervention and study dropout, analogously to the CDRS-R total score primary outcome model.
- **Serious adverse events** from baseline to week 36 will be tabulated per type and treatment arm using the safety analysis set. If appropriate, the difference in proportion (with 95% confidence interval) will be estimated.

8 Changes to the statistical analyses planned in the protocol

Version	Explanation
IICT submission	First version of study plan
Frontiers protocol	Study protocol after funding review and peer review, addition of concomitant treatment description, addition minimization factor for unit type (in-/outpatient), description of sensitivity analyses to account for antidepressant use
UZH protocol	Refinement of secondary (supportive/exploratory) outcomes and their analyses
Statistical analysis plan	Focusing the analysis plan on measures of efficacy and safety for reporting of primary trial results. Updating method for dealing with informative missingness.

9 References

N. M. Laird and J. H. Ware. Random-effects models for longitudinal data. Biometrics 1982; 38:963– 74

R J A Little and L Yau. Intent-to-treat analysis for longitudinal studies with drop-outs. Biomet-rics 19996; 52:1324–33.

S Mazumdar and K S Liu and P R Houck and C F Reynolds. Intent-to-treat analysis for longi-tudinal clinical trials: coping with the challenge of missing values. Journal of Pyschatric Research 1999; 33:87–95

Michele L Shaffer and Vernon M Chinchilli. Including multiple imputation in a sensitivity analysis for clinical trials with treatment failures. Contemporary Clinical Trials 2007 28:130–137

Ian R. White and C Bamias and P Hardy and S. Pocock and J Warner. Randomized clinical trials with added rescue medication: some approaches to their analysis and interpretation. Statistics in Medicine 2001; 20:2995–3008.

R **version and packages used to generate this report:**

R version: R version 4.0.1 (2020-06-06)

Base packages: stats, graphics, grDevices, utils, datasets, methods, base Other packages: pwr, readxl, reporttools, xtable, knitr This document was generated on May 31, 2022 at 16:02.

University of Zurich[UZH]

Epidemiology, Biostatistics
and Prevention Institute,
Biostatistics Department
www.biostat.uzh.ch

Omega-3 Fatty Acids as First-Line Treatment in Pediatric Depression: A Randomized Placebo-Controlled Trial

Analysis for Gregor Berger, Isabelle Häberling

Kelly Reeve, Priska Heinz (*priska.heinz@uzh.ch*)
Ulrike Held (*ulrike.held@uzh.ch*)

Version 4.0 of October 21, 2024

DISCLAIMER

This report was written by the Research Methods Consulting Unit of the University of Zurich, Epidemiology, Biostatistics and Prevention Institute, Biostatistics Department (EBPI). Any citation of results or comments (or parts thereof) given in this report in any format (including: paper, presentation, abstract, grant application) or the naming of EBPI or persons of the EBPI requires the explicit approval of the senior statistician (Ulrike Held). This is especially true for citations in the "acknowledgments".

1 List of abbreviations

AD	antidepressant
AE	adverse event
CDRS-R	Children's Depression Rating Scale - Revised
DIKJ	Children's Depression Inventory
DSM-IV	basis for diagnosis of major depression
EPA	eicosapentaenoic acid
K-SADS-PL	basis for diagnosis of major depression
KIDscreen-CAT-27	quality of life questionnaire
pMDD	pediatric major depressive disorder
SIQ-Jr	Suicide Ideation Questionnaire-Junior
SSRI	antidepressants
SUSAR	Suspected Unexpected Serious Adverse Reaction

2 Background

This is a 36-week multi-center 1:1 randomized, double-blind, placebo-controlled, two-armed, parallel group study investigating the superiority of EPA-rich Omega-3 to placebo in the treatment of children and adolescents with pediatric major depressive disorder. The randomization included a minimization algorithm based on the following factors: site, age (8-12 vs. 13-18), gender (male vs. female), and hsCRP (<1 mg/l; 1-3 mg/l; >3mg/l). For sites offering both in- and out-patient care, in-/out-patient type was used as an additional minimization factor. The trial aimed to recruit

children/ teenagers between 8 and 18 years old with a primary diagnosis of major depressive disorder (DSM-IV, K-SADS-PL) and at least moderate depressive symptoms (CDRS-R $\geq$ 40). In order to include a representative sample of moderately and severely ill children with major depressive disorders, the use of clinically required SSRIs at baseline is permitted. The anticipated proportion of patients receiving additional antidepressants at some point during follow-up is estimated to be around 30%. The use of additional antidepressants is expected to influence primary outcome measurements and be influenced by treatment group, with greater antidepressant use hypothesized in the control arm. Antidepressant use may therefore bias the estimation of the omega-3 treatment effect.

3 Statistical Methods

All details of the analyses can be found in the statistical analysis plan (Version 1.0 of May 31, 2022).

Sample size A two-sample t-test (equal variances) would require 111 participants per treatment arm (222 participants in total) to have 80% power to detect a standardized mean difference of 0.4 on the CDRS-R scale at the 0.05 two-sided significance level and allowing for 10% drop out. The effect size was assumed based on meta-analyses of Omega-3 treatment for adult major depressive disorder, which reported an SMD between 0.28 and 0.56 (see Protocol references 160-166).

Statistical Methods All analyses included all randomized patients in the treatment groups to which they were assigned. As per protocol, observations after loss to follow up or non-compliance (study dropouts) and observations after new initiation or dose increase of an off-trial antidepressant (intervention dropouts) were considered missing and addressed in modeling or via multiple imputation, as explained per analysis below.

The primary outcome CDRS-R score was analyzed using a joint model linking a linear mixed effects model for the score over time with a semi-parametric time-to-event model (penalized B-splines for modeling the baseline hazard function) for the risk of study or intervention dropout. It was assumed that whether a patient had missing data and at what time this occurred contained additional information that should be taken into account, corresponding to an MNAR missing data mechanism. The longitudinal model included fixed effects for treatment, time (weeks since baseline), and a treatment-time interaction, as well as the baseline covariates age, sex, hsCRP, Omega-3 index, additional antidepressant use and random intercepts and slopes for patient. Baseline covariates with apparent imbalance across treatment groups were also included as fixed effects. Treatment group and the current fitted value of the longitudinal trajectory were included as covariates in the time-to-event model. The differences in treatment arm-specific CDRS-R rates of change were examined for the primary analysis. This analysis was repeated for the suicidal ideation (SIQ-Jr) safety outcome.

Further analyses were conducted to examine the sensitivity of results to the modeling choice. These included linear mixed models applied to the data as observed (no censoring of observations after study or intervention dropout), to the censored data, to complete-case data, and to multiply imputed data. Multiple imputation under MAR was performed separately within each treatment arm using imputation models containing the minimization factors, baseline antidepressant use and omega-3 index. A range of δ from a 25% increase in CDRS-R score (25% worse outcome) to a 100% increase (by 25% increments) in the CDRS-R score in the intervention dropouts as compared to the observed cases were considered. These δ were added to the imputed CDRS-R scores for the participants with intervention dropout. Results were combined using Rubin's rule.

The consistency of the treatment effect was evaluated by adding an interaction term between treatment effect, time, and omega-3 index to the model described above. Furthermore, interactions between treatment effect and each of sex, Covid-19, and anxiety were assessed.

The binary secondary CDRS-R outcomes remission at any time during study follow up and response at any time during the first 12 weeks were analyzed using logistic regression models adjusted for the same covariates as the primary outcome analysis. Adjusted odds ratios with 95% confidence intervals were used to compare the outcome odds in the Omega-3 treatment arm to that in the placebo arm.

The continuous secondary outcomes KIDscreen-CAT-10 and DIKJ were analyzed using linear regression models with 36-week values as the outcome, treatment arm as the covariate of interest, the covariates of the primary outcome analysis, and the baseline value of the respective screening tool as a covariate (ANCOVA). The mean difference in outcome scores and 95% confidence intervals were computed to compare treatment arms. Missing outcome data were multiply imputed, as described above. In addition, the analyses of these two outcomes were done using the same joint model as for the primary outcome.

Intervention dropout per treatment arm was visualized using the Kaplan Meier method. A Cox proportional hazards model was fit including all covariates from the primary outcome analysis. Risk of additional antidepressants was compared across treatment arms using the resulting hazard ratio and 95% confidence interval.

Number of days in hospital and outpatient service use were summarized overall and per treatment arm.

Adverse events were summarized by number and percent both overall and per treatment arm. As another measure for adverse events, the ASEC items are shown by treatment group.

To assess the compliance, both EPA and DHA levels over time as well as pillcounts are shown descriptively per treatment group.

All p-values are presented for hypotheses outlined in the statistical analysis plan. They are two-sided with a nominal type I error rate of 5%.

Software All analyses were performed in the R programming language (R Core Team, 2018). The R package `knitr` in combination with LaTeXwas used for dynamic reporting.

Data This report is based on the data export from Monika Poppe, CTC USZ on July 1st, 2022.

3.1 Deviations from statistical analysis plan

As stated in the protocol, simpler models would be used in the event of computational/convergence issues. The following changes became necessary:
The primary analysis of CDRS-R ignored site and only included current longitudinal value, as opposed to both value and slope.
The linear mixed effects model considered time a continuous variable.
The survival model considered study and intervention dropout a single type of event, instead of using a competing risk model.
The secondary binary outcomes were to be analyzed using log binomial models for relative risks, however, for computational reasons, logistic regression models for odds ratios were used instead.
As self-reported quality of life measure the KIDscreen 10 was used instead of the KIDscreen-CAT-27, because KIDscreen 10 is an overall score whereas KIDscreen-CAT-27 consists of five separate scores.

3.2 Decisions made during analysis

- During the trial the use of additional antidepressants were assumed to affect future visits only if initiation of the new medication or increase in dose occurred 7 days or more prior to the next visit. This 7-day window, however, was not applied at baseline. Applying this window to baseline would have meant censoring of three participants prior to the baseline visit.

- Dropout visits were not used to supplement the missing data, even if the dropout visit occurred very close to the planned visit date. It is assumed that dropout visits often occur after non-adherence to protocol, which is supported by the data.

- Non-adherence was documented in more than one way, including compliance rate within the medication file and drop-out reasons in the premature study end file. Non-adherence was declared if either the compliance rate was below 60% in the medication file or if compliance was included among the drop-out reasons.

- 19 patients have at least 1 missing compliance value after the baseline visit and are included in the analyses (as long as they are not a drop-out).

- Pillcounts percentages over 100% were changed to 100%.

- Baseline Omega 3 Index had 2 missing values (both in the Placebo group), which were imputed by mean imputation.

- Additional analyses for the secondary outcomes KIDscreen 10 and DIKJ were done using the joint model like for the primary analysis of the primary outcome.

4 Results

Figure 1 depicts the flow of participants from screening through to the primary analysis. A total of 310 participants were screened, 53 of which were excluded at this stage. These exclusions were due to screening failure (32), declining to provide consent (12), and other reasons (9). A total of 257 participants were randomized: 128 to the placebo arm and 129 to the Omega-3 arm. By the final follow-up visit at 36 weeks, 168 participants had dropped out: 100 due to additional antidepressant use, 48 for non-adherence to protocol, and 20 for other reasons. In primary analyses, all 257 participants are analyzed as randomized.

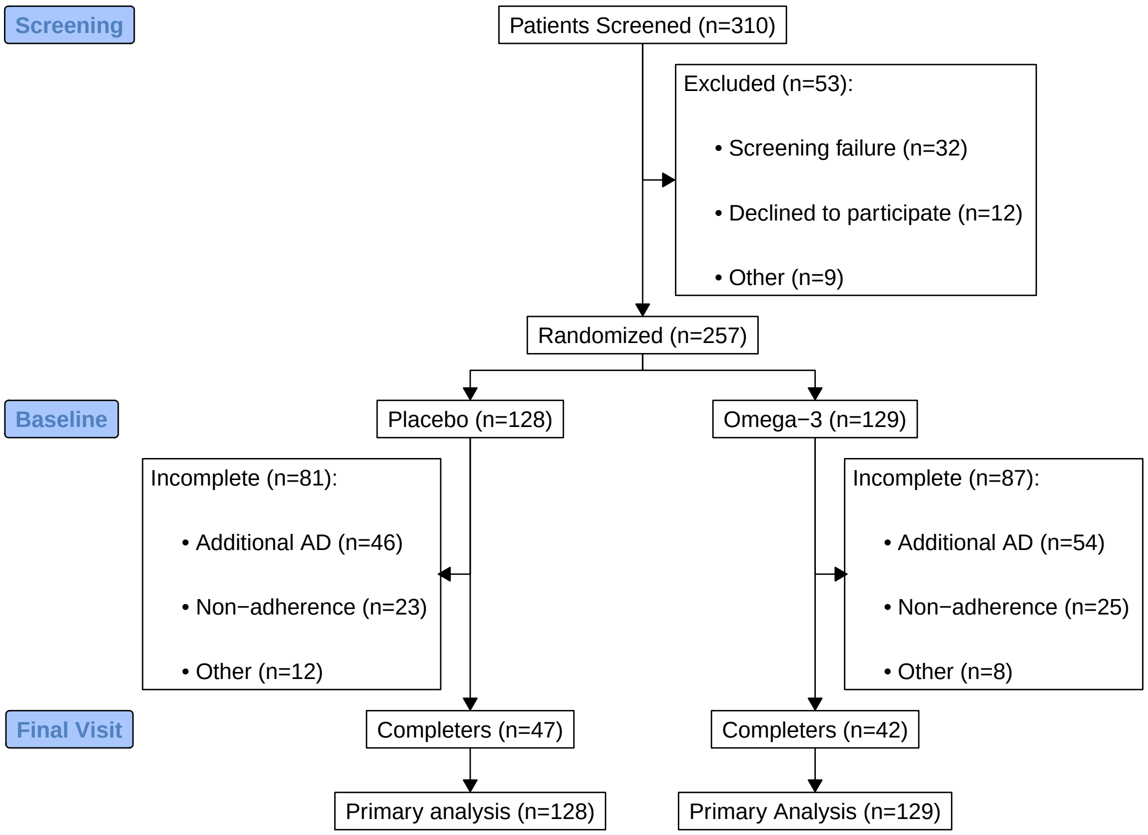

Figure 1: Consort flow diagram of trial participants from screening through follow-up.

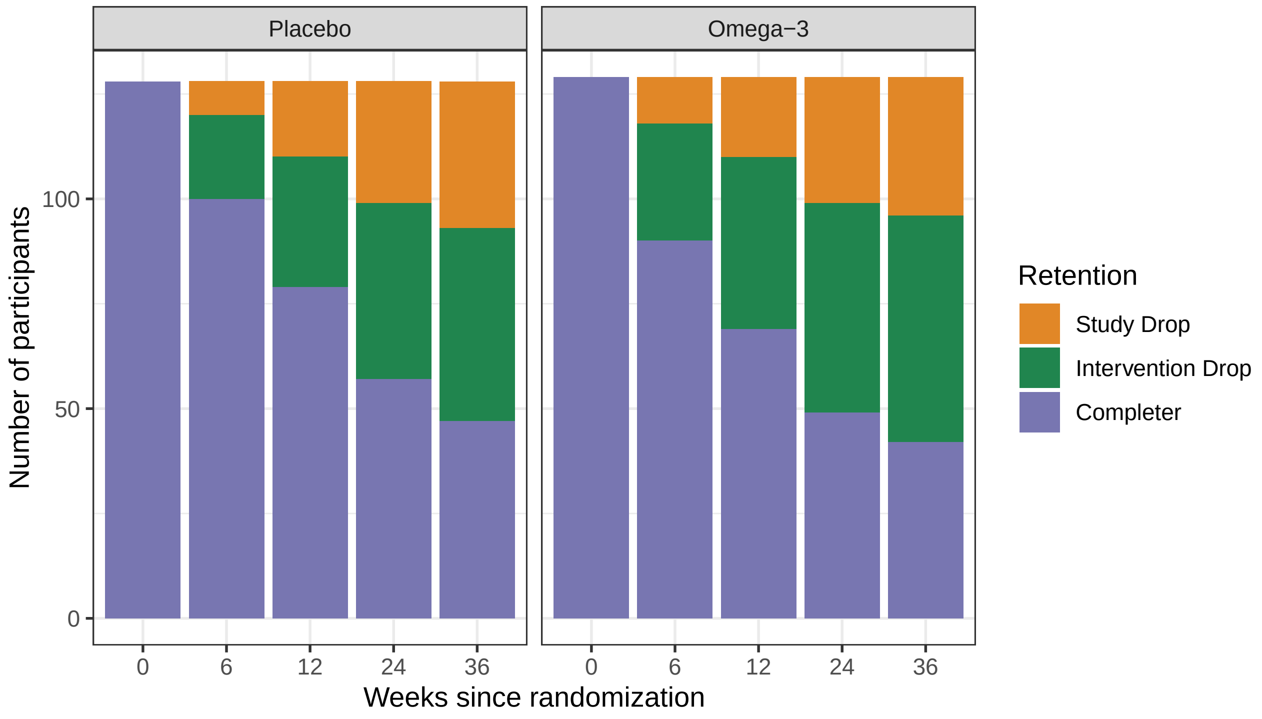

Figure 2: Participant retention per treatment arm over across study visits.

Table 1: Table depicting participant retention throughout trial.

	Baseline	6 weeks	12 weeks	24 weeks	36 weeks
			Overall		
Completer	257	190	148	106	89
Intervention Drop	0	48	72	92	100
Study Drop	0	19	37	59	68
			Placebo		
Completer	128	100	79	57	47
Intervention Drop	0	20	31	42	46
Study Drop	0	8	18	29	35
			Omega-3		
Completer	129	90	69	49	42
Intervention Drop	0	28	41	50	54
Study Drop	0	11	19	30	33

Table 1 and Figure 1 further describe the retention of study participants over the 36-week observation period. Similar trends are seen across the treatment arms. More participants initiate or increase antidepressant dose (intervention drop-out) rather than leave the study (study drop-out). Both types of drop-out increase as the study progresses. By study end, 34.6% of participants are still adherent to protocol ("completers", 36.7% and 32.6% in the placebo and treatment arms, respectively).

4.1 Descriptive statistics: Baseline characteristics

Table 2 depicts the patients' baseline characteristics. In total, 257 participants from 7 centers (SG Out-patient services: 32, SG Ganterschwil: 29, TG Out-patient services: 17, TG Littenheid: 55, BL: 12, BS: 18, ZH: 94) were included in the study.

Minimization factors were well-balanced across the treatment arms. The average age at study baseline was 15.7 years and 73.2% were female. At baseline, 35% of participants were on another antidepressant, mostly Fluoxetin or Sertralin. However, a higher percentage of participants in the Omega-3 arm were experiencing a recurrent episode of depression as compared to the Placebo arm (27.1% versus 16.4%).

Table 2: Baseline characteristics. Anxiety/panic disorders here are without specific phobias.

Characteristic	Levels	Overall	Placebo	Omega-3	Missing (%)
n		257	128	129	
Age		15.66 (1.66)	15.64 (1.74)	15.69 (1.58)	0.0
Sex	M	69 (26.8)	34 (26.6)	35 (27.1)	0.0
	W	188 (73.2)	94 (73.4)	94 (72.9)	
BMI		22.38 (5.28)	22.11 (4.52)	22.65 (5.95)	9.3
hsCRP	Low	179 (69.6)	90 (70.3)	89 (69.0)	0.0
	Average	53 (20.6)	25 (19.5)	28 (21.7)	
	High	25 (9.7)	13 (10.2)	12 (9.3)	
Omega 3 Index		4.58 (1.09)	4.54 (1.10)	4.63 (1.08)	0.0
Recurrent episode	No	201 (78.2)	107 (83.6)	94 (72.9)	0.0
	Yes	56 (21.8)	21 (16.4)	35 (27.1)	
Age of onset		13.79 (2.38)	13.93 (2.27)	13.65 (2.49)	0.0
Duration of previous episodes		16.28 (17.14)	16.76 (18.98)	15.79 (15.12)	2.3
Anxiety/panic	No	233 (90.7)	118 (92.2)	115 (89.1)	0.0
	Yes	24 (9.3)	10 (7.8)	14 (10.9)	
Attention disorder	No	220 (85.6)	112 (87.5)	108 (83.7)	0.0
	Yes	37 (14.4)	16 (12.5)	21 (16.3)	
Psychotic features	No	245 (95.3)	121 (94.5)	124 (96.1)	0.0
	Yes	12 (4.7)	7 (5.5)	5 (3.9)	
Antidepressant use	No	167 (65.0)	81 (63.3)	86 (66.7)	0.0
	Yes	90 (35.0)	47 (36.7)	43 (33.3)	
Antidepressant name	Escitalopram	11 (12.2)	6 (12.8)	5 (11.6)	
	Fluoxetin	38 (42.2)	16 (34.0)	22 (51.2)	
	Mirtazapin	1 (1.1)	1 (2.1)	0 (0.0)	
	Mirtazapin + Fluoxetin	4 (4.4)	0 (0.0)	4 (9.3)	
	Other Combination	2 (2.2)	2 (4.3)	0 (0.0)	
	Sertralin	30 (33.3)	20 (42.6)	10 (23.3)	
	Valdoxan	1 (1.1)	1 (2.1)	0 (0.0)	
	Venlafaxin	1 (1.1)	0 (0.0)	1 (2.3)	
	Vortioxetin	1 (1.1)	1 (2.1)	0 (0.0)	
	Wellbutrin	1 (1.1)	0 (0.0)	1 (2.3)	
CDRS		58.54 (8.78)	58.13 (8.42)	58.95 (9.15)	0.0
DIKJ		28.09 (9.46)	28.12 (9.91)	28.06 (9.06)	4.3
KidScreen 10		29.05 (5.72)	29.25 (5.74)	28.87 (5.71)	6.6
SIQ-Jr		40.91 (23.49)	39.80 (24.31)	41.95 (22.73)	6.6
EPA		0.40 (0.10)	0.41 (0.10)	0.40 (0.10)	0.8
DHA		4.18 (1.04)	4.13 (1.05)	4.23 (1.04)	0.8
Center	SG Out-patient	32 (12.5)	17 (13.3)	15 (11.6)	0.0
	SG Ganterschwil	29 (11.3)	14 (10.9)	15 (11.6)	
	TG Out-patient	17 (6.6)	10 (7.8)	7 (5.4)	
	TG Littenheid	55 (21.4)	27 (21.1)	28 (21.7)	
	BL	12 (4.7)	6 (4.7)	6 (4.7)	
	BS	18 (7.0)	7 (5.5)	11 (8.5)	
	ZH	94 (36.6)	47 (36.7)	47 (36.4)	
Clinic type	In-patient	80 (31.1)	39 (30.5)	41 (31.8)	0.0
	Out-patient	164 (63.8)	82 (64.1)	82 (63.6)	
	Day unit	13 (5.1)	7 (5.5)	6 (4.7)	

4.2 Descriptive statistics: Continuous outcome trajectories

The figures of this section are meant for visual exploration and show the unadjusted results.

Table 3 gives the mean outcome measurement per treatment arm for each visit (KidScreen was not collected at the 6 week and 24 week visits).

Table 3: Continuous primary and secondary outcome measurements over time (data censored at non-compliance or additional antidepressant use).

	Baseline	6 weeks	12 weeks	24 weeks	36 weeks
			CDRS		
Placebo	58.13 (8.42)	49.03 (12.05)	46.08 (12.99)	42.41 (13.85)	36.83 (15.46)
n Placebo	128	100	79	58	47
Omega-3	58.95 (9.15)	49.4 (12.26)	45.93 (11.98)	40.24 (14.06)	36.5 (13.12)
n Omega-3	129	90	69	51	42
			DIKJ		
Placebo	28.12 (9.91)	25.53 (9.61)	23.03 (11.23)	20.5 (11.87)	18.21 (12.12)
n Placebo	120	97	69	50	42
Omega-3	28.06 (9.06)	25.86 (11.24)	22.18 (9.28)	19.78 (11.29)	17.86 (11.04)
n Omega-3	126	81	62	45	37
			KidScreen		
Placebo	29.25 (5.74)		32.26 (7.12)		34.88 (8.36)
n Placebo	115		65		41
Omega-3	28.87 (5.71)		32.38 (5.91)		34.28 (7.23)
n Omega-3	125		60		36
			SIQ-Jr		
Placebo	39.8 (24.31)	31.86 (20.76)	29.52 (23.46)	27.54 (23.83)	22.95 (22.44)
n Placebo	116	95	71	52	43
Omega-3	41.95 (22.73)	33.42 (21.74)	28.25 (17.02)	27.45 (22.92)	20.86 (18)
n Omega-3	124	83	61	47	35

Figure 3 depicts the Children's Depression Rating Scale total score trajectories per participant, with treatment arm means depicted by bold dashed lines. The difference in mean score across treatment arms is small across all time points. The placebo arm means are consistently lower when looking at the data as observed (A) and the data with censored non-compliance (B), however, when looking at the data removing visits after additional antidepressants (C: censoring observations after antidepressants as well as non-compliance, D: per-protocol data), the treatment arm means are even closer and the Omega-3 means are lower for the later time points. Figure 4 depicts these trajectories separately for participants on and off antidepressants at baseline. The mean scores are slightly lower for participants without baseline antidepressant use across the study period. Again, we see very similar mean trajectories per treatment arm. Very small differences are in favor of Omega-3 for the analysis set (B), especially at later time points. Figure 5 illustrates the CDRS trajectories separately for participants experiencing a first depressive episode and those experiencing a recurrent episode. Mean difference across treatment arms are small. The difference is in favor of Omega-3 for participants experiencing a first episode, but in favor of placebo in those experiencing a recurrence of depression.

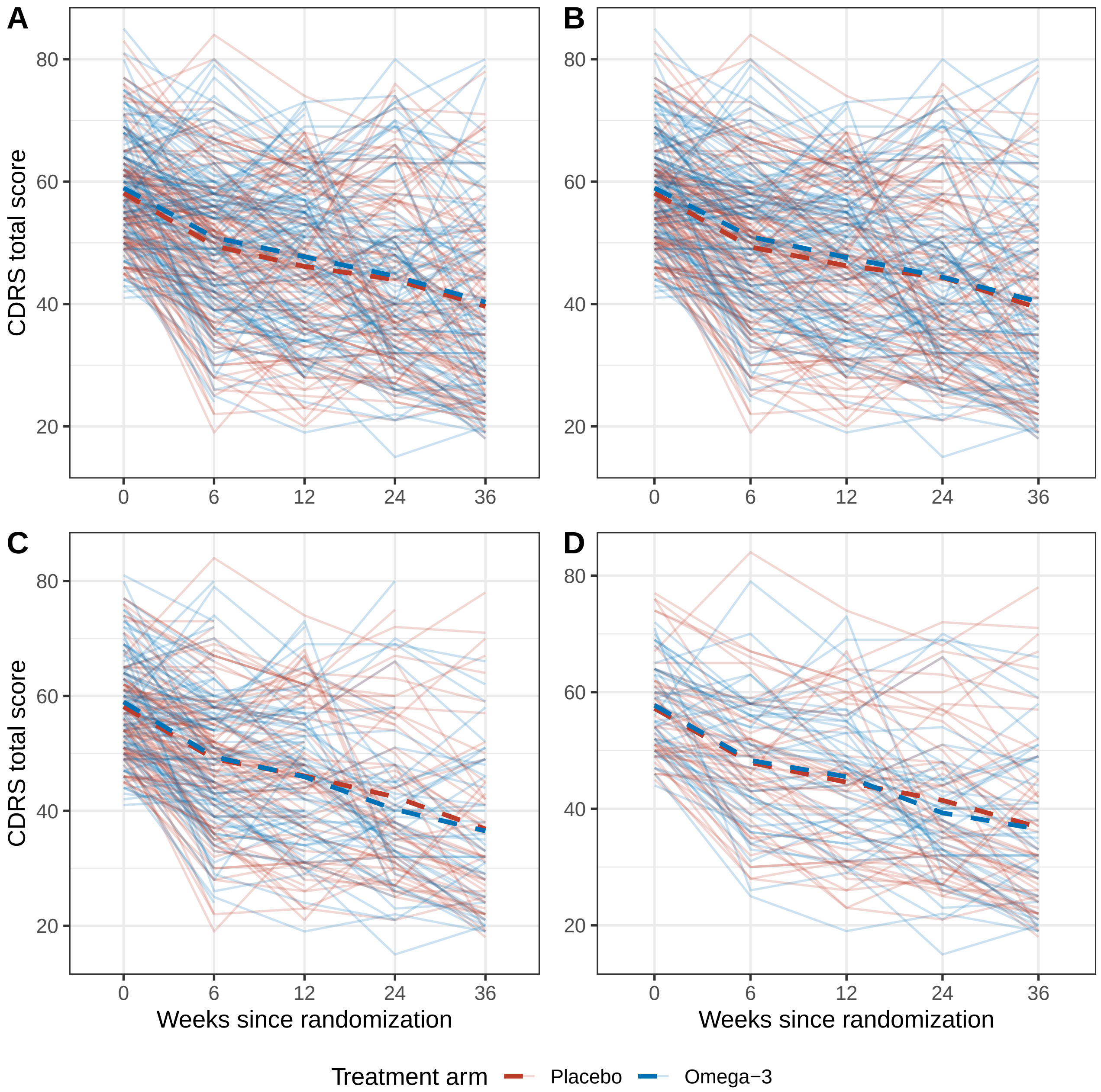

Figure 3: Individual CDRS trajectories with mean trajectories per treatment arm in bold: (A) data as observed, (B) data censored for non-compliance, (C) data censored for non-compliance and additional antidepressant use, and (D) per-protocol data.

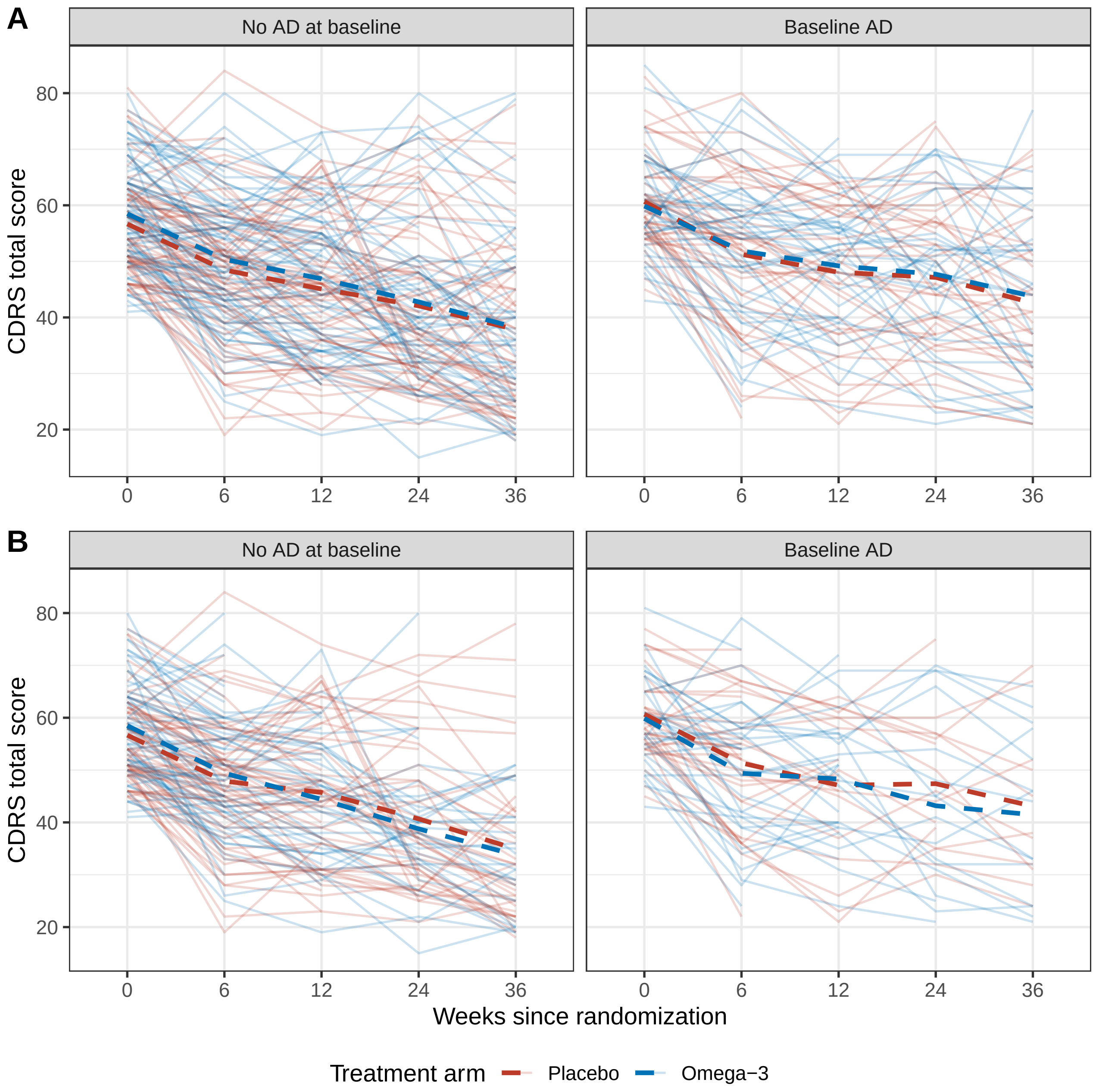

Figure 4: Individual CDRS trajectories with mean trajectories per treatment arm in bold for those on and not on an antidepressant at baseline separately: (A) on the data as observed, (B) using the analysis set (censoring observations at non-compliance and after additional antidepressant use).

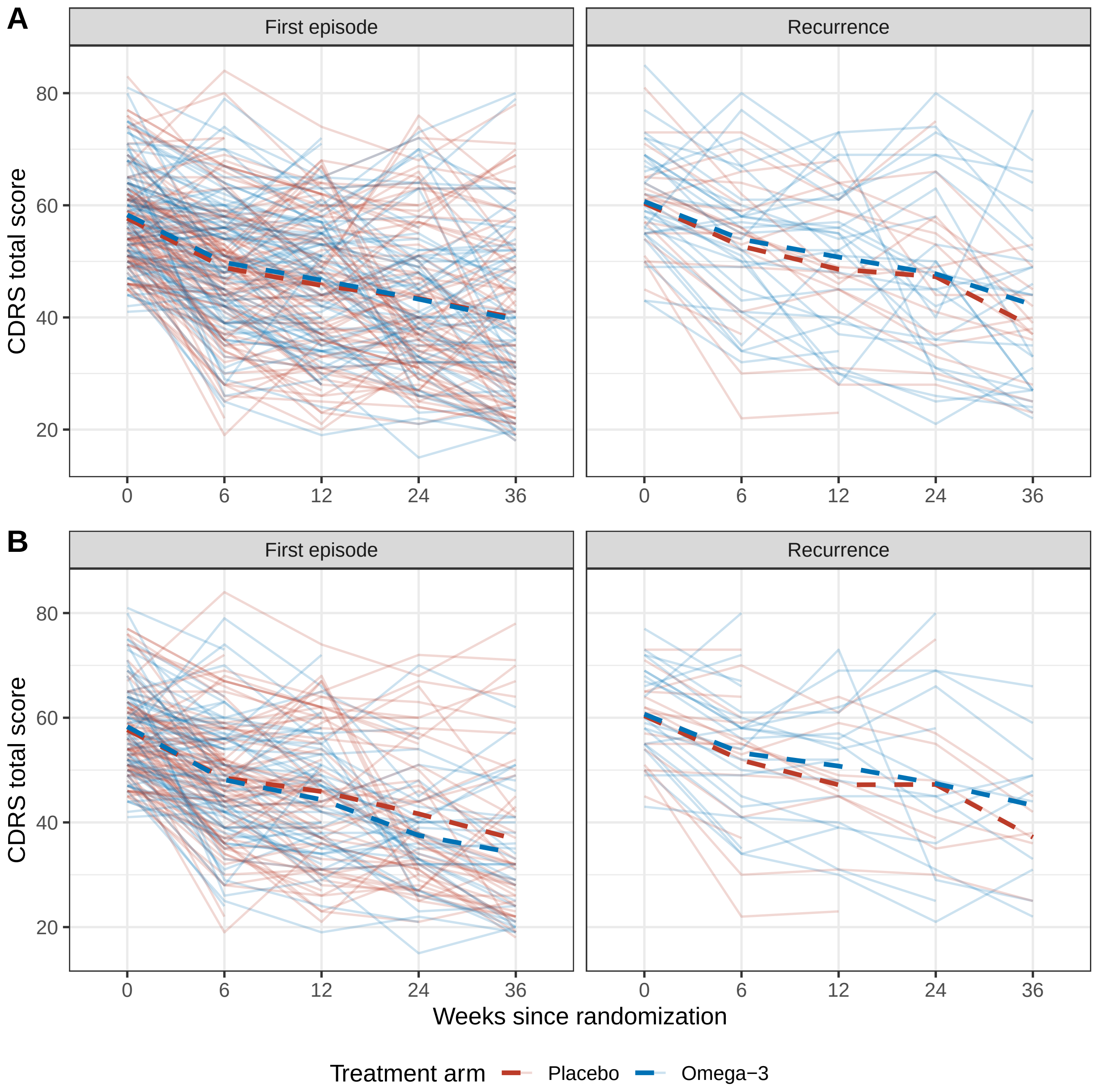

Figure 5: Individual CDRS trajectories with mean trajectories per treatment arm in bold for those experiencing first or recurrent depressive episode separately: (A) on the data as observed, (B) using the analysis set (censoring observations at non-compliance and after additional antidepressant use).

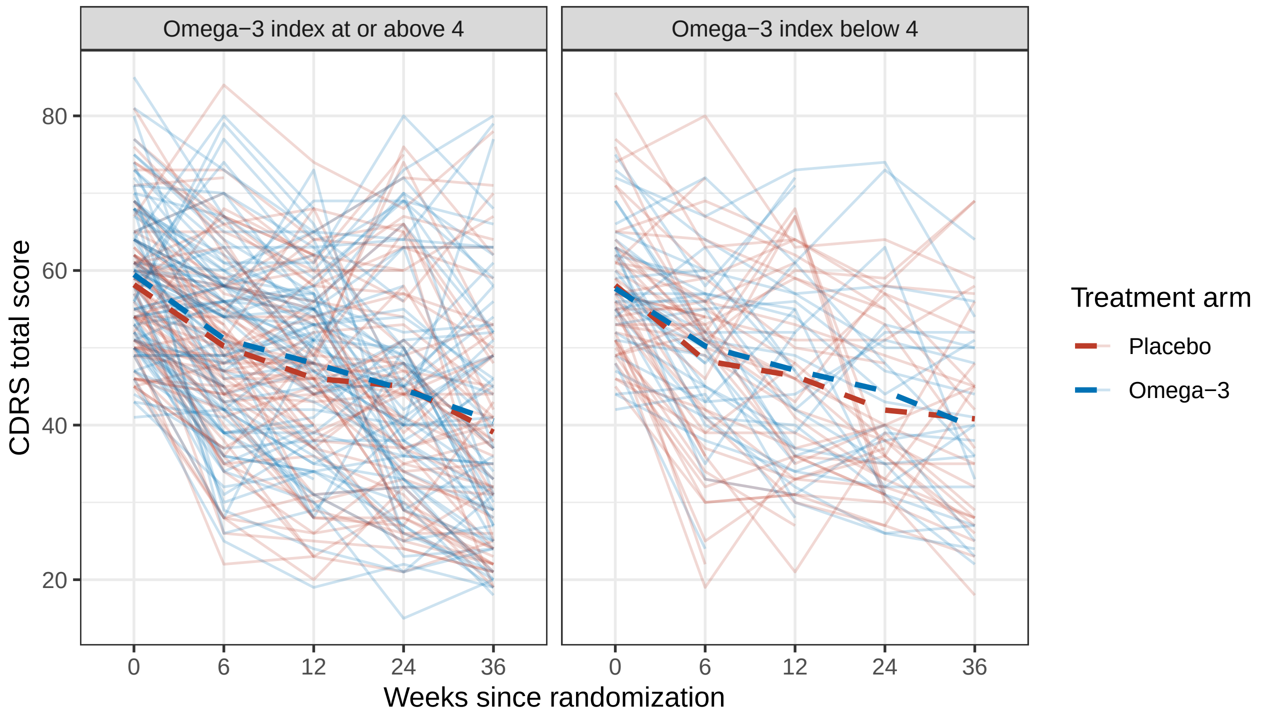

Figure 6: Individual CDRS trajectories with mean trajectories per treatment arm in bold for those with high and low baseline omega-3 index values, separately.

Figure 7 depicts the trajectories for the Children's Depression Inventory (DIKJ) total score over the study period. The differences between the means per treatment arm are small over the entire period, especially in relation to the variation between participants. Only when using the per-protocol data are the small differences in DIKJ consistently in favor of the Omega-3 arm.

Figure 8 depicts the trajectories for the KIDscreen-CAT-10 quality of life total score over the study period. The differences between treatment arms are small, but slightly in favor of the placebo arm in all data sets (as observed through to per-protocol).

Figure 9 depicts the trajectories for the Suicidal Ideation Questionnaire total score over the study period. As for the other outcomes, the differences between treatment groups are small at all time periods.

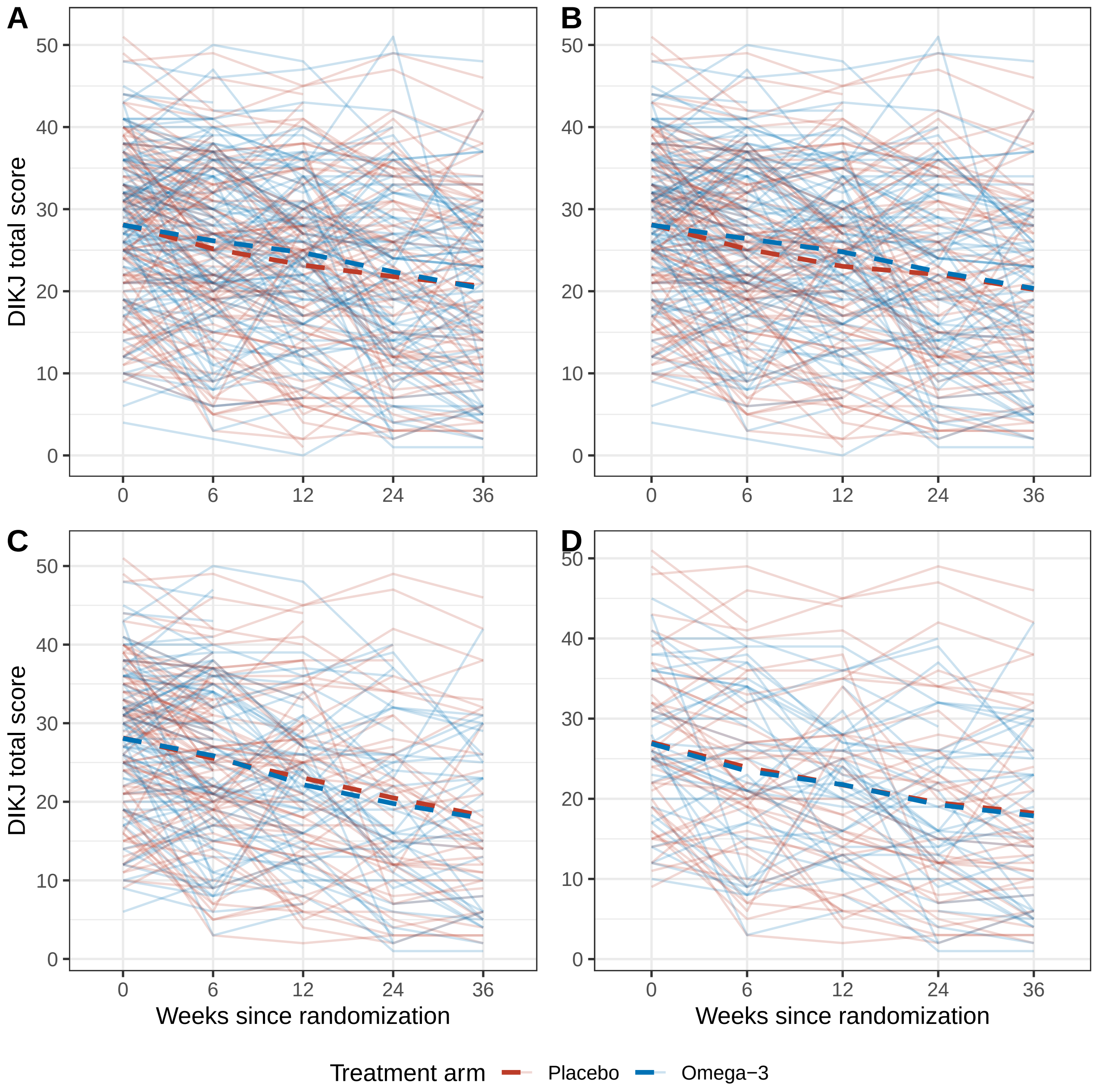

Figure 7: Individual DIKJ trajectories with mean trajectories per treatment arm in bold: (A) data as observed, (B) data censored for non-compliance, (C) data censored for non-compliance and additional antidepressant use, and (D) per-protocol data.

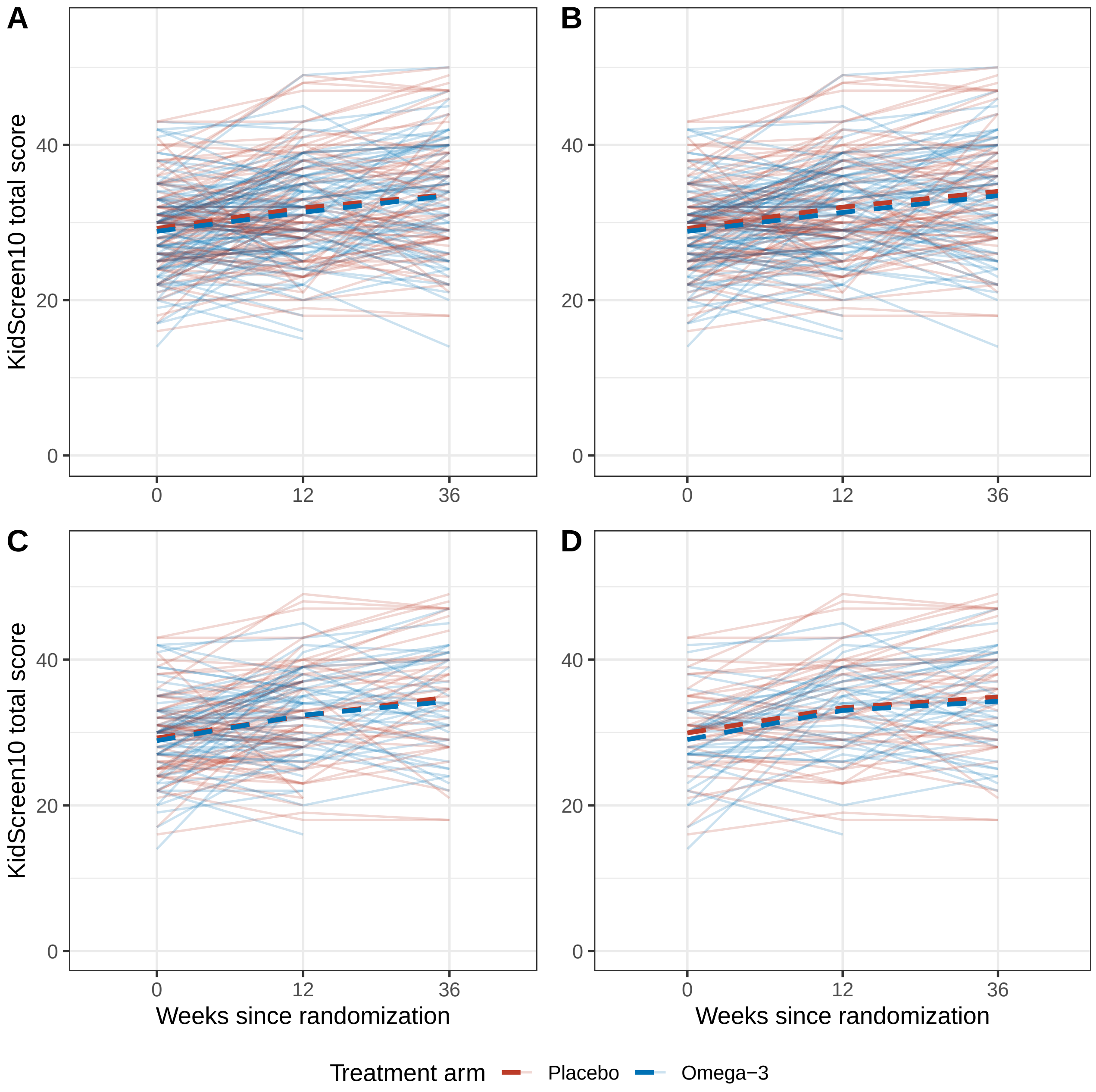

Figure 8: Individual KidScreen trajectories with mean trajectories per treatment arm in bold: (A) data as observed, (B) data censored for non-compliance, (C) data censored for non-compliance and additional antidepressant use, and (D) per-protocol data.

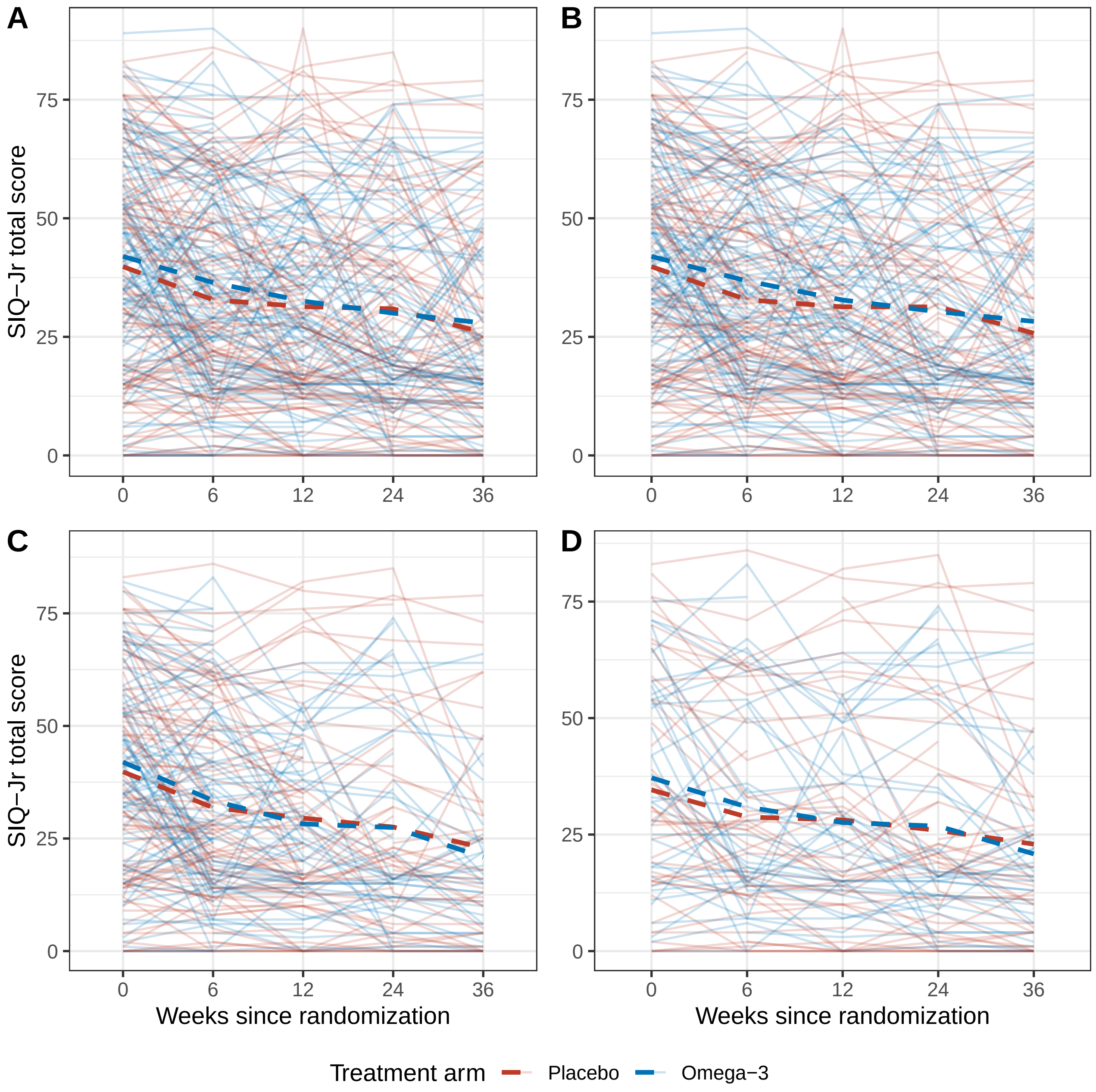

Figure 9: Individual SIQ-Jr trajectories with mean trajectories per treatment arm in bold: (A) data as observed, (B) data censored for non-compliance, (C) data censored for non-compliance and additional antidepressant use, and (D) per-protocol data.

4.2.1 Descriptive statistics: Binary outcome measures

Table 4: Remission status per visit. The denominator at each visit includes those observed at that visit and those experiencing remission before that visit but exiting the study before that visit, but not those exiting prior to that visit whom had not yet had reached remission.

	6 weeks	12 weeks	24 weeks	36 weeks
Placebo	8/120 (6.67%)	16/109 (14.68%)	22/102 (21.57%)	37/91 (40.66%)
Omega-3	4/118 (3.39%)	7/111 (6.31%)	17/94 (18.09%)	30/91 (32.97%)

Table 5: Response status per visit. The denominator at each visit includes those observed at that visit and those experiencing the response earlier but exiting the study prior to that visit, but not those exiting the study prior to the visit whom had not yet responded.

	6 weeks	12 weeks
Placebo	21/120 (17.50%)	43/110 (39.1%)
Omega-3	20/118 (16.95%)	34/111 (30.6%)

The study definition for remission is a CDRS total score <28 at any point during the 36 weeks. There were 67 participants meeting this criterium (30 in the Omega-3 arm and 37 in the Placebo arm). The study definition for response is a 30% decrease or greater in CDRS total score at any point during the first 12 weeks of the trial. There were 77 participants meeting this criterium (34 in the Omega-3 arm and 43 in the Placebo arm).

Tables 4 and 5 describe the remission and response, respectively. The unadjusted numbers presented here are somewhat difficult to interpret due to differing lengths of follow-up time across participants. This problem is dealt with during analysis through the use of multiple imputation. Remission and response are tabularized and visualized over time using cumulative numbers including participants observed at a given time point or experiencing the outcome prior to exiting the study before that time point.

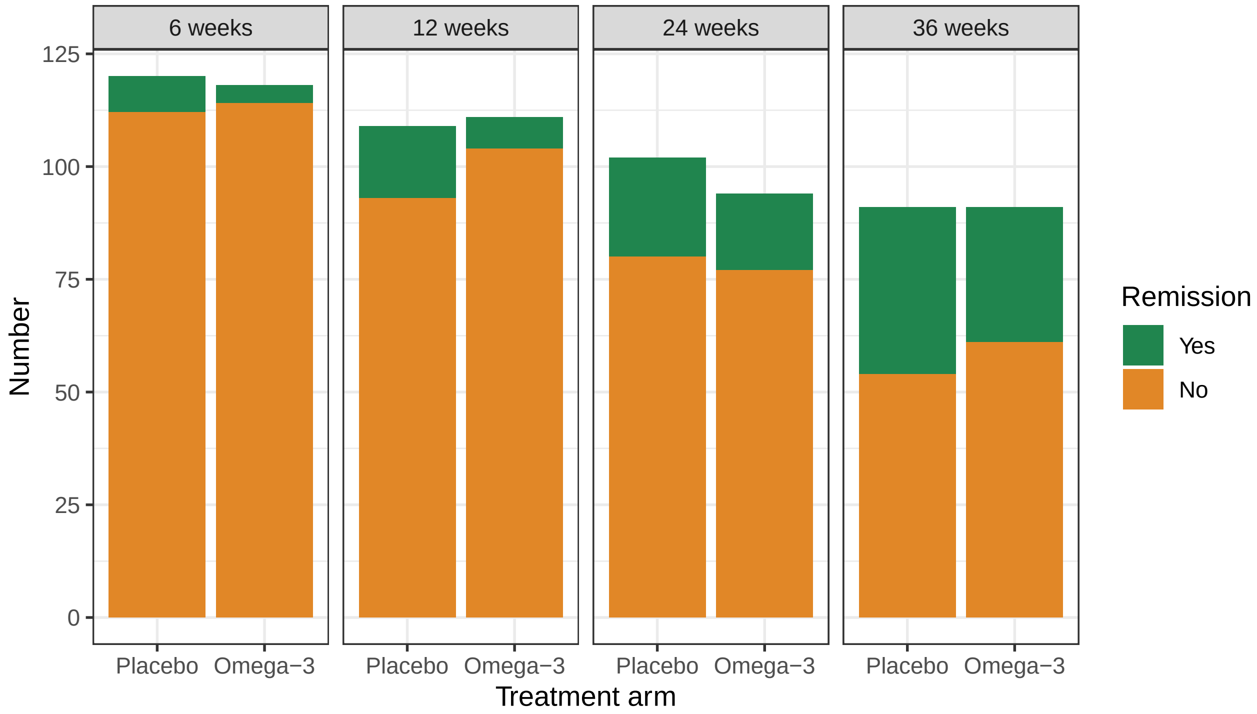

Figure 10: Remission per time point.

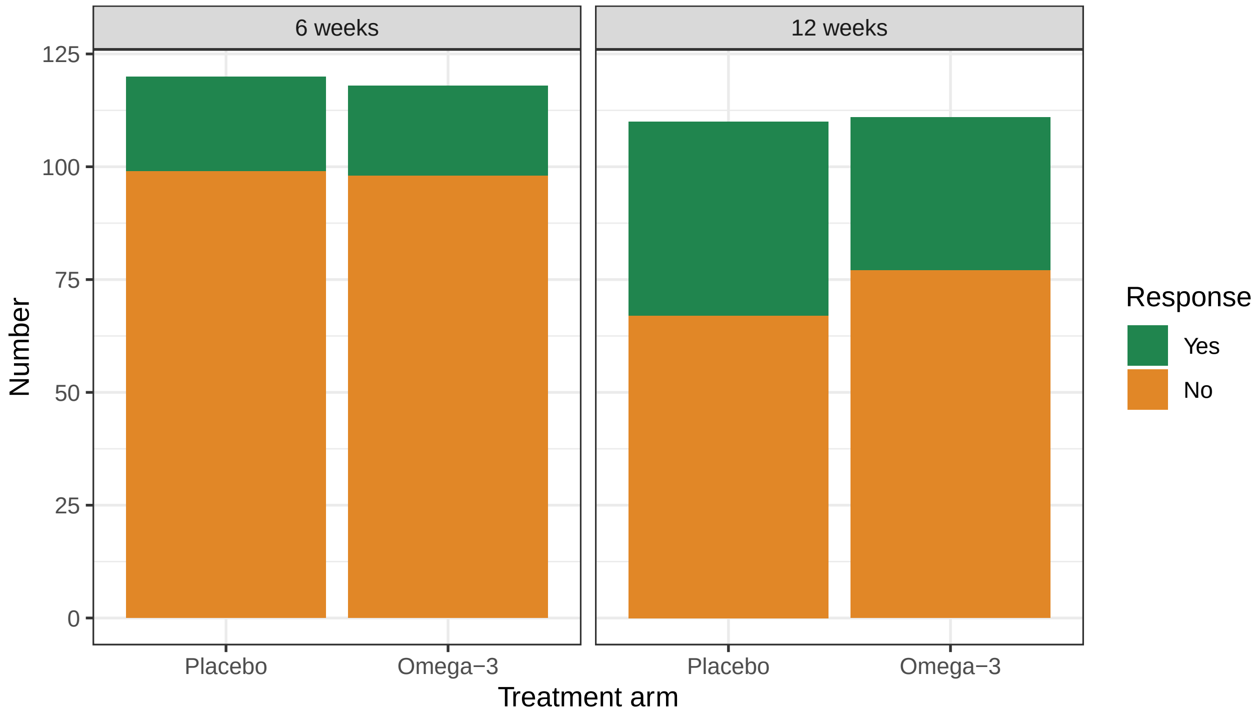

Figure 11: Response over time.

4.3 Primary endpoint

Joint model results

Table 6: Primary analysis results from joint modeling of the primary outcome and time to dropout. Study and intervention dropout were considered a single type of event.

Parameter	Estimate	CI	P
Longitudinal			
(Intercept)	44.15	32.39 to 55.66	$<$ 0.0001
Week	-0.49	-0.57 to -0.4	$<$ 0.0001
Omega-3 treatment	0.80	-1.5 to 3.04	0.48
Age	0.43	-0.24 to 1.11	0.20
Female sex	5.56	3.04 to 8.02	$<$ 0.0001
hsCRP - average	-0.33	-3.16 to 2.5	0.82
hsCRP - high	-0.35	-4.03 to 3.18	0.86
Omega3 index	-0.09	-1.14 to 0.95	0.85
baseline AD	1.98	-0.41 to 4.39	0.10
Recurrence	2.53	-0.26 to 5.33	0.080
Week:Omega-3 treatment	-0.04	-0.16 to 0.09	0.53
Time-to-Event			
Omega-3 treatment	1.22	0.83 to 1.81	0.33
Predicted CDRS-R	1.03	1.01 to 1.05	0.009

The joint model for the primary analysis additionally included the baseline covariate recurrent episode as it was found to be imbalanced across treatment groups. The time-to-event submodel considered study and intervention dropout to be a single type of event and included the current predicted estimated CDRS-R score as a covariate, but not the current predicted slope. Average CDRS-R score was found to change. However, no evidence was found to indicate a difference in CDRS-R trajectories per treatment arm.

4.3.1 Sensitivity analyses for the primary endpoint

LMM using data as observed

Table 7: Sensitivity Analysis I (data as observed): Adjusted linear mixed model results for the relationship between treatment arm and CDRS slopes. The model includes random intercepts for participant and center. This model did not result in convergence messsages.

Variable	Estimate	X95.confidence.interval	p.value
(Intercept)	41.95	[29.16, 54.74]	< 0.0001
Week	-0.47	[-0.54, -0.40]	< 0.0001
Omega-3 treatment	1.01	[-1.57, 3.59]	0.44
Age	0.46	[-0.27, 1.19]	0.21
Female sex	5.78	[3.17, 8.39]	< 0.0001
hsCRP - average	-0.49	[-3.44, 2.47]	0.75
hsCRP - high	0.86	[-3.09, 4.81]	0.67
Omega3 index	0.10	[-0.98, 1.18]	0.86
baseline AD	1.64	[-0.93, 4.21]	0.21
Recurrence	1.31	[-1.60, 4.22]	0.38
Week:Omega-3 treatment	-0.03	[-0.13, 0.07]	0.52

Table 8: Sensitivity Analysis I (data as observed): Adjusted linear mixed model results for the relationship between treatment arm and CDRS slopes. The model includes random intercepts for participant and center and random slopes for particpants. This model resulted in convergence messages.

Variable	Estimate	X95.confidence.interval	p.value
(Intercept)	43.19	[31.81, 54.56]	< 0.0001
Week	-0.53	[-0.61, -0.45]	< 0.0001
Omega-3 treatment	1.00	[-1.06, 3.06]	0.34
Age	0.46	[-0.19, 1.10]	0.16
Female sex	5.50	[3.19, 7.81]	< 0.0001
hsCRP - average	0.35	[-2.26, 2.95]	0.79
hsCRP - high	0.30	[-3.16, 3.77]	0.86
Omega3 index	-0.06	[-1.01, 0.89]	0.90
baseline AD	0.59	[-1.70, 2.89]	0.61
Recurrence	1.62	[-0.96, 4.20]	0.22
Week:Omega-3 treatment	-0.04	[-0.15, 0.08]	0.51

LMM using data censored at additional antidepressant use

Table 9: Sensitivity Analysis II (censored observations after AD use): Adjusted linear mixed model results for the relationship between treatment arm and CDRS slopes. The model includes random intercepts for participant and center. This model did not result in convergence messages.

Variable	Estimate	X95.confidence.interval	p.value
(Intercept)	43.00	[30.73, 55.26]	< 0.0001
Week	-0.53	[-0.61, -0.45]	< 0.0001
Omega-3 treatment	0.36	[-2.02, 2.74]	0.77
Age	0.55	[-0.15, 1.25]	0.12
Female sex	5.15	[2.61, 7.70]	< 0.0001
hsCRP - average	-0.86	[-3.72, 2.00]	0.56
hsCRP - high	-0.11	[-3.79, 3.56]	0.95
Omega3 index	-0.20	[-1.27, 0.86]	0.71
baseline AD	0.99	[-1.55, 3.52]	0.45
Recurrence	2.25	[-0.58, 5.09]	0.12
Week:Omega-3 treatment	-0.01	[-0.12, 0.10]	0.83

Table 10: Sensitivity Analysis II (censored observations after AD use): Adjusted linear mixed model results for the relationship between treatment arm and CDRS slopes. The model includes random intercepts for participant and center and random slopes for particpants. This model resulted in convergence messages.

Variable	Estimate	X95.confidence.interval	p.value
(Intercept)	45.19	[33.61, 56.77]	< 0.0001
Week	-0.57	[-0.66, -0.49]	< 0.0001
Omega-3 treatment	0.46	[-1.62, 2.54]	0.66
Age	0.45	[-0.21, 1.10]	0.18
Female sex	4.73	[2.36, 7.10]	< 0.0001
hsCRP - average	-0.26	[-2.92, 2.39]	0.85
hsCRP - high	0.63	[-2.83, 4.09]	0.72
Omega3 index	-0.26	[-1.25, 0.72]	0.60
baseline AD	0.61	[-1.75, 2.98]	0.61
Recurrence	1.48	[-1.17, 4.14]	0.27
Week:Omega-3 treatment	0.01	[-0.12, 0.14]	0.88

LMM using per-protocol data

Table 11: Sensitivity analysis III (per protocol data): Adjusted linear mixed model results for the relationship between treatment arm and CDRS slopes. The model includes random intercepts for participant and center. This model did not result in convergence messages.

Variable	Estimate	X95.confidence.interval	p.value
(Intercept)	38.15	[20.98, 55.31]	< 0.0001
Week	-0.51	[-0.60, -0.43]	< 0.0001
Omega-3 treatment	-1.27	[-5.10, 2.56]	0.52
Age	0.57	[-0.45, 1.59]	0.27
Female sex	6.84	[2.82, 10.85]	0.0008
hsCRP - average	-3.74	[-8.31, 0.84]	0.11
hsCRP - high	-0.81	[-6.31, 4.69]	0.77
Omega3 index	0.34	[-1.52, 2.19]	0.72
baseline AD	5.53	[1.58, 9.49]	0.006
Recurrence	1.56	[-2.98, 6.10]	0.50
Week:Omega-3 treatment	0.01	[-0.11, 0.13]	0.88

Table 12: Sensitivity analysis III (per protocol data): Adjusted linear mixed model results for the relationship between treatment arm and CDRS slopes. The model includes random intercepts for participant and center and random slopes for particpants. This model resulted in convergence messages. Unlike the other results, this model does not adjust for recurrence.

Variable	Estimate	X95.confidence.interval	p.value
(Intercept)	41.95	[26.12, 57.78]	< 0.0001
Week	-0.55	[-0.65, -0.46]	< 0.0001
Omega-3 treatment	-0.72	[-3.95, 2.51]	0.66
Age	0.36	[-0.58, 1.31]	0.45
Female sex	6.19	[2.49, 9.89]	0.001
hsCRP - average	-3.31	[-7.54, 0.92]	0.13
hsCRP - high	1.12	[-3.96, 6.20]	0.66
Omega3 index	0.34	[-1.37, 2.05]	0.70
baseline AD	5.32	[1.76, 8.88]	0.003
Week:Omega-3 treatment	0.01	[-0.12, 0.14]	0.88

LMM using imputed data

Table 13: Sensitivity analysis IV, Delta-based multiple imputation for MNAR analysis. Adjusted linear mixed model results for the relationship between treatment arm and CDRS slopes. The model includes random intercepts for participants. This model does not adjust for recurrence.

	Estimate	95%-confidence	interval	p-value
Delta = 0	0.97	-1.44	3.38	0.43
25%	1.00	-1.62	3.61	0.46
50%	1.02	-1.86	3.90	0.49
75%	1.04	-2.12	4.21	0.52
100%	1.06	-2.38	4.51	0.55

4.3.2 Subgroup analysis for the primary endpoint

Evaluation of moderating effects of the treatment and different baseline characteristics.

Table 14: Results from joint modeling of the primary outcome and time to dropout including an interaction term between treatment effect, time, and omega-3 index. Study and intervention dropout were considered a single type of event.

Parameter	Estimate	CI	P
	Longitudinal		
(Intercept)	44.79	33.03 to 56.65	< 0.0001
Week	-0.72	-1.13 to -0.3	< 0.0001
Omega-3 treatment	-0.41	-9.56 to 8.9	0.93
Age	0.43	-0.21 to 1.07	0.18
Female sex	5.60	3.26 to 7.93	< 0.0001
hsCRP - average	-0.41	-3.16 to 2.31	0.76
hsCRP - high	-0.37	-3.88 to 3.08	0.83
Omega-3 index	-0.23	-1.61 to 1.13	0.73
baseline AD	1.93	-0.33 to 4.25	0.091
Recurrence	2.56	-0.08 to 5.17	0.057
Week:Omega-3 treatment	0.44	-0.21 to 1.08	0.18
Omega-3 index:Omega-3 treatment	0.25	-1.73 to 2.21	0.81
Week:Omega-3 index	0.05	-0.04 to 0.14	0.26
Week:Omega-3 index:Omega-3 treatment	-0.10	-0.24 to 0.03	0.13
	Time-to-Event		
Omega-3 treatment	1.22	0.82 to 1.78	0.34
Predicted CDRS-R	1.02	1 to 1.05	0.021

Table 15: Results from joint modeling of the primary outcome and time to dropout including an interaction term between group and sex. Study and intervention dropout were considered a single type of event.

Parameter	Estimate	CI	P
Longitudinal			
(Intercept)	43.88	32.65 to 55.24	< 0.0001
Week	-0.49	-0.58 to -0.4	< 0.0001
Omega-3 treatment	0.61	-3.49 to 4.68	0.76
Age	0.45	-0.2 to 1.1	0.17
Female sex	5.47	2.13 to 8.82	0.002
hsCRP - average	-0.42	-3.12 to 2.29	0.76
hsCRP - high	-0.33	-3.83 to 3.07	0.87
Omega-3 index	-0.09	-1.08 to 0.9	0.85
baseline AD	1.95	-0.3 to 4.23	0.087
Recurrence	2.58	-0.08 to 5.25	0.058
Week:Omega-3 treatment	-0.04	-0.16 to 0.08	0.51
Female sex:Omega-3 treatment	0.24	-4.46 to 4.92	0.92
Time-to-Event			
Omega-3 treatment	1.22	0.84 to 1.79	0.32
Predicted CDRS-R	1.03	1 to 1.05	0.016

Table 16: Results from joint modeling of the primary outcome and time to dropout including an interaction term between group and before or after Covid-19 pandemics (with cut-off date 16/03/2020). Study and intervention dropout were considered a single type of event.

Parameter	Estimate	CI	P
Longitudinal			
(Intercept)	45.21	34.16 to 55.87	< 0.0001
Week	-0.49	-0.58 to -0.4	< 0.0001
Omega-3 treatment	0.20	-2.24 to 2.63	0.88
Age	0.36	-0.26 to 0.97	0.27
Female sex	5.38	3.13 to 7.66	< 0.0001
hsCRP - average	-0.65	-3.25 to 2	0.63
hsCRP - high	-0.49	-3.87 to 2.99	0.77
Omega-3 index	-0.20	-1.17 to 0.77	0.69
baseline AD	2.04	-0.13 to 4.23	0.065
Recurrence	2.80	0.26 to 5.36	0.029
Covid-19 (after March 16 2020)	2.28	-0.84 to 5.35	0.15
Week:Omega-3 treatment	-0.04	-0.17 to 0.08	0.51
Covid-19 (after March 16 2020):Omega-3 treatment	1.76	-2.61 to 6.1	0.43
Time-to-Event			
Omega-3 treatment	1.22	0.83 to 1.79	0.34
Predicted CDRS-R	1.02	1 to 1.04	0.031

Table 17: Results from joint modeling of the primary outcome and time to dropout including an interaction term between group and influence of Covid-19 restrictions (between 16/03/2020 and 01/04/2022). Study and intervention dropout were considered a single type of event.

Parameter	Estimate	CI	P
Longitudinal			
(Intercept)	44.62	33.91 to 55.29	< 0.0001
Week	-0.50	-0.59 to -0.41	< 0.0001
Omega-3 treatment	0.45	-1.97 to 2.79	0.70
Age	0.40	-0.21 to 1	0.20
Female sex	5.55	3.24 to 7.76	< 0.0001
hsCRP - average	-0.49	-3.09 to 2.08	0.70
hsCRP - high	-0.47	-3.78 to 2.85	0.78
Omega-3 index	-0.15	-1.11 to 0.82	0.77
baseline AD	2.00	-0.16 to 4.14	0.067
Recurrence	2.63	0.12 to 5.14	0.039
Covid restrictions	1.09	-1.68 to 3.77	0.43
Week:Omega-3 treatment	-0.04	-0.17 to 0.09	0.51
Covid restrictions:Omega-3 treatment	0.91	-2.92 to 4.81	0.65
Time-to-Event			
Omega-3 treatment	1.21	0.82 to 1.78	0.35
Predicted CDRS-R	1.02	1 to 1.04	0.036

Table 18: Results from joint modeling of the primary outcome and time to dropout including an interaction term between group and anxiety/panic. Study and intervention dropout were considered a single type of event.

Parameter	Estimate	CI	P
Longitudinal			
(Intercept)	45.25	33.86 to 56.61	< 0.0001
Week	-0.49	-0.57 to -0.4	< 0.0001
Omega-3 treatment	0.89	-1.45 to 3.24	0.45
Age	0.39	-0.27 to 1.05	0.26
Female sex	5.41	2.91 to 7.86	< 0.0001
hsCRP - average	-0.27	-3.09 to 2.53	0.84
hsCRP - high	-0.28	-3.99 to 3.36	0.88
Omega-3 index	-0.21	-1.25 to 0.84	0.68
baseline AD	1.89	-0.49 to 4.25	0.11
Recurrence	2.24	-0.53 to 5.05	0.11
Anxiety	3.90	-1.84 to 9.57	0.18
Week:Omega-3 treatment	-0.04	-0.17 to 0.08	0.50
Anxiety:Omega-3 treatment	-1.38	-8.96 to 6.24	0.71
Time-to-Event			
Omega-3 treatment	1.22	0.83 to 1.79	0.32
Predicted CDRS-R	1.03	1.01 to 1.05	0.008

The results of the model with the pre-specified interaction term between treatment effect, time, and omega-3 index is shown in Table 14. In addition, Table 15 to Table 18 show results of not pre-specified analyses of other moderating effects. No evidence was found, that any of the investigated baseline variables acts as a treatment moderator.

4.4 Secondary endpoints

4.4.1 Confirmatory secondary endpoints

Remission

Table 19: Logistic regression of remission based on CDRS, adjusted for age, sex, hsCRP, antidepressants, Omega-3 Index and CDRS score at baseline (Delta-based multiple imputed data). The estimate is the odds ratio of the Omega-3 treatment group.

	Estimate	95%-confidence	interval	p-value
Delta = 0	0.61	0.18	2.06	0.42
25%	0.77	0.23	2.52	0.66
50%	0.70	0.22	2.21	0.54
75%	0.72	0.23	2.27	0.57
100%	0.70	0.22	2.19	0.54

Please note that these results based on delta imputation are not fully consistent.

Response

Table 20: Logistic regression of response based on CDRS, adjusted for age, sex, hsCRP, antidepressants, Omega-3 Index and CDRS score at baseline (Delta-based multiple imputed data). The estimate is the odds ratio of the Omega-3 treatment group.

	Estimate	95%-confidence	interval	p-value
Delta = 0	0.89	0.29	2.68	0.83
25%	0.84	0.28	2.50	0.75
50%	0.85	0.29	2.53	0.77
75%	0.85	0.29	2.52	0.77
100%	0.87	0.30	2.57	0.80

Intervention dropout (KM)

Figure 12 depicts the probability of avoiding additional antidepressant use over the study period per treatment arm. A log rank test was applied to test for differences in these curves, but no evidence of a difference in time until additional antidepressant use across treatment groups was detected (statistic = 1.6, p-value = 0.20).

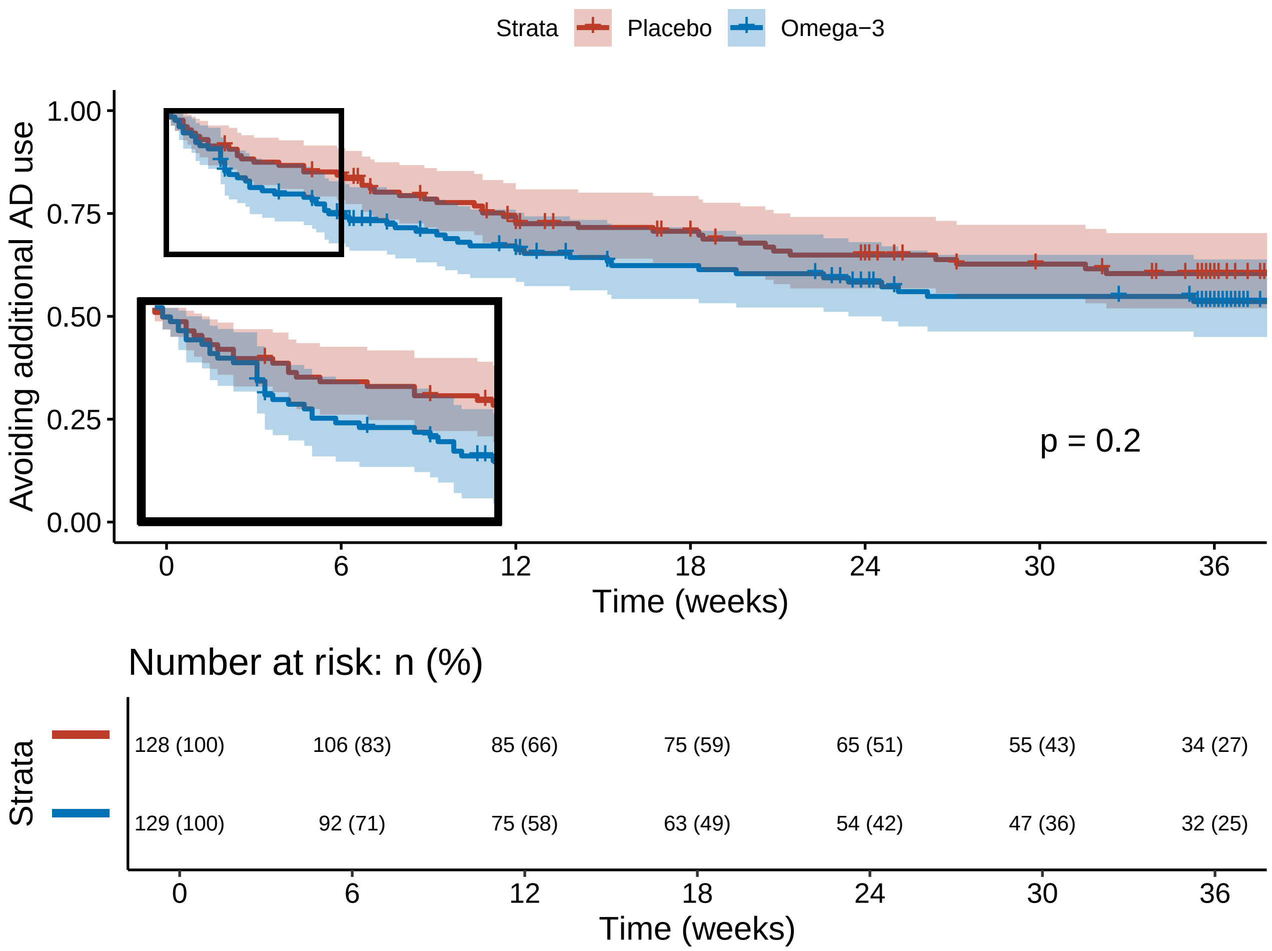

Figure 12: Kaplan Meier curves depicting probability of avoiding additional antidepressant use over time per treatment arm.

Table 21: Cox proportional hazards regression results for the relationship between treatment arm and time to additional antidepressant use.

	Hazard Ratio	95%-confidence interval	*p*-value
Omega-3 treatment	1.24	from 0.83 to 1.85	0.30
Age	1.03	from 0.90 to 1.16	0.69
Female sex	1.03	from 0.65 to 1.62	0.91
hsCRP - average	0.99	from 0.60 to 1.63	0.96
hsCRP - high	0.43	from 0.17 to 1.06	0.066
baseline AD	1.49	from 0.99 to 2.25	0.058
Omega3 index	1.02	from 0.84 to 1.24	0.83
Recurrence	1.17	from 0.73 to 1.88	0.51

A Cox proportional hazards model was also used to explore possible differences after adjusting for age, sex, hsCRP, baseline antidepressant use, and Omega-3 index. The estimate suggests a 24% increase in risk of additional antidepressant use for the Omega-3 arm compared to the placebo arm, however, this estimate is not statistically significant (HR (95% CI): 1.24, (0.83, 1.85), *p*-value = 0.30).

KidScreen-10

Table 22: Results from joint modeling of the outcome KidScreen 10 and time to dropout. Study and intervention dropout were considered a single type of event.

Parameter	Estimate	CI	P
**Longitudinal**			
(Intercept)	36.05	29.64 to 42.53	< 0.0001
Week	0.12	0.07 to 0.18	< 0.0001
Omega-3 treatment	-0.52	-1.77 to 0.75	0.42
Age	-0.33	-0.7 to 0.04	0.082
Female sex	-4.40	-5.76 to -3.03	< 0.0001
hsCRP - average	0.56	-0.99 to 2.13	0.48
hsCRP - high	0.04	-2.05 to 2.1	0.97
Omega3 index	0.44	-0.13 to 1.01	0.13
baseline AD	-0.17	-1.49 to 1.11	0.80
Recurrence	-0.38	-1.91 to 1.11	0.62
Week:Omega-3 treatment	0.00	-0.08 to 0.08	0.92
**Time-to-Event**			
Omega-3 treatment	1.26	0.85 to 1.88	0.26
Predicted KidScreen 10	0.97	0.92 to 1.02	0.27

Table 23: ANOVA of KIDscreen-10 after 36 weeks, adjusted for age, sex, hsCRP, antidepressants, Omega-3 Index and KIDscreen-10 at baseline. Only 240 patients included due to missing baseline KIDscreen-10.

	Estimate	95%-confidence interval	p-value
(Intercept)	36.35	[15.82, 56.88]	0.001
Omega-3 treatment	0.91	[-1.56, 3.38]	0.47
Age	-0.4	[-1.19, 0.39]	0.33
Female sex	-2.56	[-6.16, 1.03]	0.17
hsCRP - average	1.8	[-1.68, 5.28]	0.32
hsCRP - high	1.71	[-3.93, 7.34]	0.56
baseline AD	-0.67	[-3.94, 2.60]	0.69
Omega3 index	-0.25	[-1.58, 1.08]	0.71
baseline KIDscreen	0.41	[0.14, 0.68]	0.005
baseline CDRS	-0.1	[-0.25, 0.05]	0.20

Table 24: ANOVA of self-reported quality of life measured by the KIDscreen-10 at week 36, adjusted for age, sex, hsCRP, antidepressants, Omega-3 Index and KIDscreen-10 at baseline. Delta-based multiple imputation for MNAR analysis.

	Estimate	95%-confidence	interval	p-value
Delta = 0	0.91	-1.61	3.43	0.47
25%	1.59	-1.47	4.66	0.30
50%	2.28	-1.59	6.14	0.25
75%	2.96	-1.84	7.76	0.22
100%	3.64	-2.17	9.46	0.22

DIKJ

Table 25: Results from joint modeling of the outcome DIKJ and time to dropout. Study and intervention dropout were considered a single type of event.

Parameter	Estimate	CI	P
	Longitudinal		
(Intercept)	12.67	2.13 to 23.35	0.017
Week	-0.20	-0.27 to -0.13	< 0.0001
Omega-3 treatment	0.44	-1.64 to 2.54	0.68
Age	0.62	0 to 1.22	0.049
Female sex	7.82	5.53 to 10.13	< 0.0001
hsCRP - average	0.49	-2.1 to 3.14	0.72
hsCRP - high	1.03	-2.47 to 4.51	0.56
Omega3 index	-0.24	-1.2 to 0.72	0.62
baseline AD	0.41	-1.81 to 2.64	0.71
Recurrence	0.19	-2.41 to 2.76	0.89
Week:Omega-3 treatment	-0.02	-0.12 to 0.08	0.70
	Time-to-Event		
Omega-3 treatment	1.23	0.84 to 1.81	0.29
Predicted DIKJ	1.02	1 to 1.04	0.04

Table 26: ANOVA of DIKJ after 36 weeks, adjusted for age, sex, hsCRP, antidepressants, Omega-3 Index and DIKJ at baseline. Only 246 patients included due to missing baseline DIKJ.

	Estimate	95%-confidence interval	p-value
(Intercept)	-1.74	[-19.79, 16.30]	0.85
Omega-3 treatment	-0.9	[-4.09, 2.29]	0.58
Age	-0.32	[-1.31, 0.67]	0.53
Female sex	2.93	[-2.23, 8.09]	0.27
hsCRP - average	-2.26	[-7.00, 2.48]	0.35
hsCRP - high	-0.41	[-6.51, 5.69]	0.89
baseline AD	4.02	[0.08, 7.97]	0.051
Omega3 index	0.85	[-0.79, 2.50]	0.31
baseline DIKJ	0.44	[0.19, 0.70]	0.001
baseline CDRS	0.13	[-0.07, 0.33]	0.20

Table 27: ANOVA of depression severity measured by DIKJ at week 36, adjusted for age, sex, hsCRP, antidepressants, Omega-3 Index and DIKJ at baseline. Delta-based multiple imputation for MNAR analysis.

	Estimate	95%-confidence	interval	p-value
Delta = 0	-0.90	-4.15	2.34	0.58
25%	-0.76	-4.51	3.00	0.69
50%	-0.61	-4.99	3.76	0.78
75%	-0.47	-5.53	4.59	0.85
100%	-0.33	-6.12	5.47	0.91

4.4.2 Supportive secondary endpoints: Effect endpoints

Number of days in the hospital, Outpatient service use

Table 28: Effect endpoints. Mean and standard deviation of days under the respective treatment are shown.

	Overall	Placebo	Omega-3
n	257	128	129
total_ambulant (mean (SD))	167.9 (134.9)	172.0 (128.6)	163.8 (141.3)
total_stationär (mean (SD))	28.5 (54.9)	22.3 (43.3)	34.7 (63.9)
total_Tagesklinik (mean (SD))	8.0 (31.7)	8.8 (36.7)	7.1 (25.9)

Table 29: Effect endpoints. Median and range of days under the respective treatment are shown.

	Overall	Placebo	Omega-3
n	257	128	129
total_ambulant (median [range])	167.0 [0.0, 772.0]	177.0 [0.0, 646.0]	162.0 [0.0, 772.0]
total_stationär (median [range])	0.0 [0.0, 388.0]	0.0 [0.0, 250.0]	0.0 [0.0, 388.0]
total_Tagesklinik (median [range])	0.0 [0.0, 253.0]	0.0 [0.0, 253.0]	0.0 [0.0, 186.0]

There were 513 recordings of ambulant and stationary treatment. Thereof 26 were removed for the analyses, because there was not enough information or the treatment was ended before start of the study or started after the last visit.

4.4.3 Supportive secondary endpoints: Safety endpoints

SIQ-Jr

Table 30: Results from joint modeling of the outcome SIQ-Jr and time to study dropout. Study and intervention dropout were considered a single type of event.

Parameter	Estimate	CI	P
	Longitudinal		
(Intercept)	7.91	-16.04 to 32	0.52
Week	-0.33	-0.45 to -0.2	< 0.0001
Omega-3 treatment	2.02	-3.57 to 7.52	0.47
Age	0.99	-0.43 to 2.43	0.17
Female sex	9.42	3.7 to 15.15	0.0007
hsCRP - average	3.99	-2.33 to 10.42	0.22
hsCRP - high	-0.52	-8.78 to 7.81	0.89
Omega3 index	1.02	-1.29 to 3.43	0.40
baseline AD	4.14	-1.31 to 9.57	0.13
Recurrence	4.21	-2.14 to 10.51	0.18
Week:Omega-3 treatment	-0.09	-0.27 to 0.09	0.35
	Time-to-Event		
Omega-3 treatment	1.26	0.85 to 1.84	0.26
Predicted Jr-SIQ	1.01	1.01 to 1.02	0.001

Table 31: Safety analysis (censored observations after AD use): Adjusted linear mixed model results for the relationship between treatment arm and SIQ-Jr slopes. The model includes random intercepts for participant and center. This model did not result in convergence messages.

Variable	Estimate	X95.confidence.interval	p.value
(Intercept)	8.56	[-19.00, 36.13]	0.54
Week	-0.31	[-0.44, -0.18]	< 0.0001
Omega-3 treatment	1.83	[-3.40, 7.06]	0.49
Age	0.98	[-0.59, 2.55]	0.22
Female sex	8.93	[3.22, 14.64]	0.002
hsCRP - average	4.26	[-2.13, 10.65]	0.19
hsCRP - high	-0.56	[-8.86, 7.74]	0.89
Omega3 index	0.92	[-1.48, 3.32]	0.45
baseline AD	1.05	[-4.60, 6.69]	0.72
Recurrence	2.72	[-3.63, 9.06]	0.40
Week:Omega-3 treatment	-0.09	[-0.28, 0.10]	0.37

Table 32: Sensitivity analysis IV, Delta-based multiple imputation for MNAR analysis. Adjusted linear mixed model results for the relationship between treatment arm and siq slopes. The model includes random intercepts for participants. This model does not adjust for recurrence.

	Estimate	95%-confidence	interval	p-value
Delta = 0	2.00	-1.38	5.38	0.25
25%	2.01	-1.49	5.51	0.26
50%	2.01	-1.65	5.66	0.28
75%	2.01	-1.82	5.83	0.30
100%	2.01	-2.00	6.02	0.33

Adverse events

Table 33: Severe adverse events.

SAE Type		Overall	Placebo	Omega-3
n		76	31	45
Death (%)	No	76 (100.0)	31 (100.0)	45 (100.0)
	Yes	0 (0.0)	0 (0.0)	0 (0.0)
Life threat (%)	No	45 (59.2)	16 (51.6)	29 (64.4)
	Yes	31 (40.8)	15 (48.4)	16 (35.6)
Hospitalization (%)	No	18 (23.7)	9 (29.0)	9 (20.0)
	Yes	58 (76.3)	22 (71.0)	36 (80.0)
Disability (%)	No	76 (100.0)	31 (100.0)	45 (100.0)
	Yes	0 (0.0)	0 (0.0)	0 (0.0)
Other (%)	No	73 (96.1)	30 (96.8)	43 (95.6)
	Yes	3 (3.9)	1 (3.2)	2 (4.4)

Table 34: Adverse events.

		Overall	Placebo	Omega-3
n		115	71	44
Schweregrad (%)	Mild	74 (64.3)	45 (63.4)	29 (65.9)
	Moderate	38 (33.0)	23 (32.4)	15 (34.1)
	Severe	3 (2.6)	3 (4.2)	0 (0.0)
Massnahmen? (%)	No	60 (52.2)	37 (52.1)	23 (52.3)
	Yes	55 (47.8)	34 (47.9)	21 (47.7)
Med. Behandlung? (%)	No	24 (43.6)	17 (50.0)	7 (33.3)
	Yes	31 (56.4)	17 (50.0)	14 (66.7)

During the study, there were 191 events in 97 patients: 76 severe adverse events and 115 adverse events. Of the 76 severe adverse events, 31 occurred in the placebo arm and 45 in the Omega-3 arm (Table 33). There were 0 deaths or disabilities, however, 31 events were considered life-threatening and 58 required hospitalization. Four of these were not related to depression or suicidality (breathing problems due to influenza, seizure, paraparesis after lumbar spine trauma, hand fracture).

Twenty-eight were suicide attempts. The 3 events categorized as 'Other' included an increase in CDRS-R for suicidality from 1 to 4, isolation in acute care, and crisis intervention in the Psychiatric Emergency center in Zurich (KJPP). The investigators did not find any severe adverse events to be causally related to study treatment. Of the 115 adverse events, 71 occurred in the placebo arm and 44 in the Omega-3 arm (Table 34). Most adverse events were considered mild (64.3%). Measures were taken in 55 events (47.8%), of which 31 were treated medically. No causal relationship to study treatment was determined for 88 adverse events, but a connection was possible for 22 adverse events.

ASEC

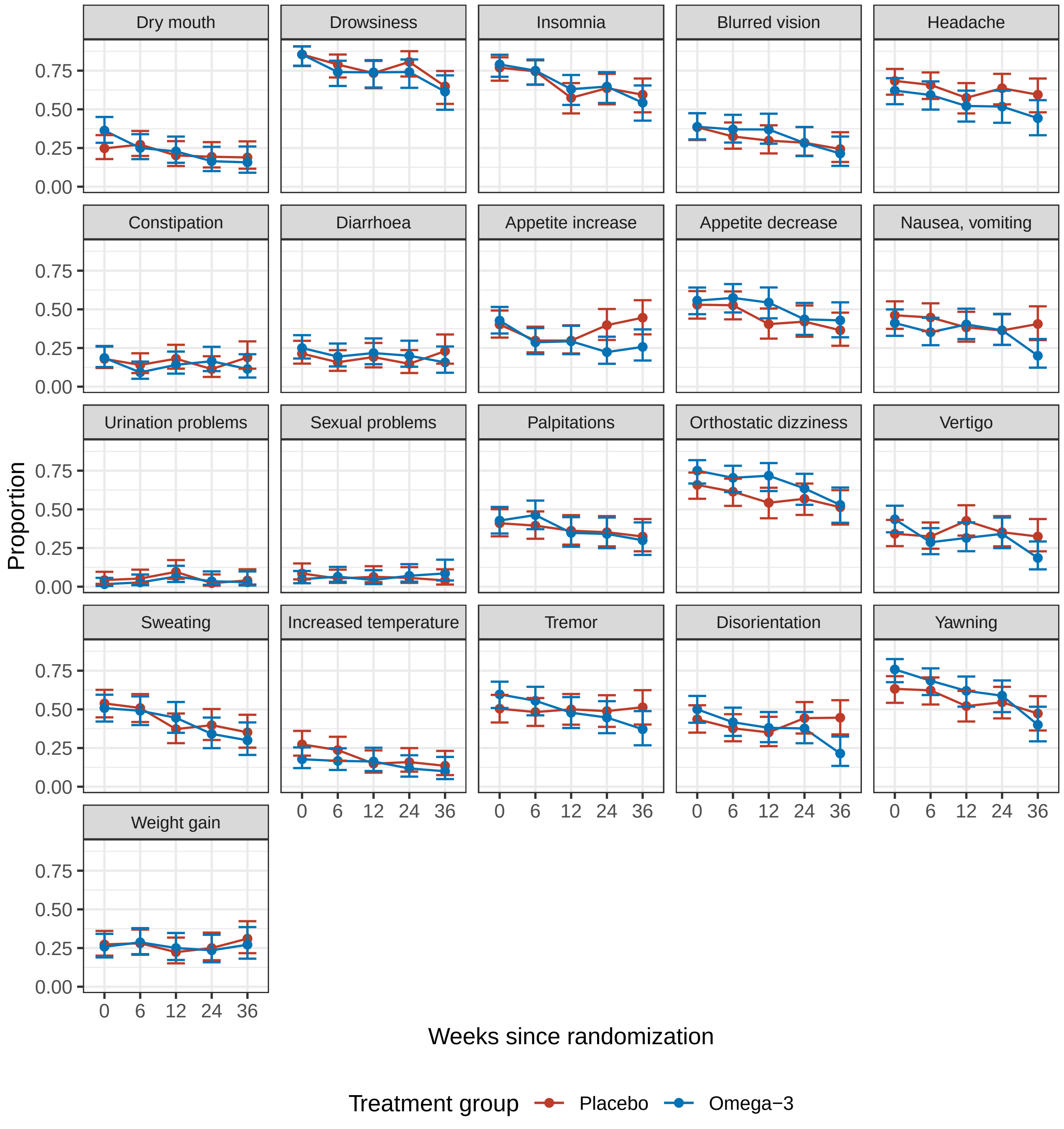

Figure 13: Proportion of patients with ASEC symptoms per treatment group over time along with 95% Wilson confidence intervals.

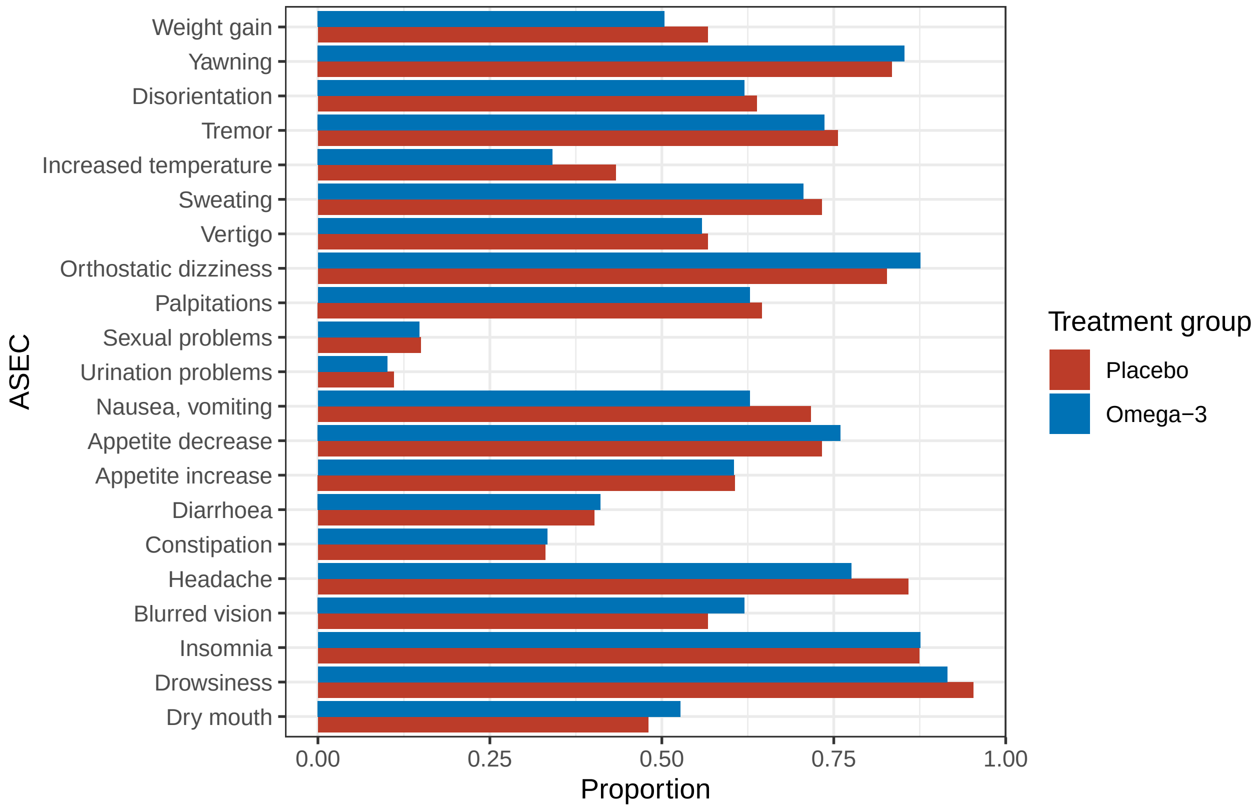

Figure 14: Proportion of patients with ASEC symptoms at least once during the study per treatment group. Total of Omega-3 group: 129, total of Placebo group: 127 (1 patient had missing ASEC data).

4.4.4 Compliance

Fatty acid levels

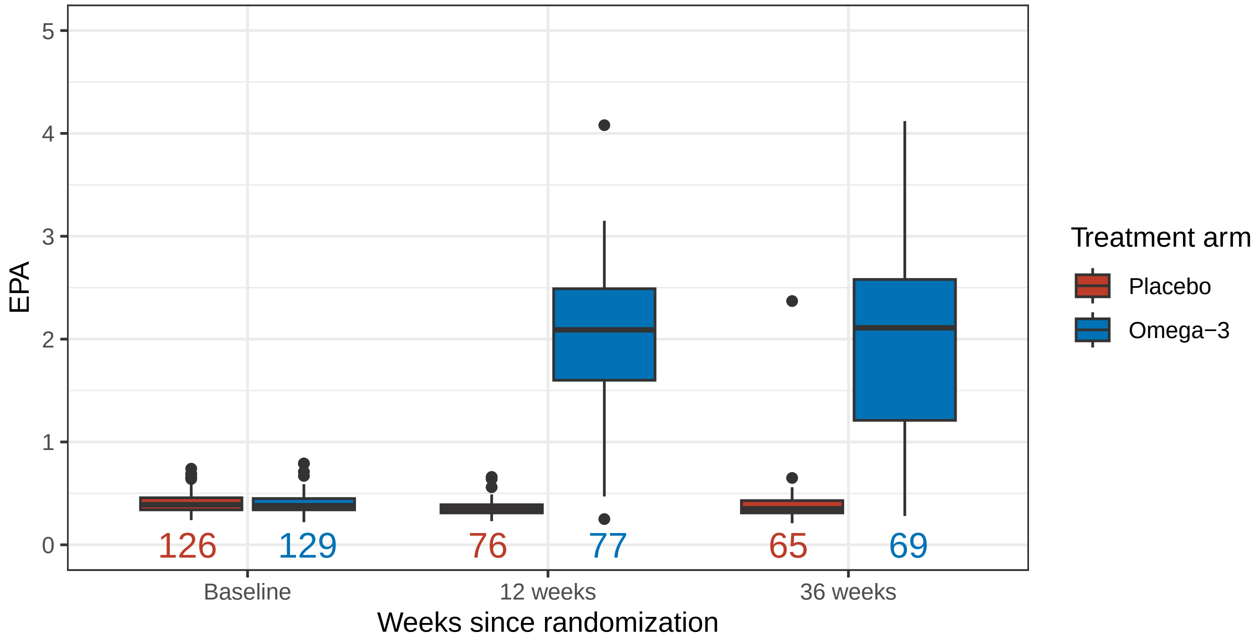

Figure 15: EPA levels per treatment arm (data as observed). Numbers of observations are shown.

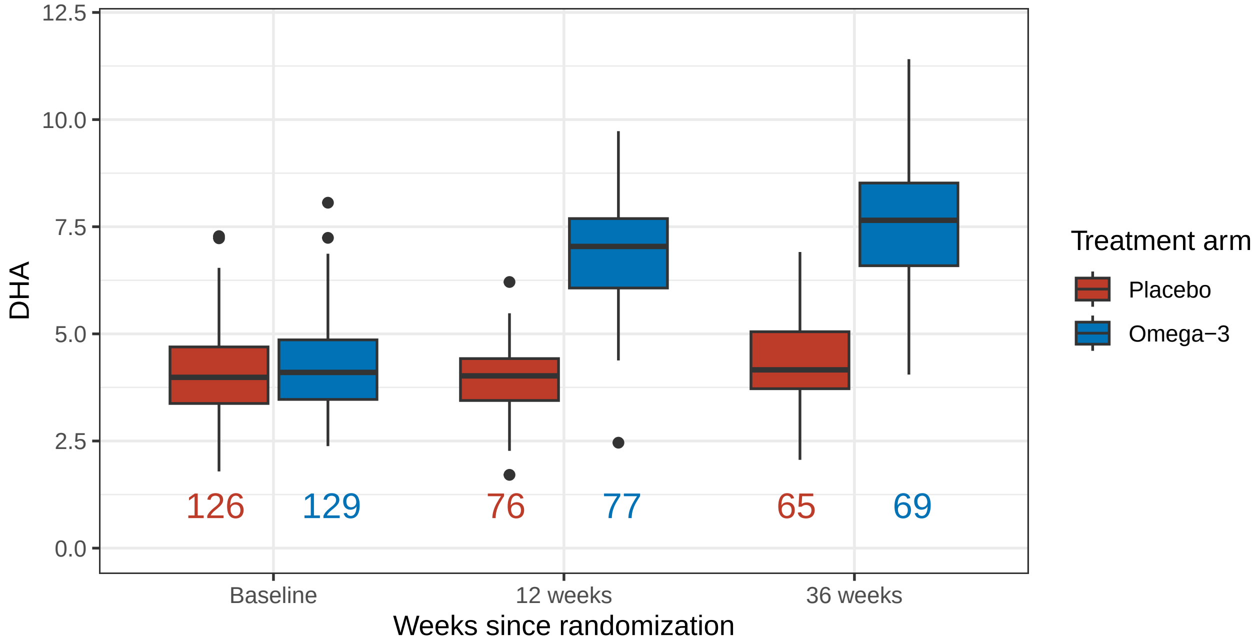

Figure 16: DHA levels per treatment arm (data as observed). Numbers of observations are shown.

]

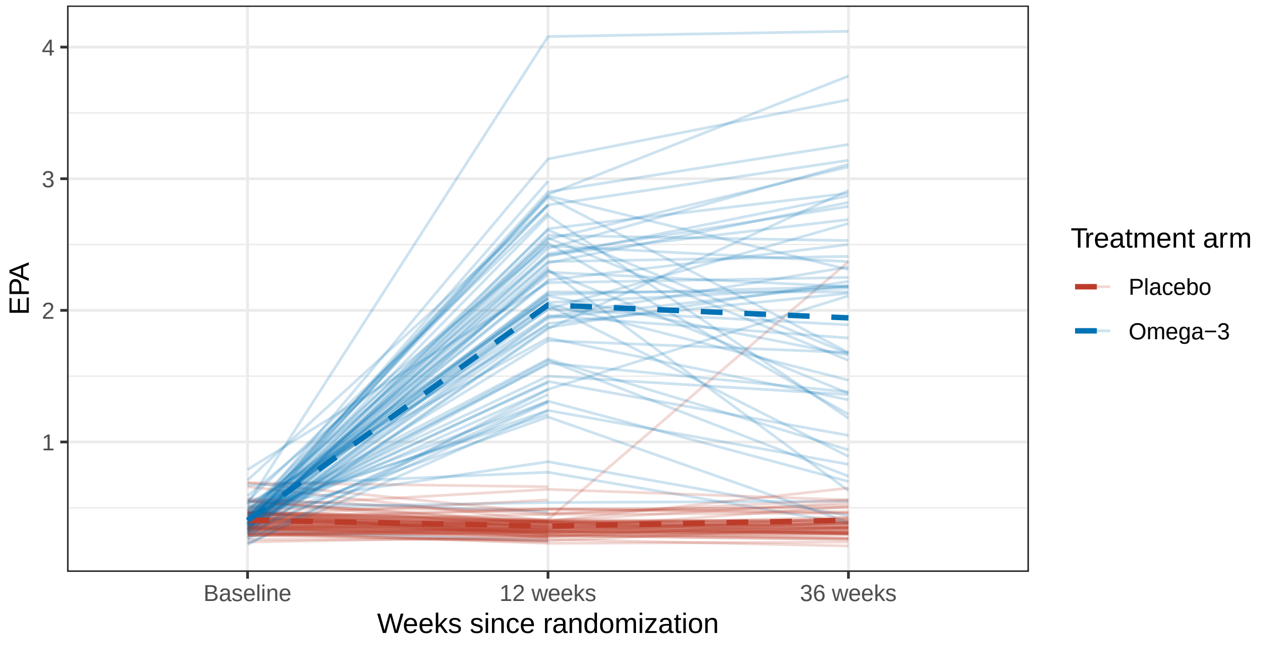

Figure 17: Individual EPA trajectories with mean trajectories per treatment arm in bold (data as observed).

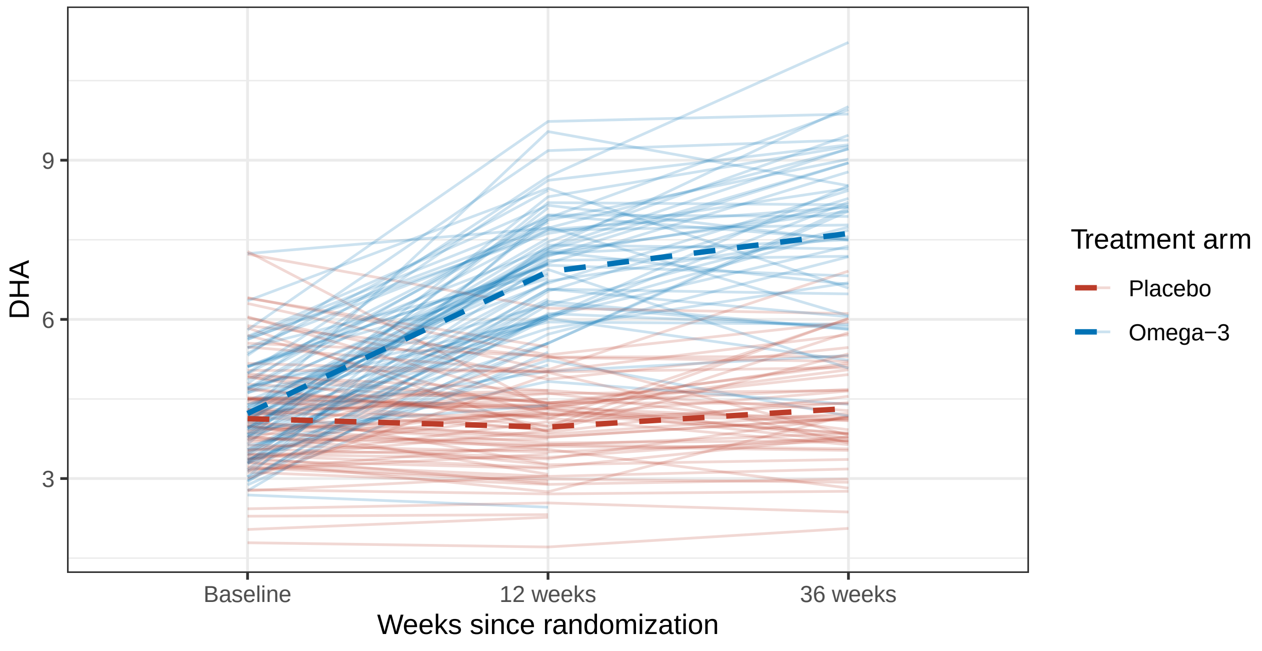

Figure 18: Individual DHA trajectories with mean trajectories per treatment arm in bold (data as observed).

```
## Error:  <text>:90:13:  unexpected symbol
## 89:                n = sum(!is.na(EPA_V2))
## 90:                mean_EPA_V3
```

^

Pillcounts

Figure 19 and 20 show the compliance rate measured by the percentage of medication taken since the last visit.

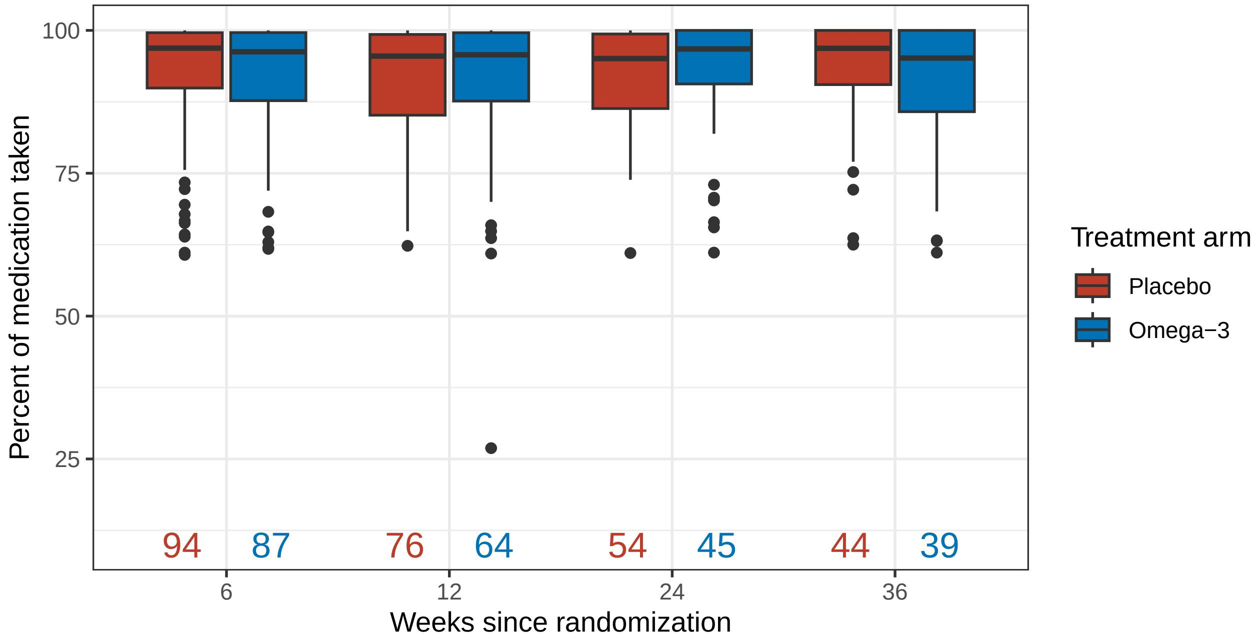

Figure 19: Pillcounts percentages per treatment arm (censored observations after study or intervention dropout). Numbers of observations are shown.

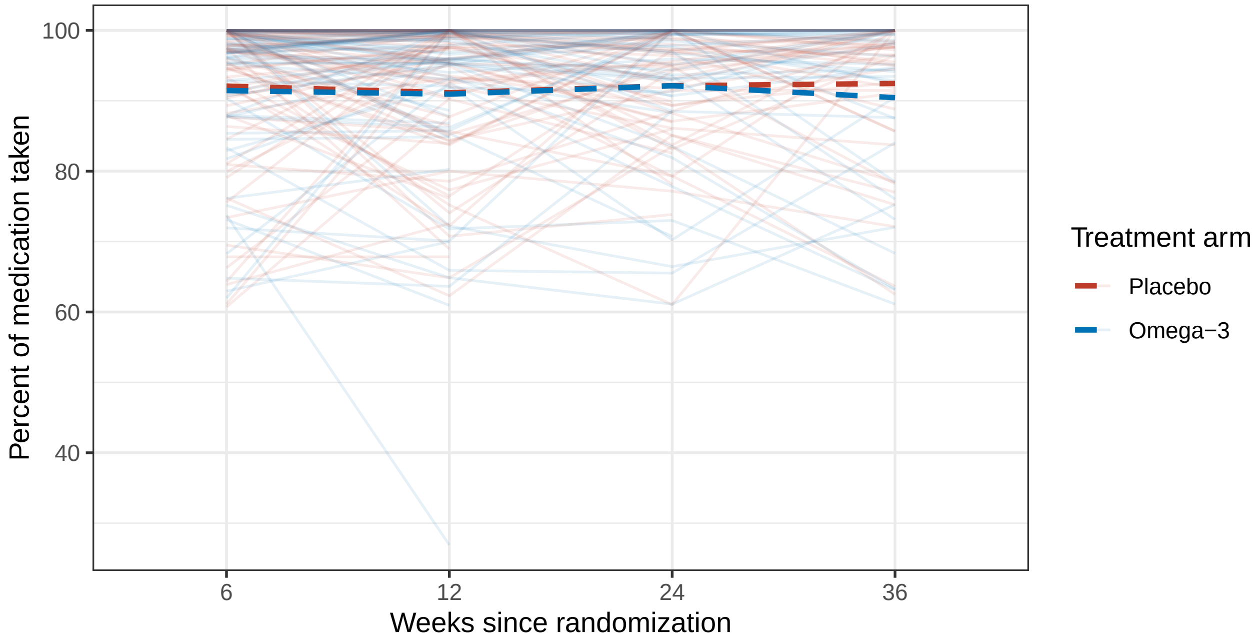

Figure 20: Individual pillcounts trajectories with mean trajectories per treatment arm in bold (censored observations after study or intervention dropout).

5 References

R Core Team (2018). *R: A Language and Environment for Statistical Computing*. R Foundation for Statistical Computing, Vienna, Austria.
URL https://www.R-project.org/

R version and packages used to generate this report:

R version: R version 4.2.2 (2022-10-31 ucrt)

Base packages: splines, stats, graphics, grDevices, utils, datasets, methods, base

Other packages: consort 1.2.0, secuTrialR 1.1.1, epitools 0.5-10.1, haven 2.5.3, biostatUZH 1.8.0, stringr 1.5.0, english 1.2-6, survminer 0.4.9, ggpubr 0.6.0, JMbayes2 0.4-5, GLMMadaptive 0.9-0, nlme 3.1-162, survival 3.4-0, mice 3.16.0, lme4 1.1-34, Matrix 1.6-0, Epi 2.47.1, logbin 2.0.5, ggplot2 3.5.1, tableone 0.13.2, xtable 1.8-4, lubridate 1.9.2, tidyr 1.3.0, dplyr 1.1.2, readxl 1.4.3, knitr 1.43

This document was generated on October 21, 2024 at 21:46.

University of Zurich[UZH]

Epidemiology, Biostatistics
and Prevention Institute,
Biostatistics Department
www.biostat.uzh.ch

Omega-3 Fatty Acids as First-Line Treatment in Pediatric Depression: A Randomized Placebo-Controlled Trial

Analysis for Gregor Berger, Isabelle Häberling

Kelly Reeve, Priska Heinz (*priska.heinz@uzh.ch*)
Ulrike Held (*ulrike.held@uzh.ch*)

Version - Amendment of November 11, 2024

DISCLAIMER

This report was written by the Research Methods Consulting Unit of the University of Zurich, Epidemiology, Biostatistics and Prevention Institute, Biostatistics Department (EBPI). Any citation of results or comments (or parts thereof) given in this report in any format (including: paper, presentation, abstract, grant application) or the naming of EBPI or persons of the EBPI requires the explicit approval of the senior statistician (Ulrike Held). This is especially true for citations in the "acknowledgments".

1 Results

1.1 Descriptive statistics: Baseline characteristics

Table 1 depicts the patients' baseline characteristics.

Table 1: Baseline characteristics. Mean (standard deviation) for continuous variables and number (percentage) for categorical variables are shown. Anxiety/panic disorders here are without specific phobias.

**Characteristic**	**Levels**	**Overall**	**Placebo**	**Omega-3**	**Missing (%)**
n		257	128	129	
Age		15.66 (1.66)	15.64 (1.74)	15.69 (1.58)	0.0
Sex	M	69 (26.8)	34 (26.6)	35 (27.1)	0.0
	W	188 (73.2)	94 (73.4)	94 (72.9)	
BMI		22.38 (5.28)	22.11 (4.52)	22.65 (5.95)	9.3
hsCRP	Low	179 (69.6)	90 (70.3)	89 (69.0)	0.0
	Average	53 (20.6)	25 (19.5)	28 (21.7)	
	High	25 (9.7)	13 (10.2)	12 (9.3)	
Omega 3 Index		4.58 (1.09)	4.54 (1.10)	4.63 (1.08)	0.0
Recurrent episode	No	201 (78.2)	107 (83.6)	94 (72.9)	0.0
	Yes	56 (21.8)	21 (16.4)	35 (27.1)	
Age of onset		13.79 (2.38)	13.93 (2.27)	13.65 (2.49)	0.0
Duration of previous episodes		16.28 (17.14)	16.76 (18.98)	15.79 (15.12)	2.3
Anxiety/panic	No	233 (90.7)	118 (92.2)	115 (89.1)	0.0
	Yes	24 (9.3)	10 (7.8)	14 (10.9)	
Attention disorder	No	220 (85.6)	112 (87.5)	108 (83.7)	0.0
	Yes	37 (14.4)	16 (12.5)	21 (16.3)	
Psychotic features	No	245 (95.3)	121 (94.5)	124 (96.1)	0.0
	Yes	12 (4.7)	7 (5.5)	5 (3.9)	
Antidepressant use	No	167 (65.0)	81 (63.3)	86 (66.7)	0.0
	Yes	90 (35.0)	47 (36.7)	43 (33.3)	
Antidepressant name	Escitalopram	11 (12.2)	6 (12.8)	5 (11.6)	
	Fluoxetin	42 (46.7)	16 (34.0)	26 (60.5)	
	Other	7 (7.8)	5 (10.6)	2 (4.7)	
	Sertralin	30 (33.3)	20 (42.6)	10 (23.3)	
CDRS		58.54 (8.78)	58.13 (8.42)	58.95 (9.15)	0.0
DIKJ		28.09 (9.46)	28.12 (9.91)	28.06 (9.06)	4.3
KidScreen 10		29.05 (5.72)	29.25 (5.74)	28.87 (5.71)	6.6
SIQ-Jr		40.91 (23.49)	39.80 (24.31)	41.95 (22.73)	6.6
EPA		0.40 (0.10)	0.41 (0.10)	0.40 (0.10)	0.8
DHA		4.18 (1.04)	4.13 (1.05)	4.23 (1.04)	0.8
Center	SG Out-patient	32 (12.5)	17 (13.3)	15 (11.6)	0.0
	SG Ganterschwil	29 (11.3)	14 (10.9)	15 (11.6)	
	TG Out-patient	17 (6.6)	10 (7.8)	7 (5.4)	
	TG Littenheid	55 (21.4)	27 (21.1)	28 (21.7)	
	BL	12 (4.7)	6 (4.7)	6 (4.7)	
	BS	18 (7.0)	7 (5.5)	11 (8.5)	
	ZH	94 (36.6)	47 (36.7)	47 (36.4)	
Clinic type	In-patient	80 (31.1)	39 (30.5)	41 (31.8)	0.0
	Out-patient	164 (63.8)	82 (64.1)	82 (63.6)	
	Day unit	13 (5.1)	7 (5.5)	6 (4.7)	

1.1.1 Compliance

Fatty acid levels

The tables below show the average levels of the fatty acids in patients with data available.

Table 2: Change in Fatty acids between baseline and 12 weeks in percentage points. Mean and standard deviation (in brackets) shown.

	**Placebo (n = 74)**			**Omega-3 (n = 77)**		
	week 0	week 12	change	week 0	week 12	change
EPA	0.40 (0.09)	0.36 (0.08)	-0.04 (0.08)	0.41 (0.10)	2.04 (0.67)	1.63 (0.67)
DHA	4.22 (1.13)	3.97 (0.84)	-0.25 (0.70)	4.20 (0.91)	6.90 (1.20)	2.70 (1.03)
Omega-3 index	4.62 (1.18)	4.33 (0.88)	-0.29 (0.73)	4.61 (0.95)	8.94 (1.71)	4.33 (1.54)

Table 3: Change in Fatty acids between baseline and 36 weeks in percentage points. Mean and standard deviation (in brackets) are shown.

	**Placebo (n = 64)**			**Omega-3 (n = 69)**		
	week 0	week 36	change	week 0	week 36	change
EPA	0.41 (0.10)	0.40 (0.27)	-0.01 (0.26)	0.40 (0.11)	1.94 (0.96)	1.54 (0.96)
DHA	4.40 (1.10)	4.32 (1.02)	-0.08 (0.83)	4.28 (1.00)	7.62 (1.58)	3.34 (1.60)
Omega-3 index	4.81 (1.15)	4.72 (1.13)	-0.09 (0.94)	4.68 (1.04)	9.56 (2.38)	4.88 (2.38)

R version and packages used to generate this report:

R version: R version 4.2.2 (2022-10-31 ucrt)

Base packages: splines, stats, graphics, grDevices, utils, datasets, methods, base

Other packages: gtsummary 1.7.2, secuTrialR 1.1.1, epitools 0.5-10.1, haven 2.5.3, biostatUZH 1.8.0, stringr 1.5.0, english 1.2-6, survminer 0.4.9, ggpubr 0.6.0, JMbayes2 0.4-5, GLMMadaptive 0.9-0, nlme 3.1-162, survival 3.4-0, mice 3.16.0, lme4 1.1-34, Matrix 1.6-0, Epi 2.47.1, logbin 2.0.5, ggplot2 3.5.1, tableone 0.13.2, xtable 1.8-4, lubridate 1.9.2, tidyr 1.3.0, dplyr 1.1.2, readxl 1.4.3, knitr 1.43

This document was generated on November 11, 2024 at 20:50.

Epidemiology, Biostatistics
and Prevention Institute,
Biostatistics Department
www.biostat.uzh.ch

Omega-3 Fatty Acids as First-Line Treatment in Pediatric Depression: A Randomized Placebo-Controlled Trial

Analysis for Gregor Berger, Isabelle Häberling

Kelly Reeve, Priska Heinz (*priska.heinz@uzh.ch*)
Ulrike Held (*ulrike.held@uzh.ch*)

Version Amendment 2 of August 4, 2025

DISCLAIMER

This report was written by the Research Methods Consulting Unit of the University of Zurich, Epidemiology, Biostatistics and Prevention Institute, Biostatistics Department (EBPI). Any citation of results or comments (or parts thereof) given in this report in any format (including: paper, presentation, abstract, grant application) or the naming of EBPI or persons of the EBPI requires the explicit approval of the senior statistician (Ulrike Held). This is especially true for citations in the "acknowledgments".

1 Results

1.1 Descriptive statistics: Continuous outcome trajectories

Most of the participants were 13 years or older (age below 12: n = 6, age below 13: n = 14, age 13 years or older: n = 243).

Table 1 and 2 give the mean outcome measurement per treatment arm and age group for each visit (KidScreen was not collected at the 6 week and 24 week visits).

Table 1: Continuous primary and secondary outcome measurements over time (data censored at non-compliance or additional antidepressant use) for participants with 13 years or older.

	Baseline	6 weeks	12 weeks	24 weeks	36 weeks
			CDRS		
Placebo	58.7 (8.21)	49.72 (11.77)	46.84 (12.85)	43.13 (13.86)	37.47 (15.5)
n Placebo	122	96	75	55	45
Omega-3	59.3 (9.28)	49.6 (12.3)	46.1 (11.98)	40.5 (14.07)	36.93 (12.99)
n Omega-3	121	88	68	50	41
			DIKJ		
Placebo	28.75 (9.72)	26.05 (9.4)	23.61 (11.09)	21.3 (11.69)	18.9 (12.01)
n Placebo	114	93	66	47	40
Omega-3	28.73 (8.79)	26.28 (11.07)	22.18 (9.28)	20.09 (11.22)	18.22 (10.97)
n Omega-3	118	79	62	44	36
			KidScreen		
Placebo	29.03 (5.68)		31.95 (7.14)		34.54 (8.37)
n Placebo	111		62		39
Omega-3	28.44 (5.47)		32.38 (5.91)		33.86 (6.87)
n Omega-3	117		60		35
			SIQ-Jr		
Placebo	40.79 (24.06)	32.92 (20.54)	30.53 (23.44)	29 (23.77)	23.93 (22.53)
n Placebo	112	91	68	49	41
Omega-3	43.28 (21.5)	34.25 (21.35)	28.25 (17.02)	28.04 (22.8)	21.47 (17.89)
n Omega-3	116	81	61	46	34

Table 2: Continuous primary and secondary outcome measurements over time (data censored at non-compliance or additional antidepressant use) for participants with under 13 years.

	Baseline	6 weeks	12 weeks	24 weeks	36 weeks
			CDRS		
Placebo	46.67 (1.86)	32.5 (4.8)	31.75 (5.38)	29.33 (3.21)	22.5 (0.71)
n Placebo	6	4	4	3	2
Omega-3	53.62 (4.31)	40.5 (6.36)	34 (NA)	27 (NA)	19 (NA)
n Omega-3	8	2	1	1	1
			DIKJ		
Placebo	16.17 (4.54)	13.25 (5.91)	10.33 (5.86)	8 (7.81)	4.5 (2.12)
n Placebo	6	4	3	3	2
Omega-3	18.25 (7.52)	9.5 (0.71)	NaN (NA)	6 (NA)	5 (NA)
n Omega-3	8	2	0	1	1
			KidScreen		
Placebo	35.5 (4.2)		38.67 (1.53)		41.5 (6.36)
n Placebo	4		3		2
Omega-3	35.25 (5.57)		NaN (NA)		49 (NA)
n Omega-3	8		0		1
			SIQ-Jr		
Placebo	12 (13.24)	7.75 (6.13)	6.67 (5.77)	3.67 (3.21)	3 (4.24)
n Placebo	4	4	3	3	2
Omega-3	22.62 (32.12)	0 (0)	NaN (NA)	0 (NA)	0 (NA)
n Omega-3	8	2	0	1	1

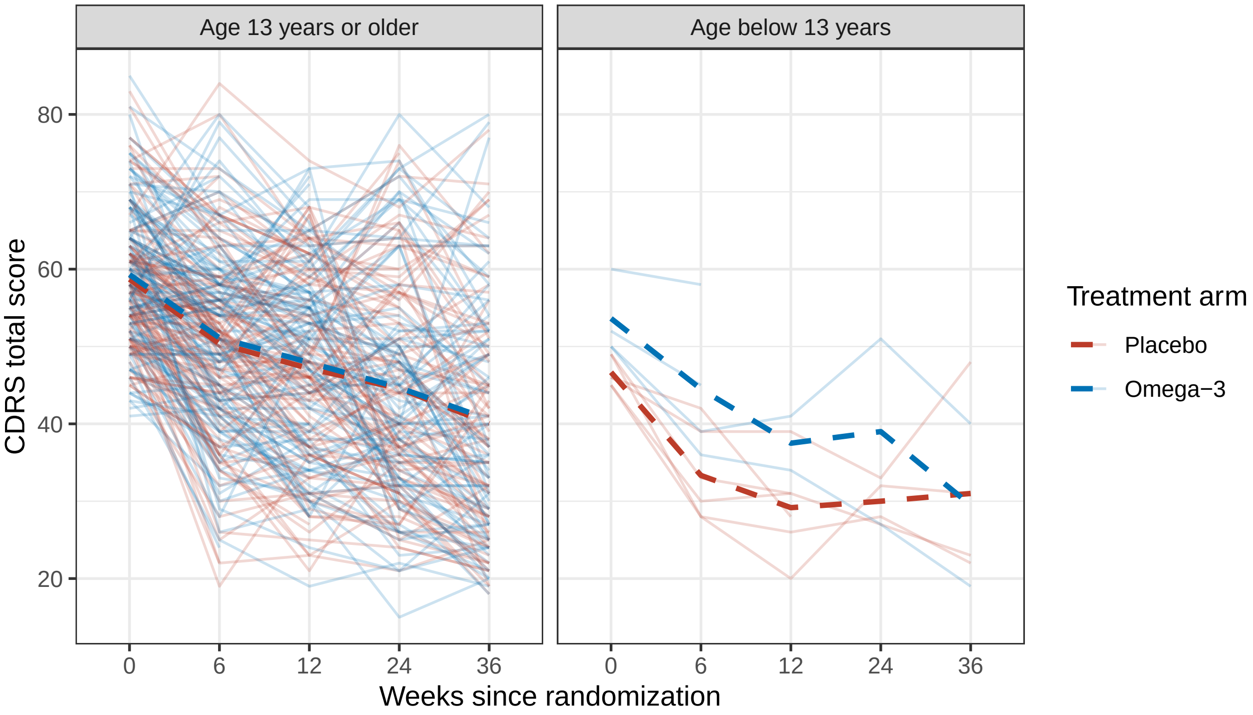

Figure 1: Individual CDRS trajectories with mean trajectories per treatment arm in bold for those above 13 years and those below (until 12.99 years), separately (data as observed).

1.1.1 Subgroup analysis for the primary endpoint

Evaluation of moderating effects of the treatment and different baseline characteristics.

Table 3: Results from joint modeling of the primary outcome and time to dropout including an interaction term between group and age (above vs. below 13 years). Study and intervention dropout were considered a single type of event.

Parameter	Estimate	CI	P
	Longitudinal		
(Intercept)	51.88	46.5 to 57.32	< 0.0001
Week	-0.48	-0.58 to -0.39	< 0.0001
Omega-3 treatment	0.47	-1.84 to 2.78	0.69
Female sex	4.83	2.22 to 7.44	0.0007
hsCRP - average	-0.56	-3.41 to 2.32	0.71
hsCRP - high	-0.36	-4.08 to 3.3	0.85
Omega-3 index	-0.06	-1.12 to 0.98	0.91
baseline AD	1.90	-0.54 to 4.35	0.12
Recurrence	2.34	-0.44 to 5.17	0.096
Age below 13	-9.20	-16.81 to -1.56	0.018
Week:Omega-3 treatment	-0.04	-0.17 to 0.09	0.57
Age below 13:Omega-3 treatment	8.22	-1.89 to 18.4	0.11
Omega-3 treatment	1.22	0.83 to 1.79	0.33
	Time-to-Event		
Predicted CDRS-R	1.03	1.01 to 1.05	0.006

R version and packages used to generate this report:

R version: R version 4.5.1 (2025-06-13 ucrt)

Base packages: splines, stats, graphics, grDevices, utils, datasets, methods, base

Other packages: secuTrialR 1.3.3, epitools 0.5-10.1, haven 2.5.5, biostatUZH 1.8.0, stringr 1.5.1, english 1.2-6, survminer 0.5.0, ggpubr 0.6.0, JMbayes2 0.5-7, GLMMadaptive 0.9-7, nlme 3.1-168, survival 3.8-3, mice 3.18.0, lme4 1.1-37, Matrix 1.7-3, Epi 2.60, logbin 2.0.6, ggplot2 3.5.2, tableone 0.13.2, xtable 1.8-4, lubridate 1.9.4, tidyr 1.3.1, dplyr 1.1.4, readxl 1.4.5, knitr 1.50

This document was generated on August 04, 2025 at 10:48.
