# Supplementary material for: ω-3 Fatty Acids in Pediatric Major Depressive Disorder: A Randomized Clinical Trial
Source: JAMA Netw Open. 2026 Jan 2;9(1):e2548703. doi: 10.1001/jamanetworkopen.2025.48703 (PMC12761337; doi:10.1001/jamanetworkopen.2025.48703)
Supplement: Supplement 2. — eTable 1. Participants Retention Rate eTable 2. Primary Analysis Results From Joint Modeling of the Primary Outcome and Time to Dropout eTable 3. Sensitivity Analysis I (Data as Observed): Adjusted Linear Mixed Model Results for the Relationship Between Treatment Arm and CDRS Slopes, Including Random Intercepts for Participant and Center eTable 4. Sensitivity Analysis I (Data as Observed): Adjusted Linear Mixed Model Results for the Relationship Between Treatment Arm and CDRS Slopes, Including Random Intercepts for Participant and Center and Random Slopes for Participants eTable 5. Sensitivity Analysis II (Censored Observations After AD Use): Adjusted Linear Mixed Model Results for the Relationship Between Treatment Arm and CDRS Slopes, Including Random Intercepts for Participant and Center eTable 6. Sensitivity Analysis II (Censored Observations After AD Use): Adjusted LLMl results for the Relationship Between Treatment Arm and CDRS Slopes, Including Random Intercepts for Participant and Center and Random Slopes for Participants eTable 7. Sensitivity Analysis III (per Protocol Data): Adjusted Linear Mixed Model Results for the Relationship Between Treatment Arm and CDRS Slopes, Including Random Intercepts for Participant and Center eTable 8. Sensitivity Analysis III (per Protocol Data): Adjusted LMM Results for the Relationship Between Treatment Arm and CDRS Slopes, Including Random Intercepts for Participants and Center and Random Slopes for Participants eTable 9. Sensitivity Analysis IV: Delta-Based Multiple Imputation for MNAR Analysis eTable 10. Remission Status per Visit eTable 11. Response Status per Visit eTable 12. Results From Joint Modeling of the Outcome KIDSCREEN-CAT-10 and Time to Dropout eTable 13. ANOVA of KIDSCREEN-CAT-10 After 36 Weeks, Adjusted for Age, Sex, hsCRP, Antidepressants, ω-3 Index, and KIDSCREEN-CAT-10 at Baseline eTable 14. ANOVA of Self-Reported Quality of Life Measured by the KIDSCREEN-CAT-10 at Week 36, Adjusted for Age, Sex, hsCRP, [file jamanetwopen-e2548703-s002.pdf]

Supplementary Online Content

Berger G, Häberling I, Emery S, et al; Omega-3 pMDD Study Group. ω-3 Fatty acids in pediatric major depressive disorder: a randomized clinical trial. *JAMA Netw Open.* 2025;8(12):e2548703. doi:10.1001/jamanetworkopen.2025.48703

eTable 1. Participants Retention Rate

eTable 2. Primary Analysis Results From Joint Modeling of the Primary Outcome and Time to Dropout

eTable 3. Sensitivity Analysis I (Data as Observed): Adjusted Linear Mixed Model Results for the Relationship Between Treatment Arm and CDRS Slopes, Including Random Intercepts for Participant and Center

eTable 4. Sensitivity Analysis I (Data as Observed): Adjusted Linear Mixed Model Results for the Relationship Between Treatment Arm and CDRS Slopes, Including Random Intercepts for Participant and Center and Random Slopes for Participants

eTable 5. Sensitivity Analysis II (Censored Observations After AD Use): Adjusted Linear Mixed Model Results for the Relationship Between Treatment Arm and CDRS Slopes, Including Random Intercepts for Participant and Center

eTable 6. Sensitivity Analysis II (Censored Observations After AD Use): Adjusted LLMI results for the Relationship Between Treatment Arm and CDRS Slopes, Including Random Intercepts for Participant and Center and Random Slopes for Participants

eTable 7. Sensitivity Analysis III (per Protocol Data): Adjusted Linear Mixed Model Results for the Relationship Between Treatment Arm and CDRS Slopes, Including Random Intercepts for Participant and Center

eTable 8. Sensitivity Analysis III (per Protocol Data): Adjusted LMM Results for the Relationship Between Treatment Arm and CDRS Slopes, Including Random Intercepts for Participants and Center and Random Slopes for Participants

eTable 9. Sensitivity Analysis IV: Delta-Based Multiple Imputation for MNAR Analysis

eTable 10. Remission Status per Visit

eTable 11. Response Status per Visit

eTable 12. Results From Joint Modeling of the Outcome KIDSCREEN-CAT-10 and Time to Dropout

eTable 13. ANOVA of KIDSCREEN-CAT-10 After 36 Weeks, Adjusted for Age, Sex, hsCRP, Antidepressants, ω-3 Index, and KIDSCREEN-CAT-10 at Baseline

eTable 14. ANOVA of Self-Reported Quality of Life Measured by the KIDSCREEN-CAT-10 at Week 36, Adjusted for Age, Sex, hsCRP, Antidepressants, ω-3 Index, and KIDSCREEN-CAT-10 at Baseline

eTable 15. Results From Joint Modeling of the Outcome DIKJ and Time to Dropout

© 2025 Berger G et al. *JAMA Network Open*.

eTable 16. ANOVA of DIKJ After 36 Weeks, Adjusted for Age, Sex, hsCRP, Antidepressants, ω-3 Index, and DIKJ at Baseline

eTable 17. ANOVA of Depression Severity Measured by DIKJ at Week 36, Adjusted for Age, Sex, hsCRP, Antidepressants, ω-3 Index, and DIKJ at Baseline

eTable 18. Cox Proportional Hazards Regression Results for the Relationship Between Treatment Arm and Time to Additional Antidepressant Use

eTable 19. Results From Joint Modeling of the Primary Outcome and Time to Dropout, Including an Interaction Term Between Among Treatment Effect, Time, and ω-3 Index

eTable 20. Results From Joint Modeling of the Primary Outcome and Time to Dropout, Including an Interaction Term Between Group and Sex

eTable 21. Results From Joint Modeling of the Primary Outcome and Time to Dropout, Including an Interaction Term Between Group and Before or After COVID-19 Pandemic

eTable 22. Results From Joint Modeling of the Primary Outcome and Time to Dropout, Including an Interaction Term Between Group and Influence of COVID-19 Restrictions

eTable 23. Results From Joint Modeling of the Primary Outcome and Time to Dropout, Including an Interaction Term Between Group and Anxiety and/or Panic

eTable 24. Results From Joint Modeling of the Outcome SIQ-Jr and Time to Study Dropout

eTable 25. Safety Analysis (Censored Observations After AD Use): Adjusted Linear Mixed Model Results for the Relationship Between Treatment Arm and SIQ-Jr Slopes

eTable 26. Sensitivity Analysis IV, Delta-Based Multiple Imputation for MNAR Analysis

eTable 27. Severe Adverse Events

eTable 28. Adverse Events

This supplementary material has been provided by the authors to give readers additional information about their work.

eTable 29. Participants Retention Rate

	Baseline	6 weeks	12 weeks	24 weeks	36 weeks
			Overall		
Completer	257	190	148	106	89
Intervention Drop	0	48	72	92	100
Study Drop	0	19	37	59	68
			Placebo		
Completer	128	100	79	57	47
Intervention Drop	0	20	31	42	46
Study Drop	0	8	18	29	35
			Omega-3		
Completer	129	90	69	49	42
Intervention Drop	0	28	41	50	54
Study Drop	0	11	19	30	33

Legend: eTable 1 further describes the retention of study participants over the 36-week observation period. Similar trends are seen across the treatment arms. More participants initiate or increase antidepressant dose (intervention drop-out) rather than leave the study (study drop-out). Both types of drop-out increase as the study progresses. By study end, 34.6% of participants are still adherent to protocol ("completers", 36.7% and 32.6% in the placebo and treatment arms, respectively).

© 2025 Berger G et al. *JAMA Network Open*.

Joint model results (eTable 2)

eTable 30. Primary Analysis Results From Joint Modeling of the Primary Outcome and Time to Dropout

Study and intervention dropout were considered a single type of event.

Parameter	Estimate	CI	P
	Longitudinal		
(Intercept)	43.64	38.18 to 49.16	< 0.0001
Week	-0.49	-0.58 to -0.4	< 0.0001
Omega-3 treatment	0.77	-1.39 to 2.93	0.49
Age	0.47	0.05 to 0.88	0.029
Female sex	5.58	3.24 to 7.92	< 0.0001
hsCRP - average	-0.39	-2.96 to 2.21	0.76
hsCRP - high	-0.32	-3.57 to 2.77	0.86
Omega3 index	-0.08	-1.05 to 0.9	0.86
baseline AD	1.94	-0.35 to 4.13	0.091
Recurrence	2.57	-0.05 to 5.14	0.053
Week:Omega-3 treatment	-0.04	-0.17 to 0.09	0.55
	Time-to-Event		
Omega-3 treatment	1.22	0.83 to 1.79	0.32
Predicted CDRS-R	1.03	1.01 to 1.05	0.013

Legend: The joint model for the primary analysis additionally included the baseline covariate recurrent episode as it was found to be imbalanced across treatment groups. The time-to-event submodel considered study and intervention dropout to be a single type of event and included the current predicted estimated CDRS-R score as a covariate, but not the current predicted slope. Average CDRS-R score was found to change. However, no evidence was found to indicate a difference in CDRS-R trajectories per treatment arm.

© 2025 Berger G et al. *JAMA Network Open*.

LMM using data as observed (eTable 3-4)

eTable 31. Sensitivity Analysis I (Data as Observed): Adjusted Linear Mixed Model Results for the Relationship Between Treatment Arm and CDRS Slopes, Including Random Intercepts for Participant and Center

This model did not result in convergence messages

Variable	Estimate	X95.confidence.interval	p.value
(Intercept)	41.95	[29.16, 54.74]	< 0.0001
Week	-0.47	[-0.54, -0.40]	< 0.0001
Omega-3 treatment	1.01	[-1.57, 3.59]	0.44
Age	0.46	[-0.27, 1.19]	0.21
Female sex	5.78	[3.17, 8.39]	< 0.0001
hsCRP - average	-0.49	[-3.44, 2.47]	0.75
hsCRP - high	0.86	[-3.09, 4.81]	0.67
Omega3 index	0.10	[-0.98, 1.18]	0.86
baseline AD	1.64	[-0.93, 4.21]	0.21
Recurrence	1.31	[-1.60, 4.22]	0.38
Week:Omega-3 treatment	-0.03	[-0.13, 0.07]	0.52

eTable 32. Sensitivity Analysis I (Data as Observed): Adjusted Linear Mixed Model Results for the Relationship Between Treatment Arm and CDRS Slopes, Including Random Intercepts for Participant and Center and Random Slopes for Participants

Variable	Estimate	X95.confidence.interval	p.value
(Intercept)	43.19	[31.81, 54.56]	< 0.0001
Week	-0.53	[-0.61, -0.45]	< 0.0001
Omega-3 treatment	1.00	[-1.06, 3.06]	0.34
Age	0.46	[-0.19, 1.10]	0.16
Female sex	5.50	[3.19, 7.81]	< 0.0001
hsCRP - average	0.35	[-2.26, 2.95]	0.79
hsCRP - high	0.30	[-3.16, 3.77]	0.86
Omega3 index	-0.06	[-1.01, 0.89]	0.90
baseline AD	0.59	[-1.70, 2.89]	0.61
Recurrence	1.62	[-0.96, 4.20]	0.22
Week:Omega-3 treatment	-0.04	[-0.15, 0.08]	0.51

Legend: This model resulted in conver-gence messages.

© 2025 Berger G et al. *JAMA Network Open*.

LMM using data censored at additional antidepressant use (eTable 5-6)

eTable 33. Sensitivity Analysis II (Censored Observations After AD Use): Adjusted Linear Mixed Model Results for the Relationship Between Treatment Arm and CDRS Slopes, Including Random Intercepts for Participant and Center

Variable	Estimate	X95.confidence.interval	p.value
(Intercept)	43.00	[30.73, 55.26]	< 0.0001
Week	-0.53	[-0.61, -0.45]	< 0.0001
Omega-3 treatment	0.36	[-2.02, 2.74]	0.77
Age	0.55	[-0.15, 1.25]	0.12
Female sex	5.15	[2.61, 7.70]	< 0.0001
hsCRP - average	-0.86	[-3.72, 2.00]	0.56
hsCRP - high	-0.11	[-3.79, 3.56]	0.95
Omega3 index	-0.20	[-1.27, 0.86]	0.71
baseline AD	0.99	[-1.55, 3.52]	0.45
Recurrence	2.25	[-0.58, 5.09]	0.12
Week:Omega-3 treatment	-0.01	[-0.12, 0.10]	0.83

Legend:. This model did not result in convergence messages.

eTable 34. Sensitivity Analysis II (Censored Observations After AD Use): Adjusted LLMI results for the Relationship Between Treatment Arm and CDRS Slopes, Including Random Intercepts for Participant and Center and Random Slopes for Participants

Variable	Estimate	X95.confidence.interval	p.value
(Intercept)	45.19	[33.61, 56.77]	< 0.0001
Week	-0.57	[-0.66, -0.49]	< 0.0001
Omega-3 treatment	0.46	[-1.62, 2.54]	0.66
Age	0.45	[-0.21, 1.10]	0.18
Female sex	4.73	[2.36, 7.10]	< 0.0001
hsCRP - average	-0.26	[-2.92, 2.39]	0.85
hsCRP - high	0.63	[-2.83, 4.09]	0.72
Omega3 index	-0.26	[-1.25, 0.72]	0.60
baseline AD	0.61	[-1.75, 2.98]	0.61
Recurrence	1.48	[-1.17, 4.14]	0.27
Week:Omega-3 treatment	0.01	[-0.12, 0.14]	0.88

Legend: This model resulted in convergence messages.

© 2025 Berger G et al. *JAMA Network Open*.

LMM using per-protocol data

eTable 35. Sensitivity Analysis III (per Protocol Data): Adjusted Linear Mixed Model Results for the Relationship Between Treatment Arm and CDRS Slopes, Including Random Intercepts for Participant and Center

Variable	Estimate	X95.confidence.interval	p.value
(Intercept)	38.15	[20.98, 55.31]	< 0.0001
Week	-0.51	[-0.60, -0.43]	< 0.0001
Omega-3 treatment	-1.27	[-5.10, 2.56]	0.52
Age	0.57	[-0.45, 1.59]	0.27
Female sex	6.84	[2.82, 10.85]	0.0008
hsCRP - average	-3.74	[-8.31, 0.84]	0.11
hsCRP - high	-0.81	[-6.31, 4.69]	0.77
Omega3 index	0.34	[-1.52, 2.19]	0.72
baseline AD	5.53	[1.58, 9.49]	0.006
Recurrence	1.56	[-2.98, 6.10]	0.50
Week:Omega-3 treatment	0.01	[-0.11, 0.13]	0.88

Legend: This model did not result in convergence messages.

eTable 36. Sensitivity Analysis III (per Protocol Data): Adjusted LMM Results for the Relationship Between Treatment Arm and CDRS Slopes, Including Random Intercepts for Participants and Center and Random Slopes for Participants

Variable	Estimate	X95.confidence.interval	p.value
(Intercept)	41.95	[26.12, 57.78]	< 0.0001
Week	-0.55	[-0.65, -0.46]	< 0.0001
Omega-3 treatment	-0.72	[-3.95, 2.51]	0.66
Age	0.36	[-0.58, 1.31]	0.45
Female sex	6.19	[2.49, 9.89]	0.001
hsCRP - average	-3.31	[-7.54, 0.92]	0.13
hsCRP - high	1.12	[-3.96, 6.20]	0.66
Omega3 index	0.34	[-1.37, 2.05]	0.70
baseline AD	5.32	[1.76, 8.88]	0.003
Week:Omega-3 treatment	0.01	[-0.12, 0.14]	0.88

Legend: This model resulted in convergence messages. Unlike the other results, this model does not adjust for recurrence.

© 2025 Berger G et al. *JAMA Network Open*.

LMM using imputed data

eTable 37. Sensitivity Analysis IV: Delta-Based Multiple Imputation for MNAR Analysis

Adjusted linear mixed model results for the relationship between treatment arm and CDRS slopes. The model includes random intercepts for participants

	Estimate	95%-confidence	interval	p-value
Delta = 0	0.97	-1.44	3.38	0.43
25%	1.00	-1.62	3.61	0.46
50%	1.02	-1.86	3.90	0.49
75%	1.04	-2.12	4.21	0.52
100%	1.06	-2.38	4.51	0.55

Legend: This model does not adjust for recurrence.

eTable 38. Remission Status per Visit

The denominator at each visit includes those observed at that visit and those experiencing remission before that visit but exiting the study before that visit, but not those exiting prior to that visit whom had not yet had reached remission.

	6 weeks	12 weeks	24 weeks	36 weeks
Placebo	8/120 (6.67%)	16/109 (14.68%)	22/102 (21.57%)	37/91 (40.66%)
Omega-3	4/118 (3.39%)	7/111 (6.31%)	17/94 (18.09%)	30/91 (32.97%)

Legend: The study definition for remission is a CDRS total score <28 at any point during the 36 weeks. There were 67 participants meeting this criterium (30 in the Omega-3 arm and 37 in the Placebo arm).

eTable 39. Response Status per Visit

The denominator at each visit includes those observed at that visit and those experiencing the response earlier but exiting the study prior to that visit, but not those exiting the study prior to the visit whom had not yet responded..

	6 weeks	12 weeks
Placebo	21/120 (17.50%)	43/110 (39.1%)
Omega-3	20/118 (16.95%)	34/111 (30.6%)

Legend: The study definition for response is a 30% decrease or greater in CDRS total score at any point during the first 12 weeks of the trial. There were 77 participants meeting this criterium (34 in the Omega-3 arm and 43 in the Placebo arm).

eTables 10 and 11 describe remission and response, respectively. The unadjusted numbers presented here are somewhat difficult to interpret due to differing lengths of follow-up time across participants. This problem is dealt with during analysis through the use of multiple imputation. Remission and response are tabularized and visualized over time using cumulative numbers including participants

© 2025 Berger G et al. *JAMA Network Open*.

observed at a given time point or experiencing the outcome prior to exiting the study before that time point.

© 2025 Berger G et al. *JAMA Network Open*.

KidScreen-10

eTable 40. Results From Joint Modeling of the Outcome KIDSCREEN-CAT-10 and Time to Dropout

Study and intervention dropout were considered a single type of event.

Parameter	Estimate	CI	P
Longitudinal			
(Intercept)	36.13	31.55 to 40.71	< 0.0001
Week	0.12	0.07 to 0.18	< 0.0001
Omega-3 treatment	-0.50	-1.77 to 0.79	0.43
Age	-0.34	-0.62 to -0.05	0.02
Female sex	-4.36	-5.72 to -3.02	< 0.0001
hsCRP - average	0.55	-0.96 to 2.07	0.48
hsCRP - high	0.00	-1.98 to 2.02	1.00
Omega3 index	0.44	-0.12 to 0.99	0.11
baseline AD	-0.14	-1.44 to 1.12	0.85
Recurrence	-0.35	-1.81 to 1.15	0.64
Week:Omega-3 treatment	-0.00	-0.08 to 0.07	0.99
Time-to-Event			
Omega-3 treatment	1.26	0.85 to 1.87	0.26
Predicted KidScreen 10	0.97	0.93 to 1.02	0.24

eTable 41. ANOVA of KIDSCREEN-CAT-10 After 36 Weeks, Adjusted for Age, Sex, hsCRP, Antidepressants, ω-3 Index and KIDSCREEN-CAT-10 at Baseline

Only 240 patients included due to missing baseline KIDscreen-10.

	Estimate	95%-confidence interval	p-value
(Intercept)	36.35	[15.82, 56.88]	0.001
Omega-3 treatment	0.91	[-1.56, 3.38]	0.47
Age	-0.4	[-1.19, 0.39]	0.33
Female sex	-2.56	[-6.16, 1.03]	0.17
hsCRP - average	1.8	[-1.68, 5.28]	0.32
hsCRP - high	1.71	[-3.93, 7.34]	0.56
baseline AD	-0.67	[-3.94, 2.60]	0.69
Omega3 index	-0.25	[-1.58, 1.08]	0.71

© 2025 Berger G et al. *JAMA Network Open*.

baseline KIDscreen	0.41	[0.14, 0.68]	0.005
baseline CDRS	-0.1	[-0.25, 0.05]	0.20

.

eTable 42. ANOVA of Self-Reported Quality of Life Measured by the KIDSCREEN-CAT-10 at Week 36, Adjusted for Age, Sex, hsCRP, Antidepressants, ω-3 Index, and KIDSCREEN-CAT-10 at Baseline

Delta-based multiple imputation for MNAR analysis

	Estimate	95%-confidence	interval	p-value
Delta = 0	0.91	-1.61	3.43	0.47
25%	1.59	-1.47	4.66	0.30
50%	2.28	-1.59	6.14	0.25
75%	2.96	-1.84	7.76	0.22
100%	3.64	-2.17	9.46	0.22

© 2025 Berger G et al. *JAMA Network Open*.

DIKJ

eTable 43. Results From Joint Modeling of the Outcome DIKJ and Time to Dropout

Study and intervention dropout were considered a single type of event.

Parameter	Estimate	CI	P
	Longitudinal		
(Intercept)	12.35	7.1 to 17.75	< 0.0001
Week	-0.20	-0.27 to -0.13	< 0.0001
Omega-3 treatment	0.43	-1.51 to 2.36	0.67
Age	0.63	0.25 to 1.01	0.002
Female sex	7.83	5.69 to 9.98	< 0.0001
hsCRP - average	0.53	-1.87 to 2.93	0.66
hsCRP - high	1.05	-2 to 4.03	0.50
Omega3 index	-0.24	-1.11 to 0.64	0.59
baseline AD	0.37	-1.69 to 2.44	0.74
Recurrence	0.16	-2.22 to 2.55	0.90
Week:Omega-3 treatment	-0.02	-0.12 to 0.08	0.74
	Time-to-Event		
Omega-3 treatment	1.24	0.84 to 1.82	0.28
Predicted DIKJ	1.02	1 to 1.04	0.042

© 2025 Berger G et al. *JAMA Network Open*.

eTable 44. ANOVA of DIKJ After 36 Weeks, Adjusted for Age, Sex, hsCRP, Antidepressants, ω-3 Index, and DIKJ at Baseline

Only 246 patients included due to missing baseline DIKJ.

	Estimate	95%-confidence interval	p-value
(Intercept)	-1.74	[-19.79, 16.30]	0.85
Omega-3 treatment	-0.9	[-4.09, 2.29]	0.58
Age	-0.32	[-1.31, 0.67]	0.53
Female sex	2.93	[-2.23, 8.09]	0.27
hsCRP - average	-2.26	[-7.00, 2.48]	0.35
hsCRP - high	-0.41	[-6.51, 5.69]	0.89
baseline AD	4.02	[0.08, 7.97]	0.051
Omega3 index	0.85	[-0.79, 2.50]	0.31
baseline DIKJ	0.44	[0.19, 0.70]	0.001
baseline CDRS	0.13	[-0.07, 0.33]	0.20

.

eTable 45. ANOVA of Depression Severity Measured by DIKJ at Week 36, Adjusted for Age, Sex, hsCRP, Antidepressants, ω-3 Index, and DIKJ at Baseline

Delta-based multiple imputation for MNAR analysis

	Estimate	95%-confidence	interval	p-value
Delta = 0	-0.90	-4.15	2.34	0.58
25%	-0.76	-4.51	3.00	0.69
50%	-0.61	-4.99	3.76	0.78
75%	-0.47	-5.53	4.59	0.85
100%	-0.33	-6.12	5.47	0.91

eTable 46. Cox Proportional Hazards Regression Results for the Relationship Between Treatment Arm and Time to Additional Antidepressant Use

	Hazard Ratio	95%-confidence interval	*p*-value
Omega-3 treatment	1.24	from 0.83 to 1.85	0.30
Age	1.03	from 0.90 to 1.16	0.69
Female sex	1.03	from 0.65 to 1.62	0.91
hsCRP - average	0.99	from 0.60 to 1.63	0.96
hsCRP - high	0.43	from 0.17 to 1.06	0.066
baseline AD	1.49	from 0.99 to 2.25	0.058
Omega3 index	1.02	from 0.84 to 1.24	0.83
Recurrence	1.17	from 0.73 to 1.88	0.51

Legend: A Cox proportional hazards model was also used to explore possible differences after adjusting for age, sex, hsCRP, baseline antidepressant use, and Omega-3 index. The estimate suggests a 24% increase in risk of additional antidepressant use for the Omega-3 arm compared to the placebo arm, however, this estimate is not statistically significant (HR (95% CI): 1.24, (0.83, 1.85), *p*-value = 0.30).

© 2025 Berger G et al. *JAMA Network Open*.

Evaluation of moderating effects of the treatment and different baseline characteristics.

.

eTable 47. Results From Joint Modeling of the Primary Outcome and Time to Dropout, Including an Interaction Term Between Among Treatment Effect, Time, and ω-3 Index

Study and intervention dropout were considered a single type of event

Parameter	Estimate	CI	P
Longitudinal			
(Intercept)	44.82	39.34 to 50.18	< 0.0001
Week	-0.72	-1.14 to -0.32	< 0.0001
Omega-3 treatment	-0.90	-5.93 to 4.12	0.73
Age	0.44	0.04 to 0.85	0.03
Female sex	5.60	3.4 to 7.82	< 0.0001
hsCRP - average	-0.41	-2.93 to 2.08	0.75
hsCRP - high	-0.33	-3.42 to 2.71	0.84
Omega-3 index	-0.27	-1.3 to 0.77	0.59
baseline AD	1.92	-0.18 to 4.04	0.075
Recurrence	2.55	0.13 to 4.94	0.04
Week:Omega-3 treatment	0.43	-0.21 to 1.07	0.19
Omega-3 index:Omega-3 treatment	0.35	-0.76 to 1.48	0.54
Week:Omega-3 index	0.05	-0.04 to 0.14	0.26
Week:Omega-3 index:Omega-3 treatment	-0.10	-0.23 to 0.03	0.14
Time-to-Event Omega-3 treatment			
	1.22	0.83 to 1.78	0.33
Predicted CDRS-R	1.03	1 to 1.05	0.021

© 2025 Berger G et al. *JAMA Network Open*.

eTable 48. Results From Joint Modeling of the Primary Outcome and Time to Dropout, Including an Interaction Term Between Group and Sex

Study and intervention dropout were considered a single type of event.

Parameter	Estimate	CI	P
Longitudinal			
(Intercept)	43.69	38.23 to 49.24	< 0.0001
Week	-0.49	-0.58 to -0.4	< 0.0001
Omega-3 treatment	0.65	-2.46 to 3.72	0.68
Age	0.46	0.05 to 0.87	0.029
Female sex	5.49	2.83 to 8.21	< 0.0001
hsCRP - average	-0.37	-2.88 to 2.17	0.76
hsCRP - high	-0.31	-3.52 to 2.76	0.86
Omega-3 index	-0.08	-1.03 to 0.89	0.87
baseline AD	1.95	-0.23 to 4.1	0.083
Recurrence	2.53	-0.02 to 5.1	0.052
Week:Omega-3 treatment	-0.04	-0.16 to 0.09	0.52
Female sex:Omega-3 treatment	0.22	-3.17 to 3.61	0.89
Time-to-Event			
Omega-3 treatment	1.22	0.83 to 1.79	0.33
Predicted CDRS-R	1.03	1.01 to 1.05	0.011

© 2025 Berger G et al. *JAMA Network Open*.

eTable 49. Results From Joint Modeling of the Primary Outcome and Time to Dropout, Including an Interaction Term Between Group and Before or After COVID-19 Pandemic

(with cut-off date 16/03/2020). Study and intervention dropout were considered a single type of event.

Parameter	Estimate	CI	P
Longitudinal			
(Intercept)	45.06	39.6 to 50.4	< 0.0001
Week	-0.49	-0.58 to -0.4	< 0.0001
Omega-3 treatment	0.19	-2.02 to 2.39	0.86
Age	0.37	-0.03 to 0.76	0.07
Female sex	5.37	3.19 to 7.57	< 0.0001
hsCRP - average	-0.65	-3.12 to 1.82	0.61
hsCRP - high	-0.49	-3.54 to 2.59	0.75
Omega-3 index	-0.19	-1.08 to 0.72	0.67
baseline AD	2.03	-0.09 to 4.11	0.061
Recurrence	2.79	0.39 to 5.21	0.024
Covid-19 (after March 16 2020)	2.26	-0.37 to 4.81	0.091
Week:Omega-3 treatment	-0.04	-0.17 to 0.08	0.50
Covid-19 (after March 16 2020):Omega-3 treatment	1.78	-1.68 to 5.22	0.30
Time-to-Event			
Omega-3 treatment	1.22	0.83 to 1.77	0.33
Predicted CDRS-R	1.02	1 to 1.04	0.032

© 2025 Berger G et al. *JAMA Network Open*.

eTable 50. Results From Joint Modeling of the Primary Outcome and Time to Dropout, Including an Interaction Term Between Group and Influence of COVID-19 Restrictions

(between 16/03/2020 and 01/04/2022). Study and intervention dropout were considered a single type of event.

Parameter	Estimate	CI	P
Longitudinal			
(Intercept)	44.28	38.87 to 49.83	< 0.0001
Week	-0.50	-0.59 to -0.4	< 0.0001
Omega-3 treatment	0.48	-1.74 to 2.72	0.68
Age	0.42	0.01 to 0.82	0.044
Female sex	5.51	3.19 to 7.77	< 0.0001
hsCRP - average	-0.40	-2.86 to 2.07	0.75
hsCRP - high	-0.42	-3.52 to 2.68	0.78
Omega-3 index	-0.11	-1.03 to 0.82	0.81
baseline AD	1.98	-0.13 to 4.14	0.067
Recurrence	2.60	0.17 to 5.04	0.037
Covid restrictions	0.95	-1.49 to 3.33	0.44
Week:Omega-3 treatment	-0.04	-0.17 to 0.09	0.56
Covid restrictions:Omega-3 treatment	0.79	-2.51 to 4.03	0.62
Time-to-Event			
Omega-3 treatment	1.22	0.83 to 1.78	0.34
Predicted CDRS-R	1.02	1 to 1.04	0.027

© 2025 Berger G et al. *JAMA Network Open*.

eTable 51. Results From Joint Modeling of the Primary Outcome and Time to Dropout, Including an Interaction Term Between Group and Anxiety and/or Panic

Study and intervention dropout were considered a single type of event.

Parameter	Estimate	CI	P
Longitudinal			
(Intercept)	44.65	39.13 to 50.08	< 0.0001
Week	-0.49	-0.58 to -0.4	< 0.0001
Omega-3 treatment	0.87	-1.22 to 3	0.41
Age	0.42	0.02 to 0.82	0.039
Female sex	5.48	3.21 to 7.75	< 0.0001
hsCRP - average	-0.36	-2.91 to 2.21	0.78
hsCRP - high	-0.29	-3.45 to 2.83	0.85
Omega-3 index	-0.20	-1.12 to 0.73	0.66
baseline AD	1.87	-0.3 to 4.06	0.09
Recurrence	2.27	-0.21 to 4.81	0.075
Anxiety	4.11	0.42 to 7.82	0.03
Week:Omega-3 treatment	-0.04	-0.16 to 0.08	0.49
Anxiety:Omega-3 treatment	-1.51	-5.98 to 2.98	0.51
Time-to-Event			
Omega-3 treatment	1.22	0.83 to 1.8	0.33
Predicted CDRS-R	1.03	1.01 to 1.05	0.012

Legend: The results of the model with the pre-specified interaction term between treatment effect, time, and omega-3 index is shown in eTable 19. In addition, eTable 20 to Table 23 show results of not prespecified analyses of other moderating effects. No evidence was found, that any of the investigated baseline variables acts as a treatment moderator.

© 2025 Berger G et al. *JAMA Network Open*.

SIQ-Jr

eTable 52. Results From Joint Modeling of the Outcome SIQ-Jr and Time to Study Dropout

Study and intervention dropout were considered a single type of event.

Parameter	Estimate	CI	P
	Longitudinal		
(Intercept)	5.89	-0.01 to 11.92	0.050
Week	-0.32	-0.45 to -0.19	< 0.0001
Omega-3 treatment	1.95	-2.19 to 6.1	0.35
Age	1.10	0.38 to 1.81	0.002
Female sex	9.44	5.2 to 13.65	< 0.0001
hsCRP - average	4.13	-0.17 to 8.59	0.062
hsCRP - high	-0.47	-5.44 to 4.54	0.84
Omega3 index	1.09	-0.96 to 3.18	0.30
baseline AD	3.96	-0.08 to 8.01	0.056
Recurrence	4.19	-0.23 to 8.54	0.063
Week:Omega-3 treatment	-0.08	-0.26 to 0.09	0.36
	Time-to-Event		
Omega-3 treatment	1.26	0.86 to 1.86	0.25
Predicted Jr-SIQ	1.01	1 to 1.02	0.001

eTable 53. Safety Analysis (Censored Observations After AD Use): Adjusted Linear Mixed Model Results for the Relationship Between Treatment Arm and SIQ-Jr Slopes

The model includes random intercepts for participant and center.

Variable	Estimate	X95.confidence.interval	p.value
(Intercept)	8.56	[-19.00, 36.13]	0.54
Week	-0.31	[-0.44, -0.18]	< 0.0001
Omega-3 treatment	1.83	[-3.40, 7.06]	0.49
Age	0.98	[-0.59, 2.55]	0.22
Female sex	8.93	[3.22, 14.64]	0.002
hsCRP - average	4.26	[-2.13, 10.65]	0.19
hsCRP - high	-0.56	[-8.86, 7.74]	0.89
Omega3 index	0.92	[-1.48, 3.32]	0.45
baseline AD	1.05	[-4.60, 6.69]	0.72
Recurrence	2.72	[-3.63, 9.06]	0.40
Week:Omega-3 treatment	-0.09	[-0.28, 0.10]	0.37

Legend: This model did not result in convergence messages.

.

eTable 54. Sensitivity Analysis IV, Delta-Based Multiple Imputation for MNAR Analysis

Adjusted linear mixed model results for the relationship between treatment arm and siq slopes. The model includes random intercepts for participants. This model does not adjust for reoccurrence.

	Estimate	95%-confidence	interval	p-value
Delta = 0	2.00	-1.38	5.38	0.25
25%	2.01	-1.49	5.51	0.26
50%	2.01	-1.65	5.66	0.28
75%	2.01	-1.82	5.83	0.30
100%	2.01	-2.00	6.02	0.33

Adverse events

eTable 55. Severe Adverse Events

SAE Type		Overall	Placebo	Omega-3
n		76	31	45
Death (%)	No	76 (100.0)	31 (100.0)	45 (100.0)
	Yes	0 (0.0)	0 (0.0)	0 (0.0)
Life threat (%)	No	45 (59.2)	16 (51.6)	29 (64.4)
	Yes	31 (40.8)	15 (48.4)	16 (35.6)
Hospitalization (%)	No	18 (23.7)	9 (29.0)	9 (20.0)
	Yes	58 (76.3)	22 (71.0)	36 (80.0)
Disability (%)	No	76 (100.0)	31 (100.0)	45 (100.0)
	Yes	0 (0.0)	0 (0.0)	0 (0.0)
Other (%)	No	73 (96.1)	30 (96.8)	43 (95.6)
	Yes	3 (3.9)	1 (3.2)	2 (4.4)

© 2025 Berger G et al. *JAMA Network Open*.

eTable 56. Adverse Events

		Overall	Placebo	Omega-3
n		115	71	44
Schweregrad (%)	Mild	74 (64.3)	45 (63.4)	29 (65.9)
	Moderate	38 (33.0)	23 (32.4)	15 (34.1)
	Severe	3 (2.6)	3 (4.2)	0 (0.0)
Massnahmen? (%)	No	60 (52.2)	37 (52.1)	23 (52.3)
	Yes	55 (47.8)	34 (47.9)	21 (47.7)
Med. Behandlung? (%)	No	24 (43.6)	17 (50.0)	7 (33.3)
	Yes	31 (56.4)	17 (50.0)	14 (66.7)

Legend eTables 27-28: During the study, there were 191 events in 97 patients: 76 severe adverse events and 115 adverse events. Of the 76 severe adverse events, 31 occurred in the placebo arm and 45 in the Omega-3 arm (Table 33). There were 0 deaths or disabilities, however, 31 events were considered life-threatening and 58 required hospitalization. Four of these were not related to depression or suicidality (breathing problems due to influenza, seizure, paraparesis after lumbar spine trauma, hand fracture). Twenty-eight were suicide attempts. The 3 events categorized as ’Other’ included an increase in CDRS-R for suicidality from 1 to 4, isolation in acute care, and crisis intervention in the Psychiatric Emergency center in Zurich (KJPP). The investigators did not find any severe adverse events to be causally related to study treatment. Of the 115 adverse events, 71 occurred in the placebo arm and 44 in the Omega-3 arm. Most adverse events were considered mild (64.3%). Measures were taken in 55 events (47.8%), of which 31 were treated medically. No causal relationship to study treatment was determined for 88 adverse events, but a connection was possible for 22 adverse events.

© 2025 Berger G et al. *JAMA Network Open*.
