# Supplementary material for: ω-3 Fatty Acids in Pediatric Major Depressive Disorder: A Randomized Clinical Trial
Source: JAMA Netw Open. 2026 Jan 2;9(1):e2548703. doi: 10.1001/jamanetworkopen.2025.48703 (PMC12761337; doi:10.1001/jamanetworkopen.2025.48703)
Supplement: Supplement 3. — Nonauthor Collaborators [file jamanetwopen-e2548703-s003.pdf]

Supplemental Online Content: Nonauthor Collaborators
*First name, last name, and suffix (if applicable) are required and will appear in PubMed.

***Group Name(s): Omega-3 pMDD Study Group**

***First Name and Middle Initial(s)**	***Last Name**	***Suffix (eg, Jr, III)**	Academic Degrees	Institution	Location (city, state/province, country)	Role or Contribution, eg, chair, principal investigator	Group (if more than 1 Group listed in the byline) and/or Subgroup (eg, Steering Committee)
Oliver	Pick		MD	Department of Child and Adolescent	Basel, Switzerland	Clinical Investigator (CI) Basel-Stadt	
Alain	Di Gallo		Prof., MD	Department of Child and Adolescent	Basel, Switzerland	Clinical Investigator (CI) Basel-Stadt	
Stefan	Müller		MD	Child and Adolescent Psychiatric Serv	Baselland, Switzerland	Clinical Investigator (CI) Baselland	
Simone	Heitzer		MSc	Clienia Littenheid	Littenheid, Switzerland	Clinical Investigator (CI) Clienia Littenheid	
Michael	Schmid		MD	Child and Adolescent Psychiatric Out	St. Gallen, Switzerland	Clinical Investigator (CI) St. Gallen	
Ioannis	Christodoulakis		MD	Klinik Sonnenhof	Switzerland	Clinical Investigator (CI) Klinik Sonnenhof	
Edna	Grünblatt		Prof., PhD	Department of Child and Adolescent	Zurich, Switzerland	Clinical Trials Biobank	
Ivan	Hartling		PhD studer	Clinical Chemistry and Biochemistry,	Zurich, Switzerland	Biobank & Biochemistry	
Ester	Osuna		PhD	ETH Zurich, Department of Health Sc	Zurich, Switzerland	Food Scientist	
Jeannine	Baumgartner		PhD	ETH Zurich, Department of Health Sc	Zurich, Switzerland	Food Scientist	
Isabelle	Herter-Aeberli		PhD	ETH Zurich, Department of Health Sc	Zurich, Switzerland	Food Scientist	
Romuald	Brunner		Prof., PhD	University of Regensburg	Regensburg, Germany	Data Monitoring Committee	
Jürgen	Drewe		Prof., PhD	University of Basel	Basel, Switzerland	Data Monitoring Committee	
Julia	Braun			Epidemiology, Biostatistics, and Prev	Zurich, Switzerland	Data Monitoring Committee	
Jenny	Peterson		MSc	Kantonsapotheke Zürich	Zurich, Switzerland	Clinical Trials Pharmacy	
Burkhardt	Seifert		Prof., PhD (	Epidemiology, Biostatistics and Preve	Zurich, Switzerland	Statistician	
