# Supplementary material for: ω-3 Fatty Acids in Pediatric Major Depressive Disorder: A Randomized Clinical Trial
Source: JAMA Netw Open. 2026 Jan 2;9(1):e2548703. doi: 10.1001/jamanetworkopen.2025.48703 (PMC12761337; doi:10.1001/jamanetworkopen.2025.48703)
Supplement: Supplement 4. — Data Sharing Statement [file jamanetwopen-e2548703-s004.pdf]

Data Sharing Statement

Berger. ω-3 Fatty Acids in Pediatric Major Depressive Disorder. *JAMA Netw Open*. Published December 29, 2025. doi:10.1001/jamanetworkopen.2025.48703

Data
Additional Information: ClinicalTrials.gov Identifier: NCT03167307
Data available: Yes
Data types: Deidentified participant data
How to access data: The clinical trial data will be made available at the Swiss National Science Data repository: https://data.snf.ch/grants/grant/166826; researchers whose proposed use of the data has been approved
When available: With publication

Supporting Documents
Document types: Other (please specify)
Additional Information: Statistical analysis plan SAP Statistical report SR
How to access documents: in the supplement of the paper
When available: With publication

Additional Information
Who can access the data: researchers whose proposed use of the data has been approved
Types of analyses: researchers whose proposed use of the data has been approved
Mechanisms of data availability: researchers whose proposed use of the data has been approved (similar to the TADS study)
